# Supplementary material for: A cross-national analysis of childhood predictors of daily smoking in adulthood
Source: Commun Med (Lond). 2025 Jul 12;5:292. doi: 10.1038/s43856-025-01005-3 (PMC12255748; doi:10.1038/s43856-025-01005-3)
Supplement: Supplementary file 2 — Supplementary information [file 43856_2025_1005_MOESM2_ESM.pdf]

Supplementary Information

A cross-national analysis of childhood predictors of daily smoking in adulthood

Sung Joon Jang[1,2], Pedro A. de la Rosa[3], R. Noah Padgett[4,5], Matt Bradshaw[1], Tyler J. VanderWeele[4,5], Byron R. Johnson[1,2,4]

[1] Institute for Studies of Religion, Baylor University, Waco, TX, U.S.A.
[2] School of Public Policy, Pepperdine University, Malibu, CA, U.S.A.
[3] Institute for Culture and Society, Universidad de Navarra, Pamplona, Spain.
[4] Human Flourishing Program, Institute for Quantitative Social Science, Harvard University, Cambridge, MA, U.S.A.
[5] Department of Epidemiology, Harvard T.H. Chan School of Public Health, Boston, MA, U.S.A.

Table of Contents

1. Table S1. Nationally representative descriptive statistics for Argentina: Childhood predictors ... 7
2. Table S2. Nationally representative descriptive statistics for Argentina: Demographic variables ... 8
3. Table S3. Childhood predictors regression for Argentina ... 9
4. Table S4. Sensitivity to unmeasured confounding of childhood predictors in Argentina ... 10
5. Table S5. Nationally representative descriptive statistics for Australia: Childhood predictors ... 11
6. Table S6. Nationally representative descriptive statistics for Australia: Demographic variables ... 12
7. Table S7. Childhood predictors regression for Australia ... 13
8. Table S8. Sensitivity to unmeasured confounding of childhood predictors in Australia ... 14
9. Table S9. Nationally representative descriptive statistics for Brazil: Childhood predictors ... 15
10. Table S10. Nationally representative descriptive statistics for Brazil: Demographic variables ... 16
11. Table S11. Childhood predictors regression for Brazil ... 17
12. Table S12. Sensitivity to unmeasured confounding of childhood predictors in Brazil ... 18
13. Table S13. Nationally representative descriptive statistics for Egypt: Childhood predictors ... 19
14. Table S14. Nationally representative descriptive statistics for Egypt: Demographic variables ... 20
15. Table S15. Childhood predictors regression for Egypt ... 21
16. Table S16. Sensitivity to unmeasured confounding of childhood predictors in Egypt ... 22
17. Table S17. Nationally representative descriptive statistics for Germany: Childhood predictors ... 23
18. Table S18. Nationally representative descriptive statistics for Germany: Demographic variables ... 24
19. Table S19. Childhood predictors regression for Germany ... 25
20. Table S20. Sensitivity to unmeasured confounding of childhood predictors in Germany ... 26
21. Table S21. Nationally representative descriptive statistics for Hong Kong: Childhood predictors ... 27
22. Table S22. Nationally representative descriptive statistics for Hong Kong: Demographic variables ... 28
23. Table S23. Childhood predictors regression for Hong Kong ... 29
24. Table S24. Sensitivity to unmeasured confounding of childhood predictors in Hong Kong ... 30
25. Table S25. Nationally representative descriptive statistics for India: Childhood predictors ... 31
26. Table S26. Nationally representative descriptive statistics for India: Demographic variables ... 32
27. Table S27. Childhood predictors regression for India ... 33
28. Table S28. Sensitivity to unmeasured confounding of childhood predictors in India ... 34
29. Table S29. Nationally representative descriptive statistics for Indonesia: Childhood predictors ... 35
30. Table S30. Nationally representative descriptive statistics for Indonesia: Demographic variables ... 36
31. Table S31. Childhood predictors regression for Indonesia ... 37
32. Table S32. Sensitivity to unmeasured confounding of childhood predictors in Indonesia ... 38
33. Table S33. Nationally representative descriptive statistics for Israel: Childhood predictors ... 39
34. Table S34. Nationally representative descriptive statistics for Israel: Demographic variables ... 40
35. Table S35. Childhood predictors regression for Israel ... 41
36. Table S36. Sensitivity to unmeasured confounding of childhood predictors in Israel ... 42
37. Table S37. Nationally representative descriptive statistics for Japan: Childhood predictors ... 43
38. Table S38. Nationally representative descriptive statistics for Japan: Demographic variables ... 44
39. Table S39. Childhood predictors regression for Japan ... 45

40. Table S40. Sensitivity to unmeasured confounding of childhood predictors in Japan ... 46
41. Table S41. Nationally representative descriptive statistics for Kenya: Childhood predictors ... 47
42. Table S42. Nationally representative descriptive statistics for Kenya: Demographic variables ... 48
43. Table S43. Childhood predictors regression for Kenya ... 49
44. Table S44. Sensitivity to unmeasured confounding of childhood predictors in Kenya ... 50
45. Table S45. Nationally representative descriptive statistics for Mexico: Childhood predictors ... 51
46. Table S46. Nationally representative descriptive statistics for Mexico: Childhood predictors: Demographic variables ... 52
47. Table S47. Childhood predictors regression for Mexico ... 53
48. Table S48. Sensitivity to unmeasured confounding of childhood predictors in Mexico ... 54
49. Table S49. Nationally representative descriptive statistics for Nigeria: Childhood predictors ... 55
50. Table S50. Nationally representative descriptive statistics for Nigeria: Demographic variables ... 56
51. Table S51. Childhood predictors regression for Nigeria ... 57
52. Table S52. Sensitivity to unmeasured confounding of childhood predictors in Nigeria ... 58
53. Table S53. Nationally representative descriptive statistics for Philippines: Childhood predictors ... 59
54. Table S54. Nationally representative descriptive statistics for Philippines: Demographic variables ... 60
55. Table S55. Childhood predictors regression for Philippines ... 61
56. Table S56. Sensitivity to unmeasured confounding of childhood predictors in Philippines ... 62
57. Table S57. Nationally representative descriptive statistics for Poland: Childhood predictors ... 63
58. Table S58. Nationally representative descriptive statistics for Poland: Demographic variables ... 64
59. Table S59. Childhood predictors regression for Poland ... 65
60. Table S60. Sensitivity to unmeasured confounding of childhood predictors in Poland ... 66
61. Table S61. Nationally representative descriptive statistics for South Africa: Childhood predictors ... 67
62. Table S62. Nationally representative descriptive statistics for South Africa: Demographic variables ... 68
63. Table S63. Childhood predictors regression for South Africa ... 69
64. Table S64. Sensitivity to unmeasured confounding of childhood predictors in South Africa ... 70
65. Table S65. Nationally representative descriptive statistics for Spain: Childhood predictors ... 71
66. Table S66. Nationally representative descriptive statistics for Spain: Childhood predictors: Demographic variables ... 72
67. Table S67. Childhood predictors regression for Spain ... 73
68. Table S68. Sensitivity to unmeasured confounding of childhood predictors in Spain ... 74
69. Table S69. Nationally representative descriptive statistics for Sweden: Childhood predictors ... 75
70. Table S70. Nationally representative descriptive statistics for Sweden: Demographic variables ... 76
71. Table S71. Childhood predictors regression for Sweden ... 77
72. Table S72. Sensitivity to unmeasured confounding of childhood predictors in Sweden ... 78
73. Table S73. Nationally representative descriptive statistics for Tanzania: Childhood predictors ... 79
74. Table S74. Nationally representative descriptive statistics for Tanzania: Demographic variables ... 80
75. Table S75. Childhood predictors regression for Tanzania ... 81
76. Table S76. Sensitivity to unmeasured confounding of childhood predictors in Tanzania ... 82
77. Table S77. Nationally representative descriptive statistics for Türkiye: Childhood predictors ... 83
78. Table S78. Nationally representative descriptive statistics for Türkiye: Demographic variables ... 84
79. Table S79. Childhood predictors regression for Türkiye ... 85
80. Table S80. Sensitivity to unmeasured confounding of childhood predictors in Türkiye ... 86
81. Table S81. Nationally representative descriptive statistics for United Kingdom: Childhood predictors ... 87
82. Table S82. Nationally representative descriptive statistics for United Kingdom: Demographic variables ... 88
83. Table S83. Childhood predictors regression for United Kingdom ... 89
84. Table S84. Sensitivity to unmeasured confounding of childhood predictors in United Kingdom ... 90
85. Table S85. Nationally representative descriptive statistics for United States: Childhood predictors ... 91
86. Table S86. Nationally representative descriptive statistics for United States: Demographic variables ... 92
87. Table S87. Childhood predictors regression for United States ... 93
88. Table S88. Sensitivity to unmeasured confounding of childhood predictors in United States ... 94

89. Table S89. Population weighted meta-analysis of regression results (binary). 95
90. Table S90. Population weighted meta-analysis of E-values (binary). 96
91. Table S91. Childhood predictors regression for Argentina 97
92. Table S92. Sensitivity to unmeasured confounding of childhood predictors in Argentina 98
93. Table S93. Childhood predictors regression for Australia 99
94. Table S94. Sensitivity to unmeasured confounding of childhood predictors in Australia 100
95. Table S95. Childhood predictors regression for Brazil 101
96. Table S96. Sensitivity to unmeasured confounding of childhood predictors in Brazil 102
97. Table S97. Childhood predictors regression for Egypt 103
98. Table S98. Sensitivity to unmeasured confounding of childhood predictors in Egypt 104
99. Table S99. Childhood predictors regression for Germany 105
100. Table S100. Sensitivity to unmeasured confounding of childhood predictors in Germany 106
101. Table S101. Childhood predictors regression for Hong Kong 107
102. Table S102. Sensitivity to unmeasured confounding of childhood predictors in Hong Kong 108
103. Table S103. Childhood predictors regression for India 109
104. Table S104. Sensitivity to unmeasured confounding of childhood predictors in India 110
105. Table S105. Childhood predictors regression for Indonesia 111
106. Table S106. Sensitivity to unmeasured confounding of childhood predictors in Indonesia 112
107. Table S107. Childhood predictors regression for Israel 113
108. Table S108. Sensitivity to unmeasured confounding of childhood predictors in Israel 114
109. Table S109. Childhood predictors regression for Japan 115
110. Table S110. Sensitivity to unmeasured confounding of childhood predictors in Japan 116
111. Table S111. Childhood predictors regression for Kenya 117
112. Table S112. Sensitivity to unmeasured confounding of childhood predictors in Kenya 118
113. Table S113. Childhood predictors regression for Mexico 119
114. Table S114. Sensitivity to unmeasured confounding of childhood predictors in Mexico 120
115. Table S115. Childhood predictors regression for Nigeria 121
116. Table S116. Sensitivity to unmeasured confounding of childhood predictors in Nigeria 122
117. Table S117. Childhood predictors regression for Philippines 123
118. Table S118. Sensitivity to unmeasured confounding of childhood predictors in Philippines 124
119. Table S119. Childhood predictors regression for Poland 125
120. Table S120. Sensitivity to unmeasured confounding of childhood predictors in Poland 126
121. Table S121. Childhood predictors regression for South Africa 127
122. Table S122. Sensitivity to unmeasured confounding of childhood predictors in South Africa 128
123. Table S123. Childhood predictors regression for Spain 129
124. Table S124. Sensitivity to unmeasured confounding of childhood predictors in Spain 130
125. Table S125. Childhood predictors regression for Sweden 131
126. Table S126. Sensitivity to unmeasured confounding of childhood predictors in Sweden 132
127. Table S127. Childhood predictors regression for Tanzania 133
128. Table S128. Sensitivity to unmeasured confounding of childhood predictors in Tanzania 134
129. Table S129. Childhood predictors regression for Türkiye 135
130. Table S130. Sensitivity to unmeasured confounding of childhood predictors in Türkiye 136
131. Table S131. Childhood predictors regression for United Kingdom 137
132. Table S132. Sensitivity to unmeasured confounding of childhood predictors in United Kingdom 138
133. Table S133. Childhood predictors regression for United States 139
134. Table S134. Sensitivity to unmeasured confounding of childhood predictors in United States 140
135. Table 135. Random effects meta-analysis of regression of daily cigarette consumption (continuous) on childhood predictors: Total sample (N = 202,898) 141
136. Table S136. Sensitivity of meta-analyzed childhood predictors to unmeasured confounding. 142

137. Table S137. Population weighted meta-analysis of regression results (continuous). 143
138. Table S138. Population weighted meta-analysis of E-values (continuous). 144
139. Table S139. Nationally representative descriptive statistics for Argentina conditional on smokers: Childhood predictors 145
140. Table S140. Nationally representative descriptive statistics for Argentina conditional on smokers: Demographic variables 146
141. Table S141. Childhood predictors regression for Argentina conditional on smokers 147
142. Table S142. Sensitivity to unmeasured confounding of childhood predictors in Argentina conditional on smokers 148
143. Table S143. Nationally representative descriptive statistics for Australia conditional on smokers: Childhood predictors 149
144. Table S144. Nationally representative descriptive statistics for Australia conditional on smokers: Demographic variables 150
145. Table S145. Childhood predictors regression for Australia conditional on smokers 151
146. Table S146. Sensitivity to unmeasured confounding of childhood predictors in Australia conditional on smokers 152
147. Table S147. Nationally representative descriptive statistics for Brazil conditional on smokers: Childhood predictors 153
148. Table S148. Nationally representative descriptive statistics for Brazil conditional on smokers: Demographic variables 154
149. Table S149. Childhood predictors regression for Brazil conditional on smokers 155
150. Table S150. Sensitivity to unmeasured confounding of childhood predictors in Brazil conditional on smokers 156
151. Table S151. Nationally representative descriptive statistics for Egypt conditional on smokers: Childhood predictors 157
152. Table S152. Nationally representative descriptive statistics for Egypt conditional on smokers: Demographic variables 158
153. Table S153. Childhood predictors regression for Egypt conditional on smokers 159
154. Table S154. Sensitivity to unmeasured confounding of childhood predictors in Egypt conditional on smokers 160
155. Table S155. Nationally representative descriptive statistics for Germany conditional on smokers: Childhood predictors 161
156. Table S156. Nationally representative descriptive statistics for Germany conditional on smokers: Demographic variables 162
157. Table S157. Childhood predictors regression for Germany conditional on smokers 163
158. Table S158. Sensitivity to unmeasured confounding of childhood predictors in Germany conditional on smokers 164
159. Table S159. Nationally representative descriptive statistics for Hong Kong conditional on smokers: Childhood predictors 165
160. Table S160. Nationally representative descriptive statistics for Hong Kong conditional on smokers: Demographic variables 166
161. Table S161. Childhood predictors regression for Hong Kong conditional on smokers 167
162. Table S162. Sensitivity to unmeasured confounding of childhood predictors in Hong Kong conditional on smokers 168
163. Table S163. Nationally representative descriptive statistics for India conditional on smokers: Childhood predictors 169
164. Table S164. Nationally representative descriptive statistics for India conditional on smokers: Demographic variables 170
165. Table S165. Childhood predictors regression for India conditional on smokers 171
166. Table S166. Sensitivity to unmeasured confounding of childhood predictors in India conditional on smokers 172
167. Table S167. Nationally representative descriptive statistics for Indonesia conditional on smokers: Childhood predictors 173
168. Table S168. Nationally representative descriptive statistics for Indonesia conditional on smokers: Demographic variables 174
169. Table S169. Childhood predictors regression for Indonesia conditional on smokers 175
170. Table S170. Sensitivity to unmeasured confounding of childhood predictors in Indonesia conditional on smokers 176
171. Table S171. Nationally representative descriptive statistics for Israel conditional on smokers: Childhood predictors 177
172. Table S172. Nationally representative descriptive statistics for Israel conditional on smokers: Demographic variables 178
173. Table S173. Childhood predictors regression for Israel conditional on smokers 179
174. Table S174. Sensitivity to unmeasured confounding of childhood predictors in Israel conditional on smokers 180
175. Table S175. Nationally representative descriptive statistics for Japan conditional on smokers: Childhood predictors 181
176. Table S176. Nationally representative descriptive statistics for Japan conditional on smokers: Demographic variables 182
177. Table S177. Childhood predictors regression for Japan conditional on smokers 183
178. Table S178. Sensitivity to unmeasured confounding of childhood predictors in Japan conditional on smokers 184
179. Table S179. Nationally representative descriptive statistics for Kenya conditional on smokers: Childhood predictors 185

180. Table S180. Nationally representative descriptive statistics for Kenya conditional on smokers: Demographic variables . 186
181. Table S181. Childhood predictors regression for Kenya conditional on smokers ... 187
182. Table S182. Sensitivity to unmeasured confounding of childhood predictors in Kenya conditional on smokers ... 188
183. Table S183. Nationally representative descriptive statistics for Mexico conditional on smokers: Childhood predictors ... 189
184. Table S184. Nationally representative descriptive statistics for Mexico conditional on smokers: Demographic variables 190
185. Table S185. Childhood predictors regression for Mexico conditional on smokers ... 191
186. Table S186. Sensitivity to unmeasured confounding of childhood predictors in Mexico conditional on smokers ... 192
187. Table S187. Nationally representative descriptive statistics for Nigeria conditional on smokers: Childhood predictors... 193
188. Table S188. Nationally representative descriptive statistics for Nigeria conditional on smokers: Demographic variables 194
189. Table S189. Childhood predictors regression for Nigeria conditional on smokers ... 195
190. Table S190. Sensitivity to unmeasured confounding of childhood predictors in Nigeria conditional on smokers ... 196
191. Table S191. Nationally representative descriptive statistics for Philippines conditional on smokers: Childhood predictors 197
192. Table S192. Nationally representative descriptive statistics for Philippines conditional on smokers: Demographic variables 198
193. Table S193. Childhood predictors regression for Philippines conditional on smokers ... 199
194. Table S194. Sensitivity to unmeasured confounding of childhood predictors in Philippines conditional on smokers ... 200
195. Table S195. Nationally representative descriptive statistics for Poland conditional on smokers: Childhood predictors ... 201
196. Table S196. Nationally representative descriptive statistics for Poland conditional on smokers: Demographic variables 202
197. Table S197. Childhood predictors regression for Poland conditional on smokers ... 203
198. Table S198. Sensitivity to unmeasured confounding of childhood predictors in Poland conditional on smokers ... 204
199. Table S199. Nationally representative descriptive statistics for South Africa conditional on smokers: Childhood predictors 205
200. Table S200. Nationally representative descriptive statistics for South Africa conditional on smokers: Demographic variables 206
201. Table S201. Childhood predictors regression for South Africa conditional on smokers ... 207
202. Table S202. Sensitivity to unmeasured confounding of childhood predictors in South Africa conditional on smokers ... 208
203. Table S203. Nationally representative descriptive statistics for Spain conditional on smokers: Childhood predictors ... 209
204. Table S204. Nationally representative descriptive statistics for Spain conditional on smokers: Demographic variables .. 210
205. Table S205. Childhood predictors regression for Spain conditional on smokers ... 211
206. Table S206. Sensitivity to unmeasured confounding of childhood predictors in Spain conditional on smokers ... 212
207. Table S207. Nationally representative descriptive statistics for Sweden conditional on smokers: Childhood predictors . 213
208. Table S208. Nationally representative descriptive statistics for Sweden conditional on smokers: Demographic variables 214
209. Table S209. Childhood predictors regression for Sweden conditional on smokers ... 215
210. Table S210. Sensitivity to unmeasured confounding of childhood predictors in Sweden conditional on smokers ... 216
211. Table S211. Nationally representative descriptive statistics for Tanzania conditional on smokers: Childhood predictors 217
212. Table S212. Nationally representative descriptive statistics for Tanzania conditional on smokers: Demographic variables 218
213. Table S213. Childhood predictors regression for Tanzania conditional on smokers ... 219
214. Table S214. Sensitivity to unmeasured confounding of childhood predictors in Tanzania conditional on smokers ... 220
215. Table S215. Nationally representative descriptive statistics for Türkiye conditional on smokers: Childhood predictors . 221
216. Table S216. Nationally representative descriptive statistics for Türkiye conditional on smokers: Demographic variables 222
217. Table S217. Childhood predictors regression for Türkiye conditional on smokers ... 223
218. Table S218. Sensitivity to unmeasured confounding of childhood predictors in Türkiye conditional on smokers ... 224
219. Table S219. Nationally representative descriptive statistics for United Kingdom conditional on smokers: Childhood predictors 225
220. Table S220. Nationally representative descriptive statistics for United Kingdom conditional on smokers: Demographic variables 226
221. Table S221. Childhood predictors regression for United Kingdom conditional on smokers ... 227

222. Table S222. Sensitivity to unmeasured confounding of childhood predictors in United Kingdom conditional on smokers 228

223. Table S223. Nationally representative descriptive statistics for United States conditional on smokers: Childhood predictors 229

224. Table S224. Nationally representative descriptive statistics for United States conditional on smokers: Demographic variables 230

225. Table S225. Childhood predictors regression for United States conditional on smokers 231

226. Table S226. Sensitivity to unmeasured confounding of childhood predictors in United States conditional on smokers 232

227. Table S227. Population weighted meta-analysis of regression results (continuous). 233

228. Table S228. Population weighted meta-analysis of E-values (continuous). 234

229. Table S229. Summary statistics of daily smoking outcome by country with missingness: Total (N = 202,898) and smoker samples (N = 38,290) 235

230. Figures S1-S27. Forest plots for childhood predictors of daily smoking (binary): Total sample (N = 202,898) 236

231. Figures S28-S54. Forest plots for childhood predictors of daily smoking (continuous): Total sample (N = 202,898) 250

232. Figures S55-S81. Forest plots for childhood predictors of daily smoking (continuous): Smoker sample (N = 38,290) 264

1. **Table S1. Nationally representative descriptive statistics for Argentina: Childhood predictors**

Characteristic	N = 6,724[1]
**Relationship with mother**	
Very good	4,463 (66%)
Somewhat good	1,436 (21%)
Somewhat bad	299 (4.4%)
Very bad	216 (3.2%)
Does not apply	273 (4.1%)
(Missing)	36 (0.5%)
**Relationship with father**	
Very good	3,612 (54%)
Somewhat good	1,537 (23%)
Somewhat bad	440 (6.5%)
Very bad	401 (6.0%)
Does not apply	694 (10%)
(Missing)	39 (0.6%)
**Parent marital status**	
Parents married	4,110 (61%)
Divorced	637 (9.5%)
Parents were never married	1,368 (20%)
One or both parents had died	199 (3.0%)
(Missing)	410 (6.1%)
**Subjective financial status of family growing up**	
Lived comfortably	2,042 (30%)
Got by	2,305 (34%)
Found it difficult	1,789 (27%)
Found it very difficult	569 (8.5%)
(Missing)	19 (0.3%)
**Abuse**	
Yes	1,302 (19%)
No	5,271 (78%)
(Missing)	151 (2.2%)
**Outsider growing up**	
Yes	1,165 (17%)
No	5,458 (81%)
(Missing)	101 (1.5%)
**Self-rated health growing up**	
Excellent	2,402 (36%)
Very good	1,819 (27%)
Good	1,830 (27%)
Fair	505 (7.5%)
Poor	156 (2.3%)
(Missing)	12 (0.2%)
**Immigration status**	
Born in this country	6,346 (94%)
Born in another country	348 (5.2%)
(Missing)	29 (0.4%)
**Age 12 religious service attendance**	
At least 1/week	2,601 (39%)
1-3/month	1,204 (18%)
<1/month	1,059 (16%)
Never	1,808 (27%)
(Missing)	53 (0.8%)

2. **Table S2. Nationally representative descriptive statistics for Argentina: Demographic variables**

Characteristic	N = 6,724[1]
**Year of birth**	
1998-2005; age 18-24	1,108 (16%)
1988-1998; age 25-34	1,527 (23%)
1978-1988; age 35-44	1,279 (19%)
1968-1978; age 45-54	1,074 (16%)
1958-1968; age 55-64	871 (13%)
1948-1958; age 65-74	590 (8.8%)
1938-1948; age 75-84	247 (3.7%)
1938 or earlier; age 85+	29 (0.4%)
(Missing)	0 (0%)
**Gender**	
Male	3,143 (47%)
Female	3,542 (53%)
Other	21 (0.3%)
(Missing)	18 (0.3%)
**Religious affiliation**	
Christianity	5,805 (86%)
Islam	11 (0.2%)
Hinduism	2 (<0.1%)
Buddhism	3 (<0.1%)
Judaism	51 (0.8%)
Sikhism	5 (<0.1%)
Baha'i	0 (0%)
Jainism	0 (0%)
Shinto	0 (0%)
Taoism	1 (<0.1%)
Confucianism	0 (0%)
Primal, Animist, or Folk religion	17 (0.2%)
Spiritism	0 (0%)
Umbanda, Candomble, and other African-derived religions	0 (0%)
Chinese folk/traditional religion	0 (0%)
Some other religion	10 (0.2%)
No religion/Atheist/Agnostic	697 (10%)
(Missing)	122 (1.8%)
**Race/Ethnicity**	
Asian	43 (0.6%)
Black	95 (1.4%)
Indigenous	129 (1.9%)
Mestizo(a)	1,801 (27%)
Mullato(a)	75 (1.1%)
Other	104 (1.5%)
White	3,406 (51%)
(Missing)	1,070 (16%)

[1]n (%)

3. **Table S3. Childhood predictors regression for Argentina**

Variable	Category	Risk-Ratio	RR 95% CI	Global p-value
Relationship with mother	(Ref: Very bad/somewhat bad)			0.727
	Very good/somewhat good	0.98	(0.82,1.18)	
Relationship with father	(Ref: Very bad/somewhat bad)			0.849
	Very good/somewhat good	1.01	(0.87,1.16)	
Parent marital status	(Ref: Parents married)			0.047
	Divorced	1.22	(1.04,1.44)	
	Parents were never married	1.11	(0.97,1.28)	
	One or both parents had died	1.02	(0.76,1.36)	
Subjective financial status of family growing up	(Ref: Got by)			0.238
	Lived comfortably	1.03	(0.91,1.16)	
	Found it difficult	1.12	(0.99,1.26)	
	Found it very difficult	1.16	(0.95,1.40)	
Abuse	(Ref: No)			0.137
	Yes	1.09	(0.97,1.24)	
Outsider growing up	(Ref: No)			0.011
	Yes	1.18	(1.04,1.35)	
Self-rated health growing up	(Ref: Good)			0.064
	Excellent	1.15	(1.01,1.31)	
	Very good	0.99	(0.86,1.14)	
	Fair	0.97	(0.78,1.21)	
	Poor	1.04	(0.75,1.44)	
Immigration status	(Ref: Born in this country)			0.005
	Born in another country	0.67	(0.51,0.89)	
Age 12 religious service attendance	(Ref: Never)			0.901
	At least 1/week	0.98	(0.86,1.11)	
	1-3/month	1.02	(0.88,1.19)	
	< 1/month	0.98	(0.84,1.13)	
Year of birth	(Ref: 1998-2005; age: 18-24)			7.63e-07
	1988-1998; age 25-34	1.32	(1.12,1.56)	
	1978-1988; age 35-44	1.39	(1.17,1.64)	
	1968-1978; age 45-54	1.14	(0.95,1.38)	
	1958-1968; age 55-64	1.10	(0.89,1.36)	
	1948-1957; age 65-74	1.03	(0.79,1.34)	
	1938-1948; age 75-84	0.39	(0.21,0.73)	
	1938 or earlier; 85 or older	0.47	(0.09,2.42)	
Gender	(Ref: Male)			0.003
	Female	0.84	(0.76,0.94)	
	Other	0.55	(0.23,1.34)	
Religious affiliation	(Ref: No religion/Atheist/Agnostic)			0.343
	Christianity	0.92	(0.79,1.09)	
	Collapsed affiliations with prevalence<3%	1.13	(0.77,1.64)	
Race/ethnicity	(Ref: Plurality group)			0.468
	Non-plurality groups	1.03	(0.91,1.16)	

4. Table S4. Sensitivity to unmeasured confounding of childhood predictors in Argentina

Variable	Category	E-value for Estimate	E-value for 95% CI
Relationship with mother	(Ref: Very bad/somewhat bad)		
	Very good/somewhat good	1.15	1.00
Relationship with father	(Ref: Very bad/somewhat bad)		
	Very good/somewhat good	1.10	1.00
Parent marital status	(Ref: Parents married)		
	Divorced	1.74	1.23
	Parents were never married	1.47	1.00
	One or both parents had died	1.16	1.00
Subjective financial status of family growing up	(Ref: Got by)		
	Lived comfortably	1.21	1.00
	Found it difficult	1.48	1.00
	Found it very difficult	1.58	1.00
Abuse	(Ref: No)		
	Yes	1.42	1.00
Outsider growing up	(Ref: No)		
	Yes	1.65	1.23
Self-rated health growing up	(Ref: Good)		
	Excellent	1.56	1.12
	Very good	1.11	1.00
	Fair	1.21	1.00
	Poor	1.24	1.00
Immigration status	(Ref: Born in this country)		
	Born in another country	2.34	1.49
Age 12 religious service attendance	(Ref: Never)		
	At least 1/week	1.18	1.00
	1-3/month	1.18	1.00
	< 1/month	1.17	1.00
Year of birth	(Ref: 1998-2005; age: 18-24)		
	1988-1998; age 25-34	1.97	1.48
	1978-1988; age 35-44	2.12	1.62
	1968-1978; age 45-54	1.55	1.00
	1958-1968; age 55-64	1.44	1.00
	1948-1957; age 65-74	1.21	1.00
	1938-1948; age 75-84	4.53	2.09
	1938 or earlier; 85 or older	3.70	1.00
Gender	(Ref: Male)		
	Female	1.65	1.34
	Other	3.03	1.00
Religious affiliation	(Ref: No religion/Atheist/Agnostic)		
	Christianity	1.38	1.00
	Collapsed affiliations with prevalence<3%	1.51	1.00
Race/ethnicity	(Ref: Plurality group)		
	Non-plurality groups	1.20	1.00

5. **Table S5. Nationally representative descriptive statistics for Australia: Childhood predictors**

**Characteristic**	**N = 3,844**[1]
**Relationship with mother**	
Very good	2,554 (66%)
Somewhat good	925 (24%)
Somewhat bad	218 (5.7%)
Very bad	107 (2.8%)
Does not apply	32 (0.8%)
(Missing)	7 (0.2%)
**Relationship with father**	
Very good	2,032 (53%)
Somewhat good	1,144 (30%)
Somewhat bad	315 (8.2%)
Very bad	196 (5.1%)
Does not apply	148 (3.9%)
(Missing)	9 (0.2%)
**Parent marital status**	
Parents married	3,048 (79%)
Divorced	462 (12%)
Parents were never married	187 (4.9%)
One or both parents had died	96 (2.5%)
(Missing)	52 (1.4%)
**Subjective financial status of family growing up**	
Lived comfortably	1,756 (46%)
Got by	1,496 (39%)
Found it difficult	422 (11%)
Found it very difficult	154 (4.0%)
(Missing)	16 (0.4%)
**Abuse**	
Yes	995 (26%)
No	2,790 (73%)
(Missing)	59 (1.5%)
**Outsider growing up**	
Yes	756 (20%)
No	3,062 (80%)
(Missing)	26 (0.7%)
**Self-rated health growing up**	
Excellent	1,736 (45%)
Very good	1,087 (28%)
Good	603 (16%)
Fair	308 (8.0%)
Poor	106 (2.8%)
(Missing)	4 (<0.1%)
**Immigration status**	
Born in this country	2,953 (77%)
Born in another country	885 (23%)
(Missing)	6 (0.2%)
**Age 12 religious service attendance**	
At least 1/week	1,362 (35%)
1-3/month	486 (13%)
<1/month	600 (16%)
Never	1,307 (34%)
(Missing)	90 (2.3%)

6. Table S6. Nationally representative descriptive statistics for Australia: Demographic variables

Characteristic	N = 3,844[1]
**Year of birth**	
1998-2005; age 18-24	345 (9.0%)
1988-1998; age 25-34	586 (15%)
1978-1988; age 35-44	681 (18%)
1968-1978; age 45-54	650 (17%)
1958-1968; age 55-64	652 (17%)
1948-1958; age 65-74	522 (14%)
1938-1948; age 75-84	359 (9.3%)
1938 or earlier; age 85+	48 (1.3%)
(Missing)	2 (<0.1%)
**Gender**	
Male	1,861 (48%)
Female	1,941 (50%)
Other	36 (0.9%)
(Missing)	6 (0.2%)
**Religious affiliation**	
Christianity	2,678 (70%)
Islam	48 (1.2%)
Hinduism	39 (1.0%)
Buddhism	16 (0.4%)
Judaism	29 (0.8%)
Sikhism	6 (0.2%)
Baha'i	5 (0.1%)
Jainism	0 (0%)
Shinto	0 (0%)
Taoism	1 (<0.1%)
Confucianism	0 (0%)
Primal, Animist, or Folk religion	4 (<0.1%)
Spiritism	0 (0%)
Umbanda, Candomble, and other African-derived religions	0 (0%)
Chinese folk/traditional religion	0 (0%)
Some other religion	8 (0.2%)
No religion/Atheist/Agnostic	990 (26%)
(Missing)	21 (0.5%)
**Race/Ethnicity**	
Aboriginal	53 (1.4%)
Australian	1,946 (51%)
Australian British/European	1,047 (27%)
Chinese	75 (1.9%)
Indian	58 (1.5%)
Japanese	1 (<0.1%)
Malay	11 (0.3%)
New Zealander	91 (2.4%)
Other	163 (4.2%)
Other European	357 (9.3%)
Russian	7 (0.2%)
Samoan	4 (0.1%)
Sinhalese	1 (<0.1%)
Spanish	2 (<0.1%)
Sri Lankan Moor	1 (<0.1%)
Sri Lankan Tamil	7 (0.2%)
Vietnamese	7 (0.2%)
(Missing)	14 (0.4%)

7. **Table S7. Childhood predictors regression for Australia**

Variable	Category	Risk-Ratio	RR 95% CI	Global p-value
Relationship with mother	(Ref: Very bad/somewhat bad)			0.529
	Very good/somewhat good	0.89	(0.62,1.28)	
Relationship with father	(Ref: Very bad/somewhat bad)			0.458
	Very good/somewhat good	0.88	(0.61,1.26)	
Parent marital status	(Ref: Parents married)			0.069
	Divorced	1.58	(1.11,2.24)	
	Parents were never married	1.50	(0.86,2.63)	
	One or both parents had died	1.28	(0.64,2.57)	
Subjective financial status of family growing up	(Ref: Got by)			0.710
	Lived comfortably	0.86	(0.67,1.12)	
	Found it difficult	0.99	(0.69,1.42)	
	Found it very difficult	0.90	(0.50,1.63)	
Abuse	(Ref: No)			0.235
	Yes	1.19	(0.88,1.60)	
Outsider growing up	(Ref: No)			0.001
	Yes	1.63	(1.21,2.18)	
Self-rated health growing up	(Ref: Good)			0.302
	Excellent	1.31	(0.90,1.91)	
	Very good	1.32	(0.88,1.96)	
	Fair	1.04	(0.61,1.76)	
	Poor	1.74	(0.97,3.15)	
Immigration status	(Ref: Born in this country)			0.221
	Born in another country	0.81	(0.57,1.15)	
Age 12 religious service attendance	(Ref: Never)			0.708
	At least 1/week	0.83	(0.60,1.15)	
	1-3/month	0.86	(0.57,1.31)	
	< 1/month	0.93	(0.66,1.33)	
Year of birth	(Ref: 1998-2005; age: 18-24)			1.39e-04
	1988-1998; age 25-34	2.08	(1.03,4.23)	
	1978-1988; age 35-44	2.51	(1.27,4.98)	
	1968-1978; age 45-54	2.64	(1.33,5.24)	
	1958-1968; age 55-64	2.79	(1.42,5.50)	
	1948-1957; age 65-74	1.42	(0.68,2.94)	
	1938-1948; age 75-84	0.87	(0.36,2.12)	
	1938 or earlier; 85 or older	1.36	(0.37,5.05)	
Gender	(Ref: Male)			0.002
	Female	0.65	(0.52,0.83)	
	Other	0.68	(0.26,1.80)	
Religious affiliation	(Ref: No religion/Atheist/Agnostic)			0.245
	Christianity	0.80	(0.60,1.06)	
	Collapsed affiliations with prevalence<3%	0.61	(0.24,1.57)	
Race/ethnicity	(Ref: Plurality group)			0.674
	Non-plurality groups	0.97	(0.74,1.27)	

8. **Table S8. Sensitivity to unmeasured confounding of childhood predictors in Australia**

Variable	Category	E-value for Estimate	E-value for 95% CI
Relationship with mother	(Ref: Very bad/somewhat bad)		
	Very good/somewhat good	1.50	1.00
Relationship with father	(Ref: Very bad/somewhat bad)		
	Very good/somewhat good	1.54	1.00
Parent marital status	(Ref: Parents married)		
	Divorced	2.53	1.47
	Parents were never married	2.37	1.00
	One or both parents had died	1.87	1.00
Subjective financial status of family growing up	(Ref: Got by)		
	Lived comfortably	1.58	1.00
	Found it difficult	1.13	1.00
	Found it very difficult	1.45	1.00
Abuse	(Ref: No)		
	Yes	1.66	1.00
Outsider growing up	(Ref: No)		
	Yes	2.64	1.72
Self-rated health growing up	(Ref: Good)		
	Excellent	1.95	1.00
	Very good	1.96	1.00
	Fair	1.24	1.00
	Poor	2.88	1.00
Immigration status	(Ref: Born in this country)		
	Born in another country	1.79	1.00
Age 12 religious service attendance	(Ref: Never)		
	At least 1/week	1.69	1.00
	1-3/month	1.58	1.00
	< 1/month	1.35	1.00
Year of birth	(Ref: 1998-2005; age: 18-24)		
	1988-1998; age 25-34	3.59	1.19
	1978-1988; age 35-44	4.47	1.85
	1968-1978; age 45-54	4.72	1.99
	1958-1968; age 55-64	5.03	2.19
	1948-1957; age 65-74	2.18	1.00
	1938-1948; age 75-84	1.55	1.00
	1938 or earlier; 85 or older	2.05	1.00
Gender	(Ref: Male)		
	Female	2.43	1.70
	Other	2.30	1.00
Religious affiliation	(Ref: No religion/Atheist/Agnostic)		
	Christianity	1.81	1.00
	Collapsed affiliations with prevalence<3%	2.64	1.00
Race/ethnicity	(Ref: Plurality group)		
	Non-plurality groups	1.22	1.00

9. **Table S9. Nationally representative descriptive statistics for Brazil: Childhood predictors**

Characteristic	N = 13,204[1]
**Relationship with mother**	
Very good	8,369 (63%)
Somewhat good	3,559 (27%)
Somewhat bad	483 (3.7%)
Very bad	214 (1.6%)
Does not apply	507 (3.8%)
(Missing)	73 (0.6%)
**Relationship with father**	
Very good	6,364 (48%)
Somewhat good	3,654 (28%)
Somewhat bad	1,035 (7.8%)
Very bad	756 (5.7%)
Does not apply	1,303 (9.9%)
(Missing)	93 (0.7%)
**Parent marital status**	
Parents married	8,546 (65%)
Divorced	1,384 (10%)
Parents were never married	1,985 (15%)
One or both parents had died	508 (3.8%)
(Missing)	781 (5.9%)
**Subjective financial status of family growing up**	
Lived comfortably	4,998 (38%)
Got by	4,616 (35%)
Found it difficult	2,484 (19%)
Found it very difficult	1,027 (7.8%)
(Missing)	79 (0.6%)
**Abuse**	
Yes	2,606 (20%)
No	10,147 (77%)
(Missing)	451 (3.4%)
**Outsider growing up**	
Yes	1,659 (13%)
No	11,234 (85%)
(Missing)	311 (2.4%)
**Self-rated health growing up**	
Excellent	5,312 (40%)
Very good	3,392 (26%)
Good	2,873 (22%)
Fair	1,368 (10%)
Poor	228 (1.7%)
(Missing)	30 (0.2%)
**Immigration status**	
Born in this country	12,688 (96%)
Born in another country	153 (1.2%)
(Missing)	363 (2.7%)
**Age 12 religious service attendance**	
At least 1/week	6,306 (48%)
1-3/month	2,491 (19%)
<1/month	2,629 (20%)
Never	1,707 (13%)
(Missing)	71 (0.5%)

10. Table S10. Nationally representative descriptive statistics for Brazil: Demographic variables

Characteristic	N = 13,204[1]
**Year of birth**	
1998-2005; age 18-24	1,986 (15%)
1988-1998; age 25-34	2,916 (22%)
1978-1988; age 35-44	2,840 (22%)
1968-1978; age 45-54	2,271 (17%)
1958-1968; age 55-64	1,805 (14%)
1948-1958; age 65-74	1,076 (8.1%)
1938-1948; age 75-84	267 (2.0%)
1938 or earlier; age 85+	44 (0.3%)
(Missing)	0 (0%)
**Gender**	
Male	6,320 (48%)
Female	6,820 (52%)
Other	35 (0.3%)
(Missing)	30 (0.2%)
**Religious affiliation**	
Christianity	11,403 (86%)
Islam	15 (0.1%)
Hinduism	1 (<0.1%)
Buddhism	27 (0.2%)
Judaism	40 (0.3%)
Sikhism	0 (0%)
Baha'i	1 (<0.1%)
Jainism	4 (<0.1%)
Shinto	4 (<0.1%)
Taoism	1 (<0.1%)
Confucianism	7 (<0.1%)
Primal, Animist, or Folk religion	17 (0.1%)
Spiritism	336 (2.5%)
Umbanda, Candomble, and other African-derived religions	262 (2.0%)
Chinese folk/traditional religion	0 (0%)
Some other religion	87 (0.7%)
No religion/Atheist/Agnostic	908 (6.9%)
(Missing)	94 (0.7%)
**Race/Ethnicity**	
Amarela	238 (1.8%)
Branca	5,169 (39%)
Indígena	131 (1.0%)
Other	61 (0.5%)
Parda	5,125 (39%)
Preta	1,615 (12%)
(Missing)	865 (6.6%)

[1]n (%)

11. Table S11. Childhood predictors regression for Brazil

Variable	Category	Risk-Ratio	RR 95% CI	Global p-value
Relationship with mother	(Ref: Very bad/somewhat bad)			0.002
	Very good/somewhat good	0.78	(0.67,0.92)	
Relationship with father	(Ref: Very bad/somewhat bad)			0.035
	Very good/somewhat good	0.88	(0.78,1.00)	
Parent marital status	(Ref: Parents married)			1.08e-06
	Divorced	1.40	(1.23,1.58)	
	Parents were never married	1.12	(0.98,1.28)	
	One or both parents had died	0.96	(0.75,1.22)	
Subjective financial status of family growing up	(Ref: Got by)			0.640
	Lived comfortably	1.00	(0.91,1.11)	
	Found it difficult	0.93	(0.82,1.06)	
	Found it very difficult	1.00	(0.83,1.21)	
Abuse	(Ref: No)			0.007
	Yes	1.16	(1.03,1.30)	
Outsider growing up	(Ref: No)			7.20e-03
	Yes	1.29	(1.15,1.45)	
Self-rated health growing up	(Ref: Good)			0.001
	Excellent	1.22	(1.07,1.38)	
	Very good	1.04	(0.91,1.20)	
	Fair	0.94	(0.79,1.12)	
	Poor	0.94	(0.65,1.35)	
Immigration status	(Ref: Born in this country)			0.671
	Born in another country	1.09	(0.73,1.63)	
Age 12 religious service attendance	(Ref: Never)			0.093
	At least 1/week	0.97	(0.83,1.13)	
	1-3/month	1.12	(0.95,1.32)	
	< 1/month	0.99	(0.84,1.16)	
Year of birth	(Ref: 1998-2005; age: 18-24)			0.000
	1988-1998; age 25-34	1.84	(1.57,2.15)	
	1978-1988; age 35-44	2.12	(1.82,2.47)	
	1968-1978; age 45-54	2.10	(1.77,2.48)	
	1958-1968; age 55-64	2.47	(2.06,2.95)	
	1948-1957; age 65-74	1.99	(1.54,2.58)	
	1938-1948; age 75-84	0.44	(0.16,1.20)	
	1938 or earlier; 85 or older	1.55	(0.47,5.05)	
Gender	(Ref: Male)			1.08e-12
	Female	0.70	(0.64,0.77)	
	Other	0.78	(0.41,1.51)	
Religious affiliation	(Ref: No religion/Atheist/Agnostic)			1.14e-11
	Christianity	0.86	(0.72,1.02)	
	Collapsed affiliations with prevalence<3%	1.54	(1.26,1.88)	
Race/ethnicity	(Ref: Plurality group)			0.688
	Non-plurality groups	0.99	(0.90,1.10)	

12. Table S12. Sensitivity to unmeasured confounding of childhood predictors in Brazil

Variable	Category	E-value for Estimate	E-value for 95% CI
Relationship with mother	(Ref: Very bad/somewhat bad)		
	Very good/somewhat good	1.88	1.41
Relationship with father	(Ref: Very bad/somewhat bad)		
	Very good/somewhat good	1.52	1.06
Parent marital status	(Ref: Parents married)		
	Divorced	2.14	1.77
	Parents were never married	1.48	1.00
	One or both parents had died	1.26	1.00
Subjective financial status of family growing up	(Ref: Got by)		
	Lived comfortably	1.07	1.00
	Found it difficult	1.36	1.00
	Found it very difficult	1.02	1.00
Abuse	(Ref: No)		
	Yes	1.59	1.22
Outsider growing up	(Ref: No)		
	Yes	1.90	1.55
Self-rated health growing up	(Ref: Good)		
	Excellent	1.73	1.36
	Very good	1.26	1.00
	Fair	1.33	1.00
	Poor	1.33	1.00
Immigration status	(Ref: Born in this country)		
	Born in another country	1.40	1.00
Age 12 religious service attendance	(Ref: Never)		
	At least 1/week	1.20	1.00
	1-3/month	1.49	1.00
	< 1/month	1.13	1.00
Year of birth	(Ref: 1998-2005; age: 18-24)		
	1988-1998; age 25-34	3.08	2.52
	1978-1988; age 35-44	3.66	3.04
	1968-1978; age 45-54	3.62	2.95
	1958-1968; age 55-64	4.37	3.54
	1948-1957; age 65-74	3.40	2.45
	1938-1948; age 75-84	3.96	1.00
	1938 or earlier; 85 or older	2.47	1.00
Gender	(Ref: Male)		
	Female	2.20	1.91
	Other	1.86	1.00
Religious affiliation	(Ref: No religion/Atheist/Agnostic)		
	Christianity	1.60	1.00
	Collapsed affiliations with prevalence<3%	2.45	1.82
Race/ethnicity	(Ref: Plurality group)		
	Non-plurality groups	1.08	1.00

13. **Table S13. Nationally representative descriptive statistics for Egypt: Childhood predictors**

Characteristic	N = 4,729[1]
**Relationship with mother**	
Very good	4,110 (87%)
Somewhat good	505 (11%)
Somewhat bad	21 (0.4%)
Very bad	10 (0.2%)
Does not apply	83 (1.8%)
(Missing)	0 (0%)
**Relationship with father**	
Very good	3,713 (79%)
Somewhat good	683 (14%)
Somewhat bad	56 (1.2%)
Very bad	30 (0.6%)
Does not apply	233 (4.9%)
(Missing)	14 (0.3%)
**Parent marital status**	
Parents married	4,049 (86%)
Divorced	131 (2.8%)
Parents were never married	9 (0.2%)
One or both parents had died	485 (10%)
(Missing)	55 (1.2%)
**Subjective financial status of family growing up**	
Lived comfortably	1,251 (26%)
Got by	2,352 (50%)
Found it difficult	857 (18%)
Found it very difficult	268 (5.7%)
(Missing)	1 (<0.1%)
**Abuse**	
Yes	405 (8.6%)
No	4,293 (91%)
(Missing)	30 (0.6%)
**Outsider growing up**	
Yes	260 (5.5%)
No	4,456 (94%)
(Missing)	13 (0.3%)
**Self-rated health growing up**	
Excellent	2,687 (57%)
Very good	1,174 (25%)
Good	497 (11%)
Fair	265 (5.6%)
Poor	106 (2.2%)
(Missing)	1 (<0.1%)
**Immigration status**	
Born in this country	4,713 (100%)
Born in another country	16 (0.3%)
(Missing)	1 (<0.1%)
**Age 12 religious service attendance**	
At least 1/week	2,307 (49%)
1-3/month	570 (12%)
<1/month	629 (13%)
Never	1,165 (25%)
(Missing)	57 (1.2%)

14. **Table S14. Nationally representative descriptive statistics for Egypt: Demographic variables**

Characteristic	N = 4,729[1]
**Year of birth**	
1998-2005; age 18-24	960 (20%)
1988-1998; age 25-34	1,296 (27%)
1978-1988; age 35-44	1,016 (21%)
1968-1978; age 45-54	706 (15%)
1958-1968; age 55-64	579 (12%)
1948-1958; age 65-74	156 (3.3%)
1938-1948; age 75-84	15 (0.3%)
1938 or earlier; age 85+	2 (<0.1%)
(Missing)	0 (0%)
**Gender**	
Male	2,394 (51%)
Female	2,334 (49%)
Other	0 (0%)
(Missing)	0 (<0.1%)
**Religious affiliation**	
Christianity	123 (2.6%)
Islam	4,602 (97%)
Hinduism	0 (0%)
Buddhism	0 (0%)
Judaism	0 (0%)
Sikhism	0 (0%)
Baha'i	0 (0%)
Jainism	1 (<0.1%)
Shinto	0 (0%)
Taoism	0 (<0.1%)
Confucianism	0 (0%)
Primal, Animist, or Folk religion	0 (0%)
Spiritism	0 (0%)
Umbanda, Candomble, and other African-derived religions	0 (0%)
Chinese folk/traditional religion	0 (0%)
Some other religion	0 (0%)
No religion/Atheist/Agnostic	0 (0%)
(Missing)	3 (<0.1%)
**Race/Ethnicity**	
Arab	4,585 (97%)
Bedouin Arab	4 (<0.1%)
Greek	1 (<0.1%)
Nubian	27 (0.6%)
Turkish	9 (0.2%)
(Missing)	102 (2.2%)

[1]n (%)

15. Table S15. Childhood predictors regression for Egypt

Variable	Category	Risk-Ratio	RR 95% CI	Global p-value
Relationship with mother	(Ref: Very bad/somewhat bad)			0.099
	Very good/somewhat good	1.48	(0.93,2.33)	
Relationship with father	(Ref: Very bad/somewhat bad)			0.019
	Very good/somewhat good	0.73	(0.56,0.96)	
Parent marital status	(Ref: Parents married)			0.092
	Divorced	1.19	(0.86,1.65)	
	Parents were never married	0.66	(0.13,3.29)	
	One or both parents had died	1.23	(1.01,1.50)	
Subjective financial status of family growing up	(Ref: Got by)			0.671
	Lived comfortably	0.99	(0.88,1.13)	
	Found it difficult	1.08	(0.91,1.28)	
	Found it very difficult	1.10	(0.85,1.43)	
Abuse	(Ref: No)			0.021
	Yes	1.22	(1.03,1.43)	
Outsider growing up	(Ref: No)			0.836
	Yes	0.98	(0.77,1.24)	
Self-rated health growing up	(Ref: Good)			0.353
	Excellent	0.97	(0.78,1.19)	
	Very good	1.03	(0.82,1.30)	
	Fair	0.78	(0.56,1.09)	
	Poor	0.95	(0.64,1.41)	
Immigration status	(Ref: Born in this country)			0.665
	Born in another country	1.02	(0.32,3.25)	
Age 12 religious service attendance	(Ref: Never)			0.348
	At least 1/week	0.88	(0.75,1.04)	
	1-3/month	0.85	(0.66,1.10)	
	< 1/month	0.98	(0.77,1.25)	
Year of birth	(Ref: 1998-2005; age: 18-24)			7.77e-16
	1988-1998; age 25-34	1.28	(1.08,1.51)	
	1978-1988; age 35-44	1.14	(0.94,1.38)	
	1968-1978; age 45-54	1.10	(0.89,1.35)	
	1958-1968; age 55-64	1.14	(0.90,1.45)	
	1948-1957; age 65-74	0.75	(0.50,1.13)	
	1938-1948; age 75-84	0.27	(0.04,1.92)	
	1938 or earlier; 85 or older	0.00	(0.00,0.00)	
Gender	(Ref: Male)			0.000
	Female	0.01	(0.01,0.02)	
Religious affiliation	(Ref: Islam)			0.151
	Collapsed affiliations with prevalence<3%	0.64	(0.35,1.17)	
Race/ethnicity	(Ref: Plurality group)			0.708
	Non-plurality groups	1.07	(0.76,1.51)	

16. Table S16. Sensitivity to unmeasured confounding of childhood predictors in Egypt

Variable	Category	E-value for Estimate	E-value for 95% CI
Relationship with mother	(Ref: Very bad/somewhat bad)		
	Very good/somewhat good	2.31	1.00
Relationship with father	(Ref: Very bad/somewhat bad)		
	Very good/somewhat good	2.08	1.26
Parent marital status	(Ref: Parents married)		
	Divorced	1.67	1.00
	Parents were never married	2.40	1.00
	One or both parents had died	1.77	1.13
Subjective financial status of family growing up	(Ref: Got by)		
	Lived comfortably	1.09	1.00
	Found it difficult	1.37	1.00
	Found it very difficult	1.44	1.00
Abuse	(Ref: No)		
	Yes	1.73	1.22
Outsider growing up	(Ref: No)		
	Yes	1.19	1.00
Self-rated health growing up	(Ref: Good)		
	Excellent	1.23	1.00
	Very good	1.21	1.00
	Fair	1.88	1.00
	Poor	1.29	1.00
Immigration status	(Ref: Born in this country)		
	Born in another country	1.15	1.00
Age 12 religious service attendance	(Ref: Never)		
	At least 1/week	1.52	1.00
	1-3/month	1.64	1.00
	< 1/month	1.17	1.00
Year of birth	(Ref: 1998-2005; age: 18-24)		
	1988-1998; age 25-34	1.87	1.36
	1978-1988; age 35-44	1.54	1.00
	1968-1978; age 45-54	1.42	1.00
	1958-1968; age 55-64	1.55	1.00
	1948-1957; age 65-74	1.99	1.00
	1938-1948; age 75-84	6.95	1.00
	1938 or earlier; 85 or older	394234.32	52972.07
Gender	(Ref: Male)		
	Female	156.55	81.12
Religious affiliation	(Ref: Islam)		
	Collapsed affiliations with prevalence<3%	2.51	1.00
Race/ethnicity	(Ref: Plurality group)		
	Non-plurality groups	1.34	1.00

17. **Table S17. Nationally representative descriptive statistics for Germany: Childhood predictors**

**Characteristic**	**N = 9,506**[1]
**Relationship with mother**	
Very good	5,497 (58%)
Somewhat good	3,031 (32%)
Somewhat bad	496 (5.2%)
Very bad	187 (2.0%)
Does not apply	241 (2.5%)
(Missing)	54 (0.6%)
**Relationship with father**	
Very good	4,652 (49%)
Somewhat good	3,012 (32%)
Somewhat bad	846 (8.9%)
Very bad	385 (4.0%)
Does not apply	538 (5.7%)
(Missing)	73 (0.8%)
**Parent marital status**	
Parents married	7,620 (80%)
Divorced	927 (9.8%)
Parents were never married	578 (6.1%)
One or both parents had died	245 (2.6%)
(Missing)	136 (1.4%)
**Subjective financial status of family growing up**	
Lived comfortably	3,177 (33%)
Got by	4,508 (47%)
Found it difficult	1,481 (16%)
Found it very difficult	314 (3.3%)
(Missing)	26 (0.3%)
**Abuse**	
Yes	1,086 (11%)
No	8,321 (88%)
(Missing)	99 (1.0%)
**Outsider growing up**	
Yes	1,105 (12%)
No	8,262 (87%)
(Missing)	139 (1.5%)
**Self-rated health growing up**	
Excellent	2,633 (28%)
Very good	3,518 (37%)
Good	2,582 (27%)
Fair	612 (6.4%)
Poor	134 (1.4%)
(Missing)	26 (0.3%)
**Immigration status**	
Born in this country	8,722 (92%)
Born in another country	744 (7.8%)
(Missing)	40 (0.4%)
**Age 12 religious service attendance**	
At least 1/week	1,943 (20%)
1-3/month	1,899 (20%)
<1/month	2,887 (30%)
Never	2,749 (29%)
(Missing)	27 0.3%)

18. Table S18. Nationally representative descriptive statistics for Germany: Demographic variables

Characteristic	N = 9,506[1]
**Year of birth**	
1998-2005; age 18-24	829 (8.7%)
1988-1998; age 25-34	1,464 (15%)
1978-1988; age 35-44	1,446 (15%)
1968-1978; age 45-54	1,590 (17%)
1958-1968; age 55-64	1,717 (18%)
1948-1958; age 65-74	1,960 (21%)
1938-1948; age 75-84	453 (4.8%)
1938 or earlier; age 85+	47 (0.5%)
(Missing)	0 (0%)
**Gender**	
Male	4,641 (49%)
Female	4,843 (51%)
Other	11 (0.1%)
(Missing)	11 (0.1%)
**Religious affiliation**	
Christianity	5,751 (61%)
Islam	350 (3.7%)
Hinduism	15 (0.2%)
Buddhism	25 (0.3%)
Judaism	18 (0.2%)
Sikhism	5 (<0.1%)
Baha'i	2 (<0.1%)
Jainism	1 (<0.1%)
Shinto	0 (0%)
Taoism	0 (0%)
Confucianism	4 (<0.1%)
Primal, Animist, or Folk religion	19 (0.2%)
Spiritism	0 (0%)
Umbanda, Candomble, and other African-derived religions	0 (0%)
Chinese folk/traditional religion	0 (0%)
Some other religion	67 (0.7%)
No religion/Atheist/Agnostic	3,163 (33%)
(Missing)	85 (0.9%)

[1]n (%)

19. Table S19. Childhood predictors regression for Germany

Variable	Category	Risk-Ratio	RR 95% CI	Global p-value
Relationship with mother	(Ref: Very bad/somewhat bad)			0.045
	Very good/somewhat good	0.86	(0.74,1.00)	
Relationship with father	(Ref: Very bad/somewhat bad)			0.118
	Very good/somewhat good	0.90	(0.80,1.03)	
Parent marital status	(Ref: Parents married)			0.354
	Divorced	1.06	(0.92,1.22)	
	Parents were never married	1.12	(0.95,1.32)	
	One or both parents had died	0.89	(0.67,1.17)	
Subjective financial status of family growing up	(Ref: Got by)			0.352
	Lived comfortably	1.01	(0.91,1.13)	
	Found it difficult	0.97	(0.85,1.11)	
	Found it very difficult	1.19	(0.95,1.48)	
Abuse	(Ref: No)			1.11e-04
	Yes	1.26	(1.12,1.42)	
Outsider growing up	(Ref: No)			0.130
	Yes	1.10	(0.97,1.24)	
Self-rated health growing up	(Ref: Good)			0.669
	Excellent	1.03	(0.90,1.17)	
	Very good	0.98	(0.88,1.10)	
	Fair	0.99	(0.83,1.18)	
	Poor	0.80	(0.57,1.13)	
Immigration status	(Ref: Born in this country)			0.037
	Born in another country	0.83	(0.69,0.99)	
Age 12 religious service attendance	(Ref: Never)			0.451
	At least 1/week	1.10	(0.96,1.25)	
	1-3/month	1.10	(0.96,1.26)	
	< 1/month	1.05	(0.93,1.19)	
Year of birth	(Ref: 1998-2005; age: 18-24)			8.66e-08
	1988-1998; age 25-34	1.33	(1.07,1.65)	
	1978-1988; age 35-44	1.35	(1.09,1.68)	
	1968-1978; age 45-54	1.65	(1.33,2.03)	
	1958-1968; age 55-64	1.54	(1.25,1.91)	
	1948-1957; age 65-74	1.21	(0.97,1.51)	
	1938-1948; age 75-84	0.98	(0.71,1.35)	
	1938 or earlier; 85 or older	0.46	(0.11,1.86)	
Gender	(Ref: Male)			0.189
	Female	0.92	(0.84,1.01)	
	Other	0.92	(0.23,3.65)	
Religious affiliation	(Ref: No religion/Atheist/Agnostic)			0.008
	Islam	1.34	(1.08,1.67)	
	Christianity	0.94	(0.85,1.05)	
	Collapsed affiliations with prevalence<3%	1.12	(0.79,1.57)	
Race/ethnicity	(Ref: Plurality group)			

20. Table S20. Sensitivity to unmeasured confounding of childhood predictors in Germany

Variable	Category	E-value for Estimate	E-value for 95% CI
Relationship with mother	(Ref: Very bad/somewhat bad)		
	Very good/somewhat good	1.60	1.03
Relationship with father	(Ref: Very bad/somewhat bad)		
	Very good/somewhat good	1.45	1.00
Parent marital status	(Ref: Parents married)		
	Divorced	1.32	1.00
	Parents were never married	1.48	1.00
	One or both parents had died	1.50	1.00
Subjective financial status of family growing up	(Ref: Got by)		
	Lived comfortably	1.14	1.00
	Found it difficult	1.21	1.00
	Found it very difficult	1.66	1.00
Abuse	(Ref: No)		
	Yes	1.84	1.49
Outsider growing up	(Ref: No)		
	Yes	1.43	1.00
Self-rated health growing up	(Ref: Good)		
	Excellent	1.20	1.00
	Very good	1.16	1.00
	Fair	1.12	1.00
	Poor	1.80	1.00
Immigration status	(Ref: Born in this country)		
	Born in another country	1.72	1.12
Age 12 religious service attendance	(Ref: Never)		
	At least 1/week	1.42	1.00
	1-3/month	1.43	1.00
	< 1/month	1.29	1.00
Year of birth	(Ref: 1998-2005; age: 18-24)		
	1988-1998; age 25-34	1.99	1.35
	1978-1988; age 35-44	2.05	1.41
	1968-1978; age 45-54	2.68	2.00
	1958-1968; age 55-64	2.46	1.81
	1948-1957; age 65-74	1.72	1.00
	1938-1948; age 75-84	1.16	1.00
	1938 or earlier; 85 or older	3.77	1.00
Gender	(Ref: Male)		
	Female	1.39	1.00
	Other	1.39	1.00
Religious affiliation	(Ref: No religion/Atheist/Agnostic)		
	Islam	2.02	1.38
	Christianity	1.31	1.00
	Collapsed affiliations with prevalence<3%	1.47	1.00
Race/ethnicity	(Ref: Plurality group)		

21. **Table S21. Nationally representative descriptive statistics for Hong Kong: Childhood predictors**

**Characteristic**	**N = 3,012**[1]
**Relationship with mother**	
Very good	1,077 (36%)
Somewhat good	1,164 (39%)
Somewhat bad	293 (9.7%)
Very bad	49 (1.6%)
Does not apply	426 (14%)
(Missing)	3 (<0.1%)
**Relationship with father**	
Very good	868 (29%)
Somewhat good	1,089 (36%)
Somewhat bad	393 (13%)
Very bad	102 (3.4%)
Does not apply	557 (19%)
(Missing)	3 (0.1%)
**Parent marital status**	
Parents married	2,752 (91%)
Divorced	114 (3.8%)
Parents were never married	40 (1.3%)
One or both parents had died	50 (1.7%)
(Missing)	56 (1.8%)
**Subjective financial status of family growing up**	
Lived comfortably	906 (30%)
Got by	1,527 (51%)
Found it difficult	473 (16%)
Found it very difficult	84 (2.8%)
(Missing)	22 (0.7%)
**Abuse**	
Yes	318 (11%)
No	2,688 (89%)
(Missing)	5 (0.2%)
**Outsider growing up**	
Yes	664 (22%)
No	2,224 (74%)
(Missing)	124 (4.1%)
**Self-rated health growing up**	
Excellent	545 (18%)
Very good	1,073 (36%)
Good	863 (29%)
Fair	426 (14%)
Poor	91 (3.0%)
(Missing)	13 (0.4%)
**Immigration status**	
Born in this country	2,637 (88%)
Born in another country	321 (11%)
(Missing)	53 (1.8%)
**Age 12 religious service attendance**	
At least 1/week	432 (14%)
1-3/month	528 (18%)
<1/month	753 (25%)
Never	1,295 (43%)
(Missing)	4 (0.1%)

22. **Table S22. Nationally representative descriptive statistics for Hong Kong: Demographic variables**

Characteristic	N = 3,012[1]
**Year of birth**	
1998-2005; age 18-24	217 (7.2%)
1988-1998; age 25-34	464 (15%)
1978-1988; age 35-44	542 (18%)
1968-1978; age 45-54	611 (20%)
1958-1968; age 55-64	644 (21%)
1948-1958; age 65-74	492 (16%)
1938-1948; age 75-84	28 (0.9%)
1938 or earlier; age 85+	15 (0.5%)
(Missing)	0 (0%)
**Gender**	
Male	1,390 (46%)
Female	1,620 (54%)
Other	2 (<0.1%)
(Missing)	0 (0%)
**Religious affiliation**	
Christianity	715 (24%)
Islam	86 (2.9%)
Hinduism	27 (0.9%)
Buddhism	323 (11%)
Judaism	16 (0.5%)
Sikhism	4 (0.1%)
Baha'i	0 (0%)
Jainism	1 (<0.1%)
Shinto	18 (0.6%)
Taoism	81 (2.7%)
Confucianism	10 (0.3%)
Primal, Animist, or Folk religion	15 (0.5%)
Spiritism	0 (0%)
Umbanda, Candomble, and other African-derived religions	0 (0%)
Chinese folk/traditional religion	108 (3.6%)
Some other religion	5 (0.2%)
No religion/Atheist/Agnostic	1,601 (53%)
(Missing)	1 (<0.1%)
**Race/Ethnicity**	
Chinese (Cantonese)	1,930 (64%)
Chinese (Chaoshan)	201 (6.7%)
Chinese (Fujianese)	117 (3.9%)
Chinese (Hakka)	121 (4.0%)
Chinese (Other ethnicity)	264 (8.8%)
Chinese (Shanghainese)	89 (2.9%)
East Asian (Korean, Japanese)	10 (0.3%)
Other	4 (0.1%)
South Asian (Indian, Nepalese, Pakistani)	17 (0.6%)
Southeast Asian (Filipino, Indonesian, Thailand)	46 (1.5%)
Taiwanese	14 (0.4%)
White	15 (0.5%)
(Missing)	184 (6.1%)

[1]n (%)

23. Table S23. Childhood predictors regression for Hong Kong

Variable	Category	Risk-Ratio	RR 95% CI	Global p-value
Relationship with mother	(Ref: Very bad/somewhat bad)			0.140
	Very good/somewhat good	0.84	(0.66,1.06)	
Relationship with father	(Ref: Very bad/somewhat bad)			0.653
	Very good/somewhat good	0.95	(0.77,1.18)	
Parent marital status	(Ref: Parents married)			0.854
	Divorced	1.17	(0.80,1.70)	
	Parents were never married	0.95	(0.47,1.92)	
	One or both parents had died	1.00	(0.47,2.14)	
Subjective financial status of family growing up	(Ref: Got by)			0.100
	Lived comfortably	1.12	(0.98,1.29)	
	Found it difficult	0.80	(0.61,1.05)	
	Found it very difficult	0.97	(0.48,1.96)	
Abuse	(Ref: No)			1.48e-04
	Yes	1.33	(1.14,1.56)	
Outsider growing up	(Ref: No)			2.96e-07
	Yes	1.41	(1.23,1.62)	
Self-rated health growing up	(Ref: Good)			3.90e-08
	Excellent	1.50	(1.23,1.83)	
	Very good	1.21	(1.01,1.46)	
	Fair	0.65	(0.48,0.88)	
	Poor	0.42	(0.15,1.12)	
Immigration status	(Ref: Born in this country)			6.34e-.04
	Born in another country	0.50	(0.34,0.76)	
Age 12 religious service attendance	(Ref: Never)			5.17e-13
	At least 1/week	1.28	(0.99,1.65)	
	1-3/month	2.00	(1.63,2.46)	
	< 1/month	1.35	(1.11,1.64)	
Year of birth	(Ref: 1998-2005; age: 18-24)			0.000
	1988-1998; age 25-34	0.96	(0.79,1.17)	
	1978-1988; age 35-44	0.92	(0.75,1.12)	
	1968-1978; age 45-54	1.14	(0.96,1.35)	
	1958-1968; age 55-64	0.91	(0.76,1.09)	
	1948-1957; age 65-74	0.32	(0.17,0.58)	
	1938-1948; age 75-84	0.00	(0.00,0.00)	
	1938 or earlier; 85 or older	0.00	(0.00,0.00)	
Gender	(Ref: Male)			0.000
	Female	0.72	(0.64,0.81)	
	Other	0.00	(0.00,0.00)	
Religious affiliation	(Ref: No religion/Atheist/Agnostic)			0.188
	Buddhism	1.27	(1.04,1.56)	
	Chinese folk/traditional religion	1.21	(0.95,1.54)	
	Christianity	1.19	(0.97,1.45)	
	Collapsed affiliations with prevalence<3%	1.19	(0.93,1.52)	
Race/ethnicity	(Ref: Plurality group)			0.353
	Non-plurality groups	1.06	(0.93,1.20)	

24. Table S24. Sensitivity to unmeasured confounding of childhood predictors in Hong Kong

Variable	Category	E-value for Estimate	E-value for 95% CI
Relationship with mother	(Ref: Very bad/somewhat bad)		
	Very good/somewhat good	1.68	1.00
Relationship with father	(Ref: Very bad/somewhat bad)		
	Very good/somewhat good	1.28	1.00
Parent marital status	(Ref: Parents married)		
	Divorced	1.61	1.00
	Parents were never married	1.28	1.00
	One or both parents had died	1.06	1.00
Subjective financial status of family growing up	(Ref: Got by)		
	Lived comfortably	1.50	1.00
	Found it difficult	1.82	1.00
	Found it very difficult	1.21	1.00
Abuse	(Ref: No)		
	Yes	2.00	1.54
Outsider growing up	(Ref: No)		
	Yes	2.17	1.77
Self-rated health growing up	(Ref: Good)		
	Excellent	2.36	1.75
	Very good	1.72	1.11
	Fair	2.44	1.54
	Poor	4.25	1.00
Immigration status	(Ref: Born in this country)		
	Born in another country	3.38	1.97
Age 12 religious service attendance	(Ref: Never)		
	At least 1/week	1.87	1.00
	1-3/month	3.42	2.65
	< 1/month	2.04	1.46
Year of birth	(Ref: 1998-2005; age: 18-24)		
	1988-1998; age 25-34	1.24	1.00
	1978-1988; age 35-44	1.41	1.00
	1968-1978; age 45-54	1.53	1.00
	1958-1968; age 55-64	1.43	1.00
	1948-1957; age 65-74	5.76	2.85
	1938-1948; age 75-84	4683923.81	1793310.13
	1938 or earlier; 85 or older	6774551.83	2035413.86
Gender	(Ref: Male)		
	Female	2.14	1.79
	Other	7508760.19	1788284.57
Religious affiliation	(Ref: No religion/Atheist/Agnostic)		
	Buddhism	1.86	1.24
	Chinese folk/traditional religion	1.72	1.00
	Christianity	1.66	1.00
	Collapsed affiliations with prevalence<3%	1.67	1.00
Race/ethnicity	(Ref: Plurality group)		
	Non-plurality groups	1.31	1.00

25. Table S25. Nationally representative descriptive statistics for India: Childhood predictors

**Characteristic**	**N = 12,765**[1]
**Relationship with mother**	
Very good	11,465 (90%)
Somewhat good	788 (6.2%)
Somewhat bad	88 (0.7%)
Very bad	73 (0.6%)
Does not apply	269 (2.1%)
(Missing)	82 (0.6%)
**Relationship with father**	
Very good	10,923 (86%)
Somewhat good	995 (7.8%)
Somewhat bad	126 (1.0%)
Very bad	100 (0.8%)
Does not apply	481 (3.8%)
(Missing)	141 (1.1%)
**Parent marital status**	
Parents married	5,578 (44%)
Divorced	236 (1.8%)
Parents were never married	1,055 (8.3%)
One or both parents had died	940 (7.4%)
(Missing)	4,956 (39%)
**Subjective financial status of family growing up**	
Lived comfortably	4,946 (39%)
Got by	3,010 (24%)
Found it difficult	2,703 (21%)
Found it very difficult	2,035 (16%)
(Missing)	70 (0.5%)
**Abuse**	
Yes	1,468 (11%)
No	10,526 (82%)
(Missing)	771 (6.0%)
**Outsider growing up**	
Yes	1,926 (15%)
No	10,780 (84%)
(Missing)	59 (0.5%)
**Self-rated health growing up**	
Excellent	2,182 (17%)
Very good	3,882 (30%)
Good	4,028 (32%)
Fair	2,202 (17%)
Poor	424 (3.3%)
(Missing)	47 (0.4%)
**Immigration status**	
Born in this country	12,629 (99%)
Born in another country	110 (0.9%)
(Missing)	26 (0.2%)
**Age 12 religious service attendance**	
At least 1/week	5,288 (41%)
1-3/month	2,959 (23%)
<1/month	2,719 (21%)
Never	1,478 (12%)
(Missing)	321 .5%)

26. **Table S26. Nationally representative descriptive statistics for India: Demographic variables**

**Characteristic**	**N = 12,765**[1]
**Year of birth**	
1998-2005; age 18-24	2,543 (20%)
1988-1998; age 25-34	3,260 (26%)
1978-1988; age 35-44	2,699 (21%)
1968-1978; age 45-54	1,893 (15%)
1958-1968; age 55-64	1,524 (12%)
1948-1958; age 65-74	676 (5.3%)
1938-1948; age 75-84	148 (1.2%)
1938 or earlier; age 85+	23 (0.2%)
(Missing)	0 (0%)
**Gender**	
Male	6,473 (51%)
Female	6,292 (49%)
Other	0 (0%)
(Missing)	0 (0%)
**Religious affiliation**	
Christianity	254 (2.0%)
Islam	1,550 (12%)
Hinduism	10,417 (82%)
Buddhism	180 (1.4%)
Judaism	0 (0%)
Sikhism	126 (1.0%)
Baha'i	0 (0%)
Jainism	9 (<0.1%)
Shinto	4 (<0.1%)
Taoism	0 (0%)
Confucianism	0 (0%)
Primal, Animist, or Folk religion	27 (0.2%)
Spiritism	0 (0%)
Umbanda, Candomble, and other African-derived religions	0 (0%)
Chinese folk/traditional religion	0 (0%)
Some other religion	59 (0.5%)
No religion/Atheist/Agnostic	7 (<0.1%)
(Missing)	131 (1.0%)
**Race/Ethnicity**	
General	3,538 (28%)
Other backward caste	4,177 (33%)
Schedule caste	3,599 (28%)
Schedule tribe	1,185 (9.3%)
(Missing)	267 (2.1%)

[1]n (%)

27. Table S27. Childhood predictors regression for India

Variable	Category	Risk-Ratio	RR 95% CI	Global p-value
Relationship with mother	(Ref: Very bad/somewhat bad)			0.870
	Very good/somewhat good	1.04	(0.65,1.66)	
Relationship with father	(Ref: Very bad/somewhat bad)			0.065
	Very good/somewhat good	0.67	(0.44,1.03)	
Parent marital status	(Ref: Parents married)			0.871
	Divorced	0.99	(0.64,1.54)	
	Parents were never married	0.99	(0.76,1.28)	
	One or both parents had died	0.94	(0.72,1.23)	
Subjective financial status of family growing up	(Ref: Got by)			0.024
	Lived comfortably	1.00	(0.83,1.21)	
	Found it difficult	0.94	(0.75,1.18)	
	Found it very difficult	1.30	(1.02,1.64)	
Abuse	(Ref: No)			1.51e-04
	Yes	1.43	(1.17,1.74)	
Outsider growing up	(Ref: No)			0.301
	Yes	1.10	(0.91,1.33)	
Self-rated health growing up	(Ref: Good)			0.196
	Excellent	1.06	(0.85,1.30)	
	Very good	1.09	(0.90,1.33)	
	Fair	1.15	(0.93,1.41)	
	Poor	0.65	(0.39,1.08)	
Immigration status	(Ref: Born in this country)			9.67e-.06
	Born in another country	2.37	(1.62,3.46)	
Age 12 religious service attendance	(Ref: Never)			0.157
	At least 1/week	0.97	(0.76,1.23)	
	1-3/month	1.18	(0.92,1.52)	
	< 1/month	1.06	(0.82,1.38)	
Year of birth	(Ref: 1998-2005; age: 18-24)			0.000
	1988-1998; age 25-34	1.21	(0.93,1.56)	
	1978-1988; age 35-44	1.37	(1.05,1.78)	
	1968-1978; age 45-54	1.68	(1.28,2.20)	
	1958-1968; age 55-64	1.57	(1.14,2.16)	
	1948-1957; age 65-74	1.14	(0.74,1.76)	
	1938-1948; age 75-84	1.22	(0.57,2.60)	
	1938 or earlier; 85 or older	0.00	(0.00,6311.20)	
Gender	(Ref: Male)			0.000
	Female	0.07	(0.05,0.10)	
Religious affiliation	(Ref: Hinduism)			0.005
	Islam	1.31	(1.03,1.66)	
	Collapsed affiliations with prevalence<3%	0.64	(0.44,0.95)	
Race/ethnicity	(Ref: Plurality group)			0.852
	Non-plurality groups	1.01	(0.86,1.18)	

28. Table S28. Sensitivity to unmeasured confounding of childhood predictors in India

Variable	Category	E-value for Estimate	E-value for 95% CI
Relationship with mother	(Ref: Very bad/somewhat bad)		
	Very good/somewhat good	1.23	1.00
Relationship with father	(Ref: Very bad/somewhat bad)		
	Very good/somewhat good	2.34	1.00
Parent marital status	(Ref: Parents married)		
	Divorced	1.09	1.00
	Parents were never married	1.13	1.00
	One or both parents had died	1.32	1.00
Subjective financial status of family growing up	(Ref: Got by)		
	Lived comfortably	1.05	1.00
	Found it difficult	1.32	1.00
	Found it very difficult	1.92	1.18
Abuse	(Ref: No)		
	Yes	2.21	1.62
Outsider growing up	(Ref: No)		
	Yes	1.44	1.00
Self-rated health growing up	(Ref: Good)		
	Excellent	1.30	1.00
	Very good	1.41	1.00
	Fair	1.55	1.00
	Poor	2.43	1.00
Immigration status	(Ref: Born in this country)		
	Born in another country	4.17	2.63
Age 12 religious service attendance	(Ref: Never)		
	At least 1/week	1.23	1.00
	1-3/month	1.65	1.00
	< 1/month	1.32	1.00
Year of birth	(Ref: 1998-2005; age: 18-24)		
	1988-1998; age 25-34	1.71	1.00
	1978-1988; age 35-44	2.07	1.28
	1968-1978; age 45-54	2.75	1.88
	1958-1968; age 55-64	2.52	1.55
	1948-1957; age 65-74	1.53	1.00
	1938-1948; age 75-84	1.74	1.00
	1938 or earlier; 85 or older	14538.94	1.00
Gender	(Ref: Male)		
	Female	26.94	18.60
Religious affiliation	(Ref: Hinduism)		
	Islam	1.94	1.19
	Collapsed affiliations with prevalence<3%	2.48	1.29
Race/ethnicity	(Ref: Plurality group)		
	Non-plurality groups	1.10	1.00

29. Table S29. Nationally representative descriptive statistics for Indonesia: Childhood predictors

Characteristic	N = 6,992[1]
**Relationship with mother**	
Very good	6,238 (89%)
Somewhat good	583 (8.3%)
Somewhat bad	50 (0.7%)
Very bad	26 (0.4%)
Does not apply	68 (1.0%)
(Missing)	27 (0.4%)
**Relationship with father**	
Very good	6,067 (87%)
Somewhat good	628 (9.0%)
Somewhat bad	68 (1.0%)
Very bad	52 (0.7%)
Does not apply	115 (1.6%)
(Missing)	61 (0.9%)
**Parent marital status**	
Parents married	5,557 (79%)
Divorced	448 (6.4%)
Parents were never married	47 (0.7%)
One or both parents had died	735 (11%)
(Missing)	205 (2.9%)
**Subjective financial status of family growing up**	
Lived comfortably	3,408 (49%)
Got by	2,955 (42%)
Found it difficult	439 (6.3%)
Found it very difficult	181 (2.6%)
(Missing)	9 (0.1%)
**Abuse**	
Yes	486 (6.9%)
No	6,427 (92%)
(Missing)	79 (1.1%)
**Outsider growing up**	
Yes	343 (4.9%)
No	6,639 (95%)
(Missing)	10 (0.1%)
**Self-rated health growing up**	
Excellent	1,246 (18%)
Very good	1,968 (28%)
Good	2,490 (36%)
Fair	1,233 (18%)
Poor	55 (0.8%)
(Missing)	1 (<0.1%)
**Immigration status**	
Born in this country	6,958 (100%)
Born in another country	34 (0.5%)
(Missing)	0 (0%)
**Age 12 religious service attendance**	
At least 1/week	5,363 (77%)
1-3/month	973 (14%)
<1/month	329 (4.7%)
Never	275 (3.9%)
(Missing)	51 (0.7%)

30. Table S30. Nationally representative descriptive statistics for Indonesia: Demographic variables

Characteristic	N = 6,992[1]
**Year of birth**	
1998-2005; age 18-24	1,216 (17%)
1988-1998; age 25-34	1,707 (24%)
1978-1988; age 35-44	1,613 (23%)
1968-1978; age 45-54	1,301 (19%)
1958-1968; age 55-64	910 (13%)
1948-1958; age 65-74	218 (3.1%)
1938-1948; age 75-84	18 (0.3%)
1938 or earlier; age 85+	9 (0.1%)
(Missing)	0 (0%)
**Gender**	
Male	3,461 (50%)
Female	3,513 (50%)
Other	7 (<0.1%)
(Missing)	11 (0.2%)
**Religious affiliation**	
Christianity	528 (7.6%)
Islam	6,373 (91%)
Hinduism	75 (1.1%)
Buddhism	5 (<0.1%)
Judaism	0 (0%)
Sikhism	0 (0%)
Baha'i	0 (0%)
Jainism	1 (<0.1%)
Shinto	0 (0%)
Taoism	0 (<0.1%)
Confucianism	1 (<0.1%)
Primal, Animist, or Folk religion	1 (<0.1%)
Spiritism	0 (0%)
Umbanda, Candomble, and other African-derived religions	0 (0%)
Chinese folk/traditional religion	0 (0%)
Some other religion	0 (0%)
No religion/Atheist/Agnostic	2 (<0.1%)
(Missing)	8 (0.1%)
**Race/Ethnicity**	
Bali	69 (1.0%)
Banjar/Melayu Banjar	320 (4.6%)
Batak	165 (2.4%)
Betawi	251 (3.6%)
Bugis	243 (3.5%)
Jawa	2,846 (41%)
Madura	262 (3.7%)
Makasar	91 (1.3%)
Minangkabau	273 (3.9%)
Other	1,262 (18%)
Sunda/Parahyangan	1,172 (17%)
(Missing)	38 (0.5%)

[1]n (%)

31. Table S31. Childhood predictors regression for Indonesia

Variable	Category	Risk-Ratio	RR 95% CI	Global p-value
Relationship with mother	(Ref: Very bad/somewhat bad)			0.706
	Very good/somewhat good	0.97	(0.77,1.21)	
Relationship with father	(Ref: Very bad/somewhat bad)			0.835
	Very good/somewhat good	1.00	(0.79,1.26)	
Parent marital status	(Ref: Parents married)			0.093
	Divorced	1.09	(0.97,1.22)	
	Parents were never married	1.09	(0.73,1.64)	
	One or both parents had died	1.11	(1.00,1.24)	
Subjective financial status of family growing up	(Ref: Got by)			0.352
	Lived comfortably	0.96	(0.91,1.02)	
	Found it difficult	0.99	(0.88,1.12)	
	Found it very difficult	1.12	(0.91,1.37)	
Abuse	(Ref: No)			0.084
	Yes	1.10	(0.98,1.24)	
Outsider growing up	(Ref: No)			0.553
	Yes	1.04	(0.90,1.20)	
Self-rated health growing up	(Ref: Good)			0.754
	Excellent	0.98	(0.90,1.07)	
	Very good	0.97	(0.90,1.04)	
	Fair	1.01	(0.93,1.09)	
	Poor	0.88	(0.63,1.24)	
Immigration status	(Ref: Born in this country)			0.587
	Born in another country	0.95	(0.67,1.33)	
Age 12 religious service attendance	(Ref: Never)			0.633
	At least 1/week	0.92	(0.80,1.07)	
	1-3/month	0.95	(0.80,1.12)	
	< 1/month	0.97	(0.80,1.18)	
Year of birth	(Ref: 1998-2005; age: 18-24)			0.000
	1988-1998; age 25-34	1.26	(1.13,1.40)	
	1978-1988; age 35-44	1.26	(1.14,1.41)	
	1968-1978; age 45-54	1.26	(1.12,1.42)	
	1958-1968; age 55-64	1.18	(1.01,1.37)	
	1948-1957; age 65-74	1.02	(0.76,1.37)	
	1938-1948; age 75-84	0.00	(0.00,0.00)	
	1938 or earlier; 85 or older	1.52	(1.13,2.03)	
Gender	(Ref: Male)			0.000
	Female	0.07	(0.06,0.09)	
	Other	0.57	(0.04,8.10)	
Religious affiliation	(Ref: Islam)			0.021
	Christianity	0.89	(0.78,1.02)	
	Collapsed affiliations with prevalence<3%	0.40	(0.18,0.88)	
Race/ethnicity	(Ref: Plurality group)			2.31e-04
	Non-plurality groups	1.14	(1.07,1.21)	

32. **Table S32. Sensitivity to unmeasured confounding of childhood predictors in Indonesia**

Variable	Category	E-value for Estimate	E-value for 95% CI
Relationship with mother	(Ref: Very bad/somewhat bad)		
	Very good/somewhat good	1.22	1.00
Relationship with father	(Ref: Very bad/somewhat bad)		
	Very good/somewhat good	1.07	1.00
Parent marital status	(Ref: Parents married)		
	Divorced	1.39	1.00
	Parents were never married	1.41	1.00
	One or both parents had died	1.47	1.04
Subjective financial status of family growing up	(Ref: Got by)		
	Lived comfortably	1.24	1.00
	Found it difficult	1.09	1.00
	Found it very difficult	1.48	1.00
Abuse	(Ref: No)		
	Yes	1.44	1.00
Outsider growing up	(Ref: No)		
	Yes	1.24	1.00
Self-rated health growing up	(Ref: Good)		
	Excellent	1.17	1.00
	Very good	1.21	1.00
	Fair	1.09	1.00
	Poor	1.52	1.00
Immigration status	(Ref: Born in this country)		
	Born in another country	1.30	1.00
Age 12 religious service attendance	(Ref: Never)		
	At least 1/week	1.38	1.00
	1-3/month	1.30	1.00
	< 1/month	1.21	1.00
Year of birth	(Ref: 1998-2005; age: 18-24)		
	1988-1998; age 25-34	1.83	1.52
	1978-1988; age 35-44	1.84	1.53
	1968-1978; age 45-54	1.84	1.50
	1958-1968; age 55-64	1.63	1.12
	1948-1957; age 65-74	1.18	1.00
	1938-1948; age 75-84	2728155.60	1092150.56
	1938 or earlier; 85 or older	2.40	1.53
Gender	(Ref: Male)		
	Female	27.31	22.18
	Other	2.90	1.00
Religious affiliation	(Ref: Islam)		
	Christianity	1.49	1.00
	Collapsed affiliations with prevalence<3%	4.40	1.51
Race/ethnicity	(Ref: Plurality group)		
	Non-plurality groups	1.53	1.34

33. Table S33. Nationally representative descriptive statistics for Israel: Childhood predictors

Characteristic	N = 3,669[1]
**Relationship with mother**	
Very good	2,686 (73%)
Somewhat good	793 (22%)
Somewhat bad	110 (3.0%)
Very bad	18 (0.5%)
Does not apply	45 (1.2%)
(Missing)	17 (0.5%)
**Relationship with father**	
Very good	2,290 (62%)
Somewhat good	912 (25%)
Somewhat bad	234 (6.4%)
Very bad	37 (1.0%)
Does not apply	171 (4.7%)
(Missing)	25 (0.7%)
**Parent marital status**	
Parents married	3,172 (86%)
Divorced	284 (7.8%)
Parents were never married	36 (1.0%)
One or both parents had died	130 (3.5%)
(Missing)	47 (1.3%)
**Subjective financial status of family growing up**	
Lived comfortably	923 (25%)
Got by	1,822 (50%)
Found it difficult	667 (18%)
Found it very difficult	239 (6.5%)
(Missing)	17 (0.5%)
**Abuse**	
Yes	0 (0%)
No	0 (0%)
(Missing)	3,669 (100%)
**Outsider growing up**	
Yes	371 (10%)
No	3,228 (88%)
(Missing)	70 (1.9%)
**Self-rated health growing up**	
Excellent	1,785 (49%)
Very good	1,284 (35%)
Good	480 (13%)
Fair	105 (2.9%)
Poor	6 (0.2%)
(Missing)	8 (0.2%)
**Immigration status**	
Born in this country	2,796 (76%)
Born in another country	868 (24%)
(Missing)	5 (0.1%)
**Age 12 religious service attendance**	
At least 1/week	867 (24%)
1-3/month	435 (12%)
<1/month	810 (22%)
Never	1,539 (42%)
(Missing)	17 (0.5%)

34. Table S34. Nationally representative descriptive statistics for Israel: Demographic variables

Characteristic	N = 3,669[1]
**Year of birth**	
1998-2005; age 18-24	553 (15%)
1988-1998; age 25-34	712 (19%)
1978-1988; age 35-44	666 (18%)
1968-1978; age 45-54	592 (16%)
1958-1968; age 55-64	504 (14%)
1948-1958; age 65-74	427 (12%)
1938-1948; age 75-84	202 (5.5%)
1938 or earlier; age 85+	12 (0.3%)
(Missing)	0 (0%)
**Gender**	
Male	1,791 (49%)
Female	1,872 (51%)
Other	0 (<0.1%)
(Missing)	6 (0.2%)
**Religious affiliation**	
Christianity	60 (1.6%)
Islam	647 (18%)
Hinduism	0 (0%)
Buddhism	0 (0%)
Judaism	2,873 (78%)
Sikhism	1 (<0.1%)
Baha'i	1 (<0.1%)
Jainism	0 (0%)
Shinto	0 (0%)
Taoism	0 (0%)
Confucianism	0 (0%)
Primal, Animist, or Folk religion	3 (<0.1%)
Spiritism	0 (0%)
Umbanda, Candomble, and other African-derived religions	0 (0%)
Chinese folk/traditional religion	0 (0%)
Some other religion	5 (0.1%)
No religion/Atheist/Agnostic	69 (1.9%)
(Missing)	10 (0.3%)
**Race/Ethnicity**	
Arab	674 (18%)
Jewish	2,926 (80%)
Other	39 (1.1%)
(Missing)	30 (0.8%)

[1]n (%)

35. Table S35. Childhood predictors regression for Israel

Variable	Category	Risk-Ratio	RR 95% CI	Global p-value
Relationship with mother	(Ref: Very bad/somewhat bad)			0.332
	Very good/somewhat good	1.19	(0.84,1.68)	
Relationship with father	(Ref: Very bad/somewhat bad)			0.016
	Very good/somewhat good	0.75	(0.59,0.95)	
Parent marital status	(Ref: Parents married)			0.068
	Divorced	1.35	(1.08,1.69)	
	Parents were never married	0.98	(0.53,1.81)	
	One or both parents had died	0.89	(0.61,1.30)	
Subjective financial status of family growing up	(Ref: Got by)			0.019
	Lived comfortably	0.92	(0.76,1.10)	
	Found it difficult	1.27	(1.07,1.51)	
	Found it very difficult	1.13	(0.88,1.46)	
Abuse	(Ref: No)			
Outsider growing up	(Ref: No)			0.695
	Yes	0.96	(0.77,1.19)	
Self-rated health growing up	(Ref: Good)			0.000
	Excellent	0.96	(0.78,1.19)	
	Very good	0.90	(0.73,1.09)	
	Fair	1.01	(0.65,1.58)	
	Poor	0.00	(0.00,0.00)	
Immigration status	(Ref: Born in this country)			0.728
	Born in another country	1.03	(0.86,1.23)	
Age 12 religious service attendance	(Ref: Never)			0.005
	At least 1/week	0.93	(0.77,1.11)	
	1-3/month	0.69	(0.51,0.92)	
	< 1/month	0.74	(0.62,0.89)	
Year of birth	(Ref: 1998-2005; age: 18-24)			1.14e-06
	1988-1998; age 25-34	1.45	(1.11,1.91)	
	1978-1988; age 35-44	1.68	(1.29,2.20)	
	1968-1978; age 45-54	1.42	(1.07,1.90)	
	1958-1968; age 55-64	1.51	(1.12,2.02)	
	1948-1957; age 65-74	0.86	(0.61,1.22)	
	1938-1948; age 75-84	0.47	(0.26,0.85)	
	1938 or earlier; 85 or older	1.07	(0.36,3.20)	
Gender	(Ref: Male)			0.000
	Female	0.39	(0.34,0.45)	
	Other	3.15	(2.03,4.89)	
Religious affiliation	(Ref: Judaism)			0.016
	Islam	0.60	(0.31,1.19)	
	Collapsed affiliations with prevalence<3%	1.08	(0.74,1.57)	
Race/ethnicity	(Ref: Plurality group)			0.056
	Non-plurality groups	1.67	(0.84,3.30)	

36. **Table S36. Sensitivity to unmeasured confounding of childhood predictors in Israel**

Variable	Category	E-value for Estimate	E-value for 95% CI
Relationship with mother	(Ref: Very bad/somewhat bad)		
	Very good/somewhat good	1.66	1.00
Relationship with father	(Ref: Very bad/somewhat bad)		
	Very good/somewhat good	2.00	1.28
Parent marital status	(Ref: Parents married)		
	Divorced	2.04	1.36
	Parents were never married	1.14	1.00
	One or both parents had died	1.50	1.00
Subjective financial status of family growing up	(Ref: Got by)		
	Lived comfortably	1.40	1.00
	Found it difficult	1.86	1.33
	Found it very difficult	1.51	1.00
Abuse	(Ref: No)		
Outsider growing up	(Ref: No)		
	Yes	1.24	1.00
Self-rated health growing up	(Ref: Good)		
	Excellent	1.24	1.00
	Very good	1.48	1.00
	Fair	1.13	1.00
	Poor	688067.06	149744.81
Immigration status	(Ref: Born in this country)		
	Born in another country	1.21	1.00
Age 12 religious service attendance	(Ref: Never)		
	At least 1/week	1.38	1.00
	1-3/month	2.27	1.39
	< 1/month	2.02	1.49
Year of birth	(Ref: 1998-2005; age: 18-24)		
	1988-1998; age 25-34	2.27	1.45
	1978-1988; age 35-44	2.75	1.89
	1968-1978; age 45-54	2.20	1.35
	1958-1968; age 55-64	2.38	1.48
	1948-1957; age 65-74	1.60	1.00
	1938-1948; age 75-84	3.68	1.65
	1938 or earlier; 85 or older	1.34	1.00
Gender	(Ref: Male)		
	Female	4.59	3.90
	Other	5.75	3.47
Religious affiliation	(Ref: Judaism)		
	Islam	2.70	1.00
	Collapsed affiliations with prevalence<3%	1.37	1.00
Race/ethnicity	(Ref: Plurality group)		
	Non-plurality groups	2.72	1.00

37. **Table S37. Nationally representative descriptive statistics for Japan: Childhood predictors**

Characteristic	N = 20,543[1]
**Relationship with mother**	
Very good	5,630 (27%)
Somewhat good	9,461 (46%)
Somewhat bad	2,750 (13%)
Very bad	799 (3.9%)
Does not apply	1,838 (8.9%)
(Missing)	66 (0.3%)
**Relationship with father**	
Very good	4,156 (20%)
Somewhat good	9,081 (44%)
Somewhat bad	3,446 (17%)
Very bad	1,223 (6.0%)
Does not apply	2,580 (13%)
(Missing)	57 (0.3%)
**Parent marital status**	
Parents married	17,713 (86%)
Divorced	1,127 (5.5%)
Parents were never married	591 (2.9%)
One or both parents had died	754 (3.7%)
(Missing)	359 (1.7%)
**Subjective financial status of family growing up**	
Lived comfortably	8,320 (41%)
Got by	8,799 (43%)
Found it difficult	2,398 (12%)
Found it very difficult	973 (4.7%)
(Missing)	52 (0.3%)
**Abuse**	
Yes	1,482 (7.2%)
No	18,964 (92%)
(Missing)	96 (0.5%)
**Outsider growing up**	
Yes	1,963 (9.6%)
No	17,136 (83%)
(Missing)	1,444 (7.0%)
**Self-rated health growing up**	
Excellent	2,711 (13%)
Very good	7,106 (35%)
Good	6,689 (33%)
Fair	3,199 (16%)
Poor	758 (3.7%)
(Missing)	80 (0.4%)
**Immigration status**	
Born in this country	19,548 (95%)
Born in another country	158 (0.8%)
(Missing)	837 (4.1%)
**Age 12 religious service attendance**	
At least 1/week	398 (1.9%)
1-3/month	883 (4.3%)
<1/month	5,023 (24%)
Never	14,117 (69%)
(Missing)	123 (0.6%)

38. **Table S38. Nationally representative descriptive statistics for Japan: Demographic variables**

Characteristic	N = 20,543[1]
**Year of birth**	
1998-2005; age 18-24	1,589 (7.7%)
1988-1998; age 25-34	2,425 (12%)
1978-1988; age 35-44	3,075 (15%)
1968-1978; age 45-54	3,595 (17%)
1958-1968; age 55-64	2,965 (14%)
1948-1958; age 65-74	5,300 (26%)
1938-1948; age 75-84	1,472 (7.2%)
1938 or earlier; age 85+	120 (0.6%)
(Missing)	0 (0%)
**Gender**	
Male	9,847 (48%)
Female	10,602 (52%)
Other	28 (0.1%)
(Missing)	66 (0.3%)
**Religious affiliation**	
Christianity	343 (1.7%)
Islam	7 (<0.1%)
Hinduism	4 (<0.1%)
Buddhism	6,536 (32%)
Judaism	0 (0%)
Sikhism	0 (0%)
Baha'i	7 (<0.1%)
Jainism	1 (<0.1%)
Shinto	382 (1.9%)
Taoism	14 (<0.1%)
Confucianism	25 (0.1%)
Primal, Animist, or Folk religion	13 (<0.1%)
Spiritism	0 (0%)
Umbanda, Candomble, and other African-derived religions	0 (0%)
Chinese folk/traditional religion	0 (0%)
Some other religion	46 (0.2%)
No religion/Atheist/Agnostic	12,950 (63%)
(Missing)	215 (1.0%)

[1]n (%)

39. Table S39. Childhood predictors regression for Japan

Variable	Category	Risk-Ratio	RR 95% CI	Global p-value
Relationship with mother	(Ref: Very bad/somewhat bad)			0.786
	Very good/somewhat good	1.01	(0.93,1.10)	
Relationship with father	(Ref: Very bad/somewhat bad)			0.356
	Very good/somewhat good	0.96	(0.89,1.04)	
Parent marital status	(Ref: Parents married)			2.13e-07
	Divorced	1.40	(1.24,1.57)	
	Parents were never married	1.15	(0.97,1.37)	
	One or both parents had died	1.13	(0.97,1.32)	
Subjective financial status of family growing up	(Ref: Got by)			0.631
	Lived comfortably	0.98	(0.91,1.05)	
	Found it difficult	0.96	(0.87,1.06)	
	Found it very difficult	0.92	(0.80,1.07)	
Abuse	(Ref: No)			5.29e-05
	Yes	1.25	(1.12,1.39)	
Outsider growing up	(Ref: No)			
	Yes	1.22	(1.10,1.35)	8.45e-05
Self-rated health growing up	(Ref: Good)			0.005
	Excellent	1.05	(0.95,1.16)	
	Very good	1.10	(1.02,1.18)	
	Fair	0.93	(0.84,1.02)	
	Poor	1.08	(0.92,1.28)	
Immigration status	(Ref: Born in this country)			0.187
	Born in another country	1.25	(0.90,1.75)	
Age 12 religious service attendance	(Ref: Never)			0.001
	At least 1/week	1.09	(0.86,1.39)	
	1-3/month	1.28	(1.12,1.47)	
	< 1/month	1.08	(1.01,1.16)	
Year of birth	(Ref: 1998-2005; age: 18-24)			0.000
	1988-1998; age 25-34	1.74	(1.42,2.14)	
	1978-1988; age 35-44	2.50	(2.06,3.03)	
	1968-1978; age 45-54	2.63	(2.17,3.18)	
	1958-1968; age 55-64	2.67	(2.21,3.23)	
	1948-1957; age 65-74	1.75	(1.44,2.12)	
	1938-1948; age 75-84	0.90	(0.70,1.17)	
	1938 or earlier; 85 or older	0.50	(0.15,1.66)	
Gender	(Ref: Male)			0.000
	Female	0.44	(0.41,0.47)	
	Other	0.30	(0.12,0.75)	
Religious affiliation	(Ref: No religion/Atheist/Agnostic)			0.098
	Buddhism	1.06	(0.99,1.13)	
	Collapsed affiliations with prevalence<3%	1.13	(0.97,1.32)	
Race/ethnicity	(Ref: Plurality group)			

40. Table S40. Sensitivity to unmeasured confounding of childhood predictors in Japan

Variable	Category	E-value for Estimate	E-value for 95% CI
Relationship with mother	(Ref: Very bad/somewhat bad)		
	Very good/somewhat good	1.11	1.00
Relationship with father	(Ref: Very bad/somewhat bad)		
	Very good/somewhat good	1.23	1.00
Parent marital status	(Ref: Parents married)		
	Divorced	2.14	1.79
	Parents were never married	1.58	1.00
	One or both parents had died	1.51	1.00
Subjective financial status of family growing up	(Ref: Got by)		
	Lived comfortably	1.17	1.00
	Found it difficult	1.25	1.00
	Found it very difficult	1.39	1.00
Abuse	(Ref: No)		
	Yes	1.80	1.49
Outsider growing up	(Ref: No)		
	Yes	1.73	1.43
Self-rated health growing up	(Ref: Good)		
	Excellent	1.27	1.00
	Very good	1.43	1.17
	Fair	1.37	1.00
	Poor	1.38	1.00
Immigration status	(Ref: Born in this country)		
	Born in another country	1.81	1.00
Age 12 religious service attendance	(Ref: Never)		
	At least 1/week	1.41	1.00
	1-3/month	1.89	1.49
	< 1/month	1.37	1.10
Year of birth	(Ref: 1998-2005; age: 18-24)		
	1988-1998; age 25-34	2.88	2.19
	1978-1988; age 35-44	4.44	3.54
	1968-1978; age 45-54	4.70	3.77
	1958-1968; age 55-64	4.79	3.84
	1948-1957; age 65-74	2.90	2.25
	1938-1948; age 75-84	1.45	1.00
	1938 or earlier; 85 or older	3.41	1.00
Gender	(Ref: Male)		
	Female	3.99	3.69
	Other	6.15	1.99
Religious affiliation	(Ref: No religion/Atheist/Agnostic)		
	Buddhism	1.32	1.00
	Collapsed affiliations with prevalence<3%	1.52	1.00
Race/ethnicity	(Ref: Plurality group)		

41. Table S41. Nationally representative descriptive statistics for Kenya: Childhood predictors

Characteristic	N = 11,389[1]
**Relationship with mother**	
Very good	9,418 (83%)
Somewhat good	1,435 (13%)
Somewhat bad	130 (1.1%)
Very bad	100 (0.9%)
Does not apply	240 (2.1%)
(Missing)	66 (0.6%)
**Relationship with father**	
Very good	7,958 (70%)
Somewhat good	1,896 (17%)
Somewhat bad	216 (1.9%)
Very bad	220 (1.9%)
Does not apply	967 (8.5%)
(Missing)	132 (1.2%)
**Parent marital status**	
Parents married	9,238 (81%)
Divorced	697 (6.1%)
Parents were never married	681 (6.0%)
One or both parents had died	471 (4.1%)
(Missing)	301 (2.6%)
**Subjective financial status of family growing up**	
Lived comfortably	3,026 (27%)
Got by	3,279 (29%)
Found it difficult	4,071 (36%)
Found it very difficult	994 (8.7%)
(Missing)	19 (0.2%)
**Abuse**	
Yes	1,300 (11%)
No	10,039 (88%)
(Missing)	49 (0.4%)
**Outsider growing up**	
Yes	1,223 (11%)
No	10,114 (89%)
(Missing)	52 (0.5%)
**Self-rated health growing up**	
Excellent	4,449 (39%)
Very good	2,598 (23%)
Good	2,582 (23%)
Fair	1,384 (12%)
Poor	349 (3.1%)
(Missing)	26 (0.2%)
**Immigration status**	
Born in this country	11,270 (99%)
Born in another country	117 (1.0%)
(Missing)	2 (<0.1%)
**Age 12 religious service attendance**	
At least 1/week	9,189 (81%)
1-3/month	1,687 (15%)
<1/month	236 (2.1%)
Never	198 (1.7%)
(Missing)	79 (0.7%)

42. **Table S42. Nationally representative descriptive statistics for Kenya: Demographic variables**

Characteristic	N = 11,389[1]
**Year of birth**	
1998-2005; age 18-24	2,868 (25%)
1988-1998; age 25-34	3,335 (29%)
1978-1988; age 35-44	2,182 (19%)
1968-1978; age 45-54	1,378 (12%)
1958-1968; age 55-64	872 (7.7%)
1948-1958; age 65-74	600 (5.3%)
1938-1948; age 75-84	122 (1.1%)
1938 or earlier; age 85+	27 (0.2%)
(Missing)	5 (<0.1%)
**Gender**	
Male	5,567 (49%)
Female	5,813 (51%)
Other	2 (<0.1%)
(Missing)	7 (<0.1%)
**Religious affiliation**	
Christianity	10,369 (91%)
Islam	916 (8.0%)
Hinduism	0 (0%)
Buddhism	5 (<0.1%)
Judaism	6 (<0.1%)
Sikhism	0 (<0.1%)
Baha'i	3 (<0.1%)
Jainism	1 (<0.1%)
Shinto	0 (0%)
Taoism	0 (0%)
Confucianism	0 (0%)
Primal, Animist, or Folk religion	13 (0.1%)
Spiritism	0 (0%)
Umbanda, Candomble, and other African-derived religions	0 (0%)
Chinese folk/traditional religion	0 (0%)
Some other religion	0 (<0.1%)
No religion/Atheist/Agnostic	67 (0.6%)
(Missing)	9 (<0.1%)
**Race/Ethnicity**	
Embu	197 (1.7%)
Kalenjin	1,377 (12%)
Kamba	1,299 (11%)
Kenyan Somali/Somali	396 (3.5%)
Kikuyu	2,119 (19%)
Kisii	789 (6.9%)
Luhya	1,943 (17%)
Luo	1,120 (9.8%)
Maasai	237 (2.1%)
Meru	630 (5.5%)
Miji Kenda tribes	708 (6.2%)
Other	548 (4.8%)
(Missing)	27 (0.2%)

[1]n (%)

43. Table S43. Childhood predictors regression for Kenya

Variable	Category	Risk-Ratio	RR 95% CI	Global p-value
Relationship with mother	(Ref: Very bad/somewhat bad)			0.740
	Very good/somewhat good	1.12	(0.56,2.25)	
Relationship with father	(Ref: Very bad/somewhat bad)			0.683
	Very good/somewhat good	0.93	(0.56,1.53)	
Parent marital status	(Ref: Parents married)			0.301
	Divorced	1.20	(0.82,1.76)	
	Parents were never married	0.78	(0.55,1.12)	
	One or both parents had died	1.22	(0.78,1.91)	
Subjective financial status of family growing up	(Ref: Got by)			0.208
	Lived comfortably	0.75	(0.55,1.03)	
	Found it difficult	1.02	(0.79,1.31)	
	Found it very difficult	0.92	(0.59,1.44)	
Abuse	(Ref: No)			1.91e-05
	Yes	1.84	(1.40,2.42)	
Outsider growing up	(Ref: No)			0.011
	Yes	1.45	(1.09,1.91)	
Self-rated health growing up	(Ref: Good)			0.204
	Excellent	0.93	(0.71,1.21)	
	Very good	0.95	(0.70,1.28)	
	Fair	0.80	(0.54,1.17)	
	Poor	0.49	(0.25,0.97)	
Immigration status	(Ref: Born in this country)			0.815
	Born in another country	0.90	(0.30,2.72)	
Age 12 religious service attendance	(Ref: Never)			0.742
	At least 1/week	0.75	(0.43,1.31)	
	1-3/month	0.72	(0.39,1.33)	
	< 1/month	0.70	(0.35,1.41)	
Year of birth	(Ref: 1998-2005; age: 18-24)			0.000
	1988-1998; age 25-34	1.64	(1.25,2.17)	
	1978-1988; age 35-44	2.11	(1.54,2.90)	
	1968-1978; age 45-54	2.69	(1.91,3.80)	
	1958-1968; age 55-64	2.82	(1.95,4.07)	
	1948-1957; age 65-74	3.04	(1.90,4.89)	
	1938-1948; age 75-84	0.89	(0.26,3.02)	
	1938 or earlier; 85 or older	0.00	(0.00,0.00)	
Gender	(Ref: Male)			0.000
	Female	0.09	(0.06,0.13)	
	Other	0.00	(0.00,0.00)	
Religious affiliation	(Ref: Christianity)			0.004
	Islam	1.59	(1.22,2.09)	
	Collapsed affiliations with prevalence<3%	2.05	(1.20,3.49)	
Race/ethnicity	(Ref: Plurality group)			5.93e-04
	Non-plurality groups	0.55	(0.42,0.72)	

44. Table S44. Sensitivity to unmeasured confounding of childhood predictors in Kenya

Variable	Category	E-value for Estimate	E-value for 95% CI
Relationship with mother	(Ref: Very bad/somewhat bad)		
	Very good/somewhat good	1.48	1.00
Relationship with father	(Ref: Very bad/somewhat bad)		
	Very good/somewhat good	1.37	1.00
Parent marital status	(Ref: Parents married)		
	Divorced	1.69	1.00
	Parents were never married	1.87	1.00
	One or both parents had died	1.73	1.00
Subjective financial status of family growing up	(Ref: Got by)		
	Lived comfortably	1.99	1.00
	Found it difficult	1.16	1.00
	Found it very difficult	1.38	1.00
Abuse	(Ref: No)		
	Yes	3.08	2.15
Outsider growing up	(Ref: No)		
	Yes	2.25	1.41
Self-rated health growing up	(Ref: Good)		
	Excellent	1.37	1.00
	Very good	1.29	1.00
	Fair	1.83	1.00
	Poor	3.47	1.23
Immigration status	(Ref: Born in this country)		
	Born in another country	1.46	1.00
Age 12 religious service attendance	(Ref: Never)		
	At least 1/week	1.99	1.00
	1-3/month	2.12	1.00
	< 1/month	2.22	1.00
Year of birth	(Ref: 1998-2005; age: 18-24)		
	1988-1998; age 25-34	2.67	1.80
	1978-1988; age 35-44	3.65	2.46
	1968-1978; age 45-54	4.83	3.24
	1958-1968; age 55-64	5.08	3.30
	1948-1957; age 65-74	5.54	3.20
	1938-1948; age 75-84	1.50	1.00
	1938 or earlier; 85 or older	641931.18	297513.08
Gender	(Ref: Male)		
	Female	22.10	14.69
	Other	2269733.39	497199.76
Religious affiliation	(Ref: Christianity)		
	Islam	2.57	1.74
	Collapsed affiliations with prevalence<3%	3.52	1.70
Race/ethnicity	(Ref: Plurality group)		
	Non-plurality groups	3.03	2.13

45. Table S45. Nationally representative descriptive statistics for Mexico: Childhood predictors

Characteristic	N = 5,776[1]
**Relationship with mother**	
Very good	3,912 (68%)
Somewhat good	1,340 (23%)
Somewhat bad	177 (3.1%)
Very bad	90 (1.6%)
Does not apply	177 (3.1%)
(Missing)	80 (1.4%)
**Relationship with father**	
Very good	3,089 (53%)
Somewhat good	1,556 (27%)
Somewhat bad	335 (5.8%)
Very bad	267 (4.6%)
Does not apply	470 (8.1%)
(Missing)	60 (1.0%)
**Parent marital status**	
Parents married	3,999 (69%)
Divorced	341 (5.9%)
Parents were never married	827 (14%)
One or both parents had died	176 (3.0%)
(Missing)	432 (7.5%)
**Subjective financial status of family growing up**	
Lived comfortably	1,775 (31%)
Got by	1,872 (32%)
Found it difficult	1,712 (30%)
Found it very difficult	369 (6.4%)
(Missing)	48 (0.8%)
**Abuse**	
Yes	905 (16%)
No	4,604 (80%)
(Missing)	267 (4.6%)
**Outsider growing up**	
Yes	772 (13%)
No	4,897 (85%)
(Missing)	107 (1.9%)
**Self-rated health growing up**	
Excellent	1,860 (32%)
Very good	1,350 (23%)
Good	1,677 (29%)
Fair	743 (13%)
Poor	133 (2.3%)
(Missing)	14 (0.2%)
**Immigration status**	
Born in this country	5,517 (96%)
Born in another country	108 (1.9%)
(Missing)	151 (2.6%)
**Age 12 religious service attendance**	
At least 1/week	2,514 (44%)
1-3/month	1,162 (20%)
<1/month	1,087 (19%)
Never	944 (16%)
(Missing)	69 (1.2%)

46. Table S46. Nationally representative descriptive statistics for Mexico: Childhood predictors: Demographic variables

Characteristic	N = 5,776[1]
**Year of birth**	
1998-2005; age 18-24	986 (17%)
1988-1998; age 25-34	1,293 (22%)
1978-1988; age 35-44	1,158 (20%)
1968-1978; age 45-54	989 (17%)
1958-1968; age 55-64	697 (12%)
1948-1958; age 65-74	498 (8.6%)
1938-1948; age 75-84	131 (2.3%)
1938 or earlier; age 85+	24 (0.4%)
(Missing)	0 (0%)
**Gender**	
Male	2,755 (48%)
Female	2,997 (52%)
Other	3 (<0.1%)
(Missing)	21 (0.4%)
**Religious affiliation**	
Christianity	5,337 (92%)
Islam	6 (<0.1%)
Hinduism	1 (<0.1%)
Buddhism	1 (<0.1%)
Judaism	8 (0.1%)
Sikhism	4 (<0.1%)
Baha'i	1 (<0.1%)
Jainism	0 (0%)
Shinto	2 (<0.1%)
Taoism	5 (<0.1%)
Confucianism	0 (0%)
Primal, Animist, or Folk religion	2 (<0.1%)
Spiritism	0 (0%)
Umbanda, Candomble, and other African-derived religions	0 (0%)
Chinese folk/traditional religion	0 (0%)
Some other religion	7 (0.1%)
No religion/Atheist/Agnostic	328 (5.7%)
(Missing)	74 (1.3%)
**Race/Ethnicity**	
Black	108 (1.9%)
Indigenous	594 (10%)
Mestizo	2,762 (48%)
Mulatto	63 (1.1%)
Other	339 (5.9%)
White	1,116 (19%)
(Missing)	794 (14%)

[1]n (%)

47. Table S47. Childhood predictors regression for Mexico

Variable	Category	Risk-Ratio	RR 95% CI	Global p-value
Relationship with mother	(Ref: Very bad/somewhat bad)			0.371
	Very good/somewhat good	0.90	(0.72,1.14)	
Relationship with father	(Ref: Very bad/somewhat bad)			0.244
	Very good/somewhat good	1.12	(0.92,1.36)	
Parent marital status	(Ref: Parents married)			0.017
	Divorced	1.34	(1.10,1.64)	
	Parents were never married	1.10	(0.93,1.30)	
	One or both parents had died	1.08	(0.79,1.48)	
Subjective financial status of family growing up	(Ref: Got by)			9.18e-05
	Lived comfortably	1.18	(1.02,1.37)	
	Found it difficult	0.86	(0.73,1.02)	
	Found it very difficult	0.64	(0.45,0.90)	
Abuse	(Ref: No)			0.110
	Yes	1.15	(0.96,1.37)	
Outsider growing up	(Ref: No)			0.019
	Yes	1.21	(1.03,1.42)	
Self-rated health growing up	(Ref: Good)			0.091
	Excellent	1.20	(1.02,1.40)	
	Very good	1.09	(0.91,1.30)	
	Fair	0.92	(0.72,1.17)	
	Poor	1.19	(0.77,1.84)	
Immigration status	(Ref: Born in this country)			0.060
	Born in another country	1.40	(0.98,1.98)	
Age 12 religious service attendance	(Ref: Never)			0.026
	At least 1/week	0.80	(0.67,0.95)	
	1-3/month	0.97	(0.81,1.17)	
	< 1/month	0.94	(0.78,1.14)	
Year of birth	(Ref: 1998-2005; age: 18-24)			0.021
	1988-1998; age 25-34	1.20	(1.01,1.42)	
	1978-1988; age 35-44	1.18	(0.98,1.41)	
	1968-1978; age 45-54	0.87	(0.69,1.09)	
	1958-1968; age 55-64	1.04	(0.80,1.34)	
	1948-1957; age 65-74	0.96	(0.69,1.34)	
	1938-1948; age 75-84	0.77	(0.43,1.40)	
	1938 or earlier; 85 or older	0.61	(0.23,1.65)	
Gender	(Ref: Male)			0.000
	Female	0.44	(0.38,0.50)	
	Other	1.02	(0.39,2.63)	
Religious affiliation	(Ref: No religion/Atheist/Agnostic)			0.532
	Christianity	0.95	(0.77,1.16)	
	Collapsed affiliations with prevalence<3%	1.16	(0.72,1.85)	
Race/ethnicity	(Ref: Plurality group)			0.548
	Non-plurality groups	0.98	(0.85,1.13)	

48. Table S48. Sensitivity to unmeasured confounding of childhood predictors in Mexico

Variable	Category	E-value for Estimate	E-value for 95% CI
Relationship with mother	(Ref: Very bad/somewhat bad)		
	Very good/somewhat good	1.45	1.00
Relationship with father	(Ref: Very bad/somewhat bad)		
	Very good/somewhat good	1.48	1.00
Parent marital status	(Ref: Parents married)		
	Divorced	2.02	1.42
	Parents were never married	1.42	1.00
	One or both parents had died	1.38	1.00
Subjective financial status of family growing up	(Ref: Got by)		
	Lived comfortably	1.65	1.18
	Found it difficult	1.60	1.00
	Found it very difficult	2.50	1.46
Abuse	(Ref: No)		
	Yes	1.55	1.00
Outsider growing up	(Ref: No)		
	Yes	1.71	1.20
Self-rated health growing up	(Ref: Good)		
	Excellent	1.68	1.16
	Very good	1.40	1.00
	Fair	1.40	1.00
	Poor	1.66	1.00
Immigration status	(Ref: Born in this country)		
	Born in another country	2.14	1.00
Age 12 religious service attendance	(Ref: Never)		
	At least 1/week	1.82	1.28
	1-3/month	1.19	1.00
	< 1/month	1.32	1.00
Year of birth	(Ref: 1998-2005; age: 18-24)		
	1988-1998; age 25-34	1.69	1.12
	1978-1988; age 35-44	1.63	1.00
	1968-1978; age 45-54	1.56	1.00
	1958-1968; age 55-64	1.24	1.00
	1948-1957; age 65-74	1.24	1.00
	1938-1948; age 75-84	1.90	1.00
	1938 or earlier; 85 or older	2.66	1.00
Gender	(Ref: Male)		
	Female	4.00	3.41
	Other	1.15	1.00
Religious affiliation	(Ref: No religion/Atheist/Agnostic)		
	Christianity	1.30	1.00
	Collapsed affiliations with prevalence<3%	1.58	1.00
Race/ethnicity	(Ref: Plurality group)		
	Non-plurality groups	1.16	1.00

49. Table S49. Nationally representative descriptive statistics for Nigeria: Childhood predictors

Characteristic	N = 6,827[1]
**Relationship with mother**	
Very good	5,986 (88%)
Somewhat good	648 (9.5%)
Somewhat bad	62 (0.9%)
Very bad	18 (0.3%)
Does not apply	104 (1.5%)
(Missing)	9 (0.1%)
**Relationship with father**	
Very good	5,578 (82%)
Somewhat good	924 (14%)
Somewhat bad	76 (1.1%)
Very bad	43 (0.6%)
Does not apply	177 (2.6%)
(Missing)	29 (0.4%)
**Parent marital status**	
Parents married	5,568 (82%)
Divorced	307 (4.5%)
Parents were never married	335 (4.9%)
One or both parents had died	462 (6.8%)
(Missing)	154 (2.3%)
**Subjective financial status of family growing up**	
Lived comfortably	2,192 (32%)
Got by	2,381 (35%)
Found it difficult	1,661 (24%)
Found it very difficult	563 (8.3%)
(Missing)	29 (0.4%)
**Abuse**	
Yes	880 (13%)
No	5,851 (86%)
(Missing)	96 (1.4%)
**Outsider growing up**	
Yes	669 (9.8%)
No	6,059 (89%)
(Missing)	99 (1.5%)
**Self-rated health growing up**	
Excellent	2,644 (39%)
Very good	2,613 (38%)
Good	1,152 (17%)
Fair	306 (4.5%)
Poor	98 (1.4%)
(Missing)	14 (0.2%)
**Immigration status**	
Born in this country	6,779 (99%)
Born in another country	47 (0.7%)
(Missing)	1 (<0.1%)
**Age 12 religious service attendance**	
At least 1/week	5,907 (87%)
1-3/month	600 (8.8%)
<1/month	136 (2.0%)
Never	138 (2.0%)
(Missing)	45 (0.7%)

50. **Table S50. Nationally representative descriptive statistics for Nigeria: Demographic variables**

**Characteristic**	**N = 6,827**[1]
**Year of birth**	
1998-2005; age 18-24	1,533 (22%)
1988-1998; age 25-34	2,145 (31%)
1978-1988; age 35-44	1,552 (23%)
1968-1978; age 45-54	873 (13%)
1958-1968; age 55-64	419 (6.1%)
1948-1958; age 65-74	224 (3.3%)
1938-1948; age 75-84	63 (0.9%)
1938 or earlier; age 85+	19 (0.3%)
(Missing)	0 (0%)
**Gender**	
Male	3,371 (49%)
Female	3,456 (51%)
Other	0 (<0.1%)
(Missing)	0 (0%)
**Religious affiliation**	
Christianity	3,463 (51%)
Islam	3,314 (49%)
Hinduism	0 (0%)
Buddhism	0 (<0.1%)
Judaism	0 (0%)
Sikhism	0 (0%)
Baha'i	0 (0%)
Jainism	0 (0%)
Shinto	0 (0%)
Taoism	0 (0%)
Confucianism	0 (<0.1%)
Primal, Animist, or Folk religion	17 (0.3%)
Spiritism	0 (0%)
Umbanda, Candomble, and other African-derived religions	0 (0%)
Chinese folk/traditional religion	0 (0%)
Some other religion	0 (0%)
No religion/Atheist/Agnostic	19 (0.3%)
(Missing)	14 (0.2%)
**Race/Ethnicity**	
Edo	116 (1.7%)
Efik	48 (0.7%)
Fulani	266 (3.9%)
Hausa	2,342 (34%)
Ibibio	180 (2.6%)
Idoma	61 (0.9%)
Igala	77 (1.1%)
Igbo (Ibo)	1,111 (16%)
Ijaw	110 (1.6%)
Kanuri	31 (0.5%)
Other	1,014 (15%)
Tiv	198 (2.9%)
Urhobo	38 (0.6%)
Yoruba	1,230 (18%)
(Missing)	4 (<0.1%)

[1]n (%)

51. Table S51. Childhood predictors regression for Nigeria

Variable	Category	Risk-Ratio	RR 95% CI	Global p-value
Relationship with mother	(Ref: Very bad/somewhat bad)			0.704
	Very good/somewhat good	1.24	(0.41,3.80)	
Relationship with father	(Ref: Very bad/somewhat bad)			0.185
	Very good/somewhat good	0.62	(0.31,1.26)	
Parent marital status	(Ref: Parents married)			0.010
	Divorced	0.77	(0.41,1.45)	
	Parents were never married	0.75	(0.40,1.41)	
	One or both parents had died	2.05	(1.26,3.32)	
Subjective financial status of family growing up	(Ref: Got by)			0.521
	Lived comfortably	0.92	(0.58,1.44)	
	Found it difficult	1.24	(0.84,1.81)	
	Found it very difficult	1.32	(0.79,2.20)	
Abuse	(Ref: No)			5.12e-04
	Yes	1.81	(1.29,2.53)	
Outsider growing up	(Ref: No)			0.524
	Yes	1.15	(0.74,1.76)	
Self-rated health growing up	(Ref: Good)			0.240
	Excellent	0.72	(0.48,1.09)	
	Very good	0.73	(0.50,1.05)	
	Fair	0.60	(0.26,1.40)	
	Poor	0.95	(0.48,1.87)	
Immigration status	(Ref: Born in this country)			0.282
	Born in another country	0.32	(0.04,2.53)	
Age 12 religious service attendance	(Ref: Never)			0.106
	At least 1/week	1.82	(0.42,7.92)	
	1-3/month	2.52	(0.57,11.10)	
	< 1/month	2.56	(0.53,12.35)	
Year of birth	(Ref: 1998-2005; age: 18-24)			0.002
	1988-1998; age 25-34	1.17	(0.85,1.62)	
	1978-1988; age 35-44	1.92	(1.26,2.95)	
	1968-1978; age 45-54	1.18	(0.72,1.93)	
	1958-1968; age 55-64	1.03	(0.41,2.61)	
	1948-1957; age 65-74	0.18	(0.02,1.32)	
	1938-1948; age 75-84	3.29	(0.93,11.63)	
	1938 or earlier; 85 or older	8.95	(2.07,38.63)	
Gender	(Ref: Male)			0.000
	Female	0.13	(0.09,0.19)	
	Other	0.00	(0.00,0.00)	
Religious affiliation	(Ref: Christianity)			0.362
	Islam	1.34	(0.79,2.27)	
	Collapsed affiliations with prevalence<3%	0.43	(0.04,4.27)	
Race/ethnicity	(Ref: Plurality group)			0.638
	Non-plurality groups	1.10	(0.63,1.90)	

52. **Table S52. Sensitivity to unmeasured confounding of childhood predictors in Nigeria**

Variable	Category	E-value for Estimate	E-value for 95% CI
Relationship with mother	(Ref: Very bad/somewhat bad)		
	Very good/somewhat good	1.79	1.00
Relationship with father	(Ref: Very bad/somewhat bad)		
	Very good/somewhat good	2.59	1.00
Parent marital status	(Ref: Parents married)		
	Divorced	1.91	1.00
	Parents were never married	1.98	1.00
	One or both parents had died	3.51	1.83
Subjective financial status of family growing up	(Ref: Got by)		
	Lived comfortably	1.41	1.00
	Found it difficult	1.78	1.00
	Found it very difficult	1.96	1.00
Abuse	(Ref: No)		
	Yes	3.01	1.90
Outsider growing up	(Ref: No)		
	Yes	1.56	1.00
Self-rated health growing up	(Ref: Good)		
	Excellent	2.11	1.00
	Very good	2.09	1.00
	Fair	2.73	1.00
	Poor	1.29	1.00
Immigration status	(Ref: Born in this country)		
	Born in another country	5.68	1.00
Age 12 religious service attendance	(Ref: Never)		
	At least 1/week	3.04	1.00
	1-3/month	4.47	1.00
	< 1/month	4.56	1.00
Year of birth	(Ref: 1998-2005; age: 18-24)		
	1988-1998; age 25-34	1.62	1.00
	1978-1988; age 35-44	3.26	1.82
	1968-1978; age 45-54	1.65	1.00
	1958-1968; age 55-64	1.21	1.00
	1948-1957; age 65-74	10.78	1.00
	1938-1948; age 75-84	6.04	1.00
	1938 or earlier; 85 or older	17.38	3.56
Gender	(Ref: Male)		
	Female	14.78	10.25
	Other	13199.63	1772.26
Religious affiliation	(Ref: Christianity)		
	Islam	2.01	1.00
	Collapsed affiliations with prevalence<3%	4.12	1.00
Race/ethnicity	(Ref: Plurality group)		
	Non-plurality groups	1.43	1.00

53. **Table S53. Nationally representative descriptive statistics for Philippines: Childhood predictors**

Characteristic	N = 5,292[1]
**Relationship with mother**	
Very good	3,333 (63%)
Somewhat good	1,703 (32%)
Somewhat bad	124 (2.3%)
Very bad	39 (0.7%)
Does not apply	59 (1.1%)
(Missing)	35 (0.7%)
**Relationship with father**	
Very good	3,443 (65%)
Somewhat good	1,429 (27%)
Somewhat bad	159 (3.0%)
Very bad	58 (1.1%)
Does not apply	108 (2.0%)
(Missing)	95 (1.8%)
**Parent marital status**	
Parents married	4,575 (86%)
Divorced	64 (1.2%)
Parents were never married	517 (9.8%)
One or both parents had died	51 (1.0%)
(Missing)	85 (1.6%)
**Subjective financial status of family growing up**	
Lived comfortably	937 (18%)
Got by	3,006 (57%)
Found it difficult	1,055 (20%)
Found it very difficult	291 (5.5%)
(Missing)	3 (<0.1%)
**Abuse**	
Yes	420 (7.9%)
No	4,837 (91%)
(Missing)	35 (0.7%)
**Outsider growing up**	
Yes	395 (7.5%)
No	4,884 (92%)
(Missing)	13 (0.2%)
**Self-rated health growing up**	
Excellent	1,041 (20%)
Very good	559 (11%)
Good	2,174 (41%)
Fair	1,246 (24%)
Poor	272 (5.1%)
(Missing)	0 (<0.1%)
**Immigration status**	
Born in this country	5,284 (100%)
Born in another country	8 (0.1%)
(Missing)	0 (0%)
**Age 12 religious service attendance**	
At least 1/week	2,453 (46%)
1-3/month	1,699 (32%)
<1/month	892 (17%)
Never	201 (3.8%)
(Missing)	47 (0.9%)

54. **Table S54. Nationally representative descriptive statistics for Philippines: Demographic variables**

Characteristic	N = 5,292[1]
**Year of birth**	
1998-2005; age 18-24	1,073 (20%)
1988-1998; age 25-34	1,322 (25%)
1978-1988; age 35-44	1,058 (20%)
1968-1978; age 45-54	813 (15%)
1958-1968; age 55-64	641 (12%)
1948-1958; age 65-74	331 (6.3%)
1938-1948; age 75-84	51 (1.0%)
1938 or earlier; age 85+	4 (<0.1%)
(Missing)	0 (0%)
**Gender**	
Male	2,625 (50%)
Female	2,643 (50%)
Other	13 (0.2%)
(Missing)	11 (0.2%)
**Religious affiliation**	
Christianity	4,968 (94%)
Islam	276 (5.2%)
Hinduism	0 (0%)
Buddhism	1 (<0.1%)
Judaism	0 (0%)
Sikhism	4 (<0.1%)
Baha'i	1 (<0.1%)
Jainism	0 (0%)
Shinto	0 (0%)
Taoism	0 (0%)
Confucianism	0 (0%)
Primal, Animist, or Folk religion	14 (0.3%)
Spiritism	0 (0%)
Umbanda, Candomble, and other African-derived religions	0 (0%)
Chinese folk/traditional religion	0 (0%)
Some other religion	9 (0.2%)
No religion/Atheist/Agnostic	9 (0.2%)
(Missing)	11 (0.2%)
**Race/Ethnicity**	
Aeta	1 (<0.1%)
Badjao	2 (<0.1%)
Bicolano/Bikolano	300 (5.7%)
Cebuano	656 (12%)
Chinese-Filipino	3 (<0.1%)
Igorot	42 (0.8%)
Ilocano/Ilokano	429 (8.1%)
Ilonggo/Hiligaynon	428 (8.1%)
Kapampangan	107 (2.0%)
Maguindanaoan	84 (1.6%)
Mangyan	2 (<0.1%)
Maranao	39 (0.7%)
Masbateno	54 (1.0%)
Other	244 (4.6%)
Pangasinense	107 (2.0%)
Tagalog	1,691 (32%)
Tausug	94 (1.8%)
Visayan/Bisaya	739 (14%)
Waray	216 (4.1%)
Zamboangueno	51 (1.0%)
(Missing)	3 (<0.1%)

55. Table S55. Childhood predictors regression for Philippines

Variable	Category	Risk-Ratio	RR 95% CI	Global p-value
Relationship with mother	(Ref: Very bad/somewhat bad)			0.810
	Very good/somewhat good	1.04	(0.73,1.48)	
Relationship with father	(Ref: Very bad/somewhat bad)			0.456
	Very good/somewhat good	0.90	(0.67,1.21)	
Parent marital status	(Ref: Parents married)			0.079
	Divorced	1.26	(0.80,1.98)	
	Parents were never married	1.23	(1.02,1.48)	
	One or both parents had died	1.27	(0.74,2.17)	
Subjective financial status of family growing up	(Ref: Got by)			0.005
	Lived comfortably	1.07	(0.91,1.27)	
	Found it difficult	0.81	(0.68,0.96)	
	Found it very difficult	1.22	(0.97,1.53)	
Abuse	(Ref: No)			0.432
	Yes	1.10	(0.87,1.39)	
Outsider growing up	(Ref: No)			0.003
	Yes	1.42	(1.13,1.78)	
Self-rated health growing up	(Ref: Good)			0.953
	Excellent	0.99	(0.83,1.20)	
	Very good	0.96	(0.76,1.21)	
	Fair	1.05	(0.89,1.22)	
	Poor	0.97	(0.72,1.31)	
Immigration status	(Ref: Born in this country)			0.639
	Born in another country	1.21	(0.48,3.02)	
Age 12 religious service attendance	(Ref: Never)			0.173
	At least 1/week	0.84	(0.62,1.15)	
	1-3/month	0.92	(0.67,1.26)	
	< 1/month	1.01	(0.74,1.37)	
Year of birth	(Ref: 1998-2005; age: 18-24)			0.000
	1988-1998; age 25-34	1.66	(1.32,2.08)	
	1978-1988; age 35-44	1.72	(1.35,2.20)	
	1968-1978; age 45-54	1.43	(1.10,1.85)	
	1958-1968; age 55-64	1.36	(1.00,1.85)	
	1948-1957; age 65-74	1.06	(0.68,1.64)	
	1938-1948; age 75-84	0.99	(0.56,1.77)	
	1938 or earlier; 85 or older	0.00	(0.00,0.00)	
Gender	(Ref: Male)			0.000
	Female	0.15	(0.13,0.18)	
	Other	0.18	(0.03,1.17)	
Religious affiliation	(Ref: Christianity)			0.438
	Islam	1.18	(0.91,1.52)	
	Collapsed affiliations with prevalence<3%	0.94	(0.49,1.82)	
Race/ethnicity	(Ref: Plurality group)			0.020
	Non-plurality groups	0.85	(0.73,0.98)	

56. **Table S56. Sensitivity to unmeasured confounding of childhood predictors in Philippines**

Variable	Category	E-value for Estimate	E-value for 95% CI
Relationship with mother	(Ref: Very bad/somewhat bad)		
	Very good/somewhat good	1.24	1.00
Relationship with father	(Ref: Very bad/somewhat bad)		
	Very good/somewhat good	1.46	1.00
Parent marital status	(Ref: Parents married)		
	Divorced	1.83	1.00
	Parents were never married	1.76	1.18
	One or both parents had died	1.85	1.00
Subjective financial status of family growing up	(Ref: Got by)		
	Lived comfortably	1.35	1.00
	Found it difficult	1.77	1.24
	Found it very difficult	1.73	1.00
Abuse	(Ref: No)		
	Yes	1.43	1.00
Outsider growing up	(Ref: No)		
	Yes	2.18	1.51
Self-rated health growing up	(Ref: Good)		
	Excellent	1.08	1.00
	Very good	1.25	1.00
	Fair	1.27	1.00
	Poor	1.21	1.00
Immigration status	(Ref: Born in this country)		
	Born in another country	1.71	1.00
Age 12 religious service attendance	(Ref: Never)		
	At least 1/week	1.66	1.00
	1-3/month	1.41	1.00
	< 1/month	1.08	1.00
Year of birth	(Ref: 1998-2005; age: 18-24)		
	1988-1998; age 25-34	2.70	1.98
	1978-1988; age 35-44	2.84	2.03
	1968-1978; age 45-54	2.20	1.43
	1958-1968; age 55-64	2.07	1.06
	1948-1957; age 65-74	1.30	1.00
	1938-1948; age 75-84	1.09	1.00
	1938 or earlier; 85 or older	166560.24	25965.68
Gender	(Ref: Male)		
	Female	12.64	10.57
	Other	10.87	1.00
Religious affiliation	(Ref: Christianity)		
	Islam	1.63	1.00
	Collapsed affiliations with prevalence<3%	1.32	1.00
Race/ethnicity	(Ref: Plurality group)		
	Non-plurality groups	1.64	1.17

57. Table S57. Nationally representative descriptive statistics for Poland: Childhood predictors

**Characteristic**	**N = 10,389**[1]
**Relationship with mother**	
Very good	4,879 (47%)
Somewhat good	4,973 (48%)
Somewhat bad	285 (2.7%)
Very bad	58 (0.6%)
Does not apply	80 (0.8%)
(Missing)	112 (1.1%)
**Relationship with father**	
Very good	4,231 (41%)
Somewhat good	4,984 (48%)
Somewhat bad	516 (5.0%)
Very bad	78 (0.7%)
Does not apply	407 (3.9%)
(Missing)	173 (1.7%)
**Parent marital status**	
Parents married	8,972 (86%)
Divorced	587 (5.7%)
Parents were never married	193 (1.9%)
One or both parents had died	313 (3.0%)
(Missing)	324 (3.1%)
**Subjective financial status of family growing up**	
Lived comfortably	1,384 (13%)
Got by	6,257 (60%)
Found it difficult	2,133 (21%)
Found it very difficult	509 (4.9%)
(Missing)	106 (1.0%)
**Abuse**	
Yes	325 (3.1%)
No	10,009 (96%)
(Missing)	55 (0.5%)
**Outsider growing up**	
Yes	490 (4.7%)
No	9,615 (93%)
(Missing)	284 (2.7%)
**Self-rated health growing up**	
Excellent	2,676 (26%)
Very good	5,371 (52%)
Good	1,779 (17%)
Fair	406 (3.9%)
Poor	123 (1.2%)
(Missing)	34 (0.3%)
**Immigration status**	
Born in this country	10,258 (99%)
Born in another country	108 (1.0%)
(Missing)	23 (0.2%)
**Age 12 religious service attendance**	
At least 1/week	4,751 (46%)
1-3/month	2,689 (26%)
<1/month	2,161 (21%)
Never	354 (3.4%)
(Missing)	434 .2%)

58. Table S58. Nationally representative descriptive statistics for Poland: Demographic variables

Characteristic	N = 10,389[1]
**Year of birth**	
1998-2005; age 18-24	955 (9.2%)
1988-1998; age 25-34	1,822 (18%)
1978-1988; age 35-44	2,139 (21%)
1968-1978; age 45-54	1,722 (17%)
1958-1968; age 55-64	1,678 (16%)
1948-1958; age 65-74	1,672 (16%)
1938-1948; age 75-84	352 (3.4%)
1938 or earlier; age 85+	47 (0.5%)
(Missing)	1 (<0.1%)
**Gender**	
Male	4,974 (48%)
Female	5,387 (52%)
Other	3 (<0.1%)
(Missing)	26 (0.2%)
**Religious affiliation**	
Christianity	9,861 (95%)
Islam	3 (<0.1%)
Hinduism	0 (0%)
Buddhism	2 (<0.1%)
Judaism	0 (0%)
Sikhism	1 (<0.1%)
Baha'i	0 (0%)
Jainism	0 (0%)
Shinto	0 (0%)
Taoism	0 (0%)
Confucianism	0 (0%)
Primal, Animist, or Folk religion	5 (<0.1%)
Spiritism	0 (0%)
Umbanda, Candomble, and other African-derived religions	0 (0%)
Chinese folk/traditional religion	0 (0%)
Some other religion	0 (0%)
No religion/Atheist/Agnostic	482 (4.6%)
(Missing)	35 (0.3%)
**Race/Ethnicity**	
Belarussian	2 (<0.1%)
German	4 (<0.1%)
Kashubians	3 (<0.1%)
Other	4 (<0.1%)
Polish	10,309 (99%)
Silesia	14 (0.1%)
Ukrainian	38 (0.4%)
(Missing)	14 (0.1%)

[1]n (%)

59. Table S59. Childhood predictors regression for Poland

Variable	Category	Risk-Ratio	RR 95% CI	Global p-value
Relationship with mother	(Ref: Very bad/somewhat bad)			0.300
	Very good/somewhat good	0.90	(0.74,1.10)	
Relationship with father	(Ref: Very bad/somewhat bad)			0.106
	Very good/somewhat good	0.88	(0.74,1.04)	
Parent marital status	(Ref: Parents married)			1.94e-08
	Divorced	1.44	(1.27,1.63)	
	Parents were never married	1.43	(1.18,1.72)	
	One or both parents had died	1.06	(0.81,1.40)	
Subjective financial status of family growing up	(Ref: Got by)			0.168
	Lived comfortably	1.08	(0.96,1.22)	
	Found it difficult	1.00	(0.89,1.13)	
	Found it very difficult	1.23	(0.99,1.55)	
Abuse	(Ref: No)			0.680
	Yes	1.04	(0.86,1.26)	
Outsider growing up	(Ref: No)			0.656
	Yes	1.02	(0.83,1.25)	
Self-rated health growing up	(Ref: Good)			0.002
	Excellent	0.91	(0.79,1.04)	
	Very good	0.79	(0.69,0.90)	
	Fair	0.95	(0.75,1.20)	
	Poor	1.05	(0.65,1.69)	
Immigration status	(Ref: Born in this country)			0.008
	Born in another country	1.58	(1.12,2.24)	
Age 12 religious service attendance	(Ref: Never)			1.73e-04
	At least 1/week	0.74	(0.60,0.92)	
	1-3/month	0.91	(0.73,1.13)	
	< 1/month	0.92	(0.75,1.13)	
Year of birth	(Ref: 1998-2005; age: 18-24)			2.74e-07
	1988-1998; age 25-34	1.19	(0.98,1.44)	
	1978-1988; age 35-44	1.24	(1.03,1.50)	
	1968-1978; age 45-54	1.35	(1.11,1.65)	
	1958-1968; age 55-64	1.37	(1.11,1.68)	
	1948-1957; age 65-74	0.85	(0.67,1.07)	
	1938-1948; age 75-84	0.47	(0.26,0.84)	
	1938 or earlier; 85 or older	0.51	(0.15,1.75)	
Gender	(Ref: Male)			0.000
	Female	0.56	(0.51,0.61)	
	Other	2.32	(1.86,2.90)	
Religious affiliation	(Ref: No religion/Atheist/Agnostic)			0.822
	Christianity	0.98	(0.80,1.19)	
	Collapsed affiliations with prevalence<3%	0.60	(0.10,3.79)	
Race/ethnicity	(Ref: Plurality group)			0.204
	Non-plurality groups	1.25	(0.89,1.75)	

60. Table S60. Sensitivity to unmeasured confounding of childhood predictors in Poland

Variable	Category	E-value for Estimate	E-value for 95% CI
Relationship with mother	(Ref: Very bad/somewhat bad)		
	Very good/somewhat good	1.46	1.00
Relationship with father	(Ref: Very bad/somewhat bad)		
	Very good/somewhat good	1.54	1.00
Parent marital status	(Ref: Parents married)		
	Divorced	2.23	1.86
	Parents were never married	2.21	1.65
	One or both parents had died	1.32	1.00
Subjective financial status of family growing up	(Ref: Got by)		
	Lived comfortably	1.39	1.00
	Found it difficult	1.06	1.00
	Found it very difficult	1.77	1.00
Abuse	(Ref: No)		
	Yes	1.24	1.00
Outsider growing up	(Ref: No)		
	Yes	1.14	1.00
Self-rated health growing up	(Ref: Good)		
	Excellent	1.44	1.00
	Very good	1.85	1.46
	Fair	1.30	1.00
	Poor	1.29	1.00
Immigration status	(Ref: Born in this country)		
	Born in another country	2.55	1.49
Age 12 religious service attendance	(Ref: Never)		
	At least 1/week	2.04	1.40
	1-3/month	1.43	1.00
	< 1/month	1.38	1.00
Year of birth	(Ref: 1998-2005; age: 18-24)		
	1988-1998; age 25-34	1.67	1.00
	1978-1988; age 35-44	1.79	1.20
	1968-1978; age 45-54	2.05	1.47
	1958-1968; age 55-64	2.07	1.47
	1948-1957; age 65-74	1.65	1.00
	1938-1948; age 75-84	3.72	1.68
	1938 or earlier; 85 or older	3.33	1.00
Gender	(Ref: Male)		
	Female	3.00	2.66
	Other	4.07	3.13
Religious affiliation	(Ref: No religion/Atheist/Agnostic)		
	Christianity	1.18	1.00
	Collapsed affiliations with prevalence<3%	2.71	1.00
Race/ethnicity	(Ref: Plurality group)		
	Non-plurality groups	1.80	1.00

61. **Table S61. Nationally representative descriptive statistics for South Africa: Childhood predictors**

Characteristic	N = 2,651[1]
**Relationship with mother**	
Very good	2,186 (82%)
Somewhat good	263 (9.9%)
Somewhat bad	51 (1.9%)
Very bad	39 (1.5%)
Does not apply	90 (3.4%)
(Missing)	21 (0.8%)
**Relationship with father**	
Very good	1,656 (62%)
Somewhat good	333 (13%)
Somewhat bad	86 (3.3%)
Very bad	159 (6.0%)
Does not apply	331 (12%)
(Missing)	85 (3.2%)
**Parent marital status**	
Parents married	1,321 (50%)
Divorced	131 (5.0%)
Parents were never married	904 (34%)
One or both parents had died	140 (5.3%)
(Missing)	155 (5.8%)
**Subjective financial status of family growing up**	
Lived comfortably	1,050 (40%)
Got by	875 (33%)
Found it difficult	432 (16%)
Found it very difficult	289 (11%)
(Missing)	5 (0.2%)
**Abuse**	
Yes	450 (17%)
No	2,149 (81%)
(Missing)	52 (2.0%)
**Outsider growing up**	
Yes	434 (16%)
No	2,211 (83%)
(Missing)	6 (0.2%)
**Self-rated health growing up**	
Excellent	1,225 (46%)
Very good	590 (22%)
Good	370 (14%)
Fair	266 (10%)
Poor	183 (6.9%)
(Missing)	17 (0.6%)
**Immigration status**	
Born in this country	2,511 (95%)
Born in another country	139 (5.2%)
(Missing)	1 (<0.1%)
**Age 12 religious service attendance**	
At least 1/week	1,681 (63%)
1-3/month	552 (21%)
<1/month	175 (6.6%)
Never	217 (8.2%)
(Missing)	26 (1.0%)

62. Table S62. Nationally representative descriptive statistics for South Africa: Demographic variables

Characteristic	N = 2,651[1]
**Year of birth**	
1998-2005; age 18-24	461 (17%)
1988-1998; age 25-34	709 (27%)
1978-1988; age 35-44	611 (23%)
1968-1978; age 45-54	381 (14%)
1958-1968; age 55-64	261 (9.9%)
1948-1958; age 65-74	158 (6.0%)
1938-1948; age 75-84	58 (2.2%)
1938 or earlier; age 85+	2 (<0.1%)
(Missing)	9 (0.3%)
**Gender**	
Male	1,288 (49%)
Female	1,356 (51%)
Other	2 (<0.1%)
(Missing)	4 (0.2%)
**Religious affiliation**	
Christianity	2,323 (88%)
Islam	52 (2.0%)
Hinduism	2 (<0.1%)
Buddhism	11 (0.4%)
Judaism	0 (0%)
Sikhism	0 (0%)
Baha'i	0 (0%)
Jainism	0 (0%)
Shinto	2 (<0.1%)
Taoism	1 (<0.1%)
Confucianism	0 (0%)
Primal, Animist, or Folk religion	117 (4.4%)
Spiritism	0 (0%)
Umbanda, Candomble, and other African-derived religions	0 (0%)
Chinese folk/traditional religion	0 (0%)
Some other religion	7 (0.3%)
No religion/Atheist/Agnostic	107 (4.1%)
(Missing)	27 (1.0%)
**Race/Ethnicity**	
Asian/Indian	6 (0.2%)
Black	2,381 (90%)
Colored	252 (9.5%)
Other	1 (<0.1%)
White	8 (0.3%)
(Missing)	3 (0.1%)

[1]n (%)

63. Table S63. Childhood predictors regression for South Africa

Variable	Category	Risk-Ratio	RR 95% CI	Global p-value
Relationship with mother	(Ref: Very bad/somewhat bad)			0.887
	Very good/somewhat good	0.97	(0.58,1.64)	
Relationship with father	(Ref: Very bad/somewhat bad)			0.299
	Very good/somewhat good	0.88	(0.69,1.13)	
Parent marital status	(Ref: Parents married)			0.270
	Divorced	0.97	(0.66,1.41)	
	Parents were never married	0.88	(0.72,1.07)	
	One or both parents had died	1.17	(0.78,1.76)	
Subjective financial status of family growing up	(Ref: Got by)			0.396
	Lived comfortably	0.98	(0.81,1.19)	
	Found it difficult	1.12	(0.89,1.41)	
	Found it very difficult	0.81	(0.57,1.16)	
Abuse	(Ref: No)			0.083
	Yes	1.18	(0.98,1.44)	
Outsider growing up	(Ref: No)			0.282
	Yes	1.13	(0.90,1.41)	
Self-rated health growing up	(Ref: Good)			0.117
	Excellent	1.16	(0.92,1.45)	
	Very good	0.98	(0.75,1.29)	
	Fair	1.13	(0.81,1.60)	
	Poor	0.75	(0.47,1.19)	
Immigration status	(Ref: Born in this country)			0.017
	Born in another country	0.48	(0.26,0.87)	
Age 12 religious service attendance	(Ref: Never)			0.673
	At least 1/week	0.86	(0.54,1.38)	
	1-3/month	0.88	(0.56,1.39)	
	< 1/month	0.73	(0.43,1.25)	
Year of birth	(Ref: 1998-2005; age: 18-24)			0.067
	1988-1998; age 25-34	1.43	(1.08,1.91)	
	1978-1988; age 35-44	1.47	(1.09,2.00)	
	1968-1978; age 45-54	1.23	(0.88,1.72)	
	1958-1968; age 55-64	1.16	(0.79,1.71)	
	1948-1957; age 65-74	0.50	(0.20,1.22)	
	1938-1948; age 75-84	1.47	(0.66,3.26)	
	1938 or earlier; 85 or older	0.92	(0.23,3.69)	
Gender	(Ref: Male)			0.000
	Female	0.30	(0.24,0.38)	
	Other	1.44	(0.79,2.63)	
Religious affiliation	(Ref: No religion/Atheist/Agnostic)			0.210
	Primal, Animist, or Folk religion	0.76	(0.38,1.52)	
	Christianity	1.05	(0.57,1.90)	
	Collapsed affiliations with prevalence<3%	1.45	(0.73,2.89)	
Race/ethnicity	(Ref: Plurality group)			7.21e-04
	Non-plurality groups	2.09	(1.53,2.85)	

64. **Table S64. Sensitivity to unmeasured confounding of childhood predictors in South Africa**

Variable	Category	E-value for Estimate	E-value for 95% CI
Relationship with mother	(Ref: Very bad/somewhat bad)		
	Very good/somewhat good	1.19	1.00
Relationship with father	(Ref: Very bad/somewhat bad)		
	Very good/somewhat good	1.53	1.00
Parent marital status	(Ref: Parents married)		
	Divorced	1.23	1.00
	Parents were never married	1.53	1.00
	One or both parents had died	1.62	1.00
Subjective financial status of family growing up	(Ref: Got by)		
	Lived comfortably	1.15	1.00
	Found it difficult	1.49	1.00
	Found it very difficult	1.77	1.00
Abuse	(Ref: No)		
	Yes	1.65	1.00
Outsider growing up	(Ref: No)		
	Yes	1.52	1.00
Self-rated health growing up	(Ref: Good)		
	Excellent	1.59	1.00
	Very good	1.17	1.00
	Fair	1.52	1.00
	Poor	2.01	1.00
Immigration status	(Ref: Born in this country)		
	Born in another country	3.63	1.56
Age 12 religious service attendance	(Ref: Never)		
	At least 1/week	1.59	1.00
	1-3/month	1.53	1.00
	< 1/month	2.08	1.00
Year of birth	(Ref: 1998-2005; age: 18-24)		
	1988-1998; age 25-34	2.22	1.36
	1978-1988; age 35-44	2.31	1.39
	1968-1978; age 45-54	1.76	1.00
	1958-1968; age 55-64	1.60	1.00
	1948-1957; age 65-74	3.41	1.00
	1938-1948; age 75-84	2.30	1.00
	1938 or earlier; 85 or older	1.41	1.00
Gender	(Ref: Male)		
	Female	6.03	4.73
	Other	2.24	1.00
Religious affiliation	(Ref: No religion/Atheist/Agnostic)		
	Primal, Animist, or Folk religion	1.95	1.00
	Christianity	1.26	1.00
	Collapsed affiliations with prevalence<3%	2.25	1.00
Race/ethnicity	(Ref: Plurality group)		
	Non-plurality groups	3.59	2.43

65. Table S65. Nationally representative descriptive statistics for Spain: Childhood predictors

Characteristic	N = 6,290[1]
**Relationship with mother**	
Very good	4,557 (72%)
Somewhat good	1,258 (20%)
Somewhat bad	248 (3.9%)
Very bad	92 (1.5%)
Does not apply	107 (1.7%)
(Missing)	28 (0.4%)
**Relationship with father**	
Very good	4,131 (66%)
Somewhat good	1,397 (22%)
Somewhat bad	309 (4.9%)
Very bad	178 (2.8%)
Does not apply	243 (3.9%)
(Missing)	33 (0.5%)
**Parent marital status**	
Parents married	5,285 (84%)
Divorced	378 (6.0%)
Parents were never married	312 (5.0%)
One or both parents had died	126 (2.0%)
(Missing)	188 (3.0%)
**Subjective financial status of family growing up**	
Lived comfortably	2,041 (32%)
Got by	2,956 (47%)
Found it difficult	1,154 (18%)
Found it very difficult	110 (1.7%)
(Missing)	29 (0.5%)
**Abuse**	
Yes	659 (10%)
No	5,510 (88%)
(Missing)	122 (1.9%)
**Outsider growing up**	
Yes	579 (9.2%)
No	5,637 (90%)
(Missing)	75 (1.2%)
**Self-rated health growing up**	
Excellent	2,450 (39%)
Very good	2,286 (36%)
Good	1,235 (20%)
Fair	164 (2.6%)
Poor	135 (2.1%)
(Missing)	20 (0.3%)
**Immigration status**	
Born in this country	5,479 (87%)
Born in another country	788 (13%)
(Missing)	23 (0.4%)
**Age 12 religious service attendance**	
At least 1/week	2,391 (38%)
1-3/month	1,132 (18%)
<1/month	1,287 (20%)
Never	1,445 (23%)
(Missing)	36 (0.6%)

66. Table S66. Nationally representative descriptive statistics for Spain: Childhood predictors: Demographic variables

Characteristic	N = 6,290[1]
**Year of birth**	
1998-2005; age 18-24	594 (9.4%)
1988-1998; age 25-34	949 (15%)
1978-1988; age 35-44	1,278 (20%)
1968-1978; age 45-54	1,354 (22%)
1958-1968; age 55-64	1,096 (17%)
1948-1958; age 65-74	855 (14%)
1938-1948; age 75-84	162 (2.6%)
1938 or earlier; age 85+	3 (<0.1%)
(Missing)	0 (0%)
**Gender**	
Male	3,142 (50%)
Female	3,119 (50%)
Other	6 (0.1%)
(Missing)	22 (0.4%)
**Religious affiliation**	
Christianity	5,119 (81%)
Islam	132 (2.1%)
Hinduism	5 (<0.1%)
Buddhism	8 (0.1%)
Judaism	5 (<0.1%)
Sikhism	2 (<0.1%)
Baha'i	0 (0%)
Jainism	0 (0%)
Shinto	0 (0%)
Taoism	0 (0%)
Confucianism	1 (<0.1%)
Primal, Animist, or Folk religion	4 (<0.1%)
Spiritism	0 (0%)
Umbanda, Candomble, and other African-derived religions	0 (0%)
Chinese folk/traditional religion	0 (0%)
Some other religion	13 (0.2%)
No religion/Atheist/Agnostic	972 (15%)
(Missing)	29 (0.5%)

[1]n (%)

67. Table S67. Childhood predictors regression for Spain

Variable	Category	Risk-Ratio	RR 95% CI	Global p-value
Relationship with mother	(Ref: Very bad/somewhat bad)			0.700
	Very good/somewhat good	0.97	(0.82,1.16)	
Relationship with father	(Ref: Very bad/somewhat bad)			0.462
	Very good/somewhat good	0.95	(0.82,1.10)	
Parent marital status	(Ref: Parents married)			0.071
	Divorced	1.19	(1.02,1.38)	
	Parents were never married	0.98	(0.80,1.21)	
	One or both parents had died	0.87	(0.64,1.17)	
Subjective financial status of family growing up	(Ref: Got by)			0.294
	Lived comfortably	1.11	(0.98,1.25)	
	Found it difficult	1.00	(0.88,1.14)	
	Found it very difficult	1.11	(0.80,1.56)	
Abuse	(Ref: No)			2.97e-04
	Yes	1.28	(1.12,1.46)	
Outsider growing up	(Ref: No)			0.180
	Yes	1.09	(0.96,1.24)	
Self-rated health growing up	(Ref: Good)			0.086
	Excellent	0.86	(0.75,0.99)	
	Very good	0.92	(0.81,1.06)	
	Fair	0.83	(0.60,1.16)	
	Poor	0.68	(0.45,1.04)	
Immigration status	(Ref: Born in this country)			7.34e-06
	Born in another country	0.71	(0.61,0.82)	
Age 12 religious service attendance	(Ref: Never)			0.648
	At least 1/week	0.92	(0.81,1.05)	
	1-3/month	0.96	(0.84,1.11)	
	< 1/month	0.96	(0.84,1.10)	
Year of birth	(Ref: 1998-2005; age: 18-24)			1.17e-05
	1988-1998; age 25-34	1.29	(1.06,1.58)	
	1978-1988; age 35-44	1.47	(1.22,1.78)	
	1968-1978; age 45-54	1.34	(1.11,1.62)	
	1958-1968; age 55-64	1.24	(1.00,1.53)	
	1948-1957; age 65-74	1.01	(0.77,1.33)	
	1938-1948; age 75-84	0.76	(0.44,1.32)	
	1938 or earlier; 85 or older	2.35	(1.33,4.13)	
Gender	(Ref: Male)			2.63e-04
	Female	0.83	(0.75,0.91)	
	Other	1.34	(0.61,2.94)	
Religious affiliation	(Ref: No religion/Atheist/Agnostic)			0.132
	Christianity	1.02	(0.89,1.17)	
	Collapsed affiliations with prevalence<3%	1.28	(0.99,1.64)	
Race/ethnicity	(Ref: Plurality group)			

68. Table S68. Sensitivity to unmeasured confounding of childhood predictors in Spain

Variable	Category	E-value for Estimate	E-value for 95% CI
Relationship with mother	(Ref: Very bad/somewhat bad)		
	Very good/somewhat good	1.20	1.00
Relationship with father	(Ref: Very bad/somewhat bad)		
	Very good/somewhat good	1.30	1.00
Parent marital status	(Ref: Parents married)		
	Divorced	1.66	1.16
	Parents were never married	1.16	1.00
	One or both parents had died	1.57	1.00
Subjective financial status of family growing up	(Ref: Got by)		
	Lived comfortably	1.46	1.00
	Found it difficult	1.05	1.00
	Found it very difficult	1.47	1.00
Abuse	(Ref: No)		
	Yes	1.87	1.47
Outsider growing up	(Ref: No)		
	Yes	1.41	1.00
Self-rated health growing up	(Ref: Good)		
	Excellent	1.59	1.11
	Very good	1.39	1.00
	Fair	1.70	1.00
	Poor	2.30	1.00
Immigration status	(Ref: Born in this country)		
	Born in another country	2.18	1.73
Age 12 religious service attendance	(Ref: Never)		
	At least 1/week	1.39	1.00
	1-3/month	1.24	1.00
	< 1/month	1.25	1.00
Year of birth	(Ref: 1998-2005; age: 18-24)		
	1988-1998; age 25-34	1.90	1.30
	1978-1988; age 35-44	2.31	1.74
	1968-1978; age 45-54	2.02	1.45
	1958-1968; age 55-64	1.78	1.02
	1948-1957; age 65-74	1.11	1.00
	1938-1948; age 75-84	1.94	1.00
	1938 or earlier; 85 or older	4.13	2.00
Gender	(Ref: Male)		
	Female	1.71	1.43
	Other	2.01	1.00
Religious affiliation	(Ref: No religion/Atheist/Agnostic)		
	Christianity	1.16	1.00
	Collapsed affiliations with prevalence<3%	1.87	1.00
Race/ethnicity	(Ref: Plurality group)		

69. **Table S69. Nationally representative descriptive statistics for Sweden: Childhood predictors**

**Characteristic**	**N = 15,068**[1]
**Relationship with mother**	
Very good	8,743 (58%)
Somewhat good	4,513 (30%)
Somewhat bad	1,194 (7.9%)
Very bad	371 (2.5%)
Does not apply	216 (1.4%)
(Missing)	30 (0.2%)
**Relationship with father**	
Very good	7,134 (47%)
Somewhat good	4,885 (32%)
Somewhat bad	1,588 (11%)
Very bad	725 (4.8%)
Does not apply	720 (4.8%)
(Missing)	16 (0.1%)
**Parent marital status**	
Parents married	10,887 (72%)
Divorced	1,927 (13%)
Parents were never married	1,747 (12%)
One or both parents had died	362 (2.4%)
(Missing)	145 (1.0%)
**Subjective financial status of family growing up**	
Lived comfortably	5,951 (39%)
Got by	7,717 (51%)
Found it difficult	1,238 (8.2%)
Found it very difficult	140 (0.9%)
(Missing)	22 (0.1%)
**Abuse**	
Yes	2,288 (15%)
No	12,735 (85%)
(Missing)	45 (0.3%)
**Outsider growing up**	
Yes	1,867 (12%)
No	13,034 (86%)
(Missing)	168 (1.1%)
**Self-rated health growing up**	
Excellent	5,733 (38%)
Very good	5,124 (34%)
Good	2,669 (18%)
Fair	1,108 (7.4%)
Poor	397 (2.6%)
(Missing)	38 (0.2%)
**Immigration status**	
Born in this country	13,922 (92%)
Born in another country	1,052 (7.0%)
(Missing)	94 (0.6%)
**Age 12 religious service attendance**	
At least 1/week	955 (6.3%)
1-3/month	1,362 (9.0%)
<1/month	6,224 (41%)
Never	6,472 (43%)
(Missing)	54 (0.4%)

70. Table S70. Nationally representative descriptive statistics for Sweden: Demographic variables

Characteristic	N = 15,068[1]
**Year of birth**	
1998-2005; age 18-24	1,515 (10%)
1988-1998; age 25-34	2,636 (17%)
1978-1988; age 35-44	2,358 (16%)
1968-1978; age 45-54	2,406 (16%)
1958-1968; age 55-64	2,282 (15%)
1948-1958; age 65-74	2,146 (14%)
1938-1948; age 75-84	1,605 (11%)
1938 or earlier; age 85+	122 (0.8%)
(Missing)	0 (0%)
**Gender**	
Male	7,536 (50%)
Female	7,493 (50%)
Other	27 (0.2%)
(Missing)	12 (<0.1%)
**Religious affiliation**	
Christianity	10,617 (70%)
Islam	462 (3.1%)
Hinduism	16 (0.1%)
Buddhism	41 (0.3%)
Judaism	51 (0.3%)
Sikhism	9 (<0.1%)
Baha'i	3 (<0.1%)
Jainism	0 (0%)
Shinto	1 (<0.1%)
Taoism	0 (0%)
Confucianism	4 (<0.1%)
Primal, Animist, or Folk religion	31 (0.2%)
Spiritism	0 (0%)
Umbanda, Candomble, and other African-derived religions	0 (0%)
Chinese folk/traditional religion	0 (0%)
Some other religion	69 (0.5%)
No religion/Atheist/Agnostic	3,738 (25%)
(Missing)	26 (0.2%)

[1]n (%)

71. Table S71. Childhood predictors regression for Sweden

Variable	Category	Risk-Ratio	RR 95% CI	Global p-value
Relationship with mother	(Ref: Very bad/somewhat bad)			0.252
	Very good/somewhat good	0.90	(0.74,1.08)	
Relationship with father	(Ref: Very bad/somewhat bad)			0.138
	Very good/somewhat good	1.13	(0.96,1.34)	
Parent marital status	(Ref: Parents married)			4.58e-06
	Divorced	1.41	(1.19,1.67)	
	Parents were never married	1.52	(1.26,1.83)	
	One or both parents had died	1.48	(1.01,2.17)	
Subjective financial status of family growing up	(Ref: Got by)			0.033
	Lived comfortably	0.83	(0.72,0.96)	
	Found it difficult	1.07	(0.88,1.31)	
	Found it very difficult	1.17	(0.77,1.79)	
Abuse	(Ref: No)			1.53e-06
	Yes	1.46	(1.25,1.71)	
Outsider growing up	(Ref: No)			0.009
	Yes	1.26	(1.06,1.51)	
Self-rated health growing up	(Ref: Good)			0.744
	Excellent	1.07	(0.89,1.29)	
	Very good	1.03	(0.86,1.23)	
	Fair	1.16	(0.91,1.46)	
	Poor	1.10	(0.80,1.52)	
Immigration status	(Ref: Born in this country)			0.037
	Born in another country	1.25	(1.01,1.56)	
Age 12 religious service attendance	(Ref: Never)			0.047
	At least 1/week	1.32	(1.03,1.69)	
	1-3/month	1.26	(1.01,1.57)	
	< 1/month	1.03	(0.90,1.18)	
Year of birth	(Ref: 1998-2005; age: 18-24)			2.80e-06
	1988-1998; age 25-34	1.30	(1.03,1.64)	
	1978-1988; age 35-44	1.27	(1.00,1.62)	
	1968-1978; age 45-54	1.32	(1.03,1.70)	
	1958-1968; age 55-64	1.53	(1.20,1.95)	
	1948-1957; age 65-74	1.46	(1.11,1.91)	
	1938-1948; age 75-84	0.84	(0.60,1.19)	
	1938 or earlier; 85 or older	0.11	(0.02,0.80)	
Gender	(Ref: Male)			0.751
	Female	1.05	(0.92,1.19)	
	Other	1.13	(0.28,4.49)	
Religious affiliation	(Ref: No religion/Atheist/Agnostic)			2.79e-04
	Islam	2.06	(1.50,2.81)	
	Christianity	0.91	(0.78,1.06)	
	Collapsed affiliations with prevalence<3%	1.46	(0.95,2.24)	
Race/ethnicity	(Ref: Plurality group)			

72. Table S72. Sensitivity to unmeasured confounding of childhood predictors in Sweden

Variable	Category	E-value for Estimate	E-value for 95% CI
Relationship with mother	(Ref: Very bad/somewhat bad)		
	Very good/somewhat good	1.47	1.00
Relationship with father	(Ref: Very bad/somewhat bad)		
	Very good/somewhat good	1.52	1.00
Parent marital status	(Ref: Parents married)		
	Divorced	2.17	1.66
	Parents were never married	2.40	1.83
	One or both parents had died	2.33	1.13
Subjective financial status of family growing up	(Ref: Got by)		
	Lived comfortably	1.71	1.26
	Found it difficult	1.35	1.00
	Found it very difficult	1.62	1.00
Abuse	(Ref: No)		
	Yes	2.29	1.81
Outsider growing up	(Ref: No)		
	Yes	1.84	1.30
Self-rated health growing up	(Ref: Good)		
	Excellent	1.35	1.00
	Very good	1.20	1.00
	Fair	1.58	1.00
	Poor	1.44	1.00
Immigration status	(Ref: Born in this country)		
	Born in another country	1.82	1.12
Age 12 religious service attendance	(Ref: Never)		
	At least 1/week	1.96	1.19
	1-3/month	1.83	1.14
	< 1/month	1.20	1.00
Year of birth	(Ref: 1998-2005; age: 18-24)		
	1988-1998; age 25-34	1.92	1.20
	1978-1988; age 35-44	1.86	1.00
	1968-1978; age 45-54	1.97	1.19
	1958-1968; age 55-64	2.43	1.68
	1948-1957; age 65-74	2.28	1.47
	1938-1948; age 75-84	1.66	1.00
	1938 or earlier; 85 or older	17.14	1.79
Gender	(Ref: Male)		
	Female	1.27	1.00
	Other	1.51	1.00
Religious affiliation	(Ref: No religion/Atheist/Agnostic)		
	Islam	3.53	2.38
	Christianity	1.43	1.00
	Collapsed affiliations with prevalence<3%	2.28	1.00
Race/ethnicity	(Ref: Plurality group)		

73. **Table S73. Nationally representative descriptive statistics for Tanzania: Childhood predictors**

**Characteristic**	**N = 9,075**[1]
**Relationship with mother**	
Very good	7,739 (85%)
Somewhat good	796 (8.8%)
Somewhat bad	84 (0.9%)
Very bad	84 (0.9%)
Does not apply	303 (3.3%)
(Missing)	70 (0.8%)
**Relationship with father**	
Very good	6,831 (75%)
Somewhat good	1,101 (12%)
Somewhat bad	203 (2.2%)
Very bad	247 (2.7%)
Does not apply	550 (6.1%)
(Missing)	142 (1.6%)
**Parent marital status**	
Parents married	6,929 (76%)
Divorced	678 (7.5%)
Parents were never married	751 (8.3%)
One or both parents had died	313 (3.4%)
(Missing)	404 (4.4%)
**Subjective financial status of family growing up**	
Lived comfortably	2,611 (29%)
Got by	2,909 (32%)
Found it difficult	2,679 (30%)
Found it very difficult	814 (9.0%)
(Missing)	61 (0.7%)
**Abuse**	
Yes	716 (7.9%)
No	8,328 (92%)
(Missing)	32 (0.3%)
**Outsider growing up**	
Yes	734 (8.1%)
No	8,320 (92%)
(Missing)	22 (0.2%)
**Self-rated health growing up**	
Excellent	2,406 (27%)
Very good	2,036 (22%)
Good	2,946 (32%)
Fair	1,177 (13%)
Poor	456 (5.0%)
(Missing)	54 (0.6%)
**Immigration status**	
Born in this country	9,048 (100%)
Born in another country	25 (0.3%)
(Missing)	1 (<0.1%)
**Age 12 religious service attendance**	
At least 1/week	5,580 (61%)
1-3/month	2,383 (26%)
<1/month	333 (3.7%)
Never	595 (6.6%)
(Missing)	184 (2.0%)

74. Table S74. Nationally representative descriptive statistics for Tanzania: Demographic variables

Characteristic	N = 9,075[1]
**Year of birth**	
1998-2005; age 18-24	2,284 (25%)
1988-1998; age 25-34	2,515 (28%)
1978-1988; age 35-44	1,719 (19%)
1968-1978; age 45-54	1,206 (13%)
1958-1968; age 55-64	700 (7.7%)
1948-1958; age 65-74	460 (5.1%)
1938-1948; age 75-84	153 (1.7%)
1938 or earlier; age 85+	35 (0.4%)
(Missing)	2 (<0.1%)
**Gender**	
Male	4,299 (47%)
Female	4,776 (53%)
Other	0 (0%)
(Missing)	0 (0%)
**Religious affiliation**	
Christianity	5,651 (62%)
Islam	3,060 (34%)
Hinduism	0 (0%)
Buddhism	0 (0%)
Judaism	0 (0%)
Sikhism	0 (0%)
Baha'i	1 (<0.1%)
Jainism	0 (0%)
Shinto	0 (0%)
Taoism	0 (0%)
Confucianism	0 (0%)
Primal, Animist, or Folk religion	11 (0.1%)
Spiritism	0 (0%)
Umbanda, Candomble, and other African-derived religions	0 (0%)
Chinese folk/traditional religion	0 (0%)
Some other religion	0 (0%)
No religion/Atheist/Agnostic	345 (3.8%)
(Missing)	7 (<0.1%)
**Race/Ethnicity**	
African	9,060 (100%)
Arab	11 (0.1%)
Indian	3 (<0.1%)
(Missing)	2 (<0.1%)

[1]n (%)

75. Table S75. Childhood predictors regression for Tanzania

Variable	Category	Risk-Ratio	RR 95% CI	Global p-value
Relationship with mother	(Ref: Very bad/somewhat bad)			0.516
	Very good/somewhat good	0.79	(0.37,1.70)	
Relationship with father	(Ref: Very bad/somewhat bad)			0.529
	Very good/somewhat good	0.86	(0.50,1.47)	
Parent marital status	(Ref: Parents married)			0.397
	Divorced	0.93	(0.57,1.53)	
	Parents were never married	1.32	(0.91,1.90)	
	One or both parents had died	0.88	(0.47,1.67)	
Subjective financial status of family growing up	(Ref: Got by)			0.900
	Lived comfortably	1.05	(0.77,1.41)	
	Found it difficult	0.93	(0.67,1.30)	
	Found it very difficult	0.92	(0.53,1.59)	
Abuse	(Ref: No)			0.015
	Yes	1.53	(1.09,2.16)	
Outsider growing up	(Ref: No)			0.853
	Yes	1.04	(0.66,1.62)	
Self-rated health growing up	(Ref: Good)			0.331
	Excellent	0.89	(0.65,1.23)	
	Very good	0.84	(0.59,1.20)	
	Fair	1.16	(0.80,1.68)	
	Poor	0.62	(0.32,1.20)	
Immigration status	(Ref: Born in this country)			0.594
	Born in another country	0.56	(0.07,4.63)	
Age 12 religious service attendance	(Ref: Never)			0.340
	At least 1/week	1.31	(0.66,2.61)	
	1-3/month	1.61	(0.78,3.29)	
	< 1/month	1.39	(0.59,3.26)	
Year of birth	(Ref: 1998-2005; age: 18-24)			0.000
	1988-1998; age 25-34	2.16	(1.19,3.91)	
	1978-1988; age 35-44	2.92	(1.62,5.28)	
	1968-1978; age 45-54	3.31	(1.83,6.00)	
	1958-1968; age 55-64	3.68	(2.01,6.74)	
	1948-1957; age 65-74	2.39	(1.13,5.09)	
	1938-1948; age 75-84	1.38	(0.44,4.33)	
	1938 or earlier; 85 or older	0.00	(0.00,0.00)	
Gender	(Ref: Male)			0.000
	Female	0.05	(0.03,0.08)	
Religious affiliation	(Ref: No religion/Atheist/Agnostic)			1.82e-10
	Islam	1.28	(0.56,2.92)	
	Christianity	0.79	(0.35,1.79)	
	Collapsed affiliations with prevalence<3%	0.00	(0.00,0.00)	
Race/ethnicity	(Ref: Plurality group)			1.50e-04
	Non-plurality groups	0.00	(0.00,0.00)	

76. **Table S76. Sensitivity to unmeasured confounding of childhood predictors in Tanzania**

Variable	Category	E-value for Estimate	E-value for 95% CI
Relationship with mother	(Ref: Very bad/somewhat bad)		
	Very good/somewhat good	1.84	1.00
Relationship with father	(Ref: Very bad/somewhat bad)		
	Very good/somewhat good	1.60	1.00
Parent marital status	(Ref: Parents married)		
	Divorced	1.35	1.00
	Parents were never married	1.96	1.00
	One or both parents had died	1.51	1.00
Subjective financial status of family growing up	(Ref: Got by)		
	Lived comfortably	1.26	1.00
	Found it difficult	1.35	1.00
	Found it very difficult	1.38	1.00
Abuse	(Ref: No)		
	Yes	2.44	1.40
Outsider growing up	(Ref: No)		
	Yes	1.23	1.00
Self-rated health growing up	(Ref: Good)		
	Excellent	1.49	1.00
	Very good	1.67	1.00
	Fair	1.59	1.00
	Poor	2.63	1.00
Immigration status	(Ref: Born in this country)		
	Born in another country	2.95	1.00
Age 12 religious service attendance	(Ref: Never)		
	At least 1/week	1.96	1.00
	1-3/month	2.60	1.00
	< 1/month	2.13	1.00
Year of birth	(Ref: 1998-2005; age: 18-24)		
	1988-1998; age 25-34	3.73	1.66
	1978-1988; age 35-44	5.29	2.61
	1968-1978; age 45-54	6.09	3.07
	1958-1968; age 55-64	6.82	3.43
	1948-1957; age 65-74	4.22	1.50
	1938-1948; age 75-84	2.10	1.00
	1938 or earlier; 85 or older	793431.16	355452.81
Gender	(Ref: Male)		
	Female	41.37	23.83
Religious affiliation	(Ref: No religion/Atheist/Agnostic)		
	Islam	1.87	1.00
	Christianity	1.85	1.00
	Collapsed affiliations with prevalence<3%	4636989.64	1770486.00
Race/ethnicity	(Ref: Plurality group)		
	Non-plurality groups	2283277.62	1062284.55

77. **Table S77. Nationally representative descriptive statistics for Türkiye: Childhood predictors**

Characteristic	N = 1,473[1]
**Relationship with mother**	
Very good	970 (66%)
Somewhat good	401 (27%)
Somewhat bad	48 (3.2%)
Very bad	26 (1.8%)
Does not apply	21 (1.4%)
(Missing)	7 (0.5%)
**Relationship with father**	
Very good	795 (54%)
Somewhat good	425 (29%)
Somewhat bad	73 (5.0%)
Very bad	95 (6.5%)
Does not apply	60 (4.1%)
(Missing)	25 (1.7%)
**Parent marital status**	
Parents married	1,325 (90%)
Divorced	57 (3.9%)
Parents were never married	7 (0.5%)
One or both parents had died	61 (4.1%)
(Missing)	23 (1.5%)
**Subjective financial status of family growing up**	
Lived comfortably	498 (34%)
Got by	647 (44%)
Found it difficult	218 (15%)
Found it very difficult	108 (7.3%)
(Missing)	2 (0.1%)
**Abuse**	
Yes	158 (11%)
No	1,290 (88%)
(Missing)	25 (1.7%)
**Outsider growing up**	
Yes	157 (11%)
No	1,306 (89%)
(Missing)	9 (0.6%)
**Self-rated health growing up**	
Excellent	377 (26%)
Very good	410 (28%)
Good	419 (28%)
Fair	220 (15%)
Poor	47 (3.2%)
(Missing)	0 (<0.1%)
**Immigration status**	
Born in this country	1,415 (96%)
Born in another country	58 (4.0%)
(Missing)	0 (0%)
**Age 12 religious service attendance**	
At least 1/week	609 (41%)
1-3/month	238 (16%)
<1/month	225 (15%)
Never	383 (26%)
(Missing)	18 1.2%)

78. Table S78. Nationally representative descriptive statistics for Türkiye: Demographic variables

Characteristic	N = 1,473[1]
**Year of birth**	
1998-2005; age 18-24	222 (15%)
1988-1998; age 25-34	312 (21%)
1978-1988; age 35-44	318 (22%)
1968-1978; age 45-54	262 (18%)
1958-1968; age 55-64	200 (14%)
1948-1958; age 65-74	120 (8.2%)
1938-1948; age 75-84	35 (2.4%)
1938 or earlier; age 85+	3 (0.2%)
(Missing)	0 (0%)
**Gender**	
Male	754 (51%)
Female	719 (49%)
Other	0 (0%)
(Missing)	0 (0%)
**Religious affiliation**	
Christianity	1 (<0.1%)
Islam	1,439 (98%)
Hinduism	0 (0%)
Buddhism	0 (0%)
Judaism	1 (<0.1%)
Sikhism	0 (0%)
Baha'i	0 (0%)
Jainism	0 (0%)
Shinto	0 (0%)
Taoism	0 (0%)
Confucianism	0 (0%)
Primal, Animist, or Folk religion	0 (0%)
Spiritism	0 (0%)
Umbanda, Candomble, and other African-derived religions	0 (0%)
Chinese folk/traditional religion	0 (0%)
Some other religion	0 (0%)
No religion/Atheist/Agnostic	13 (0.9%)
(Missing)	19 (1.3%)
**Race/Ethnicity**	
Albanian	8 (0.5%)
Arab	51 (3.5%)
Armenian	1 (<0.1%)
Azeri	9 (0.6%)
Bosnian	5 (0.3%)
Circassian	19 (1.3%)
Georgian	4 (0.3%)
Greek	1 (<0.1%)
Kurdish/Zaza	252 (17%)
Laz	25 (1.7%)
Other	58 (3.9%)
Turkish	1,030 (70%)
Uyghur	1 (<0.1%)
(Missing)	9 (0.6%)

[1]n (%)

79. Table S79. Childhood predictors regression for Türkiye

Variable	Category	Risk-Ratio	RR 95% CI	Global p-value
Relationship with mother	(Ref: Very bad/somewhat bad)			0.833
	Very good/somewhat good	1.02	(0.81,1.30)	
Relationship with father	(Ref: Very bad/somewhat bad)			0.083
	Very good/somewhat good	0.87	(0.75,1.02)	
Parent marital status	(Ref: Parents married)			0.002
	Divorced	1.41	(1.18,1.69)	
	Parents were never married	1.21	(0.72,2.03)	
	One or both parents had died	1.05	(0.76,1.45)	
Subjective financial status of family growing up	(Ref: Got by)			0.475
	Lived comfortably	1.03	(0.90,1.17)	
	Found it difficult	0.89	(0.76,1.06)	
	Found it very difficult	0.99	(0.79,1.24)	
Abuse	(Ref: No)			0.318
	Yes	1.09	(0.92,1.30)	
Outsider growing up	(Ref: No)			0.113
	Yes	1.14	(0.97,1.34)	
Self-rated health growing up	(Ref: Good)			0.530
	Excellent	1.03	(0.89,1.19)	
	Very good	1.00	(0.85,1.18)	
	Fair	1.05	(0.87,1.27)	
	Poor	1.27	(0.96,1.68)	
Immigration status	(Ref: Born in this country)			0.482
	Born in another country	0.90	(0.66,1.21)	
Age 12 religious service attendance	(Ref: Never)			0.069
	At least 1/week	1.20	(1.02,1.40)	
	1-3/month	1.18	(0.97,1.42)	
	< 1/month	1.02	(0.83,1.25)	
Year of birth	(Ref: 1998-2005; age: 18-24)			8.41e-04
	1988-1998; age 25-34	1.16	(0.98,1.36)	
	1978-1988; age 35-44	1.13	(0.95,1.33)	
	1968-1978; age 45-54	1.18	(0.99,1.41)	
	1958-1968; age 55-64	1.08	(0.86,1.34)	
	1948-1957; age 65-74	0.83	(0.57,1.20)	
	1938-1948; age 75-84	0.53	(0.21,1.34)	
	1938 or earlier; 85 or older	1.65	(1.31,2.06)	
Gender	(Ref: Male)			1.01e-08
	Female	0.69	(0.61,0.79)	
Religious affiliation	(Ref: Islam)			0.228
	Collapsed affiliations with prevalence<3%	1.21	(0.89,1.66)	
Race/ethnicity	(Ref: Plurality group)			0.376
	Non-plurality groups	1.06	(0.93,1.20)	

80. Table S80. Sensitivity to unmeasured confounding of childhood predictors in Türkiye

Variable	Category	E-value for Estimate	E-value for 95% CI
Relationship with mother	(Ref: Very bad/somewhat bad)		
	Very good/somewhat good	1.18	1.00
Relationship with father	(Ref: Very bad/somewhat bad)		
	Very good/somewhat good	1.55	1.00
Parent marital status	(Ref: Parents married)		
	Divorced	2.17	1.63
	Parents were never married	1.71	1.00
	One or both parents had died	1.28	1.00
Subjective financial status of family growing up	(Ref: Got by)		
	Lived comfortably	1.20	1.00
	Found it difficult	1.48	1.00
	Found it very difficult	1.09	1.00
Abuse	(Ref: No)		
	Yes	1.40	1.00
Outsider growing up	(Ref: No)		
	Yes	1.53	1.00
Self-rated health growing up	(Ref: Good)		
	Excellent	1.19	1.00
	Very good	1.04	1.00
	Fair	1.29	1.00
	Poor	1.86	1.00
Immigration status	(Ref: Born in this country)		
	Born in another country	1.47	1.00
Age 12 religious service attendance	(Ref: Never)		
	At least 1/week	1.68	1.18
	1-3/month	1.64	1.00
	< 1/month	1.15	1.00
Year of birth	(Ref: 1998-2005; age: 18-24)		
	1988-1998; age 25-34	1.58	1.00
	1978-1988; age 35-44	1.51	1.00
	1968-1978; age 45-54	1.65	1.00
	1958-1968; age 55-64	1.36	1.00
	1948-1957; age 65-74	1.70	1.00
	1938-1948; age 75-84	3.18	1.00
	1938 or earlier; 85 or older	2.68	1.95
Gender	(Ref: Male)		
	Female	2.24	1.86
Religious affiliation	(Ref: Islam)		
	Collapsed affiliations with prevalence<3%	1.72	1.00
Race/ethnicity	(Ref: Plurality group)		
	Non-plurality groups	1.30	1.00

81. Table S81. Nationally representative descriptive statistics for United Kingdom: Childhood predictors

Characteristic	N = 5,368[1]
**Relationship with mother**	
Very good	3,435 (64%)
Somewhat good	1,338 (25%)
Somewhat bad	325 (6.1%)
Very bad	150 (2.8%)
Does not apply	92 (1.7%)
(Missing)	27 (0.5%)
**Relationship with father**	
Very good	2,907 (54%)
Somewhat good	1,383 (26%)
Somewhat bad	407 (7.6%)
Very bad	321 (6.0%)
Does not apply	321 (6.0%)
(Missing)	29 (0.5%)
**Parent marital status**	
Parents married	4,343 (81%)
Divorced	481 (9.0%)
Parents were never married	315 (5.9%)
One or both parents had died	154 (2.9%)
(Missing)	75 (1.4%)
**Subjective financial status of family growing up**	
Lived comfortably	2,552 (48%)
Got by	1,933 (36%)
Found it difficult	632 (12%)
Found it very difficult	230 (4.3%)
(Missing)	22 (0.4%)
**Abuse**	
Yes	864 (16%)
No	4,455 (83%)
(Missing)	49 (0.9%)
**Outsider growing up**	
Yes	1,017 (19%)
No	4,308 (80%)
(Missing)	43 (0.8%)
**Self-rated health growing up**	
Excellent	2,154 (40%)
Very good	1,736 (32%)
Good	995 (19%)
Fair	332 (6.2%)
Poor	130 (2.4%)
(Missing)	20 (0.4%)
**Immigration status**	
Born in this country	4,659 (87%)
Born in another country	682 (13%)
(Missing)	27 (0.5%)
**Age 12 religious service attendance**	
At least 1/week	1,732 (32%)
1-3/month	733 (14%)
<1/month	903 (17%)
Never	1,972 (37%)
(Missing)	28 (0.5%)

82. **Table S82. Nationally representative descriptive statistics for United Kingdom: Demographic variables**

Characteristic	N = 5,368[1]
**Year of birth**	
1998-2005; age 18-24	490 (9.1%)
1988-1998; age 25-34	911 (17%)
1978-1988; age 35-44	901 (17%)
1968-1978; age 45-54	901 (17%)
1958-1968; age 55-64	864 (16%)
1948-1958; age 65-74	838 (16%)
1938-1948; age 75-84	417 (7.8%)
1938 or earlier; age 85+	46 (0.9%)
(Missing)	1 (<0.1%)
**Gender**	
Male	2,557 (48%)
Female	2,789 (52%)
Other	14 (0.3%)
(Missing)	9 (0.2%)
**Religious affiliation**	
Christianity	3,461 (64%)
Islam	230 (4.3%)
Hinduism	88 (1.6%)
Buddhism	15 (0.3%)
Judaism	59 (1.1%)
Sikhism	30 (0.6%)
Baha'i	5 (<0.1%)
Jainism	0 (<0.1%)
Shinto	0 (0%)
Taoism	2 (<0.1%)
Confucianism	3 (<0.1%)
Primal, Animist, or Folk religion	22 (0.4%)
Spiritism	0 (0%)
Umbanda, Candomble, and other African-derived religions	0 (0%)
Chinese folk/traditional religion	0 (0%)
Some other religion	24 (0.5%)
No religion/Atheist/Agnostic	1,409 (26%)
(Missing)	21 (0.4%)
**Race/Ethnicity**	
Asian	426 (7.9%)
Black	152 (2.8%)
Other	96 (1.8%)
White	4,647 (87%)
(Missing)	47 (0.9%)

[1]n (%)

83. Table S83. Childhood predictors regression for United Kingdom

Variable	Category	Risk-Ratio	RR 95% CI	Global p-value
Relationship with mother	(Ref: Very bad/somewhat bad)			0.148
	Very good/somewhat good	0.82	(0.62,1.08)	
Relationship with father	(Ref: Very bad/somewhat bad)			0.446
	Very good/somewhat good	1.10	(0.86,1.40)	
Parent marital status	(Ref: Parents married)			0.023
	Divorced	1.29	(0.97,1.72)	
	Parents were never married	1.48	(1.09,2.01)	
	One or both parents had died	1.43	(0.93,2.18)	
Subjective financial status of family growing up	(Ref: Got by)			0.954
	Lived comfortably	1.01	(0.84,1.21)	
	Found it difficult	0.97	(0.73,1.29)	
	Found it very difficult	0.91	(0.61,1.35)	
Abuse	(Ref: No)			5.43e-05
	Yes	1.47	(1.22,1.78)	
Outsider growing up	(Ref: No)			3.46e-06
	Yes	1.55	(1.28,1.87)	
Self-rated health growing up	(Ref: Good)			0.445
	Excellent	0.99	(0.77,1.27)	
	Very good	1.10	(0.86,1.40)	
	Fair	0.84	(0.58,1.22)	
	Poor	0.77	(0.43,1.39)	
Immigration status	(Ref: Born in this country)			0.256
	Born in another country	0.85	(0.64,1.13)	
Age 12 religious service attendance	(Ref: Never)			0.906
	At least 1/week	1.03	(0.81,1.31)	
	1-3/month	1.10	(0.84,1.43)	
	< 1/month	1.01	(0.78,1.30)	
Year of birth	(Ref: 1998-2005; age: 18-24)			9.12e-09
	1988-1998; age 25-34	3.12	(1.87,5.19)	
	1978-1988; age 35-44	2.69	(1.61,4.51)	
	1968-1978; age 45-54	2.38	(1.40,4.04)	
	1958-1968; age 55-64	2.18	(1.28,3.72)	
	1948-1957; age 65-74	1.95	(1.11,3.40)	
	1938-1948; age 75-84	0.89	(0.41,1.91)	
	1938 or earlier; 85 or older	0.26	(0.05,1.26)	
Gender	(Ref: Male)			3.10e-05
	Female	0.68	(0.58,0.81)	
	Other	0.61	(0.18,2.04)	
Religious affiliation	(Ref: No religion/Atheist/Agnostic)			0.311
	Islam	1.48	(0.92,2.36)	
	Christianity	1.15	(0.92,1.44)	
	Collapsed affiliations with prevalence<3%	1.34	(0.86,2.10)	
Race/ethnicity	(Ref: Plurality group)			0.255
	Non-plurality groups	0.82	(0.58,1.16)	

84. Table S84. Sensitivity to unmeasured confounding of childhood predictors in United Kingdom

Variable	Category	E-value for Estimate	E-value for 95% CI
Relationship with mother	(Ref: Very bad/somewhat bad)		
	Very good/somewhat good	1.75	1.00
Relationship with father	(Ref: Very bad/somewhat bad)		
	Very good/somewhat good	1.42	1.00
Parent marital status	(Ref: Parents married)		
	Divorced	1.89	1.00
	Parents were never married	2.33	1.41
	One or both parents had died	2.20	1.00
Subjective financial status of family growing up	(Ref: Got by)		
	Lived comfortably	1.10	1.00
	Found it difficult	1.21	1.00
	Found it very difficult	1.44	1.00
Abuse	(Ref: No)		
	Yes	2.31	1.73
Outsider growing up	(Ref: No)		
	Yes	2.47	1.88
Self-rated health growing up	(Ref: Good)		
	Excellent	1.10	1.00
	Very good	1.43	1.00
	Fair	1.66	1.00
	Poor	1.91	1.00
Immigration status	(Ref: Born in this country)		
	Born in another country	1.63	1.00
Age 12 religious service attendance	(Ref: Never)		
	At least 1/week	1.22	1.00
	1-3/month	1.42	1.00
	< 1/month	1.10	1.00
Year of birth	(Ref: 1998-2005; age: 18-24)		
	1988-1998; age 25-34	5.69	3.15
	1978-1988; age 35-44	4.83	2.59
	1968-1978; age 45-54	4.19	2.15
	1958-1968; age 55-64	3.79	1.88
	1948-1957; age 65-74	3.30	1.47
	1938-1948; age 75-84	1.50	1.00
	1938 or earlier; 85 or older	7.07	1.00
Gender	(Ref: Male)		
	Female	2.28	1.78
	Other	2.67	1.00
Religious affiliation	(Ref: No religion/Atheist/Agnostic)		
	Islam	2.31	1.00
	Christianity	1.57	1.00
	Collapsed affiliations with prevalence<3%	2.01	1.00
Race/ethnicity	(Ref: Plurality group)		
	Non-plurality groups	1.75	1.00

85. Table S85. Nationally representative descriptive statistics for United States: Childhood predictors

Characteristic	N = 38,312[1]
**Relationship with mother**	
Very good	20,590 (54%)
Somewhat good	11,525 (30%)
Somewhat bad	3,523 (9.2%)
Very bad	1,874 (4.9%)
Does not apply	694 (1.8%)
(Missing)	106 (0.3%)
**Relationship with father**	
Very good	15,313 (40%)
Somewhat good	12,665 (33%)
Somewhat bad	4,879 (13%)
Very bad	2,604 (6.8%)
Does not apply	2,811 (7.3%)
(Missing)	38 (0.1%)
**Parent marital status**	
Parents married	27,415 (72%)
Divorced	6,325 (17%)
Parents were never married	3,048 (8.0%)
One or both parents had died	1,024 (2.7%)
(Missing)	500 (1.3%)
**Subjective financial status of family growing up**	
Lived comfortably	15,116 (39%)
Got by	15,682 (41%)
Found it difficult	5,152 (13%)
Found it very difficult	2,342 (6.1%)
(Missing)	19 (<0.1%)
**Abuse**	
Yes	10,026 (26%)
No	28,045 (73%)
(Missing)	242 (0.6%)
**Outsider growing up**	
Yes	10,185 (27%)
No	27,714 (72%)
(Missing)	413 (1.1%)
**Self-rated health growing up**	
Excellent	16,866 (44%)
Very good	12,108 (32%)
Good	6,444 (17%)
Fair	2,303 (6.0%)
Poor	520 (1.4%)
(Missing)	71 (0.2%)
**Immigration status**	
Born in this country	34,865 (91%)
Born in another country	3,020 (7.9%)
(Missing)	427 (1.1%)
**Age 12 religious service attendance**	
At least 1/week	18,609 (49%)
1-3/month	6,644 (17%)
<1/month	5,829 (15%)
Never	7,085 (18%)
(Missing)	145 (0.4%)

86. **Table S86. Nationally representative descriptive statistics for United States: Demographic variables**

Characteristic	N = 38,312[1]
**Year of birth**	
1998-2005; age 18-24	2,682 (7.0%)
1988-1998; age 25-34	7,802 (20%)
1978-1988; age 35-44	5,850 (15%)
1968-1978; age 45-54	5,957 (16%)
1958-1968; age 55-64	7,518 (20%)
1948-1958; age 65-74	5,459 (14%)
1938-1948; age 75-84	2,364 (6.2%)
1938 or earlier; age 85+	679 (1.8%)
(Missing)	0 (0%)
**Gender**	
Male	18,222 (48%)
Female	19,562 (51%)
Other	392 (1.0%)
(Missing)	136 (0.4%)
**Religious affiliation**	
Christianity	30,444 (79%)
Islam	220 (0.6%)
Hinduism	203 (0.5%)
Buddhism	172 (0.4%)
Judaism	787 (2.1%)
Sikhism	47 (0.1%)
Baha'i	4 (<0.1%)
Jainism	18 (<0.1%)
Shinto	6 (<0.1%)
Taoism	17 (<0.1%)
Confucianism	8 (<0.1%)
Primal, Animist, or Folk religion	67 (0.2%)
Spiritism	0 (0%)
Umbanda, Candomble, and other African-derived religions	0 (0%)
Chinese folk/traditional religion	0 (0%)
Some other religion	359 (0.9%)
No religion/Atheist/Agnostic	5,845 (15%)
(Missing)	115 (0.3%)
**Race/Ethnicity**	
Asian	2,466 (6.4%)
Black	4,501 (12%)
Hispanic	6,724 (18%)
Other	997 (2.6%)
White	23,605 (62%)
(Missing)	20 (<0.1%)

[1]n (%)

87. Table S87. Childhood predictors regression for United States

Variable	Category	Risk-Ratio	RR 95% CI	Global p-value
Relationship with mother	(Ref: Very bad/somewhat bad)			0.692
	Very good/somewhat good	0.96	(0.77,1.19)	
Relationship with father	(Ref: Very bad/somewhat bad)			0.176
	Very good/somewhat good	1.15	(0.94,1.40)	
Parent marital status	(Ref: Parents married)			2.10e-04
	Divorced	1.27	(1.04,1.54)	
	Parents were never married	2.02	(1.44,2.81)	
	One or both parents had died	1.36	(0.82,2.27)	
Subjective financial status of family growing up	(Ref: Got by)			0.867
	Lived comfortably	0.94	(0.80,1.11)	
	Found it difficult	0.94	(0.74,1.20)	
	Found it very difficult	1.01	(0.75,1.37)	
Abuse	(Ref: No)			8.21e-06
	Yes	1.49	(1.25,1.77)	
Outsider growing up	(Ref: No)			1.70e-05
	Yes	1.47	(1.23,1.75)	
Self-rated health growing up	(Ref: Good)			0.397
	Excellent	0.86	(0.69,1.07)	
	Very good	0.84	(0.68,1.05)	
	Fair	0.79	(0.54,1.18)	
	Poor	1.16	(0.64,2.12)	
Immigration status	(Ref: Born in this country)			9.79e-11
	Born in another country	0.31	(0.22,0.45)	
Age 12 religious service attendance	(Ref: Never)			0.008
	At least 1/week	0.72	(0.56,0.93)	
	1-3/month	0.89	(0.67,1.18)	
	< 1/month	0.90	(0.67,1.23)	
Year of birth	(Ref: 1998-2005; age: 18-24)			0.000
	1988-1998; age 25-34	1.82	(0.96,3.43)	
	1978-1988; age 35-44	2.28	(1.24,4.20)	
	1968-1978; age 45-54	1.96	(1.06,3.62)	
	1958-1968; age 55-64	2.48	(1.35,4.54)	
	1948-1957; age 65-74	1.96	(1.07,3.59)	
	1938-1948; age 75-84	0.99	(0.53,1.85)	
	1938 or earlier; 85 or older	0.25	(0.09,0.66)	
Gender	(Ref: Male)			0.406
	Female	0.91	(0.78,1.05)	
	Other	0.73	(0.20,2.60)	
Religious affiliation	(Ref: No religion/Atheist/Agnostic)			0.444
	Christianity	1.16	(0.88,1.54)	
	Collapsed affiliations with prevalence<3%	0.98	(0.62,1.56)	
Race/ethnicity	(Ref: Plurality group)			0.842
	Non-plurality groups	0.99	(0.83,1.17)	

88. **Table S88. Sensitivity to unmeasured confounding of childhood predictors in United States**

Variable	Category	E-value for Estimate	E-value for 95% CI
Relationship with mother	(Ref: Very bad/somewhat bad)		
	Very good/somewhat good	1.25	1.00
Relationship with father	(Ref: Very bad/somewhat bad)		
	Very good/somewhat good	1.55	1.00
Parent marital status	(Ref: Parents married)		
	Divorced	1.85	1.25
	Parents were never married	3.45	2.25
	One or both parents had died	2.06	1.00
Subjective financial status of family growing up	(Ref: Got by)		
	Lived comfortably	1.31	1.00
	Found it difficult	1.32	1.00
	Found it very difficult	1.13	1.00
Abuse	(Ref: No)		
	Yes	2.34	1.80
Outsider growing up	(Ref: No)		
	Yes	2.30	1.77
Self-rated health growing up	(Ref: Good)		
	Excellent	1.60	1.00
	Very good	1.66	1.00
	Fair	1.83	1.00
	Poor	1.59	1.00
Immigration status	(Ref: Born in this country)		
	Born in another country	5.85	3.84
Age 12 religious service attendance	(Ref: Never)		
	At least 1/week	2.12	1.36
	1-3/month	1.50	1.00
	< 1/month	1.45	1.00
Year of birth	(Ref: 1998-2005; age: 18-24)		
	1988-1998; age 25-34	3.04	1.00
	1978-1988; age 35-44	3.99	1.78
	1968-1978; age 45-54	3.33	1.32
	1958-1968; age 55-64	4.40	2.05
	1948-1957; age 65-74	3.33	1.35
	1938-1948; age 75-84	1.14	1.00
	1938 or earlier; 85 or older	7.55	2.38
Gender	(Ref: Male)		
	Female	1.44	1.00
	Other	2.10	1.00
Religious affiliation	(Ref: No religion/Atheist/Agnostic)		
	Christianity	1.60	1.00
	Collapsed affiliations with prevalence<3%	1.16	1.00
Race/ethnicity	(Ref: Plurality group)		
	Non-plurality groups	1.13	1.00

89. Table S89. Population weighted meta-analysis of regression results (binary).

Variable	Category	RR	95% CI
Relationship with mother	(Ref: Very bad/somewhat bad)		
	Very good/somewhat good	0.99	(0.82,1.21)
Relationship with father	(Ref: Very bad/somewhat bad)		
	Very good/somewhat good	0.82	(0.69,0.98)
Parent marital status	(Ref: Parents married)		
	Divorced	1.11	(0.93,1.33)
	Single, never married	1.11	(0.98,1.26)
	One or both parents had died	1.08	(0.96,1.22)
Subjective financial status of family growing up	(Ref: Got by)		
	Lived comfortably	1.00	(0.92,1.08)
	Found it difficult	0.96	(0.87,1.06)
	Found it very difficult	1.12	(1.01,1.25)
Abuse	(Ref: No)		
	Yes	1.34	(1.23,1.46)
Outsider growing up	(Ref: No)		
	Yes	1.18	(1.08,1.28)
Self-rated health growing up	(Ref: Good)		
	Excellent	1.01	(0.92,1.11)
	Very good	1.01	(0.92,1.10)
	Fair	0.99	(0.89,1.09)
	Poor	0.80	(0.65,1.00)
Immigration status	(Ref: Born in this country)		
	Born in another country	1.17	(0.96,1.42)
Age 12 religious service attendance	(Ref: Never)		
	At least 1/week	0.96	(0.85,1.08)
	1-3/month	1.12	(0.98,1.27)
	< 1/month	1.05	(0.92,1.20)
Year of birth	(Ref: 1998-2005; age 18-24)		
	1988-1998; age 25-34	1.39	(1.23,1.58)
	1978-1988; age 35-44	1.58	(1.39,1.79)
	1968-1978; age 45-54	1.63	(1.43,1.86)
	1958-1968; age 55-64	1.63	(1.40,1.89)
	1948-1957; age 65-74	1.15	(0.93,1.41)
	1938-1948; age 75-84	0.31	(0.22,0.43)
	1938 or earlier; 85 or older	0.01	(0.00,1.36)
Gender	(Ref: Male)		
	Female	0.18	(0.15,0.21)
	Other	0.24	(0.15,0.39)

90. Table S90. Population weighted meta-analysis of E-values (binary).

Variable	Category	E-value	E-value.limit
Relationship with mother	(Ref: Very bad/somewhat bad)		
	Very good/somewhat good	1.08	1.00
Relationship with father	(Ref: Very bad/somewhat bad)		
	Very good/somewhat good	1.72	1.15
Parent marital status	(Ref: Parents married)		
	Divorced	1.47	1.00
	Single, never married	1.46	1.00
	One or both parents had died	1.38	1.00
Subjective financial status of family growing up	(Ref: Got by)		
	Lived comfortably	1.07	1.00
	Found it difficult	1.25	1.00
	Found it very difficult	1.50	1.12
Abuse	(Ref: No)		
	Yes	2.01	1.76
Outsider growing up	(Ref: No)		
	Yes	1.63	1.38
Self-rated health growing up	(Ref: Good)		
	Excellent	1.12	1.00
	Very good	1.08	1.00
	Fair	1.13	1.00
	Poor	1.80	1.05
Immigration status	(Ref: Born in this country)		
	Born in another country	1.61	1.00
Age 12 religious service attendance	(Ref: Never)		
	At least 1/week	1.25	1.00
	1-3/month	1.48	1.00
	< 1/month	1.28	1.00
Year of birth	(Ref: 1998-2005; age 18-24)		
	1988-1998; age 25-34	2.13	1.75
	1978-1988; age 35-44	2.54	2.13
	1968-1978; age 45-54	2.64	2.22
	1958-1968; age 55-64	2.64	2.15
	1948-1957; age 65-74	1.55	1.00
	1938-1948; age 75-84	5.88	4.03
	1938 or earlier; 85 or older	192.43	1.00
Gender	(Ref: Male)		
	Female	10.63	9.10
	Other	7.89	4.62

91. **Table S91. Childhood predictors regression for Argentina**

Variable	Category	Est	95% CI	SE	Global p-value
Relationship with mother	(Ref: Very bad/somewhat bad)				0.852
	Very good/somewhat good	0.02	(-1.15, 1.19)	0.59	
Relationship with father	(Ref: Very bad/somewhat bad)				0.720
	Very good/somewhat good	-0.15	(-1.06, 0.75)	0.46	
Parent marital status	(Ref: Parents married)				0.461
	Divorced	0.84	(-0.32, 2.00)	0.59	
	Parents were never married	0.21	(-0.64, 1.06)	0.43	
	One or both parents had died	0.00	(-1.65, 1.66)	0.84	
Subjective financial status of family growing up	(Ref: Got by)				0.253
	Lived comfortably	-0.11	(-0.75, 0.54)	0.33	
	Found it difficult	0.04	(-0.69, 0.76)	0.37	
	Found it very difficult	1.34	(-0.09, 2.78)	0.73	
Abuse	(Ref: No)				0.059
	Yes	0.79	(-0.06, 1.64)	0.43	
Outsider growing up	(Ref: No)				0.069
	Yes	0.82	(-0.09, 1.73)	0.46	
Self-rated health growing up	(Ref: Good)				0.020
	Excellent	0.65	(-0.09, 1.39)	0.38	
	Very good	-0.37	(-1.08, 0.33)	0.36	
	Fair	0.22	(-1.21, 1.64)	0.73	
	Poor	1.88	(-1.37, 5.13)	1.66	
Immigration status	(Ref: Born in this country)				5.24e-04
	Born in another country	-1.56	(-2.45, -0.66)	0.46	
Age 12 religious service attendance	(Ref: Never)				0.359
	At least 1/week	0.03	(-0.83, 0.88)	0.43	
	1-3/month	-0.49	(-1.32, 0.33)	0.42	
	< 1/month	-0.44	(-1.26, 0.39)	0.42	
Year of birth	(Ref: 1998-2005; age: 18-24)				4.73e-10
	1988-1998; age 25-34	1.65	(0.71, 2.60)	0.48	
	1978-1988; age 35-44	2.43	(1.43, 3.42)	0.51	
	1968-1978; age 45-54	1.56	(0.62, 2.49)	0.48	
	1958-1968; age 55-64	1.75	(0.67, 2.82)	0.55	
	1948-1957; age 65-74	1.43	(0.19, 2.66)	0.63	
	1938-1948; age 75-84	-0.81	(-2.02, 0.39)	0.61	
	1938 or earlier; 85 or older	-0.79	(-2.78, 1.20)	1.02	
Gender	(Ref: Male)				2.61e-06
	Female	-1.49	(-2.08, -0.90)	0.30	
	Other	-2.60	(-5.93, 0.73)	1.70	
Religious affiliation	(Ref: No religion/Atheist/Agnostic)				0.013
	Christianity	-0.62	(-1.73, 0.48)	0.56	
	Collapsed affiliations with prevalence<3%	-2.33	(-3.95, -0.72)	0.82	
Race/ethnicity	(Ref: Plurality group)				0.384
	Non-plurality groups	-0.18	(-0.96, 0.60)	0.38	

92. **Table S92. Sensitivity to unmeasured confounding of childhood predictors in Argentina**

Variable	Category	E-value for Estimate	E-value for 95% CI
Relationship with mother	(Ref: Very bad/somewhat bad)		
	Very good/somewhat good	1.05	1.00
Relationship with father	(Ref: Very bad/somewhat bad)		
	Very good/somewhat good	1.15	1.00
Parent marital status	(Ref: Parents married)		
	Divorced	1.43	1.00
	Parents were never married	1.18	1.00
	One or both parents had died	1.02	1.00
Subjective financial status of family growing up	(Ref: Got by)		
	Lived comfortably	1.12	1.00
	Found it difficult	1.07	1.00
	Found it very difficult	1.60	1.00
Abuse	(Ref: No)		
	Yes	1.42	1.00
Outsider growing up	(Ref: No)		
	Yes	1.43	1.00
Self-rated health growing up	(Ref: Good)		
	Excellent	1.36	1.00
	Very good	1.26	1.00
	Fair	1.18	1.00
	Poor	1.78	1.00
Immigration status	(Ref: Born in this country)		
	Born in another country	1.67	1.37
Age 12 religious service attendance	(Ref: Never)		
	At least 1/week	1.06	1.00
	1-3/month	1.30	1.00
	< 1/month	1.28	1.00
Year of birth	(Ref: 1998-2005; age: 18-24)		
	1988-1998; age 25-34	1.70	1.39
	1978-1988; age 35-44	1.96	1.63
	1968-1978; age 45-54	1.67	1.36
	1958-1968; age 55-64	1.74	1.37
	1948-1957; age 65-74	1.63	1.17
	1938-1948; age 75-84	1.42	1.00
	1938 or earlier; 85 or older	1.41	1.00
Gender	(Ref: Male)		
	Female	1.65	1.45
	Other	2.02	1.00
Religious affiliation	(Ref: No religion/Atheist/Agnostic)		
	Christianity	1.35	1.00
	Collapsed affiliations with prevalence<3%	1.93	1.39
Race/ethnicity	(Ref: Plurality group)		
	Non-plurality groups	1.16	1.00

93. **Table S93. Childhood predictors regression for Australia**

Variable	Category	Est	95% CI	SE	Global p-value
Relationship with mother	(Ref: Very bad/somewhat bad)				0.087
	Very good/somewhat good	-0.95	(-2.04, 0.14)	0.56	
Relationship with father	(Ref: Very bad/somewhat bad)				0.462
	Very good/somewhat good	-0.28	(-1.02, 0.47)	0.38	
Parent marital status	(Ref: Parents married)				0.077
	Divorced	1.19	(0.23, 2.15)	0.49	
	Parents were never married	0.73	(-0.52, 1.97)	0.63	
	One or both parents had died	0.09	(-1.24, 1.41)	0.68	
Subjective financial status of family growing up	(Ref: Got by)				0.902
	Lived comfortably	-0.12	(-0.54, 0.29)	0.21	
	Found it difficult	-0.21	(-0.91, 0.49)	0.36	
	Found it very difficult	0.07	(-1.42, 1.56)	0.76	
Abuse	(Ref: No)				0.427
	Yes	0.22	(-0.33, 0.77)	0.28	
Outsider growing up	(Ref: No)				0.317
	Yes	0.34	(-0.33, 1.00)	0.34	
Self-rated health growing up	(Ref: Good)				0.459
	Excellent	-0.15	(-0.74, 0.44)	0.30	
	Very good	0.10	(-0.55, 0.75)	0.33	
	Fair	-0.22	(-1.13, 0.69)	0.46	
	Poor	1.68	(-0.77, 4.14)	1.25	
Immigration status	(Ref: Born in this country)				0.390
	Born in another country	-0.21	(-0.71, 0.28)	0.25	
Age 12 religious service attendance	(Ref: Never)				0.841
	At least 1/week	-0.09	(-0.69, 0.51)	0.31	
	1-3/month	-0.25	(-0.89, 0.40)	0.33	
	< 1/month	0.03	(-0.61, 0.67)	0.33	
Year of birth	(Ref: 1998-2005; age: 18-24)				7.65e-11
	1988-1998; age 25-34	0.80	(0.29, 1.31)	0.26	
	1978-1988; age 35-44	1.71	(1.01, 2.42)	0.36	
	1968-1978; age 45-54	1.73	(1.09, 2.38)	0.33	
	1958-1968; age 55-64	1.57	(1.05, 2.10)	0.27	
	1948-1957; age 65-74	0.89	(0.34, 1.44)	0.28	
	1938-1948; age 75-84	0.67	(0.08, 1.25)	0.30	
	1938 or earlier; 85 or older	0.82	(-0.08, 1.73)	0.46	
Gender	(Ref: Male)				0.002
	Female	-0.66	(-1.06, -0.25)	0.21	
	Other	-1.46	(-2.72, -0.20)	0.64	
Religious affiliation	(Ref: No religion/Atheist/Agnostic)				0.699
	Christianity	-0.14	(-0.76, 0.48)	0.32	
	Collapsed affiliations with prevalence<3%	-0.39	(-1.31, 0.52)	0.47	
Race/ethnicity	(Ref: Plurality group)				0.660
	Non-plurality groups	-0.09	(-0.51, 0.34)	0.22	

94. Table S94. Sensitivity to unmeasured confounding of childhood predictors in Australia

Variable	Category	E-value for Estimate	E-value for 95% CI
Relationship with mother	(Ref: Very bad/somewhat bad)		
	Very good/somewhat good	1.71	1.00
Relationship with father	(Ref: Very bad/somewhat bad)		
	Very good/somewhat good	1.30	1.00
Parent marital status	(Ref: Parents married)		
	Divorced	1.85	1.27
	Parents were never married	1.58	1.00
	One or both parents had died	1.15	1.00
Subjective financial status of family growing up	(Ref: Got by)		
	Lived comfortably	1.18	1.00
	Found it difficult	1.25	1.00
	Found it very difficult	1.13	1.00
Abuse	(Ref: No)		
	Yes	1.26	1.00
Outsider growing up	(Ref: No)		
	Yes	1.34	1.00
Self-rated health growing up	(Ref: Good)		
	Excellent	1.21	1.00
	Very good	1.17	1.00
	Fair	1.26	1.00
	Poor	2.15	1.00
Immigration status	(Ref: Born in this country)		
	Born in another country	1.26	1.00
Age 12 religious service attendance	(Ref: Never)		
	At least 1/week	1.16	1.00
	1-3/month	1.28	1.00
	< 1/month	1.08	1.00
Year of birth	(Ref: 1998-2005; age: 18-24)		
	1988-1998; age 25-34	1.62	1.31
	1978-1988; age 35-44	2.16	1.75
	1968-1978; age 45-54	2.18	1.80
	1958-1968; age 55-64	2.08	1.77
	1948-1957; age 65-74	1.68	1.35
	1938-1948; age 75-84	1.54	1.14
	1938 or earlier; 85 or older	1.64	1.00
Gender	(Ref: Male)		
	Female	1.54	1.29
	Other	2.01	1.25
Religious affiliation	(Ref: No religion/Atheist/Agnostic)		
	Christianity	1.20	1.00
	Collapsed affiliations with prevalence<3%	1.38	1.00
Race/ethnicity	(Ref: Plurality group)		
	Non-plurality groups	1.15	1.00

95. Table S95. Childhood predictors regression for Brazil

Variable	Category	Est	95% CI	SE	Global p-value
Relationship with mother	(Ref: Very bad/somewhat bad)				0.033
	Very good/somewhat good	-0.70	(-1.37, -0.04)	0.34	
Relationship with father	(Ref: Very bad/somewhat bad)				0.018
	Very good/somewhat good	-0.48	(-0.90, -0.05)	0.21	
Parent marital status	(Ref: Parents married)				0.003
	Divorced	0.87	(0.36, 1.39)	0.26	
	Parents were never married	0.32	(-0.13, 0.77)	0.23	
	One or both parents had died	0.09	(-0.78, 0.97)	0.44	
Subjective financial status of family growing up	(Ref: Got by)				0.431
	Lived comfortably	0.05	(-0.29, 0.39)	0.17	
	Found it difficult	-0.19	(-0.58, 0.21)	0.20	
	Found it very difficult	0.30	(-0.36, 0.97)	0.34	
Abuse	(Ref: No)				0.067
	Yes	0.37	(-0.05, 0.79)	0.21	
Outsider growing up	(Ref: No)				0.004
	Yes	0.78	(0.24, 1.32)	0.27	
Self-rated health growing up	(Ref: Good)				0.049
	Excellent	0.52	(0.14, 0.90)	0.19	
	Very good	0.08	(-0.32, 0.48)	0.20	
	Fair	0.12	(-0.42, 0.66)	0.28	
	Poor	0.24	(-1.56, 2.05)	0.92	
Immigration status	(Ref: Born in this country)				0.121
	Born in another country	-0.82	(-1.86, 0.22)	0.53	
Age 12 religious service attendance	(Ref: Never)				0.446
	At least 1/week	-0.24	(-0.76, 0.28)	0.27	
	1-3/month	0.06	(-0.53, 0.65)	0.30	
	< 1/month	-0.11	(-0.66, 0.44)	0.28	
Year of birth	(Ref: 1998-2005; age: 18-24)				0.000
	1988-1998; age 25-34	1.29	(0.94, 1.63)	0.18	
	1978-1988; age 35-44	1.65	(1.29, 2.00)	0.18	
	1968-1978; age 45-54	1.85	(1.41, 2.28)	0.22	
	1958-1968; age 55-64	2.19	(1.64, 2.74)	0.28	
	1948-1957; age 65-74	1.82	(0.99, 2.64)	0.42	
	1938-1948; age 75-84	-0.44	(-0.91, 0.03)	0.24	
	1938 or earlier; 85 or older	0.68	(-1.01, 2.36)	0.86	
Gender	(Ref: Male)				9.32e-12
	Female	-1.07	(-1.37, -0.77)	0.15	
	Other	-1.82	(-3.27, -0.37)	0.74	
Religious affiliation	(Ref: No religion/Atheist/Agnostic)				2.69e-07
	Christianity	-0.07	(-0.62, 0.48)	0.28	
	Collapsed affiliations with prevalence<3%	2.32	(1.34, 3.29)	0.50	
Race/ethnicity	(Ref: Plurality group)				0.032
	Non-plurality groups	-0.32	(-0.61, -0.02)	0.15	

96. **Table S96. Sensitivity to unmeasured confounding of childhood predictors in Brazil**

Variable	Category	E-value for Estimate	E-value for 95% CI
Relationship with mother	(Ref: Very bad/somewhat bad)		
	Very good/somewhat good	1.45	1.08
Relationship with father	(Ref: Very bad/somewhat bad)		
	Very good/somewhat good	1.34	1.10
Parent marital status	(Ref: Parents married)		
	Divorced	1.52	1.29
	Parents were never married	1.27	1.00
	One or both parents had died	1.13	1.00
Subjective financial status of family growing up	(Ref: Got by)		
	Lived comfortably	1.09	1.00
	Found it difficult	1.19	1.00
	Found it very difficult	1.26	1.00
Abuse	(Ref: No)		
	Yes	1.29	1.00
Outsider growing up	(Ref: No)		
	Yes	1.48	1.23
Self-rated health growing up	(Ref: Good)		
	Excellent	1.36	1.16
	Very good	1.12	1.00
	Fair	1.15	1.00
	Poor	1.23	1.00
Immigration status	(Ref: Born in this country)		
	Born in another country	1.50	1.00
Age 12 religious service attendance	(Ref: Never)		
	At least 1/week	1.22	1.00
	1-3/month	1.10	1.00
	< 1/month	1.14	1.00
Year of birth	(Ref: 1998-2005; age: 18-24)		
	1988-1998; age 25-34	1.69	1.55
	1978-1988; age 35-44	1.84	1.69
	1968-1978; age 45-54	1.93	1.75
	1958-1968; age 55-64	2.07	1.84
	1948-1957; age 65-74	1.91	1.57
	1938-1948; age 75-84	1.33	1.00
	1938 or earlier; 85 or older	1.43	1.00
Gender	(Ref: Male)		
	Female	1.60	1.47
	Other	1.92	1.30
Religious affiliation	(Ref: No religion/Atheist/Agnostic)		
	Christianity	1.11	1.00
	Collapsed affiliations with prevalence<3%	2.13	1.72
Race/ethnicity	(Ref: Plurality group)		
	Non-plurality groups	1.27	1.06

97. Table S97. Childhood predictors regression for Egypt

Variable	Category	Est	95% CI	SE	Global p-value
Relationship with mother	(Ref: Very bad/somewhat bad)				0.009
	Very good/somewhat good	2.45	(0.42, 4.48)	1.03	
Relationship with father	(Ref: Very bad/somewhat bad)				0.035
	Very good/somewhat good	-2.79	(-5.37, -0.22)	1.31	
Parent marital status	(Ref: Parents married)				0.038
	Divorced	1.20	(-0.72, 3.11)	0.98	
	Parents were never married	0.13	(-3.33, 3.58)	1.76	
	One or both parents had died	1.50	(0.40, 2.59)	0.56	
Subjective financial status of family growing up	(Ref: Got by)				0.833
	Lived comfortably	0.03	(-0.52, 0.59)	0.28	
	Found it difficult	0.16	(-0.62, 0.94)	0.40	
	Found it very difficult	0.48	(-0.61, 1.57)	0.55	
Abuse	(Ref: No)				0.020
	Yes	1.56	(0.28, 2.84)	0.65	
Outsider growing up	(Ref: No)				0.789
	Yes	-0.15	(-1.30, 0.99)	0.58	
Self-rated health growing up	(Ref: Good)				0.030
	Excellent	0.25	(-0.57, 1.07)	0.42	
	Very good	0.45	(-0.49, 1.40)	0.48	
	Fair	-0.80	(-1.81, 0.20)	0.51	
	Poor	-1.04	(-2.38, 0.30)	0.68	
Immigration status	(Ref: Born in this country)				0.465
	Born in another country	-0.89	(-3.70, 1.93)	1.43	
Age 12 religious service attendance	(Ref: Never)				0.203
	At least 1/week	-0.09	(-0.68, 0.49)	0.30	
	1-3/month	-0.75	(-1.51, 0.01)	0.39	
	< 1/month	0.06	(-0.50, 0.62)	0.28	
Year of birth	(Ref: 1998-2005; age: 18-24)				2.64e-06
	1988-1998; age 25-34	0.98	(0.21, 1.74)	0.39	
	1978-1988; age 35-44	0.36	(-0.38, 1.10)	0.38	
	1968-1978; age 45-54	0.31	(-0.53, 1.15)	0.43	
	1958-1968; age 55-64	1.11	(0.03, 2.19)	0.55	
	1948-1957; age 65-74	-0.93	(-2.51, 0.66)	0.81	
	1938-1948; age 75-84	-2.66	(-5.31, -0.02)	1.35	
	1938 or earlier; 85 or older	-4.85	(-6.85, -2.86)	1.02	
Gender	(Ref: Male)				0.000
	Female	-6.48	(-7.08, -5.87)	0.31	
Religious affiliation	(Ref: Islam)				0.579
	Collapsed affiliations with prevalence<3%	-0.52	(-2.35, 1.31)	0.93	
Race/ethnicity	(Ref: Plurality group)				0.488
	Non-plurality groups	-0.43	(-1.64, 0.78)	0.62	

98. Table S98. Sensitivity to unmeasured confounding of childhood predictors in Egypt

Variable	Category	E-value for Estimate	E-value for 95% CI
Relationship with mother	(Ref: Very bad/somewhat bad)		
	Very good/somewhat good	2.01	1.29
Relationship with father	(Ref: Very bad/somewhat bad)		
	Very good/somewhat good	2.14	1.19
Parent marital status	(Ref: Parents married)		
	Divorced	1.57	1.00
	Parents were never married	1.14	1.00
	One or both parents had died	1.68	1.28
Subjective financial status of family growing up	(Ref: Got by)		
	Lived comfortably	1.07	1.00
	Found it difficult	1.16	1.00
	Found it very difficult	1.31	1.00
Abuse	(Ref: No)		
	Yes	1.70	1.22
Outsider growing up	(Ref: No)		
	Yes	1.16	1.00
Self-rated health growing up	(Ref: Good)		
	Excellent	1.21	1.00
	Very good	1.30	1.00
	Fair	1.43	1.00
	Poor	1.52	1.00
Immigration status	(Ref: Born in this country)		
	Born in another country	1.46	1.00
Age 12 religious service attendance	(Ref: Never)		
	At least 1/week	1.12	1.00
	1-3/month	1.41	1.00
	< 1/month	1.09	1.00
Year of birth	(Ref: 1998-2005; age: 18-24)		
	1988-1998; age 25-34	1.50	1.19
	1978-1988; age 35-44	1.26	1.00
	1968-1978; age 45-54	1.24	1.00
	1958-1968; age 55-64	1.54	1.06
	1948-1957; age 65-74	1.48	1.00
	1938-1948; age 75-84	2.09	1.05
	1938 or earlier; 85 or older	2.97	2.16
Gender	(Ref: Male)		
	Female	3.75	3.44
Religious affiliation	(Ref: Islam)		
	Collapsed affiliations with prevalence<3%	1.32	1.00
Race/ethnicity	(Ref: Plurality group)		
	Non-plurality groups	1.29	1.00

99. **Table S99. Childhood predictors regression for Germany**

Variable	Category	Est	95% CI	SE	Global p-value
Relationship with mother	(Ref: Very bad/somewhat bad)				0.077
	Very good/somewhat good	-0.85	(-1.80, 0.11)	0.49	
Relationship with father	(Ref: Very bad/somewhat bad)				0.195
	Very good/somewhat good	-0.42	(-1.09, 0.24)	0.34	
Parent marital status	(Ref: Parents married)				0.225
	Divorced	0.21	(-0.47, 0.89)	0.35	
	Parents were never married	0.51	(-0.36, 1.37)	0.44	
	One or both parents had died	-0.71	(-1.77, 0.34)	0.54	
Subjective financial status of family growing up	(Ref: Got by)				0.400
	Lived comfortably	0.15	(-0.36, 0.67)	0.26	
	Found it difficult	-0.17	(-0.80, 0.45)	0.32	
	Found it very difficult	0.75	(-0.43, 1.92)	0.60	
Abuse	(Ref: No)				5.62e-04
	Yes	1.15	(0.47, 1.83)	0.35	
Outsider growing up	(Ref: No)				0.635
	Yes	0.16	(-0.62, 0.93)	0.40	
Self-rated health growing up	(Ref: Good)				0.361
	Excellent	0.49	(-0.13, 1.11)	0.32	
	Very good	0.08	(-0.42, 0.59)	0.26	
	Fair	0.49	(-0.42, 1.40)	0.46	
	Poor	-0.37	(-1.95, 1.21)	0.81	
Immigration status	(Ref: Born in this country)				5.74e-04
	Born in another country	-1.18	(-1.85, -0.51)	0.34	
Age 12 religious service attendance	(Ref: Never)				0.728
	At least 1/week	0.35	(-0.29, 0.98)	0.33	
	1-3/month	0.07	(-0.52, 0.66)	0.30	
	< 1/month	0.15	(-0.40, 0.70)	0.28	
Year of birth	(Ref: 1998-2005; age: 18-24)				0.000
	1988-1998; age 25-34	0.99	(0.32, 1.66)	0.34	
	1978-1988; age 35-44	1.69	(0.98, 2.39)	0.36	
	1968-1978; age 45-54	2.69	(1.89, 3.48)	0.41	
	1958-1968; age 55-64	2.88	(2.08, 3.67)	0.40	
	1948-1957; age 65-74	1.63	(0.88, 2.38)	0.38	
	1938-1948; age 75-84	0.07	(-0.75, 0.89)	0.42	
	1938 or earlier; 85 or older	-1.24	(-2.38, -0.10)	0.58	
Gender	(Ref: Male)				0.005
	Female	-0.64	(-1.05, -0.24)	0.21	
	Other	-2.11	(-5.18, 0.95)	1.56	
Religious affiliation	(Ref: No religion/Atheist/Agnostic)				0.389
	Islam	0.56	(-0.83, 1.96)	0.71	
	Christianity	-0.22	(-0.72, 0.28)	0.26	
	Collapsed affiliations with prevalence<3%	1.00	(-1.04, 3.03)	1.04	
Race/ethnicity	(Ref: Plurality group)				

100. Table S100. Sensitivity to unmeasured confounding of childhood predictors in Germany

Variable	Category	E-value for Estimate	E-value for 95% CI
Relationship with mother	(Ref: Very bad/somewhat bad)		
	Very good/somewhat good	1.44	1.00
Relationship with father	(Ref: Very bad/somewhat bad)		
	Very good/somewhat good	1.28	1.00
Parent marital status	(Ref: Parents married)		
	Divorced	1.18	1.00
	Parents were never married	1.32	1.00
	One or both parents had died	1.40	1.00
Subjective financial status of family growing up	(Ref: Got by)		
	Lived comfortably	1.15	1.00
	Found it difficult	1.16	1.00
	Found it very difficult	1.41	1.00
Abuse	(Ref: No)		
	Yes	1.55	1.30
Outsider growing up	(Ref: No)		
	Yes	1.16	1.00
Self-rated health growing up	(Ref: Good)		
	Excellent	1.31	1.00
	Very good	1.11	1.00
	Fair	1.31	1.00
	Poor	1.26	1.00
Immigration status	(Ref: Born in this country)		
	Born in another country	1.56	1.32
Age 12 religious service attendance	(Ref: Never)		
	At least 1/week	1.25	1.00
	1-3/month	1.10	1.00
	< 1/month	1.15	1.00
Year of birth	(Ref: 1998-2005; age: 18-24)		
	1988-1998; age 25-34	1.49	1.24
	1978-1988; age 35-44	1.73	1.49
	1968-1978; age 45-54	2.08	1.81
	1958-1968; age 55-64	2.15	1.87
	1948-1957; age 65-74	1.72	1.46
	1938-1948; age 75-84	1.10	1.00
	1938 or earlier; 85 or older	1.58	1.12
Gender	(Ref: Male)		
	Female	1.37	1.20
	Other	1.88	1.00
Religious affiliation	(Ref: No religion/Atheist/Agnostic)		
	Islam	1.34	1.00
	Christianity	1.19	1.00
	Collapsed affiliations with prevalence<3%	1.50	1.00
Race/ethnicity	(Ref: Plurality group)		

101. **Table S101. Childhood predictors regression for Hong Kong**

Variable	Category	Est	95% CI	SE	Global p-value
Relationship with mother	(Ref: Very bad/somewhat bad)				0.107
	Very good/somewhat good	-0.61	(-1.35, 0.14)	0.38	
Relationship with father	(Ref: Very bad/somewhat bad)				0.269
	Very good/somewhat good	-0.35	(-0.97, 0.28)	0.32	
Parent marital status	(Ref: Parents married)				0.044
	Divorced	-0.83	(-1.58, -0.09)	0.38	
	Parents were never married	-0.84	(-1.68, -0.01)	0.42	
	One or both parents had died	-0.37	(-1.57, 0.82)	0.61	
Subjective financial status of family growing up	(Ref: Got by)				0.111
	Lived comfortably	-0.51	(-1.03, 0.01)	0.26	
	Found it difficult	-0.42	(-1.03, 0.20)	0.32	
	Found it very difficult	-0.49	(-1.63, 0.65)	0.58	
Abuse	(Ref: No)				0.657
	Yes	0.15	(-0.71, 1.00)	0.43	
Outsider growing up	(Ref: No)				1.28e-05
	Yes	1.51	(0.81, 2.21)	0.36	
Self-rated health growing up	(Ref: Good)				0.033
	Excellent	0.23	(-0.42, 0.89)	0.33	
	Very good	-0.02	(-0.64, 0.59)	0.31	
	Fair	-0.70	(-1.33, -0.07)	0.32	
	Poor	-0.96	(-2.49, 0.56)	0.75	
Immigration status	(Ref: Born in this country)				0.024
	Born in another country	-0.66	(-1.33, 0.00)	0.34	
Age 12 religious service attendance	(Ref: Never)				1.92e-04
	At least 1/week	0.14	(-0.61, 0.90)	0.39	
	1-3/month	1.50	(0.76, 2.25)	0.38	
	< 1/month	0.25	(-0.22, 0.72)	0.24	
Year of birth	(Ref: 1998-2005; age: 18-24)				2.15e-13
	1988-1998; age 25-34	0.21	(-0.46, 0.87)	0.34	
	1978-1988; age 35-44	0.30	(-0.31, 0.90)	0.31	
	1968-1978; age 45-54	1.52	(0.74, 2.30)	0.40	
	1958-1968; age 55-64	0.20	(-0.35, 0.75)	0.28	
	1948-1957; age 65-74	-0.67	(-1.41, 0.07)	0.38	
	1938-1948; age 75-84	-1.44	(-2.31, -0.58)	0.44	
	1938 or earlier; 85 or older	-1.59	(-2.74, -0.44)	0.59	
Gender	(Ref: Male)				4.85e-05
	Female	-0.78	(-1.18, -0.38)	0.20	
	Other	-1.75	(-3.40, -0.10)	0.82	
Religious affiliation	(Ref: No religion/Atheist/Agnostic)				0.102
	Buddhism	0.51	(-0.08, 1.09)	0.30	
	Chinese folk/traditional religion	0.81	(-0.14, 1.77)	0.49	
	Christianity	0.74	(0.02, 1.46)	0.37	
	Collapsed affiliations with prevalence<3%	0.26	(-0.76, 1.28)	0.51	
Race/ethnicity	(Ref: Plurality group)				0.249
	Non-plurality groups	-0.23	(-0.69, 0.23)	0.23	

102. Table S102. Sensitivity to unmeasured confounding of childhood predictors in Hong Kong

Variable	Category	E-value for Estimate	E-value for 95% CI
Relationship with mother	(Ref: Very bad/somewhat bad)		
	Very good/somewhat good	1.54	1.00
Relationship with father	(Ref: Very bad/somewhat bad)		
	Very good/somewhat good	1.37	1.00
Parent marital status	(Ref: Parents married)		
	Divorced	1.68	1.16
	Parents were never married	1.69	1.06
	One or both parents had died	1.39	1.00
Subjective financial status of family growing up	(Ref: Got by)		
	Lived comfortably	1.48	1.00
	Found it difficult	1.41	1.00
	Found it very difficult	1.46	1.00
Abuse	(Ref: No)		
	Yes	1.21	1.00
Outsider growing up	(Ref: No)		
	Yes	2.12	1.67
Self-rated health growing up	(Ref: Good)		
	Excellent	1.29	1.00
	Very good	1.08	1.00
	Fair	1.60	1.14
	Poor	1.76	1.00
Immigration status	(Ref: Born in this country)		
	Born in another country	1.57	1.03
Age 12 religious service attendance	(Ref: Never)		
	At least 1/week	1.21	1.00
	1-3/month	2.11	1.64
	< 1/month	1.30	1.00
Year of birth	(Ref: 1998-2005; age: 18-24)		
	1988-1998; age 25-34	1.26	1.00
	1978-1988; age 35-44	1.33	1.00
	1968-1978; age 45-54	2.12	1.62
	1958-1968; age 55-64	1.26	1.00
	1948-1957; age 65-74	1.58	1.00
	1938-1948; age 75-84	2.07	1.52
	1938 or earlier; 85 or older	2.17	1.43
Gender	(Ref: Male)		
	Female	1.65	1.39
	Other	2.28	1.21
Religious affiliation	(Ref: No religion/Atheist/Agnostic)		
	Buddhism	1.48	1.00
	Chinese folk/traditional religion	1.67	1.00
	Christianity	1.63	1.08
	Collapsed affiliations with prevalence<3%	1.30	1.00
Race/ethnicity	(Ref: Plurality group)		
	Non-plurality groups	1.28	1.00

103. Table S103. Childhood predictors regression for India

Variable	Category	Est	95% CI	SE	Global p-value
Relationship with mother	(Ref: Very bad/somewhat bad)				0.560
	Very good/somewhat good	0.34	(-0.80, 1.48)	0.58	
Relationship with father	(Ref: Very bad/somewhat bad)				0.350
	Very good/somewhat good	0.60	(-0.66, 1.87)	0.65	
Parent marital status	(Ref: Parents married)				0.052
	Divorced	-0.26	(-0.86, 0.35)	0.29	
	Parents were never married	0.06	(-0.29, 0.41)	0.17	
	One or both parents had died	0.21	(-0.31, 0.73)	0.26	
Subjective financial status of family growing up	(Ref: Got by)				0.346
	Lived comfortably	0.06	(-0.17, 0.28)	0.11	
	Found it difficult	0.05	(-0.23, 0.33)	0.14	
	Found it very difficult	0.39	(-0.06, 0.84)	0.23	
Abuse	(Ref: No)				0.005
	Yes	0.50	(0.14, 0.85)	0.18	
Outsider growing up	(Ref: No)				0.116
	Yes	0.23	(-0.06, 0.52)	0.15	
Self-rated health growing up	(Ref: Good)				0.957
	Excellent	0.01	(-0.24, 0.27)	0.13	
	Very good	0.01	(-0.25, 0.28)	0.14	
	Fair	-0.06	(-0.38, 0.26)	0.16	
	Poor	0.18	(-0.54, 0.89)	0.36	
Immigration status	(Ref: Born in this country)				0.079
	Born in another country	1.53	(-0.17, 3.24)	0.87	
Age 12 religious service attendance	(Ref: Never)				0.090
	At least 1/week	-0.18	(-0.56, 0.20)	0.19	
	1-3/month	0.12	(-0.29, 0.53)	0.21	
	< 1/month	-0.20	(-0.55, 0.15)	0.18	
Year of birth	(Ref: 1998-2005; age: 18-24)				0.003
	1988-1998; age 25-34	0.21	(-0.14, 0.55)	0.18	
	1978-1988; age 35-44	0.17	(-0.12, 0.46)	0.15	
	1968-1978; age 45-54	0.62	(0.25, 1.00)	0.19	
	1958-1968; age 55-64	0.49	(0.10, 0.87)	0.20	
	1948-1957; age 65-74	0.03	(-0.35, 0.42)	0.20	
	1938-1948; age 75-84	0.45	(-0.47, 1.38)	0.47	
	1938 or earlier; 85 or older	-0.20	(-0.73, 0.32)	0.27	
Gender	(Ref: Male)				0.000
	Female	-1.02	(-1.22, -0.82)	0.10	
Religious affiliation	(Ref: Hinduism)				0.109
	Islam	0.42	(-0.05, 0.90)	0.24	
	Collapsed affiliations with prevalence<3%	-0.21	(-0.59, 0.16)	0.19	
Race/ethnicity	(Ref: Plurality group)				0.319
	Non-plurality groups	0.10	(-0.11, 0.32)	0.11	

104. Table S104. Sensitivity to unmeasured confounding of childhood predictors in India

Variable	Category	E-value for Estimate	E-value for 95% CI
Relationship with mother	(Ref: Very bad/somewhat bad)		
	Very good/somewhat good	1.34	1.00
Relationship with father	(Ref: Very bad/somewhat bad)		
	Very good/somewhat good	1.50	1.00
Parent marital status	(Ref: Parents married)		
	Divorced	1.28	1.00
	Parents were never married	1.12	1.00
	One or both parents had died	1.25	1.00
Subjective financial status of family growing up	(Ref: Got by)		
	Lived comfortably	1.12	1.00
	Found it difficult	1.11	1.00
	Found it very difficult	1.37	1.00
Abuse	(Ref: No)		
	Yes	1.44	1.20
Outsider growing up	(Ref: No)		
	Yes	1.26	1.00
Self-rated health growing up	(Ref: Good)		
	Excellent	1.05	1.00
	Very good	1.05	1.00
	Fair	1.12	1.00
	Poor	1.23	1.00
Immigration status	(Ref: Born in this country)		
	Born in another country	2.04	1.00
Age 12 religious service attendance	(Ref: Never)		
	At least 1/week	1.23	1.00
	1-3/month	1.18	1.00
	< 1/month	1.24	1.00
Year of birth	(Ref: 1998-2005; age: 18-24)		
	1988-1998; age 25-34	1.25	1.00
	1978-1988; age 35-44	1.22	1.00
	1968-1978; age 45-54	1.51	1.28
	1958-1968; age 55-64	1.43	1.17
	1948-1957; age 65-74	1.09	1.00
	1938-1948; age 75-84	1.41	1.00
	1938 or earlier; 85 or older	1.25	1.00
Gender	(Ref: Male)		
	Female	1.74	1.63
Religious affiliation	(Ref: Hinduism)		
	Islam	1.39	1.00
	Collapsed affiliations with prevalence<3%	1.25	1.00
Race/ethnicity	(Ref: Plurality group)		
	Non-plurality groups	1.16	1.00

105. Table S105. Childhood predictors regression for Indonesia

Variable	Category	Est	95% CI	SE	Global p-value
Relationship with mother	(Ref: Very bad/somewhat bad)				0.122
	Very good/somewhat good	-1.34	(-3.07, 0.39)	0.88	
Relationship with father	(Ref: Very bad/somewhat bad)				0.638
	Very good/somewhat good	-0.28	(-1.60, 1.04)	0.67	
Parent marital status	(Ref: Parents married)				0.449
	Divorced	0.12	(-0.58, 0.81)	0.35	
	Parents were never married	-0.65	(-2.25, 0.95)	0.81	
	One or both parents had died	0.51	(-0.24, 1.26)	0.38	
Subjective financial status of family growing up	(Ref: Got by)				0.050
	Lived comfortably	-0.35	(-0.71, 0.00)	0.18	
	Found it difficult	-0.08	(-0.87, 0.71)	0.40	
	Found it very difficult	1.36	(-0.13, 2.84)	0.76	
Abuse	(Ref: No)				0.106
	Yes	0.66	(-0.15, 1.48)	0.41	
Outsider growing up	(Ref: No)				0.263
	Yes	0.59	(-0.55, 1.73)	0.58	
Self-rated health growing up	(Ref: Good)				0.725
	Excellent	-0.05	(-0.62, 0.51)	0.29	
	Very good	0.00	(-0.44, 0.45)	0.23	
	Fair	0.28	(-0.22, 0.78)	0.26	
	Poor	-0.79	(-3.14, 1.56)	1.20	
Immigration status	(Ref: Born in this country)				0.002
	Born in another country	-1.55	(-3.66, 0.57)	1.00	
Age 12 religious service attendance	(Ref: Never)				0.698
	At least 1/week	-0.11	(-0.89, 0.68)	0.40	
	1-3/month	-0.04	(-0.90, 0.83)	0.44	
	< 1/month	0.30	(-0.73, 1.33)	0.52	
Year of birth	(Ref: 1998-2005; age: 18-24)				3.80e-10
	1988-1998; age 25-34	1.36	(0.82, 1.89)	0.27	
	1978-1988; age 35-44	1.50	(0.91, 2.09)	0.30	
	1968-1978; age 45-54	1.04	(0.45, 1.63)	0.30	
	1958-1968; age 55-64	0.92	(0.11, 1.72)	0.41	
	1948-1957; age 65-74	0.19	(-0.95, 1.33)	0.58	
	1938-1948; age 75-84	-4.39	(-6.91, -1.87)	1.29	
	1938 or earlier; 85 or older	0.81	(-3.62, 5.23)	2.26	
Gender	(Ref: Male)				0.000
	Female	-6.96	(-7.35, -6.57)	0.20	
	Other	-0.78	(-20.27, 18.71)	8.50	
Religious affiliation	(Ref: Islam)				0.022
	Christianity	-0.35	(-1.30, 0.61)	0.49	
	Collapsed affiliations with prevalence<3%	-2.66	(-4.61, -0.70)	1.00	
Race/ethnicity	(Ref: Plurality group)				0.001
	Non-plurality groups	0.75	(0.33, 1.16)	0.21	

106. Table S106. Sensitivity to unmeasured confounding of childhood predictors in Indonesia

Variable	Category	E-value for Estimate	E-value for 95% CI
Relationship with mother	(Ref: Very bad/somewhat bad)		
	Very good/somewhat good	1.70	1.00
Relationship with father	(Ref: Very bad/somewhat bad)		
	Very good/somewhat good	1.24	1.00
Parent marital status	(Ref: Parents married)		
	Divorced	1.15	1.00
	Parents were never married	1.42	1.00
	One or both parents had died	1.36	1.00
Subjective financial status of family growing up	(Ref: Got by)		
	Lived comfortably	1.28	1.00
	Found it difficult	1.12	1.00
	Found it very difficult	1.71	1.00
Abuse	(Ref: No)		
	Yes	1.42	1.00
Outsider growing up	(Ref: No)		
	Yes	1.39	1.00
Self-rated health growing up	(Ref: Good)		
	Excellent	1.09	1.00
	Very good	1.02	1.00
	Fair	1.24	1.00
	Poor	1.48	1.00
Immigration status	(Ref: Born in this country)		
	Born in another country	1.79	1.00
Age 12 religious service attendance	(Ref: Never)		
	At least 1/week	1.14	1.00
	1-3/month	1.08	1.00
	< 1/month	1.25	1.00
Year of birth	(Ref: 1998-2005; age: 18-24)		
	1988-1998; age 25-34	1.71	1.49
	1978-1988; age 35-44	1.77	1.53
	1968-1978; age 45-54	1.58	1.33
	1958-1968; age 55-64	1.53	1.14
	1948-1957; age 65-74	1.19	1.00
	1938-1948; age 75-84	3.09	1.92
	1938 or earlier; 85 or older	1.48	1.00
Gender	(Ref: Male)		
	Female	4.71	4.43
	Other	1.47	1.00
Religious affiliation	(Ref: Islam)		
	Christianity	1.28	1.00
	Collapsed affiliations with prevalence<3%	2.25	1.44
Race/ethnicity	(Ref: Plurality group)		
	Non-plurality groups	1.46	1.27

107. Table S107. Childhood predictors regression for Israel

Variable	Category	Est	95% CI	SE	Global p-value
Relationship with mother	(Ref: Very bad/somewhat bad)				0.210
	Very good/somewhat good	0.83	(-0.48, 2.14)	0.67	
Relationship with father	(Ref: Very bad/somewhat bad)				0.092
	Very good/somewhat good	-0.90	(-1.93, 0.13)	0.53	
Parent marital status	(Ref: Parents married)				0.067
	Divorced	1.08	(-0.80, 2.96)	0.92	
	Parents were never married	0.93	(-1.98, 3.84)	1.48	
	One or both parents had died	-0.81	(-1.83, 0.20)	0.51	
Subjective financial status of family growing up	(Ref: Got by)				0.053
	Lived comfortably	-0.04	(-0.75, 0.67)	0.36	
	Found it difficult	0.94	(0.10, 1.78)	0.43	
	Found it very difficult	-0.41	(-1.36, 0.55)	0.49	
Abuse	(Ref: No)				
Outsider growing up	(Ref: No)				0.893
	Yes	0.06	(-0.85, 0.98)	0.47	
Self-rated health growing up	(Ref: Good)				0.057
	Excellent	-0.18	(-0.95, 0.59)	0.39	
	Very good	0.26	(-0.52, 1.03)	0.40	
	Fair	0.40	(-1.32, 2.12)	0.88	
	Poor	-3.53	(-6.48, -0.57)	1.51	
Immigration status	(Ref: Born in this country)				0.574
	Born in another country	-0.19	(-0.87, 0.49)	0.35	
Age 12 religious service attendance	(Ref: Never)				0.009
	At least 1/week	-0.63	(-1.67, 0.42)	0.53	
	1-3/month	-1.57	(-2.49, -0.65)	0.47	
	< 1/month	-0.80	(-1.48, -0.12)	0.35	
Year of birth	(Ref: 1998-2005; age: 18-24)				7.99e-08
	1988-1998; age 25-34	1.59	(0.42, 2.75)	0.60	
	1978-1988; age 35-44	2.24	(1.36, 3.13)	0.45	
	1968-1978; age 45-54	1.50	(0.69, 2.31)	0.41	
	1958-1968; age 55-64	2.16	(1.19, 3.13)	0.50	
	1948-1957; age 65-74	0.40	(-0.65, 1.45)	0.53	
	1938-1948; age 75-84	-0.17	(-1.29, 0.95)	0.57	
	1938 or earlier; 85 or older	-1.03	(-3.17, 1.12)	1.09	
Gender	(Ref: Male)				1.787e-15
	Female	-3.73	(-4.44, -3.01)	0.36	
	Other	-1.41	(-2.79, -0.02)	0.70	
Religious affiliation	(Ref: Judaism)				0.101
	Islam	-0.90	(-5.48, 3.68)	2.31	
	Collapsed affiliations with prevalence<3%	1.35	(-0.18, 2.89)	0.78	
Race/ethnicity	(Ref: Plurality group)				0.131
	Non-plurality groups	3.14	(-1.52, 7.80)	2.35	

108. Table S108. Sensitivity to unmeasured confounding of childhood predictors in Israel

Variable	Category	E-value for Estimate	E-value for 95% CI
Relationship with mother	(Ref: Very bad/somewhat bad)		
	Very good/somewhat good	1.46	1.00
Relationship with father	(Ref: Very bad/somewhat bad)		
	Very good/somewhat good	1.48	1.00
Parent marital status	(Ref: Parents married)		
	Divorced	1.55	1.00
	Parents were never married	1.49	1.00
	One or both parents had died	1.45	1.00
Subjective financial status of family growing up	(Ref: Got by)		
	Lived comfortably	1.08	1.00
	Found it difficult	1.50	1.13
	Found it very difficult	1.29	1.00
Abuse	(Ref: No)		
Outsider growing up	(Ref: No)		
	Yes	1.10	1.00
Self-rated health growing up	(Ref: Good)		
	Excellent	1.18	1.00
	Very good	1.22	1.00
	Fair	1.28	1.00
	Poor	2.48	1.36
Immigration status	(Ref: Born in this country)		
	Born in another country	1.18	1.00
Age 12 religious service attendance	(Ref: Never)		
	At least 1/week	1.38	1.00
	1-3/month	1.73	1.39
	< 1/month	1.44	1.14
Year of birth	(Ref: 1998-2005; age: 18-24)		
	1988-1998; age 25-34	1.73	1.29
	1978-1988; age 35-44	1.98	1.65
	1968-1978; age 45-54	1.70	1.40
	1958-1968; age 55-64	1.94	1.59
	1948-1957; age 65-74	1.28	1.00
	1938-1948; age 75-84	1.17	1.00
	1938 or earlier; 85 or older	1.53	1.00
Gender	(Ref: Male)		
	Female	2.56	2.27
	Other	1.67	1.07
Religious affiliation	(Ref: Judaism)		
	Islam	1.48	1.00
	Collapsed affiliations with prevalence<3%	1.65	1.00
Race/ethnicity	(Ref: Plurality group)		
	Non-plurality groups	2.32	1.00

109. Table S109. Childhood predictors regression for Japan

Variable	Category	Est	95% CI	SE	Global p-value
Relationship with mother	(Ref: Very bad/somewhat bad)				0.771
	Very good/somewhat good	-0.04	(-0.35, 0.27)	0.16	
Relationship with father	(Ref: Very bad/somewhat bad)				0.008
	Very good/somewhat good	-0.39	(-0.68, -0.09)	0.15	
Parent marital status	(Ref: Parents married)				7.88e-05
	Divorced	1.32	(0.74, 1.90)	0.30	
	Parents were never married	0.19	(-0.67, 1.05)	0.42	
	One or both parents had died	0.23	(-0.42, 0.88)	0.33	
Subjective financial status of family growing up	(Ref: Got by)				0.888
	Lived comfortably	-0.01	(-0.25, 0.23)	0.12	
	Found it difficult	-0.14	(-0.50, 0.22)	0.18	
	Found it very difficult	-0.06	(-0.64, 0.52)	0.30	
Abuse	(Ref: No)				0.011
	Yes	0.65	(0.15, 1.16)	0.26	
Outsider growing up	(Ref: No)				0.076
	Yes	0.36	(-0.08, 0.79)	0.22	
Self-rated health growing up	(Ref: Good)				0.011
	Excellent	0.42	(0.06, 0.78)	0.18	
	Very good	0.43	(0.18, 0.69)	0.13	
	Fair	0.09	(-0.25, 0.42)	0.17	
	Poor	0.13	(-0.45, 0.72)	0.30	
Immigration status	(Ref: Born in this country)				0.131
	Born in another country	-0.59	(-1.44, 0.25)	0.43	
Age 12 religious service attendance	(Ref: Never)				0.770
	At least 1/week	-0.01	(-0.80, 0.79)	0.41	
	1-3/month	0.27	(-0.31, 0.85)	0.29	
	< 1/month	0.07	(-0.18, 0.33)	0.13	
Year of birth	(Ref: 1998-2005; age: 18-24)				0.000
	1988-1998; age 25-34	1.33	(0.95, 1.71)	0.20	
	1978-1988; age 35-44	2.95	(2.54, 3.36)	0.21	
	1968-1978; age 45-54	3.41	(3.02, 3.80)	0.20	
	1958-1968; age 55-64	3.68	(3.26, 4.09)	0.21	
	1948-1957; age 65-74	1.84	(1.50, 2.18)	0.17	
	1938-1948; age 75-84	0.54	(0.15, 0.93)	0.20	
	1938 or earlier; 85 or older	0.13	(-0.59, 0.85)	0.37	
Gender	(Ref: Male)				0.000
	Female	-2.78	(-2.99, -2.56)	0.11	
	Other	-3.45	(-4.74, -2.15)	0.66	
Religious affiliation	(Ref: No religion/Atheist/Agnostic)				0.160
	Buddhism	0.24	(-0.01, 0.49)	0.13	
	Collapsed affiliations with prevalence<3%	0.23	(-0.39, 0.85)	0.32	
Race/ethnicity	(Ref: Plurality group)				

110. Table S110. Sensitivity to unmeasured confounding of childhood predictors in Japan

Variable	Category	E-value for Estimate	E-value for 95% CI
Relationship with mother	(Ref: Very bad/somewhat bad)		
	Very good/somewhat good	1.08	1.00
Relationship with father	(Ref: Very bad/somewhat bad)		
	Very good/somewhat good	1.29	1.13
Parent marital status	(Ref: Parents married)		
	Divorced	1.67	1.44
	Parents were never married	1.19	1.00
	One or both parents had died	1.21	1.00
Subjective financial status of family growing up	(Ref: Got by)		
	Lived comfortably	1.04	1.00
	Found it difficult	1.16	1.00
	Found it very difficult	1.10	1.00
Abuse	(Ref: No)		
	Yes	1.41	1.16
Outsider growing up	(Ref: No)		
	Yes	1.27	1.00
Self-rated health growing up	(Ref: Good)		
	Excellent	1.30	1.10
	Very good	1.31	1.18
	Fair	1.12	1.00
	Poor	1.15	1.00
Immigration status	(Ref: Born in this country)		
	Born in another country	1.38	1.00
Age 12 religious service attendance	(Ref: Never)		
	At least 1/week	1.03	1.00
	1-3/month	1.23	1.00
	< 1/month	1.11	1.00
Year of birth	(Ref: 1998-2005; age: 18-24)		
	1988-1998; age 25-34	1.67	1.53
	1978-1988; age 35-44	2.32	2.15
	1968-1978; age 45-54	2.52	2.35
	1958-1968; age 55-64	2.65	2.46
	1948-1957; age 65-74	1.87	1.74
	1938-1948; age 75-84	1.36	1.17
	1938 or earlier; 85 or older	1.15	1.00
Gender	(Ref: Male)		
	Female	2.25	2.16
	Other	2.54	2.00
Religious affiliation	(Ref: No religion/Atheist/Agnostic)		
	Buddhism	1.21	1.00
	Collapsed affiliations with prevalence<3%	1.21	1.00
Race/ethnicity	(Ref: Plurality group)		

111. Table S111. Childhood predictors regression for Kenya

Variable	Category	Est	95% CI	SE	Global p-value
Relationship with mother	(Ref: Very bad/somewhat bad)				0.076
	Very good/somewhat good	0.13	(-0.02, 0.28)	0.08	
Relationship with father	(Ref: Very bad/somewhat bad)				0.373
	Very good/somewhat good	0.11	(-0.14, 0.36)	0.13	
Parent marital status	(Ref: Parents married)				0.475
	Divorced	0.09	(-0.10, 0.28)	0.10	
	Parents were never married	-0.03	(-0.20, 0.14)	0.09	
	One or both parents had died	0.14	(-0.16, 0.44)	0.15	
Subjective financial status of family growing up	(Ref: Got by)				0.378
	Lived comfortably	-0.06	(-0.18, 0.06)	0.06	
	Found it difficult	0.05	(-0.07, 0.17)	0.06	
	Found it very difficult	-0.07	(-0.24, 0.09)	0.08	
Abuse	(Ref: No)				0.008
	Yes	0.26	(0.07, 0.45)	0.10	
Outsider growing up	(Ref: No)				0.023
	Yes	0.23	(0.03, 0.42)	0.10	
Self-rated health growing up	(Ref: Good)				5.71e-06
	Excellent	0.02	(-0.11, 0.14)	0.06	
	Very good	-0.06	(-0.17, 0.05)	0.06	
	Fair	-0.10	(-0.25, 0.05)	0.08	
	Poor	-0.29	(-0.42, -0.17)	0.06	
Immigration status	(Ref: Born in this country)				0.141
	Born in another country	-0.13	(-0.30, 0.04)	0.09	
Age 12 religious service attendance	(Ref: Never)				0.977
	At least 1/week	0.06	(-0.70, 0.81)	0.38	
	1-3/month	0.04	(-0.71, 0.79)	0.38	
	< 1/month	0.00	(-0.80, 0.80)	0.41	
Year of birth	(Ref: 1998-2005; age: 18-24)				5.58e-08
	1988-1998; age 25-34	0.10	(0.01, 0.18)	0.04	
	1978-1988; age 35-44	0.20	(0.08, 0.31)	0.06	
	1968-1978; age 45-54	0.34	(0.17, 0.52)	0.09	
	1958-1968; age 55-64	0.39	(0.14, 0.63)	0.13	
	1948-1957; age 65-74	0.27	(0.04, 0.49)	0.11	
	1938-1948; age 75-84	-0.11	(-0.27, 0.05)	0.08	
	1938 or earlier; 85 or older	-0.16	(-0.35, 0.02)	0.10	
Gender	(Ref: Male)				0.000
	Female	-0.45	(-0.53, -0.36)	0.04	
	Other	-0.56	(-0.76, -0.37)	0.10	
Religious affiliation	(Ref: Christianity)				0.025
	Islam	0.18	(0.01, 0.34)	0.08	
	Collapsed affiliations with prevalence<3%	1.33	(-0.40, 3.07)	0.88	
Race/ethnicity	(Ref: Plurality group)				0.042
	Non-plurality groups	-0.12	(-0.24, 0.01)	0.06	

112. Table S112. Sensitivity to unmeasured confounding of childhood predictors in Kenya

Variable	Category	E-value for Estimate	E-value for 95% CI
Relationship with mother	(Ref: Very bad/somewhat bad)		
	Very good/somewhat good	1.32	1.00
Relationship with father	(Ref: Very bad/somewhat bad)		
	Very good/somewhat good	1.30	1.00
Parent marital status	(Ref: Parents married)		
	Divorced	1.26	1.00
	Parents were never married	1.13	1.00
	One or both parents had died	1.34	1.00
Subjective financial status of family growing up	(Ref: Got by)		
	Lived comfortably	1.19	1.00
	Found it difficult	1.18	1.00
	Found it very difficult	1.23	1.00
Abuse	(Ref: No)		
	Yes	1.51	1.21
Outsider growing up	(Ref: No)		
	Yes	1.47	1.14
Self-rated health growing up	(Ref: Good)		
	Excellent	1.10	1.00
	Very good	1.20	1.00
	Fair	1.28	1.00
	Poor	1.56	1.39
Immigration status	(Ref: Born in this country)		
	Born in another country	1.32	1.00
Age 12 religious service attendance	(Ref: Never)		
	At least 1/week	1.19	1.00
	1-3/month	1.16	1.00
	< 1/month	1.04	1.00
Year of birth	(Ref: 1998-2005; age: 18-24)		
	1988-1998; age 25-34	1.27	1.09
	1978-1988; age 35-44	1.42	1.24
	1968-1978; age 45-54	1.63	1.39
	1958-1968; age 55-64	1.69	1.34
	1948-1957; age 65-74	1.53	1.17
	1938-1948; age 75-84	1.29	1.00
	1938 or earlier; 85 or older	1.37	1.00
Gender	(Ref: Male)		
	Female	1.78	1.66
	Other	1.93	1.66
Religious affiliation	(Ref: Christianity)		
	Islam	1.40	1.09
	Collapsed affiliations with prevalence<3%	3.16	1.00
Race/ethnicity	(Ref: Plurality group)		
	Non-plurality groups	1.30	1.00

113. Table S113. Childhood predictors regression for Mexico

Variable	Category	Est	95% CI	SE	Global p-value
Relationship with mother	(Ref: Very bad/somewhat bad)				0.571
	Very good/somewhat good	-0.18	(-0.82, 0.47)	0.33	
Relationship with father	(Ref: Very bad/somewhat bad)				0.150
	Very good/somewhat good	0.23	(-0.12, 0.57)	0.17	
Parent marital status	(Ref: Parents married)				0.344
	Divorced	0.38	(-0.16, 0.91)	0.27	
	Parents were never married	-0.04	(-0.45, 0.38)	0.21	
	One or both parents had died	-0.08	(-0.73, 0.58)	0.34	
Subjective financial status of family growing up	(Ref: Got by)				0.001
	Lived comfortably	0.17	(-0.31, 0.65)	0.24	
	Found it difficult	-0.40	(-0.83, 0.04)	0.22	
	Found it very difficult	-0.74	(-1.30, -0.18)	0.29	
Abuse	(Ref: No)				0.476
	Yes	0.10	(-0.19, 0.39)	0.15	
Outsider growing up	(Ref: No)				0.371
	Yes	0.14	(-0.18, 0.45)	0.16	
Self-rated health growing up	(Ref: Good)				0.172
	Excellent	0.31	(-0.00, 0.61)	0.16	
	Very good	0.28	(-0.06, 0.62)	0.17	
	Fair	0.17	(-0.61, 0.94)	0.40	
	Poor	0.43	(-0.31, 1.17)	0.38	
Immigration status	(Ref: Born in this country)				0.028
	Born in another country	1.95	(0.21, 3.68)	0.88	
Age 12 religious service attendance	(Ref: Never)				0.394
	At least 1/week	-0.46	(-1.00, 0.09)	0.28	
	1-3/month	-0.44	(-1.03, 0.14)	0.30	
	< 1/month	-0.51	(-1.13, 0.12)	0.32	
Year of birth	(Ref: 1998-2005; age: 18-24)				0.305
	1988-1998; age 25-34	0.24	(-0.29, 0.77)	0.27	
	1978-1988; age 35-44	0.44	(-0.13, 1.01)	0.29	
	1968-1978; age 45-54	0.23	(-0.36, 0.82)	0.30	
	1958-1968; age 55-64	0.38	(-0.23, 0.99)	0.31	
	1948-1957; age 65-74	0.71	(-0.07, 1.50)	0.40	
	1938-1948; age 75-84	0.54	(-0.54, 1.63)	0.55	
	1938 or earlier; 85 or older	-0.61	(-1.57, 0.36)	0.49	
Gender	(Ref: Male)				7.33e-15
	Female	-1.31	(-1.63, -0.98)	0.17	
	Other	0.73	(-3.59, 5.06)	2.21	
Religious affiliation	(Ref: No religion/Atheist/Agnostic)				0.401
	Christianity	-1.03	(-2.55, 0.49)	0.78	
	Collapsed affiliations with prevalence<3%	-0.91	(-2.62, 0.80)	0.87	
Race/ethnicity	(Ref: Plurality group)				0.366
	Non-plurality groups	0.14	(-0.22, 0.51)	0.18	

114. Table S114. Sensitivity to unmeasured confounding of childhood predictors in Mexico

Variable	Category	E-value for Estimate	E-value for 95% CI
Relationship with mother	(Ref: Very bad/somewhat bad)		
	Very good/somewhat good	1.24	1.00
Relationship with father	(Ref: Very bad/somewhat bad)		
	Very good/somewhat good	1.28	1.00
Parent marital status	(Ref: Parents married)		
	Divorced	1.39	1.00
	Parents were never married	1.10	1.00
	One or both parents had died	1.15	1.00
Subjective financial status of family growing up	(Ref: Got by)		
	Lived comfortably	1.24	1.00
	Found it difficult	1.41	1.00
	Found it very difficult	1.63	1.25
Abuse	(Ref: No)		
	Yes	1.18	1.00
Outsider growing up	(Ref: No)		
	Yes	1.21	1.00
Self-rated health growing up	(Ref: Good)		
	Excellent	1.34	1.00
	Very good	1.32	1.00
	Fair	1.23	1.00
	Poor	1.43	1.00
Immigration status	(Ref: Born in this country)		
	Born in another country	2.45	1.28
Age 12 religious service attendance	(Ref: Never)		
	At least 1/week	1.45	1.00
	1-3/month	1.44	1.00
	< 1/month	1.48	1.00
Year of birth	(Ref: 1998-2005; age: 18-24)		
	1988-1998; age 25-34	1.30	1.00
	1978-1988; age 35-44	1.44	1.00
	1968-1978; age 45-54	1.29	1.00
	1958-1968; age 55-64	1.40	1.00
	1948-1957; age 65-74	1.62	1.00
	1938-1948; age 75-84	1.51	1.00
	1938 or earlier; 85 or older	1.55	1.00
Gender	(Ref: Male)		
	Female	2.01	1.79
	Other	1.63	1.00
Religious affiliation	(Ref: No religion/Atheist/Agnostic)		
	Christianity	1.82	1.00
	Collapsed affiliations with prevalence<3%	1.75	1.00
Race/ethnicity	(Ref: Plurality group)		
	Non-plurality groups	1.21	1.00

115. Table S115. Childhood predictors regression for Nigeria

Variable	Category	Est	95% CI	SE	Global p-value
Relationship with mother	(Ref: Very bad/somewhat bad)				0.342
	Very good/somewhat good	-0.21	(-0.65, 0.22)	0.22	
Relationship with father	(Ref: Very bad/somewhat bad)				0.805
	Very good/somewhat good	0.04	(-0.30, 0.38)	0.17	
Parent marital status	(Ref: Parents married)				0.045
	Divorced	-0.13	(-0.25, -0.00)	0.06	
	Parents were never married	0.10	(-0.34, 0.54)	0.22	
	One or both parents had died	0.22	(-0.03, 0.47)	0.13	
Subjective financial status of family growing up	(Ref: Got by)				0.407
	Lived comfortably	-0.13	(-0.29, 0.02)	0.08	
	Found it difficult	-0.05	(-0.22, 0.12)	0.09	
	Found it very difficult	-0.11	(-0.27, 0.06)	0.08	
Abuse	(Ref: No)				0.024
	Yes	0.19	(0.02, 0.35)	0.09	
Outsider growing up	(Ref: No)				0.515
	Yes	0.08	(-0.16, 0.32)	0.12	
Self-rated health growing up	(Ref: Good)				0.021
	Excellent	-0.08	(-0.29, 0.13)	0.11	
	Very good	-0.13	(-0.34, 0.07)	0.10	
	Fair	-0.24	(-0.44, -0.05)	0.10	
	Poor	0.13	(-0.40, 0.66)	0.27	
Immigration status	(Ref: Born in this country)				0.915
	Born in another country	0.03	(-0.61, 0.68)	0.33	
Age 12 religious service attendance	(Ref: Never)				0.099
	At least 1/week	0.13	(-0.02, 0.29)	0.08	
	1-3/month	0.37	(0.06, 0.68)	0.16	
	< 1/month	0.23	(-0.19, 0.66)	0.22	
Year of birth	(Ref: 1998-2005; age: 18-24)				0.023
	1988-1998; age 25-34	0.07	(-0.04, 0.19)	0.06	
	1978-1988; age 35-44	0.31	(0.08, 0.54)	0.12	
	1968-1978; age 45-54	0.06	(-0.15, 0.26)	0.10	
	1958-1968; age 55-64	-0.04	(-0.16, 0.09)	0.06	
	1948-1957; age 65-74	-0.10	(-0.26, 0.06)	0.08	
	1938-1948; age 75-84	0.21	(-0.11, 0.54)	0.17	
	1938 or earlier; 85 or older	0.51	(-0.34, 1.36)	0.44	
Gender	(Ref: Male)				1.37e-07
	Female	-0.32	(-0.43, -0.20)	0.06	
	Other	-0.47	(-0.68, -0.26)	0.11	
Religious affiliation	(Ref: Christianity)				0.373
	Islam	0.15	(-0.06, 0.36)	0.11	
	Collapsed affiliations with prevalence<3%	0.07	(-0.46, 0.59)	0.27	
Race/ethnicity	(Ref: Plurality group)				0.753
	Non-plurality groups	0.03	(-0.21, 0.27)	0.12	

116. Table S116. Sensitivity to unmeasured confounding of childhood predictors in Nigeria

Variable	Category	E-value for Estimate	E-value for 95% CI
Relationship with mother	(Ref: Very bad/somewhat bad)		
	Very good/somewhat good	1.37	1.00
Relationship with father	(Ref: Very bad/somewhat bad)		
	Very good/somewhat good	1.14	1.00
Parent marital status	(Ref: Parents married)		
	Divorced	1.27	1.02
	Parents were never married	1.23	1.00
	One or both parents had died	1.38	1.00
Subjective financial status of family growing up	(Ref: Got by)		
	Lived comfortably	1.27	1.00
	Found it difficult	1.16	1.00
	Found it very difficult	1.24	1.00
Abuse	(Ref: No)		
	Yes	1.34	1.10
Outsider growing up	(Ref: No)		
	Yes	1.20	1.00
Self-rated health growing up	(Ref: Good)		
	Excellent	1.20	1.00
	Very good	1.28	1.00
	Fair	1.41	1.16
	Poor	1.28	1.00
Immigration status	(Ref: Born in this country)		
	Born in another country	1.13	1.00
Age 12 religious service attendance	(Ref: Never)		
	At least 1/week	1.28	1.00
	1-3/month	1.55	1.18
	< 1/month	1.40	1.00
Year of birth	(Ref: 1998-2005; age: 18-24)		
	1988-1998; age 25-34	1.20	1.00
	1978-1988; age 35-44	1.48	1.20
	1968-1978; age 45-54	1.17	1.00
	1958-1968; age 55-64	1.13	1.00
	1948-1957; age 65-74	1.24	1.00
	1938-1948; age 75-84	1.38	1.00
	1938 or earlier; 85 or older	1.70	1.00
Gender	(Ref: Male)		
	Female	1.49	1.36
	Other	1.65	1.42
Religious affiliation	(Ref: Christianity)		
	Islam	1.30	1.00
	Collapsed affiliations with prevalence<3%	1.18	1.00
Race/ethnicity	(Ref: Plurality group)		
	Non-plurality groups	1.12	1.00

117. Table S117. Childhood predictors regression for Philippines

Variable	Category	Est	95% CI	SE	Global p-value
Relationship with mother	(Ref: Very bad/somewhat bad)				0.462
	Very good/somewhat good	-0.26	(-0.98, 0.46)	0.37	
Relationship with father	(Ref: Very bad/somewhat bad)				0.380
	Very good/somewhat good	-0.23	(-0.80, 0.33)	0.29	
Parent marital status	(Ref: Parents married)				0.066
	Divorced	-0.26	(-0.91, 0.40)	0.33	
	Parents were never married	0.54	(0.05, 1.03)	0.25	
	One or both parents had died	0.76	(-0.90, 2.42)	0.85	
Subjective financial status of family growing up	(Ref: Got by)				0.015
	Lived comfortably	-0.08	(-0.45, 0.30)	0.19	
	Found it difficult	-0.57	(-0.97, -0.18)	0.20	
	Found it very difficult	0.44	(-0.38, 1.25)	0.42	
Abuse	(Ref: No)				0.532
	Yes	0.28	(-0.60, 1.16)	0.45	
Outsider growing up	(Ref: No)				0.036
	Yes	0.68	(0.05, 1.31)	0.32	
Self-rated health growing up	(Ref: Good)				0.327
	Excellent	0.05	(-0.37, 0.47)	0.21	
	Very good	0.22	(-0.46, 0.91)	0.35	
	Fair	0.41	(0.02, 0.80)	0.20	
	Poor	0.27	(-0.35, 0.89)	0.32	
Immigration status	(Ref: Born in this country)				0.438
	Born in another country	1.21	(-1.85, 4.27)	1.56	
Age 12 religious service attendance	(Ref: Never)				0.049
	At least 1/week	-0.95	(-2.02, 0.12)	0.54	
	1-3/month	-0.69	(-1.76, 0.37)	0.54	
	< 1/month	-0.48	(-1.54, 0.57)	0.54	
Year of birth	(Ref: 1998-2005; age: 18-24)				1.58e-06
	1988-1998; age 25-34	0.80	(0.32, 1.27)	0.24	
	1978-1988; age 35-44	1.32	(0.78, 1.85)	0.27	
	1968-1978; age 45-54	1.00	(0.50, 1.49)	0.25	
	1958-1968; age 55-64	0.93	(0.33, 1.53)	0.31	
	1948-1957; age 65-74	0.35	(-0.39, 1.08)	0.38	
	1938-1948; age 75-84	0.77	(-0.76, 2.29)	0.78	
	1938 or earlier; 85 or older	-1.14	(-2.32, 0.03)	0.60	
Gender	(Ref: Male)				0.000
	Female	-2.90	(-3.24, -2.57)	0.17	
	Other	-2.96	(-3.64, -2.27)	0.35	
Religious affiliation	(Ref: Christianity)				0.431
	Islam	0.70	(-0.46, 1.86)	0.59	
	Collapsed affiliations with prevalence<3%	-0.38	(-1.93, 1.17)	0.79	
Race/ethnicity	(Ref: Plurality group)				0.040
	Non-plurality groups	-0.37	(-0.74, -0.00)	0.19	

118. Table S118. Sensitivity to unmeasured confounding of childhood predictors in Philippines

Variable	Category	E-value for Estimate	E-value for 95% CI
Relationship with mother	(Ref: Very bad/somewhat bad)		
	Very good/somewhat good	1.29	1.00
Relationship with father	(Ref: Very bad/somewhat bad)		
	Very good/somewhat good	1.27	1.00
Parent marital status	(Ref: Parents married)		
	Divorced	1.29	1.00
	Parents were never married	1.47	1.11
	One or both parents had died	1.60	1.00
Subjective financial status of family growing up	(Ref: Got by)		
	Lived comfortably	1.14	1.00
	Found it difficult	1.49	1.23
	Found it very difficult	1.41	1.00
Abuse	(Ref: No)		
	Yes	1.30	1.00
Outsider growing up	(Ref: No)		
	Yes	1.55	1.11
Self-rated health growing up	(Ref: Good)		
	Excellent	1.11	1.00
	Very good	1.26	1.00
	Fair	1.39	1.07
	Poor	1.30	1.00
Immigration status	(Ref: Born in this country)		
	Born in another country	1.87	1.00
Age 12 religious service attendance	(Ref: Never)		
	At least 1/week	1.71	1.00
	1-3/month	1.56	1.00
	< 1/month	1.44	1.00
Year of birth	(Ref: 1998-2005; age: 18-24)		
	1988-1998; age 25-34	1.62	1.33
	1978-1988; age 35-44	1.93	1.61
	1968-1978; age 45-54	1.74	1.45
	1958-1968; age 55-64	1.70	1.34
	1948-1957; age 65-74	1.35	1.00
	1938-1948; age 75-84	1.61	1.00
	1938 or earlier; 85 or older	1.83	1.00
Gender	(Ref: Male)		
	Female	2.98	2.73
	Other	3.02	2.53
Religious affiliation	(Ref: Christianity)		
	Islam	1.57	1.00
	Collapsed affiliations with prevalence<3%	1.37	1.00
Race/ethnicity	(Ref: Plurality group)		
	Non-plurality groups	1.37	1.03

119. Table S119. Childhood predictors regression for Poland

Variable	Category	Est	95% CI	SE	Global p-value
Relationship with mother	(Ref: Very bad/somewhat bad)				0.059
	Very good/somewhat good	-1.51	(-3.09, 0.07)	0.80	
Relationship with father	(Ref: Very bad/somewhat bad)				0.131
	Very good/somewhat good	-0.65	(-1.55, 0.25)	0.46	
Parent marital status	(Ref: Parents married)				9.41e-05
	Divorced	1.40	(0.71, 2.09)	0.35	
	Parents were never married	1.66	(0.30, 3.02)	0.69	
	One or both parents had died	0.21	(-1.66, 2.08)	0.91	
Subjective financial status of family growing up	(Ref: Got by)				0.126
	Lived comfortably	0.25	(-0.25, 0.74)	0.25	
	Found it difficult	0.29	(-0.49, 1.06)	0.40	
	Found it very difficult	1.38	(0.18, 2.58)	0.61	
Abuse	(Ref: No)				0.327
	Yes	0.57	(-0.59, 1.73)	0.59	
Outsider growing up	(Ref: No)				0.594
	Yes	-0.21	(-1.18, 0.76)	0.50	
Self-rated health growing up	(Ref: Good)				0.013
	Excellent	0.33	(-0.35, 1.02)	0.35	
	Very good	-0.50	(-1.15, 0.15)	0.33	
	Fair	-0.43	(-1.60, 0.74)	0.59	
	Poor	-0.83	(-2.23, 0.57)	0.72	
Immigration status	(Ref: Born in this country)				0.253
	Born in another country	1.50	(-1.08, 4.07)	1.31	
Age 12 religious service attendance	(Ref: Never)				0.001
	At least 1/week	-2.07	(-3.33, -0.81)	0.64	
	1-3/month	-1.47	(-2.79, -0.15)	0.67	
	< 1/month	-1.30	(-2.59, -0.01)	0.66	
Year of birth	(Ref: 1998-2005; age: 18-24)				7.02e-12
	1988-1998; age 25-34	1.18	(0.58, 1.78)	0.31	
	1978-1988; age 35-44	1.58	(0.96, 2.21)	0.32	
	1968-1978; age 45-54	1.91	(1.23, 2.59)	0.35	
	1958-1968; age 55-64	2.80	(1.89, 3.72)	0.46	
	1948-1957; age 65-74	0.47	(-0.23, 1.17)	0.36	
	1938-1948; age 75-84	-0.76	(-1.82, 0.31)	0.54	
	1938 or earlier; 85 or older	-0.89	(-3.00, 1.21)	1.07	
Gender	(Ref: Male)				0.000
	Female	-2.82	(-3.27, -2.36)	0.23	
	Other	1.40	(0.66, 2.15)	0.38	
Religious affiliation	(Ref: No religion/Atheist/Agnostic)				0.317
	Christianity	0.02	(-1.03, 1.08)	0.53	
	Collapsed affiliations with prevalence<3%	-1.99	(-4.87, 0.88)	1.47	
Race/ethnicity	(Ref: Plurality group)				0.241
	Non-plurality groups	2.50	(-1.68, 6.69)	2.14	

120. Table S120. Sensitivity to unmeasured confounding of childhood predictors in Poland

Variable	Category	E-value for Estimate	E-value for 95% CI
Relationship with mother	(Ref: Very bad/somewhat bad)		
	Very good/somewhat good	1.73	1.00
Relationship with father	(Ref: Very bad/somewhat bad)		
	Very good/somewhat good	1.40	1.00
Parent marital status	(Ref: Parents married)		
	Divorced	1.69	1.42
	Parents were never married	1.79	1.25
	One or both parents had died	1.20	1.00
Subjective financial status of family growing up	(Ref: Got by)		
	Lived comfortably	1.22	1.00
	Found it difficult	1.24	1.00
	Found it very difficult	1.68	1.18
Abuse	(Ref: No)		
	Yes	1.37	1.00
Outsider growing up	(Ref: No)		
	Yes	1.20	1.00
Self-rated health growing up	(Ref: Good)		
	Excellent	1.26	1.00
	Very good	1.34	1.00
	Fair	1.31	1.00
	Poor	1.47	1.00
Immigration status	(Ref: Born in this country)		
	Born in another country	1.73	1.00
Age 12 religious service attendance	(Ref: Never)		
	At least 1/week	1.95	1.46
	1-3/month	1.72	1.16
	< 1/month	1.65	1.04
Year of birth	(Ref: 1998-2005; age: 18-24)		
	1988-1998; age 25-34	1.61	1.37
	1978-1988; age 35-44	1.76	1.52
	1968-1978; age 45-54	1.89	1.63
	1958-1968; age 55-64	2.24	1.88
	1948-1957; age 65-74	1.32	1.00
	1938-1948; age 75-84	1.44	1.00
	1938 or earlier; 85 or older	1.50	1.00
Gender	(Ref: Male)		
	Female	2.25	2.06
	Other	1.69	1.41
Religious affiliation	(Ref: No religion/Atheist/Agnostic)		
	Christianity	1.06	1.00
	Collapsed affiliations with prevalence<3%	1.92	1.00
Race/ethnicity	(Ref: Plurality group)		
	Non-plurality groups	2.12	1.00

121. Table S121. Childhood predictors regression for South Africa

Variable	Category	Est	95% CI	SE	Global p-value
Relationship with mother	(Ref: Very bad/somewhat bad)				0.306
	Very good/somewhat good	-1.93	(-5.63, 1.76)	1.88	
Relationship with father	(Ref: Very bad/somewhat bad)				0.138
	Very good/somewhat good	-1.03	(-2.37, 0.32)	0.69	
Parent marital status	(Ref: Parents married)				0.364
	Divorced	-0.77	(-1.95, 0.41)	0.60	
	Parents were never married	-0.70	(-1.50, 0.11)	0.41	
	One or both parents had died	-0.26	(-1.22, 0.69)	0.49	
Subjective financial status of family growing up	(Ref: Got by)				0.489
	Lived comfortably	0.03	(-0.40, 0.46)	0.22	
	Found it difficult	1.07	(-0.35, 2.49)	0.72	
	Found it very difficult	-0.50	(-1.40, 0.39)	0.46	
Abuse	(Ref: No)				0.133
	Yes	0.87	(-0.27, 2.00)	0.58	
Outsider growing up	(Ref: No)				0.150
	Yes	0.60	(-0.22, 1.41)	0.42	
Self-rated health growing up	(Ref: Good)				0.041
	Excellent	-0.17	(-0.75, 0.42)	0.30	
	Very good	0.26	(-0.87, 1.39)	0.58	
	Fair	-0.18	(-0.98, 0.61)	0.41	
	Poor	-1.04	(-1.79, -0.29)	0.38	
Immigration status	(Ref: Born in this country)				0.023
	Born in another country	-0.99	(-1.84, -0.14)	0.43	
Age 12 religious service attendance	(Ref: Never)				0.120
	At least 1/week	-0.35	(-1.55, 0.84)	0.61	
	1-3/month	-0.70	(-1.82, 0.42)	0.57	
	< 1/month	-0.97	(-2.12, 0.18)	0.59	
Year of birth	(Ref: 1998-2005; age: 18-24)				0.004
	1988-1998; age 25-34	0.49	(0.01, 0.98)	0.25	
	1978-1988; age 35-44	1.28	(0.32, 2.24)	0.49	
	1968-1978; age 45-54	0.73	(0.05, 1.41)	0.35	
	1958-1968; age 55-64	0.57	(-0.38, 1.51)	0.48	
	1948-1957; age 65-74	-0.53	(-1.22, 0.15)	0.35	
	1938-1948; age 75-84	0.02	(-0.99, 1.04)	0.52	
	1938 or earlier; 85 or older	-0.04	(-1.43, 1.35)	0.71	
Gender	(Ref: Male)				2.34e-08
	Female	-1.57	(-2.07, -1.07)	0.26	
	Other	-0.71	(-3.53, 2.11)	1.44	
Religious affiliation	(Ref: No religion/Atheist/Agnostic)				0.785
	Primal, Animist, or Folk religion	-0.07	(-1.93, 1.78)	0.95	
	Christianity	0.27	(-1.54, 2.09)	0.92	
	Collapsed affiliations with prevalence<3%	0.06	(-2.31, 2.43)	1.21	
Race/ethnicity	(Ref: Plurality group)				0.001
	Non-plurality groups	3.60	(1.43, 5.77)	1.11	

122. Table S122. Sensitivity to unmeasured confounding of childhood predictors in South Africa

Variable	Category	E-value for Estimate	E-value for 95% CI
Relationship with mother	(Ref: Very bad/somewhat bad)		
	Very good/somewhat good	2.30	1.00
Relationship with father	(Ref: Very bad/somewhat bad)		
	Very good/somewhat good	1.75	1.00
Parent marital status	(Ref: Parents married)		
	Divorced	1.60	1.00
	Parents were never married	1.56	1.00
	One or both parents had died	1.29	1.00
Subjective financial status of family growing up	(Ref: Got by)		
	Lived comfortably	1.09	1.00
	Found it difficult	1.78	1.00
	Found it very difficult	1.45	1.00
Abuse	(Ref: No)		
	Yes	1.66	1.00
Outsider growing up	(Ref: No)		
	Yes	1.50	1.00
Self-rated health growing up	(Ref: Good)		
	Excellent	1.22	1.00
	Very good	1.29	1.00
	Fair	1.23	1.00
	Poor	1.76	1.31
Immigration status	(Ref: Born in this country)		
	Born in another country	1.73	1.20
Age 12 religious service attendance	(Ref: Never)		
	At least 1/week	1.35	1.00
	1-3/month	1.56	1.00
	< 1/month	1.72	1.00
Year of birth	(Ref: 1998-2005; age: 18-24)		
	1988-1998; age 25-34	1.44	1.04
	1978-1988; age 35-44	1.90	1.33
	1968-1978; age 45-54	1.58	1.11
	1958-1968; age 55-64	1.48	1.00
	1948-1957; age 65-74	1.47	1.00
	1938-1948; age 75-84	1.07	1.00
	1938 or earlier; 85 or older	1.10	1.00
Gender	(Ref: Male)		
	Female	2.07	1.78
	Other	1.57	1.00
Religious affiliation	(Ref: No religion/Atheist/Agnostic)		
	Primal, Animist, or Folk religion	1.14	1.00
	Christianity	1.30	1.00
	Collapsed affiliations with prevalence<3%	1.13	1.00
Race/ethnicity	(Ref: Plurality group)		
	Non-plurality groups	3.51	1.99

123. Table S123. Childhood predictors regression for Spain

Variable	Category	Est	95% CI	SE	Global p-value
Relationship with mother	(Ref: Very bad/somewhat bad)				0.106
	Very good/somewhat good	-0.76	(-1.71, 0.20)	0.49	
Relationship with father	(Ref: Very bad/somewhat bad)				0.214
	Very good/somewhat good	-0.47	(-1.57, 0.62)	0.53	
Parent marital status	(Ref: Parents married)				0.162
	Divorced	0.07	(-0.72, 0.86)	0.40	
	Parents were never married	-0.76	(-1.48, -0.04)	0.37	
	One or both parents had died	-0.35	(-1.69, 1.00)	0.69	
Subjective financial status of family growing up	(Ref: Got by)				0.520
	Lived comfortably	0.37	(-0.18, 0.92)	0.28	
	Found it difficult	0.18	(-0.45, 0.82)	0.32	
	Found it very difficult	0.79	(-1.20, 2.78)	1.01	
Abuse	(Ref: No)				0.014
	Yes	1.07	(0.19, 1.94)	0.45	
Outsider growing up	(Ref: No)				0.282
	Yes	0.39	(-0.38, 1.15)	0.39	
Self-rated health growing up	(Ref: Good)				0.442
	Excellent	-0.40	(-1.07, 0.27)	0.34	
	Very good	-0.29	(-0.94, 0.36)	0.33	
	Fair	-0.68	(-2.09, 0.72)	0.71	
	Poor	-1.07	(-2.94, 0.80)	0.95	
Immigration status	(Ref: Born in this country)				2.66e-08
	Born in another country	-1.43	(-1.94, -0.92)	0.26	
Age 12 religious service attendance	(Ref: Never)				0.143
	At least 1/week	-0.64	(-1.26, -0.02)	0.32	
	1-3/month	-0.69	(-1.32, -0.05)	0.32	
	< 1/month	-0.42	(-1.10, 0.26)	0.35	
Year of birth	(Ref: 1998-2005; age: 18-24)				0.000
	1988-1998; age 25-34	1.21	(0.60, 1.81)	0.31	
	1978-1988; age 35-44	2.48	(1.86, 3.11)	0.32	
	1968-1978; age 45-54	2.35	(1.74, 2.96)	0.31	
	1958-1968; age 55-64	2.33	(1.59, 3.07)	0.38	
	1948-1957; age 65-74	1.46	(0.49, 2.43)	0.49	
	1938-1948; age 75-84	-0.03	(-1.10, 1.04)	0.55	
	1938 or earlier; 85 or older	3.85	(-0.42, 8.11)	2.18	
Gender	(Ref: Male)				1.24e-07
	Female	-1.25	(-1.71, -0.80)	0.23	
	Other	1.09	(-2.23, 4.41)	1.69	
Religious affiliation	(Ref: No religion/Atheist/Agnostic)				0.968
	Christianity	0.00	(-0.63, 0.64)	0.33	
	Collapsed affiliations with prevalence<3%	0.08	(-1.01, 1.17)	0.55	
Race/ethnicity	(Ref: Plurality group)				

124. Table S124. Sensitivity to unmeasured confounding of childhood predictors in Spain

Variable	Category	E-value for Estimate	E-value for 95% CI
Relationship with mother	(Ref: Very bad/somewhat bad)		
	Very good/somewhat good	1.45	1.00
Relationship with father	(Ref: Very bad/somewhat bad)		
	Very good/somewhat good	1.33	1.00
Parent marital status	(Ref: Parents married)		
	Divorced	1.11	1.00
	Parents were never married	1.45	1.09
	One or both parents had died	1.27	1.00
Subjective financial status of family growing up	(Ref: Got by)		
	Lived comfortably	1.28	1.00
	Found it difficult	1.18	1.00
	Found it very difficult	1.46	1.00
Abuse	(Ref: No)		
	Yes	1.57	1.19
Outsider growing up	(Ref: No)		
	Yes	1.29	1.00
Self-rated health growing up	(Ref: Good)		
	Excellent	1.30	1.00
	Very good	1.24	1.00
	Fair	1.42	1.00
	Poor	1.57	1.00
Immigration status	(Ref: Born in this country)		
	Born in another country	1.72	1.51
Age 12 religious service attendance	(Ref: Never)		
	At least 1/week	1.40	1.05
	1-3/month	1.42	1.09
	< 1/month	1.30	1.00
Year of birth	(Ref: 1998-2005; age: 18-24)		
	1988-1998; age 25-34	1.63	1.39
	1978-1988; age 35-44	2.13	1.88
	1968-1978; age 45-54	2.08	1.84
	1958-1968; age 55-64	2.07	1.78
	1948-1957; age 65-74	1.73	1.34
	1938-1948; age 75-84	1.07	1.00
	1938 or earlier; 85 or older	2.73	1.00
Gender	(Ref: Male)		
	Female	1.65	1.47
	Other	1.58	1.00
Religious affiliation	(Ref: No religion/Atheist/Agnostic)		
	Christianity	1.03	1.00
	Collapsed affiliations with prevalence<3%	1.11	1.00
Race/ethnicity	(Ref: Plurality group)		

125. Table S125. Childhood predictors regression for Sweden

Variable	Category	Est	95% CI	SE	Global p-value
Relationship with mother	(Ref: Very bad/somewhat bad)				0.107
	Very good/somewhat good	-0.22	(-0.49, 0.05)	0.14	
Relationship with father	(Ref: Very bad/somewhat bad)				0.235
	Very good/somewhat good	0.12	(-0.08, 0.32)	0.10	
Parent marital status	(Ref: Parents married)				5.08e-05
	Divorced	0.43	(0.19, 0.67)	0.12	
	Parents were never married	0.42	(0.20, 0.63)	0.11	
	One or both parents had died	0.41	(-0.12, 0.95)	0.27	
Subjective financial status of family growing up	(Ref: Got by)				0.210
	Lived comfortably	-0.10	(-0.23, 0.04)	0.07	
	Found it difficult	0.12	(-0.15, 0.39)	0.14	
	Found it very difficult	0.46	(-0.42, 1.34)	0.45	
Abuse	(Ref: No)				0.051
	Yes	0.22	(-0.00, 0.43)	0.11	
Outsider growing up	(Ref: No)				0.006
	Yes	0.34	(0.09, 0.58)	0.12	
Self-rated health growing up	(Ref: Good)				0.138
	Excellent	0.18	(-0.00, 0.37)	0.10	
	Very good	0.11	(-0.08, 0.29)	0.09	
	Fair	0.30	(-0.03, 0.62)	0.17	
	Poor	0.37	(-0.12, 0.86)	0.25	
Immigration status	(Ref: Born in this country)				0.093
	Born in another country	0.28	(-0.05, 0.62)	0.17	
Age 12 religious service attendance	(Ref: Never)				0.863
	At least 1/week	0.00	(-0.25, 0.26)	0.13	
	1-3/month	0.09	(-0.15, 0.34)	0.13	
	< 1/month	0.04	(-0.11, 0.18)	0.08	
Year of birth	(Ref: 1998-2005; age: 18-24)				0.000
	1988-1998; age 25-34	0.32	(0.11, 0.52)	0.11	
	1978-1988; age 35-44	0.50	(0.27, 0.72)	0.11	
	1968-1978; age 45-54	0.61	(0.38, 0.85)	0.12	
	1958-1968; age 55-64	0.75	(0.52, 0.99)	0.12	
	1948-1957; age 65-74	0.76	(0.49, 1.03)	0.14	
	1938-1948; age 75-84	0.18	(-0.03, 0.40)	0.11	
	1938 or earlier; 85 or older	-0.08	(-0.48, 0.32)	0.19	
Gender	(Ref: Male)				0.219
	Female	0.12	(-0.02, 0.25)	0.07	
	Other	0.21	(-1.33, 1.75)	0.78	
Religious affiliation	(Ref: No religion/Atheist/Agnostic)				0.012
	Islam	1.19	(0.45, 1.94)	0.38	
	Christianity	-0.02	(-0.17, 0.13)	0.08	
	Collapsed affiliations with prevalence<3%	0.12	(-0.45, 0.69)	0.29	
Race/ethnicity	(Ref: Plurality group)				

126. Table S126. Sensitivity to unmeasured confounding of childhood predictors in Sweden

Variable	Category	E-value for Estimate	E-value for 95% CI
Relationship with mother	(Ref: Very bad/somewhat bad)		
	Very good/somewhat good	1.32	1.00
Relationship with father	(Ref: Very bad/somewhat bad)		
	Very good/somewhat good	1.22	1.00
Parent marital status	(Ref: Parents married)		
	Divorced	1.50	1.29
	Parents were never married	1.49	1.30
	One or both parents had died	1.49	1.00
Subjective financial status of family growing up	(Ref: Got by)		
	Lived comfortably	1.19	1.00
	Found it difficult	1.22	1.00
	Found it very difficult	1.53	1.00
Abuse	(Ref: No)		
	Yes	1.32	1.00
Outsider growing up	(Ref: No)		
	Yes	1.42	1.19
Self-rated health growing up	(Ref: Good)		
	Excellent	1.28	1.00
	Very good	1.20	1.00
	Fair	1.39	1.00
	Poor	1.45	1.00
Immigration status	(Ref: Born in this country)		
	Born in another country	1.38	1.00
Age 12 religious service attendance	(Ref: Never)		
	At least 1/week	1.03	1.00
	1-3/month	1.19	1.00
	< 1/month	1.11	1.00
Year of birth	(Ref: 1998-2005; age: 18-24)		
	1988-1998; age 25-34	1.40	1.21
	1978-1988; age 35-44	1.56	1.37
	1968-1978; age 45-54	1.65	1.46
	1958-1968; age 55-64	1.76	1.57
	1948-1957; age 65-74	1.76	1.55
	1938-1948; age 75-84	1.28	1.00
	1938 or earlier; 85 or older	1.17	1.00
Gender	(Ref: Male)		
	Female	1.22	1.00
	Other	1.31	1.00
Religious affiliation	(Ref: No religion/Atheist/Agnostic)		
	Islam	2.12	1.51
	Christianity	1.08	1.00
	Collapsed affiliations with prevalence<3%	1.22	1.00
Race/ethnicity	(Ref: Plurality group)		

127. Table S127. Childhood predictors regression for Tanzania

Variable	Category	Est	95% CI	SE	Global p-value
Relationship with mother	(Ref: Very bad/somewhat bad)				0.805
	Very good/somewhat good	0.01	(-0.13, 0.16)	0.08	
Relationship with father	(Ref: Very bad/somewhat bad)				0.862
	Very good/somewhat good	0.01	(-0.21, 0.22)	0.11	
Parent marital status	(Ref: Parents married)				0.671
	Divorced	0.04	(-0.35, 0.44)	0.19	
	Parents were never married	0.07	(-0.06, 0.20)	0.07	
	One or both parents had died	0.01	(-0.15, 0.17)	0.08	
Subjective financial status of family growing up	(Ref: Got by)				0.945
	Lived comfortably	-0.00	(-0.10, 0.09)	0.05	
	Found it difficult	-0.04	(-0.18, 0.10)	0.07	
	Found it very difficult	-0.02	(-0.22, 0.17)	0.10	
Abuse	(Ref: No)				0.297
	Yes	0.07	(-0.07, 0.21)	0.07	
Outsider growing up	(Ref: No)				0.681
	Yes	0.04	(-0.13, 0.21)	0.09	
Self-rated health growing up	(Ref: Good)				0.024
	Excellent	-0.04	(-0.15, 0.08)	0.06	
	Very good	-0.11	(-0.24, 0.02)	0.07	
	Fair	0.04	(-0.12, 0.20)	0.08	
	Poor	-0.17	(-0.31, -0.04)	0.07	
Immigration status	(Ref: Born in this country)				0.379
	Born in another country	-0.11	(-0.37, 0.14)	0.13	
Age 12 religious service attendance	(Ref: Never)				0.103
	At least 1/week	0.12	(-0.01, 0.25)	0.06	
	1-3/month	0.18	(-0.01, 0.36)	0.09	
	< 1/month	0.13	(-0.06, 0.32)	0.10	
Year of birth	(Ref: 1998-2005; age: 18-24)				1.51e-05
	1988-1998; age 25-34	0.13	(0.05, 0.22)	0.04	
	1978-1988; age 35-44	0.22	(0.11, 0.34)	0.06	
	1968-1978; age 45-54	0.23	(0.12, 0.33)	0.05	
	1958-1968; age 55-64	0.44	(0.05, 0.84)	0.20	
	1948-1957; age 65-74	0.16	(-0.01, 0.32)	0.09	
	1938-1948; age 75-84	0.19	(-0.23, 0.61)	0.22	
	1938 or earlier; 85 or older	-0.06	(-0.19, 0.06)	0.06	
Gender	(Ref: Male)				7.11e-15
	Female	-0.38	(-0.47, -0.29)	0.05	
Religious affiliation	(Ref: No religion/Atheist/Agnostic)				6.04e-07
	Islam	0.21	(0.04, 0.39)	0.09	
	Christianity	0.01	(-0.13, 0.15)	0.07	
	Collapsed affiliations with prevalence<3%	-0.31	(-0.48, -0.14)	0.09	
Race/ethnicity	(Ref: Plurality group)				1.45e-04
	Non-plurality groups	-0.31	(-0.46, -0.16)	0.08	

128. Table S128. Sensitivity to unmeasured confounding of childhood predictors in Tanzania

Variable	Category	E-value for Estimate	E-value for 95% CI
Relationship with mother	(Ref: Very bad/somewhat bad)		
	Very good/somewhat good	1.09	1.00
Relationship with father	(Ref: Very bad/somewhat bad)		
	Very good/somewhat good	1.06	1.00
Parent marital status	(Ref: Parents married)		
	Divorced	1.17	1.00
	Parents were never married	1.22	1.00
	One or both parents had died	1.08	1.00
Subjective financial status of family growing up	(Ref: Got by)		
	Lived comfortably	1.04	1.00
	Found it difficult	1.15	1.00
	Found it very difficult	1.12	1.00
Abuse	(Ref: No)		
	Yes	1.24	1.00
Outsider growing up	(Ref: No)		
	Yes	1.15	1.00
Self-rated health growing up	(Ref: Good)		
	Excellent	1.15	1.00
	Very good	1.30	1.00
	Fair	1.16	1.00
	Poor	1.41	1.16
Immigration status	(Ref: Born in this country)		
	Born in another country	1.31	1.00
Age 12 religious service attendance	(Ref: Never)		
	At least 1/week	1.32	1.00
	1-3/month	1.41	1.00
	< 1/month	1.34	1.00
Year of birth	(Ref: 1998-2005; age: 18-24)		
	1988-1998; age 25-34	1.34	1.18
	1978-1988; age 35-44	1.49	1.31
	1968-1978; age 45-54	1.49	1.33
	1958-1968; age 55-64	1.81	1.18
	1948-1957; age 65-74	1.38	1.00
	1938-1948; age 75-84	1.44	1.00
	1938 or earlier; 85 or older	1.21	1.00
Gender	(Ref: Male)		
	Female	1.72	1.59
Religious affiliation	(Ref: No religion/Atheist/Agnostic)		
	Islam	1.47	1.17
	Christianity	1.07	1.00
	Collapsed affiliations with prevalence<3%	1.62	1.36
Race/ethnicity	(Ref: Plurality group)		
	Non-plurality groups	1.62	1.39

129. Table S129. Childhood predictors regression for Türkiye

Variable	Category	Est	95% CI	SE	Global p-value
Relationship with mother	(Ref: Very bad/somewhat bad)				0.292
	Very good/somewhat good	1.71	(-1.51, 4.94)	1.65	
Relationship with father	(Ref: Very bad/somewhat bad)				0.011
	Very good/somewhat good	-3.40	(-6.04, -0.76)	1.35	
Parent marital status	(Ref: Parents married)				0.319
	Divorced	3.21	(-0.56, 6.97)	1.92	
	Parents were never married	-1.93	(-8.08, 4.22)	3.13	
	One or both parents had died	-0.77	(-5.15, 3.62)	2.23	
Subjective financial status of family growing up	(Ref: Got by)				0.662
	Lived comfortably	0.40	(-1.20, 2.00)	0.81	
	Found it difficult	-1.15	(-3.67, 1.37)	1.28	
	Found it very difficult	-0.70	(-4.29, 2.89)	1.83	
Abuse	(Ref: No)				0.199
	Yes	1.57	(-0.87, 4.01)	1.24	
Outsider growing up	(Ref: No)				0.198
	Yes	1.77	(-0.94, 4.48)	1.38	
Self-rated health growing up	(Ref: Good)				0.151
	Excellent	-1.31	(-3.39, 0.78)	1.06	
	Very good	-2.04	(-4.05, -0.03)	1.03	
	Fair	-1.36	(-3.96, 1.25)	1.33	
	Poor	3.72	(-2.65, 10.09)	3.25	
Immigration status	(Ref: Born in this country)				0.768
	Born in another country	0.71	(-4.07, 5.49)	2.44	
Age 12 religious service attendance	(Ref: Never)				0.306
	At least 1/week	1.36	(-0.65, 3.36)	1.02	
	1-3/month	0.36	(-1.88, 2.61)	1.14	
	< 1/month	-0.59	(-2.64, 1.46)	1.04	
Year of birth	(Ref: 1998-2005; age: 18-24)				8.49e-07
	1988-1998; age 25-34	2.22	(-0.03, 4.46)	1.15	
	1978-1988; age 35-44	2.23	(-0.00, 4.46)	1.14	
	1968-1978; age 45-54	2.99	(0.47, 5.51)	1.28	
	1958-1968; age 55-64	1.42	(-1.38, 4.22)	1.43	
	1948-1957; age 65-74	-0.64	(-4.48, 3.21)	1.96	
	1938-1948; age 75-84	-3.81	(-7.88, 0.25)	2.07	
	1938 or earlier; 85 or older	8.64	(5.58, 11.71)	1.56	
Gender	(Ref: Male)				0.000
	Female	-7.71	(-9.29, -6.12)	0.81	
Religious affiliation	(Ref: Islam)				0.804
	Collapsed affiliations with prevalence<3%	-0.50	(-4.68, 3.67)	2.13	
Race/ethnicity	(Ref: Plurality group)				0.154
	Non-plurality groups	1.32	(-0.56, 3.21)	0.96	

130. Table S130. Sensitivity to unmeasured confounding of childhood predictors in Türkiye

Variable	Category	E-value for Estimate	E-value for 95% CI
Relationship with mother	(Ref: Very bad/somewhat bad)		
	Very good/somewhat good	1.50	1.00
Relationship with father	(Ref: Very bad/somewhat bad)		
	Very good/somewhat good	1.84	1.29
Parent marital status	(Ref: Parents married)		
	Divorced	1.80	1.00
	Parents were never married	1.54	1.00
	One or both parents had died	1.29	1.00
Subjective financial status of family growing up	(Ref: Got by)		
	Lived comfortably	1.20	1.00
	Found it difficult	1.38	1.00
	Found it very difficult	1.28	1.00
Abuse	(Ref: No)		
	Yes	1.47	1.00
Outsider growing up	(Ref: No)		
	Yes	1.51	1.00
Self-rated health growing up	(Ref: Good)		
	Excellent	1.41	1.00
	Very good	1.57	1.05
	Fair	1.42	1.00
	Poor	1.90	1.00
Immigration status	(Ref: Born in this country)		
	Born in another country	1.28	1.00
Age 12 religious service attendance	(Ref: Never)		
	At least 1/week	1.43	1.00
	1-3/month	1.19	1.00
	< 1/month	1.25	1.00
Year of birth	(Ref: 1998-2005; age: 18-24)		
	1988-1998; age 25-34	1.60	1.00
	1978-1988; age 35-44	1.60	1.01
	1968-1978; age 45-54	1.76	1.22
	1958-1968; age 55-64	1.44	1.00
	1948-1957; age 65-74	1.26	1.00
	1938-1948; age 75-84	1.92	1.00
	1938 or earlier; 85 or older	3.02	2.30
Gender	(Ref: Male)		
	Female	2.79	2.42
Religious affiliation	(Ref: Islam)		
	Collapsed affiliations with prevalence<3%	1.23	1.00
Race/ethnicity	(Ref: Plurality group)		
	Non-plurality groups	1.42	1.00

131. Table S131. Childhood predictors regression for United Kingdom

Variable	Category	Est	95% CI	SE	Global p-value
Relationship with mother	(Ref: Very bad/somewhat bad)				0.326
	Very good/somewhat good	-0.37	(-1.12, 0.37)	0.38	
Relationship with father	(Ref: Very bad/somewhat bad)				0.587
	Very good/somewhat good	-0.17	(-0.79, 0.45)	0.32	
Parent marital status	(Ref: Parents married)				0.096
	Divorced	0.59	(-0.18, 1.36)	0.39	
	Parents were never married	0.87	(-0.00, 1.74)	0.44	
	One or both parents had died	0.62	(-0.73, 1.97)	0.69	
Subjective financial status of family growing up	(Ref: Got by)				0.819
	Lived comfortably	0.18	(-0.22, 0.59)	0.21	
	Found it difficult	0.20	(-0.51, 0.90)	0.36	
	Found it very difficult	0.17	(-0.94, 1.28)	0.57	
Abuse	(Ref: No)				0.002
	Yes	0.99	(0.36, 1.62)	0.32	
Outsider growing up	(Ref: No)				0.168
	Yes	0.36	(-0.17, 0.88)	0.27	
Self-rated health growing up	(Ref: Good)				0.468
	Excellent	-0.22	(-0.77, 0.33)	0.28	
	Very good	-0.04	(-0.62, 0.53)	0.29	
	Fair	-0.58	(-1.35, 0.18)	0.39	
	Poor	-0.64	(-1.89, 0.62)	0.64	
Immigration status	(Ref: Born in this country)				0.041
	Born in another country	-0.48	(-0.94, -0.02)	0.24	
Age 12 religious service attendance	(Ref: Never)				0.229
	At least 1/week	-0.45	(-0.99, 0.09)	0.27	
	1-3/month	-0.13	(-0.82, 0.56)	0.35	
	< 1/month	-0.46	(-1.02, 0.10)	0.28	
Year of birth	(Ref: 1998-2005; age: 18-24)				2.22e-16
	1988-1998; age 25-34	1.25	(0.77, 1.73)	0.25	
	1978-1988; age 35-44	1.82	(1.17, 2.48)	0.33	
	1968-1978; age 45-54	1.59	(0.96, 2.22)	0.32	
	1958-1968; age 55-64	1.74	(1.10, 2.38)	0.33	
	1948-1957; age 65-74	1.50	(0.84, 2.17)	0.34	
	1938-1948; age 75-84	0.37	(-0.29, 1.04)	0.34	
	1938 or earlier; 85 or older	-0.06	(-0.73, 0.61)	0.34	
Gender	(Ref: Male)				0.063
	Female	-0.43	(-0.79, -0.07)	0.18	
	Other	-0.16	(-2.42, 2.09)	1.15	
Religious affiliation	(Ref: No religion/Atheist/Agnostic)				0.931
	Islam	0.28	(-0.68, 1.24)	0.49	
	Christianity	-0.01	(-0.54, 0.51)	0.27	
	Collapsed affiliations with prevalence<3%	0.14	(-1.14, 1.43)	0.66	
Race/ethnicity	(Ref: Plurality group)				0.051
	Non-plurality groups	-0.61	(-1.22, 0.01)	0.31	

132. Table S132. Sensitivity to unmeasured confounding of childhood predictors in United Kingdom

Variable	Category	E-value for Estimate	E-value for 95% CI
Relationship with mother	(Ref: Very bad/somewhat bad)		
	Very good/somewhat good	1.34	1.00
Relationship with father	(Ref: Very bad/somewhat bad)		
	Very good/somewhat good	1.21	1.00
Parent marital status	(Ref: Parents married)		
	Divorced	1.46	1.00
	Parents were never married	1.61	1.00
	One or both parents had died	1.48	1.00
Subjective financial status of family growing up	(Ref: Got by)		
	Lived comfortably	1.22	1.00
	Found it difficult	1.23	1.00
	Found it very difficult	1.21	1.00
Abuse	(Ref: No)		
	Yes	1.67	1.33
Outsider growing up	(Ref: No)		
	Yes	1.33	1.00
Self-rated health growing up	(Ref: Good)		
	Excellent	1.24	1.00
	Very good	1.10	1.00
	Fair	1.46	1.00
	Poor	1.49	1.00
Immigration status	(Ref: Born in this country)		
	Born in another country	1.40	1.06
Age 12 religious service attendance	(Ref: Never)		
	At least 1/week	1.38	1.00
	1-3/month	1.18	1.00
	< 1/month	1.39	1.00
Year of birth	(Ref: 1998-2005; age: 18-24)		
	1988-1998; age 25-34	1.81	1.56
	1978-1988; age 35-44	2.12	1.77
	1968-1978; age 45-54	1.99	1.66
	1958-1968; age 55-64	2.07	1.74
	1948-1957; age 65-74	1.94	1.60
	1938-1948; age 75-84	1.34	1.00
	1938 or earlier; 85 or older	1.12	1.00
Gender	(Ref: Male)		
	Female	1.37	1.13
	Other	1.21	1.00
Religious affiliation	(Ref: No religion/Atheist/Agnostic)		
	Islam	1.29	1.00
	Christianity	1.05	1.00
	Collapsed affiliations with prevalence<3%	1.19	1.00
Race/ethnicity	(Ref: Plurality group)		
	Non-plurality groups	1.47	1.00

133. Table S133. Childhood predictors regression for United States

Variable	Category	Est	95% CI	SE	Global p-value
Relationship with mother	(Ref: Very bad/somewhat bad)				0.347
	Very good/somewhat good	-0.22	(-0.67, 0.24)	0.23	
Relationship with father	(Ref: Very bad/somewhat bad)				0.064
	Very good/somewhat good	0.29	(-0.02, 0.59)	0.16	
Parent marital status	(Ref: Parents married)				0.050
	Divorced	0.22	(-0.14, 0.57)	0.18	
	Parents were never married	1.07	(0.14, 2.00)	0.48	
	One or both parents had died	0.36	(-0.35, 1.07)	0.36	
Subjective financial status of family growing up	(Ref: Got by)				0.099
	Lived comfortably	-0.20	(-0.40, 0.00)	0.10	
	Found it difficult	-0.11	(-0.45, 0.23)	0.17	
	Found it very difficult	0.56	(-0.49, 1.61)	0.54	
Abuse	(Ref: No)				2.44e-07
	Yes	0.73	(0.45, 1.01)	0.14	
Outsider growing up	(Ref: No)				0.001
	Yes	0.47	(0.19, 0.76)	0.15	
Self-rated health growing up	(Ref: Good)				0.697
	Excellent	-0.08	(-0.45, 0.28)	0.19	
	Very good	-0.09	(-0.47, 0.29)	0.20	
	Fair	-0.31	(-0.98, 0.36)	0.34	
	Poor	0.86	(-1.02, 2.74)	0.96	
Immigration status	(Ref: Born in this country)				1.57e-12
	Born in another country	-0.84	(-1.07, -0.60)	0.12	
Age 12 religious service attendance	(Ref: Never)				0.033
	At least 1/week	-0.57	(-1.00, -0.14)	0.22	
	1-3/month	-0.45	(-0.90, 0.01)	0.23	
	< 1/month	-0.37	(-0.92, 0.18)	0.28	
Year of birth	(Ref: 1998-2005; age: 18-24)				0.000
	1988-1998; age 25-34	0.79	(0.23, 1.35)	0.29	
	1978-1988; age 35-44	1.02	(0.57, 1.47)	0.23	
	1968-1978; age 45-54	1.09	(0.64, 1.54)	0.23	
	1958-1968; age 55-64	1.41	(0.96, 1.86)	0.23	
	1948-1957; age 65-74	1.16	(0.69, 1.62)	0.24	
	1938-1948; age 75-84	0.47	(0.01, 0.92)	0.23	
	1938 or earlier; 85 or older	0.10	(-0.43, 0.62)	0.27	
Gender	(Ref: Male)				0.007
	Female	-0.26	(-0.47, -0.04)	0.11	
	Other	-0.70	(-1.20, -0.20)	0.26	
Religious affiliation	(Ref: No religion/Atheist/Agnostic)				0.047
	Christianity	-0.15	(-0.67, 0.37)	0.26	
	Collapsed affiliations with prevalence<3%	-0.48	(-1.02, 0.06)	0.28	
Race/ethnicity	(Ref: Plurality group)				8.39e-04
	Non-plurality groups	-0.43	(-0.67, -0.19)	0.12	

134. Table S134. Sensitivity to unmeasured confounding of childhood predictors in United States

Variable	Category	E-value for Estimate	E-value for 95% CI
Relationship with mother	(Ref: Very bad/somewhat bad)		
	Very good/somewhat good	1.26	1.00
Relationship with father	(Ref: Very bad/somewhat bad)		
	Very good/somewhat good	1.31	1.00
Parent marital status	(Ref: Parents married)		
	Divorced	1.26	1.00
	Parents were never married	1.77	1.20
	One or both parents had died	1.35	1.00
Subjective financial status of family growing up	(Ref: Got by)		
	Lived comfortably	1.24	1.00
	Found it difficult	1.17	1.00
	Found it very difficult	1.48	1.00
Abuse	(Ref: No)		
	Yes	1.58	1.41
Outsider growing up	(Ref: No)		
	Yes	1.43	1.23
Self-rated health growing up	(Ref: Good)		
	Excellent	1.15	1.00
	Very good	1.15	1.00
	Fair	1.32	1.00
	Poor	1.65	1.00
Immigration status	(Ref: Born in this country)		
	Born in another country	1.64	1.51
Age 12 religious service attendance	(Ref: Never)		
	At least 1/week	1.49	1.20
	1-3/month	1.41	1.00
	< 1/month	1.36	1.00
Year of birth	(Ref: 1998-2005; age: 18-24)		
	1988-1998; age 25-34	1.62	1.27
	1978-1988; age 35-44	1.75	1.49
	1968-1978; age 45-54	1.79	1.53
	1958-1968; age 55-64	1.97	1.71
	1948-1957; age 65-74	1.83	1.56
	1938-1948; age 75-84	1.42	1.06
	1938 or earlier; 85 or older	1.16	1.00
Gender	(Ref: Male)		
	Female	1.29	1.10
	Other	1.56	1.25
Religious affiliation	(Ref: No religion/Atheist/Agnostic)		
	Christianity	1.21	1.00
	Collapsed affiliations with prevalence<3%	1.43	1.00
Race/ethnicity	(Ref: Plurality group)		
	Non-plurality groups	1.40	1.24

135. Table 135. Random effects meta-analysis of regression of daily cigarette consumption (continuous) on childhood predictors: Total sample (N = 202,898)

Variable	Category	Est	95% CI	SE	Estimated proportion of effects by threshold < -0.10	> 0.10	Heterogeneity (τ)	I^2	Global *p*-value
Relationship with mother	(Ref: Very bad/somewhat bad)								0.141
	Very good/somewhat good	-0.22	(-0.44,-0.00)	0.11	0.64	0.18	0.34	70.5	
Relationship with father	(Ref: Very bad/somewhat bad)								0.082
	Very good/somewhat good	-0.16	(-0.34,0.03)	0.09	0.68	0.18	0.31	65.8	
Parental marital status	(Ref: Parents married)								5.09e-04**
	Divorced	0.32	(0.06,0.59)	0.13	0.23	0.68	0.51	85.0	
	Single, never married	0.15	(-0.06,0.37)	0.11	0.18	0.59	0.36	74.5	
	One or both parents had died	0.11	(0.00,0.21)	0.05	0.00	1.00	<.01ǂ	<0.1ǂ	
Subjective financial status of family growing up	(Ref: Got by)								0.027*
	Lived comfortably	-0.05	(-0.10,0.00)	0.03	0.00	0.00	<.01ǂ	<0.1ǂ	
	Found it difficult	-0.04	(-0.14,0.05)	0.05	0.23	0.09	0.11	31.2	
	Found it very difficult	0.10	(-0.10,0.31)	0.10	0.18	0.55	0.29	59.9	
Abuse	(Ref: No)								2.09e-05**
	Yes	0.43	(0.28,0.57)	0.08	0.00	0.95	0.23	61.9	
Outsider growing up	(Ref: No)								2.79e-04**
	Yes	0.32	(0.19,0.45)	0.07	0.00	0.95	0.18	45.9	
Self-rated health growing up	(Ref: Good)								1.26e-04**
	Excellent	0.08	(-0.02,0.18)	0.05	0.00	0.41	0.12	37.1	
	Very good	0.01	(-0.08,0.10)	0.05	0.09	0.14	0.10	30.6	
	Fair	-0.03	(-0.15,0.09)	0.06	0.36	0.18	0.15	35.8	
	Poor	-0.12	(-0.41,0.16)	0.14	0.50	0.36	0.42	75.2	
Immigration status°	(Ref: Born in this country)								1.10e-05**
	Born in another country	-0.41	(-0.75,-0.08)	0.17	0.73	0.23	0.63	86.2	
Age 12 religious service attendance	(Ref: Never)								0.004**
	At least 1/week	-0.16	(-0.33,0.01)	0.09	0.59	0.18	0.26	61.7	
	1-3/month	-0.15	(-0.40,0.11)	0.13	0.50	0.18	0.49	78.3	
	< 1/month	-0.08	(-0.21,0.05)	0.07	0.50	0.14	0.16	35.9	
Age; year of Birth	(Ref: age 18-24; 1998-2005)								1.37e-06**
	age 25-34; 1988-1998	0.75	(0.53,0.97)	0.11	0.00	0.95	0.46	92.8	
	age 35-44; 1978-1988	1.23	(0.86,1.59)	0.19	0.00	1.00	0.81	96.0	
	age 45-54; 1968-1978	1.26	(0.88,1.65)	0.20	0.00	0.95	0.85	95.8	
	age 55-64; 1958-1968	1.34	(0.91,1.76)	0.22	0.00	0.95	0.94	95.1	
	age 65-74; 1948-1957	0.62	(0.29,0.95)	0.17	0.14	0.77	0.69	91.7	
	age 75-84; 1938-1948	-0.10	(-0.49,0.29)	0.20	0.41	0.45	0.79	90.8	
	85 or older; 1938 or earlierǂǂ	-0.07	(-0.95,0.81)	0.45	0.45	0.32	1.94	98.1	
Gender	(Ref: Male)								1.49e-06**
	Female	-2.00	(-2.94,-1.07)	0.48	0.95	0.05	2.23	99.8	
	Otherǂǂ	-1.04	(-1.67,-0.41)	0.32	0.83	0.06	1.04	90.8	

ǂ Estimate of heterogeneity is likely unstable. See our online supplement forest plots for more detail on heterogeneity of effects.
ǂǂ Group is very small (< 0.1% of the observed sample) within several countries leading large uncertainty in this estimate—be cautious about interpreting this estimate; CI = confidence interval; the estimated proportion of effects is the estimated proportion of effects above (or below) a threshold based on the calibrated effect sizes[43]; I^2 is an estimate of the variability in means due to heterogeneity across countries vs. sampling variability; Global *p*-value corresponds to the joint test of the null hypothesis that the country-specific joint parameter Wald tests (all parameters within variable groups are zero) are all null all 22 countries; and additional details of heterogeneity of effects are available in the forest plots of our online supplemental material.
* $p < .05$; ** $p < .004$ (Bonferroni corrected threshold).

136. Table S136. Sensitivity of meta-analyzed childhood predictors to unmeasured confounding.

Variable	Category	evalue	evalue.limit
Relationship with mother	(Ref: Very bad/somewhat bad)		
	Very good/somewhat good	1.24	1.01
Relationship with father	(Ref: Very bad/somewhat bad)		
	Very good/somewhat good	1.20	1.00
Parent marital status	(Ref: Parents married)		
	Divorced	1.30	1.11
	Single, never married	1.19	1.00
	One or both parents had died	1.16	1.02
Subjective financial status of family growing up	(Ref: Got by)		
	Lived comfortably	1.10	1.00
	Found it difficult	1.10	1.00
	Found it very difficult	1.15	1.00
Abuse	(Ref: No)		
	Yes	1.36	1.27
Outsider growing up	(Ref: No)		
	Yes	1.30	1.22
Self-rated health growing up	(Ref: Good)		
	Excellent	1.13	1.00
	Very good	1.05	1.00
	Fair	1.08	1.00
	Poor	1.17	1.00
Immigration status	(Ref: Born in this country)		
	Born in another country	1.35	1.13
Age 12 religious service attendance	(Ref: Never)		
	At least 1/week	1.20	1.00
	1-3/month	1.19	1.00
	< 1/month	1.13	1.00
Year of birth	(Ref: 1998-2005; age 18-24)		
	1988-1998; age 25-34	1.53	1.41
	1978-1988; age 35-44	1.77	1.58
	1968-1978; age 45-54	1.78	1.59
	1958-1968; age 55-64	1.82	1.61
	1948-1957; age 65-74	1.46	1.28
	1938-1948; age 75-84	1.15	1.00
	1938 or earlier; 85 or older	1.12	1.00
Gender	(Ref: Male)		
	Female	2.16	1.69
	Other	1.67	1.35

137. Table S137. Population weighted meta-analysis of regression results (continuous).

Variable	Category	Est	95% CI	SE
Relationship with mother	(Ref: Very bad/somewhat bad)			
	Very good/somewhat good	-0.07	(-0.56,0.43)	0.251
Relationship with father	(Ref: Very bad/somewhat bad)			
	Very good/somewhat good	-0.00	(-0.53,0.52)	0.268
Parent marital status	(Ref: Parents married)			
	Divorced	0.21	(-0.06,0.47)	0.135
	Single, never married	0.13	(-0.15,0.41)	0.145
	One or both parents had died	0.21	(-0.06,0.48)	0.139
Subjective financial status of family growing up	(Ref: Got by)			
	Lived comfortably	-0.00	(-0.11,0.11)	0.057
	Found it difficult	-0.05	(-0.21,0.10)	0.080
	Found it very difficult	0.38	(0.11,0.65)	0.139
Abuse	(Ref: No)			
	Yes	0.59	(0.41,0.77)	0.093
Outsider growing up	(Ref: No)			
	Yes	0.37	(0.19,0.55)	0.090
Self-rated health growing up	(Ref: Good)			
	Excellent	0.04	(-0.10,0.18)	0.070
	Very good	-0.02	(-0.16,0.12)	0.072
	Fair	-0.09	(-0.27,0.09)	0.092
	Poor	0.18	(-0.27,0.63)	0.231
Immigration status	(Ref: Born in this country)			
	Born in another country	0.33	(-0.39,1.05)	0.369
Age 12 religious service attendance	(Ref: Never)			
	At least 1/week	-0.20	(-0.40,-0.01)	0.097
	1-3/month	-0.06	(-0.27,0.14)	0.105
	< 1/month	-0.19	(-0.38,0.01)	0.098
Year of birth	(Ref: 1998-2005; age 18-24)			
	1988-1998; age 25-34	0.67	(0.49,0.84)	0.088
	1978-1988; age 35-44	0.89	(0.74,1.05)	0.080
	1968-1978; age 45-54	1.08	(0.89,1.26)	0.094
	1958-1968; age 55-64	1.08	(0.88,1.28)	0.101
	1948-1957; age 65-74	0.48	(0.25,0.70)	0.115
	1938-1948; age 75-84	-0.24	(-0.68,0.21)	0.227
	1938 or earlier; 85 or older	0.12	(-0.33,0.57)	0.230
Gender	(Ref: Male)			
	Female	-1.83	(-1.93,-1.72)	0.053
	Other	-1.09	(-3.57,1.40)	1.269

138. Table S138. Population weighted meta-analysis of E-values (continuous).

Variable	Category	E-value	E-value limit
Relationship with mother	(Ref: Very bad/somewhat bad)		
	Very good/somewhat good	1.12	1.00
Relationship with father	(Ref: Very bad/somewhat bad)		
	Very good/somewhat good	1.02	1.00
Parent marital status	(Ref: Parents married)		
	Divorced	1.23	1.00
	Single, never married	1.17	1.00
	One or both parents had died	1.23	1.00
Subjective financial status of family growing up	(Ref: Got by)		
	Lived comfortably	1.02	1.00
	Found it difficult	1.11	1.00
	Found it very difficult	1.33	1.15
Abuse	(Ref: No)		
	Yes	1.45	1.35
Outsider growing up	(Ref: No)		
	Yes	1.33	1.22
Self-rated health growing up	(Ref: Good)		
	Excellent	1.09	1.00
	Very good	1.06	1.00
	Fair	1.14	1.00
	Poor	1.21	1.00
Immigration status	(Ref: Born in this country)		
	Born in another country	1.31	1.00
Age 12 religious service attendance	(Ref: Never)		
	At least 1/week	1.23	1.05
	1-3/month	1.12	1.00
	< 1/month	1.21	1.00
Year of birth	(Ref: 1998-2005; age 18-24)		
	1988-1998; age 25-34	1.49	1.40
	1978-1988; age 35-44	1.60	1.52
	1968-1978; age 45-54	1.69	1.60
	1958-1968; age 55-64	1.69	1.59
	1948-1957; age 65-74	1.39	1.26
	1938-1948; age 75-84	1.25	1.00
	1938 or earlier; 85 or older	1.17	1.00
Gender	(Ref: Male)		
	Female	2.07	2.02
	Other	1.70	1.00

139. Table S139. Nationally representative descriptive statistics for Argentina conditional on smokers: Childhood predictors

Characteristic	N = 2,293[1]
**Relationship with mother**	
Very good	1,508 (66%)
Somewhat good	469 (20%)
Somewhat bad	108 (4.7%)
Very bad	86 (3.8%)
Does not apply	107 (4.7%)
(Missing)	15 (0.7%)
**Relationship with father**	
Very good	1,150 (50%)
Somewhat good	538 (23%)
Somewhat bad	151 (6.6%)
Very bad	167 (7.3%)
Does not apply	270 (12%)
(Missing)	18 (0.8%)
**Parent marital status**	
Parents married	1,291 (56%)
Divorced	261 (11%)
Parents were never married	504 (22%)
One or both parents had died	67 (2.9%)
(Missing)	170 (7.4%)
**Subjective financial status of family growing up**	
Lived comfortably	664 (29%)
Got by	746 (33%)
Found it difficult	654 (29%)
Found it very difficult	221 (9.7%)
(Missing)	8 (0.3%)
**Abuse**	
Yes	489 (21%)
No	1,734 (76%)
(Missing)	71 (3.1%)
**Outsider growing up**	
Yes	468 (20%)
No	1,778 (78%)
(Missing)	47 (2.1%)
**Self-rated health growing up**	
Excellent	902 (39%)
Very good	571 (25%)
Good	596 (26%)
Fair	161 (7.0%)
Poor	56 (2.4%)
(Missing)	6 (0.3%)
**Immigration status**	
Born in this country	2,205 (96%)
Born in another country	86 (3.7%)
(Missing)	3 (0.1%)
**Age 12 religious service attendance**	
At least 1/week	843 (37%)
1-3/month	419 (18%)
<1/month	362 (16%)
Never	652 (28%)
(Missing)	17 (0.7%)

140. Table S140. Nationally representative descriptive statistics for Argentina conditional on smokers: Demographic variables

Characteristic	N = 2,293[1]
**Year of birth**	
1998-2005; age 18-24	343 (15%)
1988-1998; age 25-34	603 (26%)
1978-1988; age 35-44	521 (23%)
1968-1978; age 45-54	356 (16%)
1958-1968; age 55-64	267 (12%)
1948-1958; age 65-74	172 (7.5%)
1938-1948; age 75-84	28 (1.2%)
1938 or earlier; age 85+	3 (0.1%)
(Missing)	0 (0%)
**Gender**	
Male	1,142 (50%)
Female	1,134 (49%)
Other	5 (0.2%)
(Missing)	13 (0.6%)
**Religious affiliation**	
Christianity	1,943 (85%)
Islam	0 (0%)
Hinduism	2 (<0.1%)
Buddhism	1 (<0.1%)
Judaism	25 (1.1%)
Sikhism	5 (0.2%)
Baha'i	0 (0%)
Jainism	0 (0%)
Shinto	0 (0%)
Taoism	1 (<0.1%)
Confucianism	0 (0%)
Primal, Animist, or Folk religion	4 (0.2%)
Spiritism	0 (0%)
Umbanda, Candomble, and other African-derived religions	0 (0%)
Chinese folk/traditional religion	0 (0%)
Some other religion	4 (0.2%)
No religion/Atheist/Agnostic	269 (12%)
(Missing)	39 (1.7%)
**Race/Ethnicity**	
Asian	17 (0.7%)
Black	40 (1.7%)
Indigenous	58 (2.5%)
Mestizo(a)	610 (27%)
Mullato(a)	18 (0.8%)
Other	42 (1.8%)
White	1,088 (47%)
(Missing)	420 (18%)

[1]n (%)

141. Table S141. Childhood predictors regression for Argentina conditional on smokers

Variable	Category	Est	95% CI	SE	Global p-value
Relationship with mother	(Ref: Very bad/somewhat bad)				0.503
	Very good/somewhat good	0.68	(-1.52, 2.88)	1.12	
Relationship with father	(Ref: Very bad/somewhat bad)				0.635
	Very good/somewhat good	-0.41	(-2.16, 1.33)	0.89	
Parent marital status	(Ref: Parents married)				0.568
	Divorced	0.37	(-1.72, 2.47)	1.07	
	Parents were never married	-0.65	(-2.29, 0.99)	0.83	
	One or both parents had died	0.51	(-2.57, 3.58)	1.55	
Subjective financial status of family growing up	(Ref: Got by)				0.177
	Lived comfortably	-0.72	(-2.18, 0.73)	0.74	
	Found it difficult	-1.02	(-2.60, 0.57)	0.81	
	Found it very difficult	1.63	(-1.11, 4.36)	1.40	
Abuse	(Ref: No)				0.059
	Yes	1.59	(-0.10, 3.28)	0.86	
Outsider growing up	(Ref: No)				0.310
	Yes	0.86	(-0.88, 2.61)	0.89	
Self-rated health growing up	(Ref: Good)				0.163
	Excellent	0.19	(-1.34, 1.72)	0.78	
	Very good	-1.02	(-2.49, 0.46)	0.75	
	Fair	1.13	(-2.46, 4.72)	1.83	
	Poor	4.38	(-2.75, 11.52)	3.64	
Immigration status	(Ref: Born in this country)				0.156
	Born in another country	-1.70	(-4.05, 0.66)	1.20	
Age 12 religious service attendance	(Ref: Never)				0.072
	At least 1/week	0.32	(-1.45, 2.09)	0.90	
	1-3/month	-1.55	(-3.19, 0.09)	0.84	
	< 1/month	-0.82	(-2.47, 0.83)	0.84	
Year of birth	(Ref: 1998-2005; current age: 18-24)				4.48e-04
	1988-1998; age 25-34	2.33	(0.15, 4.52)	1.12	
	1978-1988; age 35-44	3.70	(1.65, 5.75)	1.05	
	1968-1978; age 45-54	3.90	(1.77, 6.02)	1.08	
	1958-1968; age 55-64	5.12	(2.74, 7.51)	1.22	
	1948-1957; age 65-74	5.10	(2.43, 7.76)	1.36	
	1938-1948; age 75-84	2.87	(-0.77, 6.52)	1.86	
	1938 or earlier; 85 or older	3.61	(-0.11, 7.32)	1.90	
Gender	(Ref: Male)				1.57e-05
	Female	-2.95	(-4.19, -1.71)	0.63	
	Other	-0.91	(-9.67, 7.84)	4.47	
Religious affiliation	(Ref: No religion/Atheist/Agnostic)				3.98e-05
	Christianity	-0.82	(-2.77, 1.13)	0.99	
	Collapsed affiliations with prevalence<3%	-6.83	(-9.84, -3.83)	1.53	
Race/ethnicity	(Ref: Plurality group)				0.104
	Non-plurality groups	-0.92	(-2.67, 0.82)	0.84	

142. Table S142. Sensitivity to unmeasured confounding of childhood predictors in Argentina conditional on smokers

Variable	Category	E-value for Estimate	E-value for 95% CI
Relationship with mother	(Ref: Very bad/somewhat bad)		
	Very good/somewhat good	1.32	1.00
Relationship with father	(Ref: Very bad/somewhat bad)		
	Very good/somewhat good	1.24	1.00
Parent marital status	(Ref: Parents married)		
	Divorced	1.22	1.00
	Parents were never married	1.31	1.00
	One or both parents had died	1.27	1.00
Subjective financial status of family growing up	(Ref: Got by)		
	Lived comfortably	1.34	1.00
	Found it difficult	1.42	1.00
	Found it very difficult	1.59	1.00
Abuse	(Ref: No)		
	Yes	1.58	1.00
Outsider growing up	(Ref: No)		
	Yes	1.38	1.00
Self-rated health growing up	(Ref: Good)		
	Excellent	1.15	1.00
	Very good	1.42	1.00
	Fair	1.45	1.00
	Poor	2.34	1.00
Immigration status	(Ref: Born in this country)		
	Born in another country	1.61	1.00
Age 12 religious service attendance	(Ref: Never)		
	At least 1/week	1.20	1.00
	1-3/month	1.57	1.00
	< 1/month	1.37	1.00
Year of birth	(Ref: 1998-2005; current age: 18-24)		
	1988-1998; age 25-34	1.77	1.13
	1978-1988; age 35-44	2.14	1.59
	1968-1978; age 45-54	2.20	1.63
	1958-1968; age 55-64	2.56	1.88
	1948-1957; age 65-74	2.55	1.80
	1938-1948; age 75-84	1.92	1.00
	1938 or earlier; 85 or older	2.12	1.00
Gender	(Ref: Male)		
	Female	1.94	1.61
	Other	1.39	1.00
Religious affiliation	(Ref: No religion/Atheist/Agnostic)		
	Christianity	1.36	1.00
	Collapsed affiliations with prevalence<3%	3.11	2.18
Race/ethnicity	(Ref: Plurality group)		
	Non-plurality groups	1.39	1.00

143. **Table S143. Nationally representative descriptive statistics for Australia conditional on smokers: Childhood predictors**

Characteristic	N = 483[1]
**Relationship with mother**	
Very good	291 (60%)
Somewhat good	113 (24%)
Somewhat bad	40 (8.3%)
Very bad	33 (6.8%)
Does not apply	4 (0.8%)
(Missing)	2 (0.4%)
**Relationship with father**	
Very good	209 (43%)
Somewhat good	146 (30%)
Somewhat bad	50 (10%)
Very bad	49 (10%)
Does not apply	26 (5.4%)
(Missing)	2 (0.4%)
**Parent marital status**	
Parents married	321 (67%)
Divorced	102 (21%)
Parents were never married	36 (7.5%)
One or both parents had died	14 (3.0%)
(Missing)	9 (1.8%)
**Subjective financial status of family growing up**	
Lived comfortably	180 (37%)
Got by	210 (44%)
Found it difficult	64 (13%)
Found it very difficult	28 (5.8%)
(Missing)	1 (0.1%)
**Abuse**	
Yes	162 (34%)
No	310 (64%)
(Missing)	10 (2.1%)
**Outsider growing up**	
Yes	158 (33%)
No	325 (67%)
(Missing)	0 (0%)
**Self-rated health growing up**	
Excellent	212 (44%)
Very good	138 (29%)
Good	63 (13%)
Fair	47 (9.8%)
Poor	23 (4.7%)
(Missing)	0 (0%)
**Immigration status**	
Born in this country	395 (82%)
Born in another country	88 (18%)
(Missing)	0 (0%)
**Age 12 religious service attendance**	
At least 1/week	136 (28%)
1-3/month	55 (11%)
<1/month	71 (15%)
Never	212 (44%)
(Missing)	10 (2.0%)

144. Table S144. Nationally representative descriptive statistics for Australia conditional on smokers: Demographic variables

Characteristic	N = 483[1]
**Year of birth**	
1998-2005; age 18-24	20 (4.2%)
1988-1998; age 25-34	77 (16%)
1978-1988; age 35-44	106 (22%)
1968-1978; age 45-54	108 (22%)
1958-1968; age 55-64	103 (21%)
1948-1958; age 65-74	39 (8.1%)
1938-1948; age 75-84	23 (4.8%)
1938 or earlier; age 85+	7 (1.4%)
(Missing)	0 (0%)
**Gender**	
Male	266 (55%)
Female	209 (43%)
Other	6 (1.2%)
(Missing)	2 (0.4%)
**Religious affiliation**	
Christianity	313 (65%)
Islam	8 (1.7%)
Hinduism	0 (<0.1%)
Buddhism	0 (0%)
Judaism	1 (0.2%)
Sikhism	0 (0%)
Baha'i	0 (0%)
Jainism	0 (0%)
Shinto	0 (0%)
Taoism	0 (0%)
Confucianism	0 (0%)
Primal, Animist, or Folk religion	0 (0%)
Spiritism	0 (0%)
Umbanda, Candomble, and other African-derived religions	0 (0%)
Chinese folk/traditional religion	0 (0%)
Some other religion	0 (0%)
No religion/Atheist/Agnostic	158 (33%)
(Missing)	2 (0.5%)
**Race/Ethnicity**	
Aboriginal	11 (2.3%)
Australian	248 (51%)
Australian British/European	132 (27%)
Chinese	4 (0.9%)
Indian	0 (<0.1%)
Japanese	0 (0%)
Malay	0 (0%)
New Zealander	18 (3.7%)
Other	25 (5.3%)
Other European	32 (6.6%)
Russian	3 (0.6%)
Samoan	4 (0.9%)
Sinhalese	0 (0%)
Spanish	0 (<0.1%)
Sri Lankan Moor	0 (0%)
Sri Lankan Tamil	0 (0%)
Vietnamese	1 (0.2%)
(Missing)	3 (0.7%)

145. Table S145. Childhood predictors regression for Australia conditional on smokers

Variable	Category	Est	95% CI	SE	Global p-value
Relationship with mother	(Ref: Very bad/somewhat bad)				0.064
	Very good/somewhat good	-2.75	(-5.65, 0.15)	1.48	
Relationship with father	(Ref: Very bad/somewhat bad)				0.935
	Very good/somewhat good	0.04	(-2.71, 2.78)	1.40	
Parent marital status	(Ref: Parents married)				0.176
	Divorced	2.91	(0.30, 5.51)	1.33	
	Parents were never married	0.95	(-2.78, 4.69)	1.90	
	One or both parents had died	0.07	(-4.97, 5.12)	2.57	
Subjective financial status of family growing up	(Ref: Got by)				0.509
	Lived comfortably	0.26	(-2.11, 2.63)	1.21	
	Found it difficult	-0.70	(-3.38, 1.97)	1.36	
	Found it very difficult	2.45	(-1.59, 6.48)	2.06	
Abuse	(Ref: No)				0.854
	Yes	0.13	(-2.22, 2.49)	1.20	
Outsider growing up	(Ref: No)				0.015
	Yes	-2.90	(-5.23, -0.56)	1.19	
Self-rated health growing up	(Ref: Good)				0.017
	Excellent	-4.95	(-7.98, -1.93)	1.54	
	Very good	-3.64	(-6.76, -0.52)	1.59	
	Fair	-2.79	(-7.20, 1.63)	2.25	
	Poor	1.11	(-6.19, 8.41)	3.72	
Immigration status	(Ref: Born in this country)				0.824
	Born in another country	-0.33	(-3.62, 2.96)	1.68	
Age 12 religious service attendance	(Ref: Never)				0.124
	At least 1/week	0.81	(-1.81, 3.44)	1.34	
	1-3/month	-2.57	(-6.31, 1.17)	1.91	
	< 1/month	1.83	(-1.40, 5.06)	1.65	
Year of birth	(Ref: 1998-2005; current age: 18-24)				2.25e-06
	1988-1998; age 25-34	5.11	(1.89, 8.34)	1.65	
	1978-1988; age 35-44	9.49	(5.98, 13.00)	1.79	
	1968-1978; age 45-54	9.78	(6.24, 13.32)	1.81	
	1958-1968; age 55-64	8.68	(5.49, 11.86)	1.62	
	1948-1957; age 65-74	8.93	(4.19, 13.68)	2.42	
	1938-1948; age 75-84	9.41	(3.26, 15.56)	3.14	
	1938 or earlier; 85 or older	10.60	(4.70, 16.49)	3.01	
Gender	(Ref: Male)				0.135
	Female	-1.39	(-3.44, 0.67)	1.05	
	Other	-5.27	(-11.35, 0.82)	3.10	
Religious affiliation	(Ref: No religion/Atheist/Agnostic)				0.491
	Christianity	1.24	(-1.76, 4.24)	1.53	
	Collapsed affiliations with prevalence<3%	3.39	(-2.65, 9.42)	3.08	
Race/ethnicity	(Ref: Plurality group)				0.697
	Non-plurality groups	-0.32	(-2.45, 1.81)	1.08	

146. Table S146. Sensitivity to unmeasured confounding of childhood predictors in Australia conditional on smokers

Variable	Category	E-value for Estimate	E-value for 95% CI
Relationship with mother	(Ref: Very bad/somewhat bad)		
	Very good/somewhat good	2.05	1.00
Relationship with father	(Ref: Very bad/somewhat bad)		
	Very good/somewhat good	1.07	1.00
Parent marital status	(Ref: Parents married)		
	Divorced	2.10	1.22
	Parents were never married	1.46	1.00
	One or both parents had died	1.10	1.00
Subjective financial status of family growing up	(Ref: Got by)		
	Lived comfortably	1.20	1.00
	Found it difficult	1.38	1.00
	Found it very difficult	1.95	1.00
Abuse	(Ref: No)		
	Yes	1.14	1.00
Outsider growing up	(Ref: No)		
	Yes	2.10	1.33
Self-rated health growing up	(Ref: Good)		
	Excellent	2.85	1.78
	Very good	2.35	1.31
	Fair	2.06	1.00
	Poor	1.51	1.00
Immigration status	(Ref: Born in this country)		
	Born in another country	1.23	1.00
Age 12 religious service attendance	(Ref: Never)		
	At least 1/week	1.41	1.00
	1-3/month	1.99	1.00
	< 1/month	1.75	1.00
Year of birth	(Ref: 1998-2005; current age: 18-24)		
	1988-1998; age 25-34	2.91	1.77
	1978-1988; age 35-44	5.14	3.28
	1968-1978; age 45-54	5.33	3.40
	1958-1968; age 55-64	4.65	3.07
	1948-1957; age 65-74	4.80	2.56
	1938-1948; age 75-84	5.10	2.23
	1938 or earlier; 85 or older	5.89	2.75
Gender	(Ref: Male)		
	Female	1.60	1.00
	Other	2.97	1.00
Religious affiliation	(Ref: No religion/Atheist/Agnostic)		
	Christianity	1.56	1.00
	Collapsed affiliations with prevalence<3%	2.26	1.00
Race/ethnicity	(Ref: Plurality group)		
	Non-plurality groups	1.23	1.00

147. Table S147. Nationally representative descriptive statistics for Brazil conditional on smokers: Childhood predictors

Characteristic	N = 2,785[1]
**Relationship with mother**	
Very good	1,718 (62%)
Somewhat good	725 (26%)
Somewhat bad	146 (5.2%)
Very bad	62 (2.2%)
Does not apply	121 (4.3%)
(Missing)	13 (0.5%)
**Relationship with father**	
Very good	1,282 (46%)
Somewhat good	739 (27%)
Somewhat bad	213 (7.7%)
Very bad	201 (7.2%)
Does not apply	328 (12%)
(Missing)	21 (0.8%)
**Parent marital status**	
Parents married	1,669 (60%)
Divorced	394 (14%)
Parents were never married	430 (15%)
One or both parents had died	105 (3.8%)
(Missing)	187 (6.7%)
**Subjective financial status of family growing up**	
Lived comfortably	1,039 (37%)
Got by	992 (36%)
Found it difficult	511 (18%)
Found it very difficult	228 (8.2%)
(Missing)	15 (0.5%)
**Abuse**	
Yes	603 (22%)
No	2,073 (74%)
(Missing)	110 (3.9%)
**Outsider growing up**	
Yes	433 (16%)
No	2,272 (82%)
(Missing)	80 (2.9%)
**Self-rated health growing up**	
Excellent	1,254 (45%)
Very good	675 (24%)
Good	540 (19%)
Fair	268 (9.6%)
Poor	46 (1.7%)
(Missing)	2 (<0.1%)
**Immigration status**	
Born in this country	2,669 (96%)
Born in another country	35 (1.3%)
(Missing)	82 (2.9%)
**Age 12 religious service attendance**	
At least 1/week	1,258 (45%)
1-3/month	569 (20%)
<1/month	567 (20%)
Never	378 (14%)
(Missing)	12 (0.4%)

148. Table S148. Nationally representative descriptive statistics for Brazil conditional on smokers: Demographic variables

Characteristic	N = 2,785[1]
**Year of birth**	
1998-2005; age 18-24	258 (9.3%)
1988-1998; age 25-34	619 (22%)
1978-1988; age 35-44	684 (25%)
1968-1978; age 45-54	525 (19%)
1958-1968; age 55-64	462 (17%)
1948-1958; age 65-74	218 (7.8%)
1938-1948; age 75-84	12 (0.4%)
1938 or earlier; age 85+	6 (0.2%)
(Missing)	0 (0%)
**Gender**	
Male	1,549 (56%)
Female	1,216 (44%)
Other	10 (0.4%)
(Missing)	10 (0.4%)
**Religious affiliation**	
Christianity	2,252 (81%)
Islam	1 (<0.1%)
Hinduism	0 (<0.1%)
Buddhism	10 (0.3%)
Judaism	14 (0.5%)
Sikhism	0 (0%)
Baha'i	0 (0%)
Jainism	3 (0.1%)
Shinto	1 (<0.1%)
Taoism	1 (<0.1%)
Confucianism	1 (<0.1%)
Primal, Animist, or Folk religion	2 (<0.1%)
Spiritism	130 (4.7%)
Umbanda, Candomble, and other African-derived religions	114 (4.1%)
Chinese folk/traditional religion	0 (0%)
Some other religion	21 (0.7%)
No religion/Atheist/Agnostic	215 (7.7%)
(Missing)	21 (0.8%)
**Race/Ethnicity**	
Amarela	58 (2.1%)
Branca	1,105 (40%)
Indígena	36 (1.3%)
Other	17 (0.6%)
Parda	1,018 (37%)
Preta	339 (12%)
(Missing)	213 (7.6%)

[1]n (%)

149. Table S149. Childhood predictors regression for Brazil conditional on smokers

Variable	Category	Est	95% CI	SE	Global p-value
Relationship with mother	(Ref: Very bad/somewhat bad)				0.605
	Very good/somewhat good	-0.45	(-2.31, 1.41)	0.95	
Relationship with father	(Ref: Very bad/somewhat bad)				0.152
	Very good/somewhat good	-0.85	(-2.06, 0.37)	0.62	
Parent marital status	(Ref: Parents married)				0.851
	Divorced	0.02	(-1.25, 1.29)	0.65	
	Parents were never married	-0.03	(-1.36, 1.30)	0.68	
	One or both parents had died	0.93	(-1.65, 3.51)	1.31	
Subjective financial status of family growing up	(Ref: Got by)				0.262
	Lived comfortably	0.01	(-1.04, 1.06)	0.54	
	Found it difficult	-0.04	(-1.31, 1.23)	0.65	
	Found it very difficult	1.87	(-0.12, 3.85)	1.01	
Abuse	(Ref: No)				0.719
	Yes	0.19	(-1.13, 1.51)	0.67	
Outsider growing up	(Ref: No)				0.350
	Yes	0.78	(-0.89, 2.44)	0.85	
Self-rated health growing up	(Ref: Good)				0.333
	Excellent	0.41	(-0.85, 1.67)	0.64	
	Very good	-0.01	(-1.41, 1.40)	0.72	
	Fair	1.70	(-0.18, 3.58)	0.96	
	Poor	2.89	(-4.10, 9.88)	3.57	
Immigration status	(Ref: Born in this country)				1.96e-04
	Born in another country	-4.15	(-6.35, -1.94)	1.12	
Age 12 religious service attendance	(Ref: Never)				0.675
	At least 1/week	-0.72	(-2.24, 0.81)	0.78	
	1-3/month	-0.77	(-2.53, 0.99)	0.90	
	< 1/month	-0.26	(-1.85, 1.33)	0.81	
Year of birth	(Ref: 1998-2005; current age: 18-24)				0.002
	1988-1998; age 25-34	1.88	(0.27, 3.50)	0.83	
	1978-1988; age 35-44	1.99	(0.42, 3.55)	0.80	
	1968-1978; age 45-54	3.08	(1.36, 4.79)	0.88	
	1958-1968; age 55-64	2.86	(0.93, 4.78)	0.98	
	1948-1957; age 65-74	4.35	(1.43, 7.27)	1.49	
	1938-1948; age 75-84	-2.97	(-7.69, 1.75)	2.41	
	1938 or earlier; 85 or older	-0.34	(-4.58, 3.91)	2.17	
Gender	(Ref: Male)				7.64e-04
	Female	-1.66	(-2.64, -0.68)	0.50	
	Other	-4.60	(-8.31, -0.89)	1.89	
Religious affiliation	(Ref: No religion/Atheist/Agnostic)				0.207
	Spiritism	3.20	(0.13, 6.27)	1.57	
	Umbanda, Candomble, and other African-derived religions	2.20	(-0.34, 4.73)	1.29	
	Christianity	0.97	(-0.54, 2.49)	0.77	
	Collapsed affiliations with prevalence<3%	1.29	(-2.31, 4.89)	1.84	
Race/ethnicity	(Ref: Plurality group)				7.06e-04
	Non-plurality groups	-1.50	(-2.45, -0.55)	0.48	

150. Table S150. Sensitivity to unmeasured confounding of childhood predictors in Brazil conditional on smokers

Variable	Category	E-value for Estimate	E-value for 95% CI
Relationship with mother	(Ref: Very bad/somewhat bad)		
	Very good/somewhat good	1.26	1.00
Relationship with father	(Ref: Very bad/somewhat bad)		
	Very good/somewhat good	1.39	1.00
Parent marital status	(Ref: Parents married)		
	Divorced	1.05	1.00
	Parents were never married	1.05	1.00
	One or both parents had died	1.41	1.00
Subjective financial status of family growing up	(Ref: Got by)		
	Lived comfortably	1.04	1.00
	Found it difficult	1.07	1.00
	Found it very difficult	1.68	1.00
Abuse	(Ref: No)		
	Yes	1.16	1.00
Outsider growing up	(Ref: No)		
	Yes	1.37	1.00
Self-rated health growing up	(Ref: Good)		
	Excellent	1.25	1.00
	Very good	1.02	1.00
	Fair	1.64	1.00
	Poor	1.97	1.00
Immigration status	(Ref: Born in this country)		
	Born in another country	2.35	1.71
Age 12 religious service attendance	(Ref: Never)		
	At least 1/week	1.35	1.00
	1-3/month	1.37	1.00
	< 1/month	1.19	1.00
Year of birth	(Ref: 1998-2005; current age: 18-24)		
	1988-1998; age 25-34	1.69	1.19
	1978-1988; age 35-44	1.72	1.25
	1968-1978; age 45-54	2.03	1.54
	1958-1968; age 55-64	1.96	1.41
	1948-1957; age 65-74	2.41	1.56
	1938-1948; age 75-84	2.00	1.00
	1938 or earlier; 85 or older	1.22	1.00
Gender	(Ref: Male)		
	Female	1.62	1.34
	Other	2.49	1.41
Religious affiliation	(Ref: No religion/Atheist/Agnostic)		
	Spiritism	2.06	1.13
	Umbanda, Candomble, and other African-derived religions	1.78	1.00
	Christianity	1.43	1.00
	Collapsed affiliations with prevalence<3%	1.52	1.00
Race/ethnicity	(Ref: Plurality group)		
	Non-plurality groups	1.58	1.30

151. Table S151. Nationally representative descriptive statistics for Egypt conditional on smokers: Childhood predictors

Characteristic	N = 1,085[1]
**Relationship with mother**	
Very good	973 (90%)
Somewhat good	90 (8.3%)
Somewhat bad	2 (0.2%)
Very bad	2 (0.2%)
Does not apply	18 (1.7%)
(Missing)	0 (0%)
**Relationship with father**	
Very good	844 (78%)
Somewhat good	151 (14%)
Somewhat bad	15 (1.4%)
Very bad	12 (1.1%)
Does not apply	57 (5.3%)
(Missing)	6 (0.5%)
**Parent marital status**	
Parents married	891 (82%)
Divorced	40 (3.6%)
Parents were never married	1 (<0.1%)
One or both parents had died	130 (12%)
(Missing)	24 (2.2%)
**Subjective financial status of family growing up**	
Lived comfortably	283 (26%)
Got by	523 (48%)
Found it difficult	218 (20%)
Found it very difficult	60 (5.6%)
(Missing)	0 (0%)
**Abuse**	
Yes	135 (12%)
No	942 (87%)
(Missing)	8 (0.7%)
**Outsider growing up**	
Yes	59 (5.4%)
No	1,018 (94%)
(Missing)	8 (0.8%)
**Self-rated health growing up**	
Excellent	594 (55%)
Very good	306 (28%)
Good	113 (10%)
Fair	47 (4.3%)
Poor	25 (2.3%)
(Missing)	0 (0%)
**Immigration status**	
Born in this country	1,081 (100%)
Born in another country	4 (0.3%)
(Missing)	0 (0%)
**Age 12 religious service attendance**	
At least 1/week	711 (66%)
1-3/month	150 (14%)
<1/month	101 (9.3%)
Never	116 (11%)
(Missing)	7 (0.6%)

152. Table S152. Nationally representative descriptive statistics for Egypt conditional on smokers: Demographic variables

Characteristic	N = 1,085[1]
**Year of birth**	
1998-2005; age 18-24	193 (18%)
1988-1998; age 25-34	329 (30%)
1978-1988; age 35-44	226 (21%)
1968-1978; age 45-54	155 (14%)
1958-1968; age 55-64	152 (14%)
1948-1958; age 65-74	30 (2.7%)
1938-1948; age 75-84	1 (<0.1%)
1938 or earlier; age 85+	0 (0%)
(Missing)	0 (0%)
**Gender**	
Male	1,055 (97%)
Female	30 (2.8%)
Other	0 (0%)
(Missing)	0 (0%)
**Religious affiliation**	
Christianity	17 (1.6%)
Islam	1,065 (98%)
Hinduism	0 (0%)
Buddhism	0 (0%)
Judaism	0 (0%)
Sikhism	0 (0%)
Baha'i	0 (0%)
Jainism	0 (0%)
Shinto	0 (0%)
Taoism	0 (<0.1%)
Confucianism	0 (0%)
Primal, Animist, or Folk religion	0 (0%)
Spiritism	0 (0%)
Umbanda, Candomble, and other African-derived religions	0 (0%)
Chinese folk/traditional religion	0 (0%)
Some other religion	0 (0%)
No religion/Atheist/Agnostic	0 (0%)
(Missing)	3 (0.2%)
**Race/Ethnicity**	
Arab	1,063 (98%)
Bedouin Arab	1 (<0.1%)
Greek	0 (0%)
Nubian	9 (0.8%)
Turkish	2 (0.2%)
(Missing)	10 (0.9%)

[1]n (%)

153. Table S153. Childhood predictors regression for Egypt conditional on smokers

Variable	Category	Est	95% CI	SE	Global p-value
Relationship with mother	(Ref: Very bad/somewhat bad)				0.036
	Very good/somewhat good	5.36	(0.33, 10.40)	2.57	
Relationship with father	(Ref: Very bad/somewhat bad)				0.125
	Very good/somewhat good	-5.43	(-12.32, 1.46)	3.51	
Parent marital status	(Ref: Parents married)				0.002
	Divorced	2.42	(-2.26, 7.11)	2.39	
	Parents were never married	4.32	(2.23, 6.41)	1.07	
	One or both parents had died	1.48	(-0.76, 3.73)	1.14	
Subjective financial status of family growing up	(Ref: Got by)				0.949
	Lived comfortably	0.20	(-1.39, 1.79)	0.81	
	Found it difficult	-0.30	(-2.23, 1.64)	0.99	
	Found it very difficult	0.56	(-2.49, 3.60)	1.55	
Abuse	(Ref: No)				0.051
	Yes	2.46	(0.02, 4.89)	1.24	
Outsider growing up	(Ref: No)				0.664
	Yes	-0.73	(-4.03, 2.57)	1.68	
Self-rated health growing up	(Ref: Good)				0.016
	Excellent	1.63	(-0.50, 3.77)	1.09	
	Very good	1.39	(-1.21, 3.99)	1.33	
	Fair	-1.19	(-4.18, 1.80)	1.53	
	Poor	-3.68	(-7.78, 0.42)	2.09	
Immigration status	(Ref: Born in this country)				0.246
	Born in another country	-2.99	(-12.76, 6.77)	4.81	
Age 12 religious service attendance	(Ref: Never)				0.087
	At least 1/week	0.57	(-1.95, 3.08)	1.28	
	1-3/month	-1.82	(-4.52, 0.87)	1.38	
	< 1/month	0.17	(-2.88, 3.22)	1.56	
Year of birth	(Ref: 1998-2005; current age: 18-24)				0.049
	1988-1998; age 25-34	0.81	(-1.62, 3.24)	1.24	
	1978-1988; age 35-44	-0.30	(-2.67, 2.07)	1.21	
	1968-1978; age 45-54	0.34	(-2.40, 3.09)	1.40	
	1958-1968; age 55-64	2.45	(-0.53, 5.43)	1.52	
	1948-1957; age 65-74	0.45	(-4.77, 5.67)	2.66	
	1938-1948; age 75-84	-2.71	(-6.00, 0.58)	1.68	
Gender	(Ref: Male)				0.034
	Female	-6.25	(-13.56, 1.06)	3.66	
Religious affiliation	(Ref: Islam)				0.001
	Collapsed affiliations with prevalence<3%	5.34	(2.18, 8.51)	1.61	
Race/ethnicity	(Ref: Plurality group)				0.292
	Non-plurality groups	-3.44	(-9.80, 2.92)	3.24	

154. Table S154. Sensitivity to unmeasured confounding of childhood predictors in Egypt conditional on smokers

Variable	Category	E-value for Estimate	E-value for 95% CI
Relationship with mother	(Ref: Very bad/somewhat bad)		
	Very good/somewhat good	2.74	1.22
Relationship with father	(Ref: Very bad/somewhat bad)		
	Very good/somewhat good	2.76	1.00
Parent marital status	(Ref: Parents married)		
	Divorced	1.84	1.00
	Parents were never married	2.40	1.79
	One or both parents had died	1.57	1.00
Subjective financial status of family growing up	(Ref: Got by)		
	Lived comfortably	1.16	1.00
	Found it difficult	1.20	1.00
	Found it very difficult	1.30	1.00
Abuse	(Ref: No)		
	Yes	1.85	1.06
Outsider growing up	(Ref: No)		
	Yes	1.35	1.00
Self-rated health growing up	(Ref: Good)		
	Excellent	1.62	1.00
	Very good	1.55	1.00
	Fair	1.49	1.00
	Poor	2.20	1.00
Immigration status	(Ref: Born in this country)		
	Born in another country	2.00	1.00
Age 12 religious service attendance	(Ref: Never)		
	At least 1/week	1.30	1.00
	1-3/month	1.67	1.00
	< 1/month	1.15	1.00
Year of birth	(Ref: 1998-2005; current age: 18-24)		
	1988-1998; age 25-34	1.38	1.00
	1978-1988; age 35-44	1.20	1.00
	1968-1978; age 45-54	1.22	1.00
	1958-1968; age 55-64	1.84	1.00
	1948-1957; age 65-74	1.26	1.00
	1938-1948; age 75-84	1.92	1.00
Gender	(Ref: Male)		
	Female	3.05	1.00
Religious affiliation	(Ref: Islam)		
	Collapsed affiliations with prevalence<3%	2.73	1.77
Race/ethnicity	(Ref: Plurality group)		
	Non-plurality groups	2.13	1.00

155. Table S155. Nationally representative descriptive statistics for Germany conditional on smokers: Childhood predictors

Characteristic	N = 2,590[1]
**Relationship with mother**	
Very good	1,458 (56%)
Somewhat good	789 (30%)
Somewhat bad	170 (6.6%)
Very bad	64 (2.5%)
Does not apply	93 (3.6%)
(Missing)	16 (0.6%)
**Relationship with father**	
Very good	1,240 (48%)
Somewhat good	777 (30%)
Somewhat bad	257 (9.9%)
Very bad	130 (5.0%)
Does not apply	170 (6.6%)
(Missing)	16 (0.6%)
**Parent marital status**	
Parents married	2,039 (79%)
Divorced	271 (10%)
Parents were never married	177 (6.8%)
One or both parents had died	60 (2.3%)
(Missing)	43 (1.7%)
**Subjective financial status of family growing up**	
Lived comfortably	876 (34%)
Got by	1,197 (46%)
Found it difficult	400 (15%)
Found it very difficult	108 (4.2%)
(Missing)	9 (0.4%)
**Abuse**	
Yes	385 (15%)
No	2,178 (84%)
(Missing)	28 (1.1%)
**Outsider growing up**	
Yes	370 (14%)
No	2,183 (84%)
(Missing)	38 (1.4%)
**Self-rated health growing up**	
Excellent	732 (28%)
Very good	925 (36%)
Good	704 (27%)
Fair	184 (7.1%)
Poor	38 (1.5%)
(Missing)	7 (0.3%)
**Immigration status**	
Born in this country	2,395 (92%)
Born in another country	183 (7.1%)
(Missing)	13 (0.5%)
**Age 12 religious service attendance**	
At least 1/week	545 (21%)
1-3/month	525 (20%)
<1/month	772 (30%)
Never	739 (29%)
(Missing)	9 (0.4%)

156. **Table S156. Nationally representative descriptive statistics for Germany conditional on smokers: Demographic variables**

**Characteristic**	**N = 2,590**[1]
**Year of birth**	
1998-2005; age 18-24	171 (6.6%)
1988-1998; age 25-34	391 (15%)
1978-1988; age 35-44	390 (15%)
1968-1978; age 45-54	532 (21%)
1958-1968; age 55-64	535 (21%)
1948-1958; age 65-74	480 (19%)
1938-1948; age 75-84	88 (3.4%)
1938 or earlier; age 85+	4 (0.2%)
(Missing)	0 (0%)
**Gender**	
Male	1,299 (50%)
Female	1,287 (50%)
Other	3 (0.1%)
(Missing)	1 (<0.1%)
**Religious affiliation**	
Christianity	1,530 (59%)
Islam	125 (4.8%)
Hinduism	5 (0.2%)
Buddhism	9 (0.4%)
Judaism	6 (0.2%)
Sikhism	1 (<0.1%)
Baha'i	2 (<0.1%)
Jainism	0 (0%)
Shinto	0 (0%)
Taoism	0 (0%)
Confucianism	0 (0%)
Primal, Animist, or Folk religion	7 (0.3%)
Spiritism	0 (0%)
Umbanda, Candomble, and other African-derived religions	0 (0%)
Chinese folk/traditional religion	0 (0%)
Some other religion	21 (0.8%)
No religion/Atheist/Agnostic	855 (33%)
(Missing)	28 (1.1%)

[1]n (%)

157. Table S157. Childhood predictors regression for Germany conditional on smokers

Variable	Category	Est	95% CI	SE	Global p-value
Relationship with mother	(Ref: Very bad/somewhat bad)				0.335
	Very good/somewhat good	-0.89	(-2.74, 0.97)	0.95	
Relationship with father	(Ref: Very bad/somewhat bad)				0.623
	Very good/somewhat good	0.19	(-1.32, 1.70)	0.76	
Parent marital status	(Ref: Parents married)				0.707
	Divorced	0.22	(-1.32, 1.76)	0.79	
	Parents were never married	0.19	(-1.45, 1.83)	0.84	
	One or both parents had died	-1.07	(-3.16, 1.02)	1.06	
Subjective financial status of family growing up	(Ref: Got by)				0.784
	Lived comfortably	0.39	(-0.77, 1.55)	0.59	
	Found it difficult	-0.23	(-1.65, 1.18)	0.72	
	Found it very difficult	0.60	(-1.61, 2.82)	1.13	
Abuse	(Ref: No)				0.559
	Yes	0.35	(-0.85, 1.54)	0.61	
Outsider growing up	(Ref: No)				0.463
	Yes	-0.58	(-2.19, 1.02)	0.82	
Self-rated health growing up	(Ref: Good)				0.281
	Excellent	1.19	(-0.21, 2.60)	0.72	
	Very good	0.52	(-0.63, 1.67)	0.59	
	Fair	1.58	(-0.15, 3.30)	0.88	
	Poor	1.39	(-2.37, 5.14)	1.92	
Immigration status	(Ref: Born in this country)				0.020
	Born in another country	-1.90	(-3.50, -0.30)	0.81	
Age 12 religious service attendance	(Ref: Never)				0.605
	At least 1/week	-0.09	(-1.61, 1.43)	0.78	
	1-3/month	-0.83	(-2.18, 0.52)	0.69	
	< 1/month	-0.31	(-1.59, 0.98)	0.65	
Year of birth	(Ref: 1998-2005; current age: 18-24)				2.30e-13
	1988-1998; age 25-34	1.25	(-0.66, 3.16)	0.97	
	1978-1988; age 35-44	3.51	(1.52, 5.49)	1.01	
	1968-1978; age 45-54	4.24	(2.21, 6.27)	1.03	
	1958-1968; age 55-64	5.78	(3.77, 7.79)	1.03	
	1948-1957; age 65-74	5.19	(3.05, 7.32)	1.09	
	1938-1948; age 75-84	0.70	(-1.71, 3.12)	1.23	
	1938 or earlier; 85 or older	-4.06	(-9.47, 1.34)	2.76	
Gender	(Ref: Male)				0.002
	Female	-1.43	(-2.36, -0.50)	0.48	
	Other	-3.99	(-7.35, -0.63)	1.71	
Religious affiliation	(Ref: No religion/Atheist/Agnostic)				0.815
	Islam	-1.32	(-4.33, 1.70)	1.54	
	Christianity	-0.08	(-1.27, 1.12)	0.61	
	Collapsed affiliations with prevalence<3%	0.44	(-3.02, 3.90)	1.77	
Race/ethnicity	(Ref: Plurality group)				

158. Table S158. Sensitivity to unmeasured confounding of childhood predictors in Germany conditional on smokers

Variable	Category	E-value for Estimate	E-value for 95% CI
Relationship with mother	(Ref: Very bad/somewhat bad)		
	Very good/somewhat good	1.40	1.00
Relationship with father	(Ref: Very bad/somewhat bad)		
	Very good/somewhat good	1.16	1.00
Parent marital status	(Ref: Parents married)		
	Divorced	1.17	1.00
	Parents were never married	1.16	1.00
	One or both parents had died	1.46	1.00
Subjective financial status of family growing up	(Ref: Got by)		
	Lived comfortably	1.24	1.00
	Found it difficult	1.18	1.00
	Found it very difficult	1.31	1.00
Abuse	(Ref: No)		
	Yes	1.22	1.00
Outsider growing up	(Ref: No)		
	Yes	1.31	1.00
Self-rated health growing up	(Ref: Good)		
	Excellent	1.49	1.00
	Very good	1.28	1.00
	Fair	1.60	1.00
	Poor	1.55	1.00
Immigration status	(Ref: Born in this country)		
	Born in another country	1.70	1.21
Age 12 religious service attendance	(Ref: Never)		
	At least 1/week	1.10	1.00
	1-3/month	1.39	1.00
	< 1/month	1.21	1.00
Year of birth	(Ref: 1998-2005; current age: 18-24)		
	1988-1998; age 25-34	1.51	1.00
	1978-1988; age 35-44	2.16	1.59
	1968-1978; age 45-54	2.39	1.79
	1958-1968; age 55-64	2.90	2.24
	1948-1957; age 65-74	2.70	2.03
	1938-1948; age 75-84	1.35	1.00
	1938 or earlier; 85 or older	2.33	1.00
Gender	(Ref: Male)		
	Female	1.56	1.28
	Other	2.31	1.32
Religious affiliation	(Ref: No religion/Atheist/Agnostic)		
	Islam	1.53	1.00
	Christianity	1.09	1.00
	Collapsed affiliations with prevalence<3%	1.26	1.00
Race/ethnicity	(Ref: Plurality group)		

159. Table S159. Nationally representative descriptive statistics for Hong Kong conditional on smokers: Childhood predictors

Characteristic	N = 956[1]
**Relationship with mother**	
Very good	322 (34%)
Somewhat good	356 (37%)
Somewhat bad	86 (9.0%)
Very bad	13 (1.4%)
Does not apply	179 (19%)
(Missing)	0 (0%)
**Relationship with father**	
Very good	290 (30%)
Somewhat good	340 (36%)
Somewhat bad	99 (10%)
Very bad	28 (3.0%)
Does not apply	197 (21%)
(Missing)	1 (0.2%)
**Parent marital status**	
Parents married	889 (93%)
Divorced	34 (3.6%)
Parents were never married	9 (1.0%)
One or both parents had died	11 (1.1%)
(Missing)	14 (1.4%)
**Subjective financial status of family growing up**	
Lived comfortably	425 (44%)
Got by	415 (43%)
Found it difficult	90 (9.4%)
Found it very difficult	13 (1.4%)
(Missing)	13 (1.3%)
**Abuse**	
Yes	167 (17%)
No	789 (83%)
(Missing)	0 (0%)
**Outsider growing up**	
Yes	348 (36%)
No	580 (61%)
(Missing)	28 (2.9%)
**Self-rated health growing up**	
Excellent	290 (30%)
Very good	371 (39%)
Good	211 (22%)
Fair	65 (6.8%)
Poor	12 (1.3%)
(Missing)	7 (0.8%)
**Immigration status**	
Born in this country	894 (94%)
Born in another country	49 (5.2%)
(Missing)	13 (1.3%)
**Age 12 religious service attendance**	
At least 1/week	179 (19%)
1-3/month	306 (32%)
<1/month	217 (23%)
Never	251 (26%)
(Missing)	2 (0.3%)

160. Table S160. Nationally representative descriptive statistics for Hong Kong conditional on smokers: Demographic variables

Characteristic	N = 956[1]
**Year of birth**	
1998-2005; age 18-24	91 (9.5%)
1988-1998; age 25-34	167 (17%)
1978-1988; age 35-44	161 (17%)
1968-1978; age 45-54	257 (27%)
1958-1968; age 55-64	237 (25%)
1948-1958; age 65-74	43 (4.5%)
1938-1948; age 75-84	0 (0%)
1938 or earlier; age 85+	0 (0%)
(Missing)	0 (0%)
**Gender**	
Male	528 (55%)
Female	428 (45%)
Other	0 (0%)
(Missing)	0 (0%)
**Religious affiliation**	
.2Christianity	279 (29%)
Islam	40 (4.2%)
Hinduism	18 (1.9%)
Buddhism	161 (17%)
Judaism	16 (1.6%)
Sikhism	4 (0.4%)
Baha'i	0 (0%)
Jainism	1 (<0.1%)
Shinto	2 (0.2%)
Taoism	26 (2.8%)
Confucianism	4 (0.4%)
Primal, Animist, or Folk religion	8 (0.8%)
Spiritism	0 (0%)
Umbanda, Candomble, and other African-derived religions	0 (0%)
Chinese folk/traditional religion	45 (4.7%)
Some other religion	0 (0%)
No religion/Atheist/Agnostic	351 (37%)
(Missing)	0 (<0.1%)
**Race/Ethnicity**	
Chinese (Cantonese)	573 (60%)
Chinese (Chaoshan)	87 (9.1%)
Chinese (Fujianese)	63 (6.6%)
Chinese (Hakka)	37 (3.9%)
Chinese (Other ethnicity)	52 (5.4%)
Chinese (Shanghainese)	30 (3.2%)
East Asian (Korean, Japanese)	8 (0.8%)
Other	0 (0%)
South Asian (Indian, Nepalese, Pakistani)	5 (0.6%)
Southeast Asian (Filipino, Indonesian, Thailand)	2 (0.2%)
Taiwanese	5 (0.5%)
White	3 (0.3%)
(Missing)	90 (9.4%)

[1]n (%)

161. **Table S161. Childhood predictors regression for Hong Kong conditional on smokers**

Variable	Category	Est	95% CI	SE	Global p-value
Relationship with mother	(Ref: Very bad/somewhat bad)				0.183
	Very good/somewhat good	-0.89	(-2.28, 0.49)	0.71	
Relationship with father	(Ref: Very bad/somewhat bad)				0.281
	Very good/somewhat good	-0.75	(-2.12, 0.62)	0.70	
Parent marital status	(Ref: Parents married)				6.42e-04
	Divorced	-3.20	(-5.03, -1.37)	0.93	
	Parents were never married	-3.43	(-6.70, -0.16)	1.67	
	One or both parents had died	-1.77	(-4.78, 1.24)	1.54	
Subjective financial status of family growing up	(Ref: Got by)				1.84e-06
	Lived comfortably	-2.16	(-3.00, -1.31)	0.43	
	Found it difficult	0.19	(-1.26, 1.64)	0.74	
	Found it very difficult	-1.80	(-4.87, 1.28)	1.57	
Abuse	(Ref: No)				0.013
	Yes	-1.31	(-2.55, -0.08)	0.62	
Outsider growing up	(Ref: No)				0.009
	Yes	1.33	(0.31, 2.35)	0.52	
Self-rated health growing up	(Ref: Good)				0.002
	Excellent	-2.20	(-3.45, -0.96)	0.63	
	Very good	-1.63	(-2.97, -0.28)	0.68	
	Fair	0.62	(-1.05, 2.28)	0.85	
	Poor	-1.29	(-4.96, 2.37)	1.87	
Immigration status	(Ref: Born in this country)				0.162
	Born in another country	1.82	(-1.68, 5.32)	1.70	
Age 12 religious service attendance	(Ref: Never)				0.172
	At least 1/week	-0.14	(-1.67, 1.39)	0.78	
	1-3/month	-0.88	(-2.17, 0.41)	0.66	
	< 1/month	-1.04	(-2.15, 0.06)	0.57	
Year of birth	(Ref: 1998-2005; current age: 18-24)				0.001
	1988-1998; age 25-34	0.97	(-0.22, 2.16)	0.61	
	1978-1988; age 35-44	1.84	(0.48, 3.20)	0.69	
	1968-1978; age 45-54	3.00	(1.59, 4.40)	0.72	
	1958-1968; age 55-64	1.50	(0.34, 2.66)	0.59	
	1948-1957; age 65-74	1.99	(-2.12, 6.11)	2.10	
Gender	(Ref: Male)				0.135
	Female	-0.61	(-1.43, 0.20)	0.42	
Religious affiliation	(Ref: No religion/Atheist/Agnostic)				0.022
	Islam	0.19	(-2.60, 2.98)	1.42	
	Buddhism	-0.68	(-1.86, 0.50)	0.60	
	Chinese folk/traditional religion	0.97	(-1.04, 2.98)	1.02	
	Christianity	0.47	(-0.78, 1.73)	0.64	
	Collapsed affiliations with prevalence<3%	-1.34	(-2.90, 0.21)	0.78	
Race/ethnicity	(Ref: Plurality group)				0.059
	Non-plurality groups	-0.71	(-1.54, 0.11)	0.42	

162. Table S162. Sensitivity to unmeasured confounding of childhood predictors in Hong Kong conditional on smokers

Variable	Category	E-value for Estimate	E-value for 95% CI
Relationship with mother	(Ref: Very bad/somewhat bad)		
	Very good/somewhat good	1.61	1.00
Relationship with father	(Ref: Very bad/somewhat bad)		
	Very good/somewhat good	1.53	1.00
Parent marital status	(Ref: Parents married)		
	Divorced	2.86	1.85
	Parents were never married	3.01	1.20
	One or both parents had died	2.05	1.00
Subjective financial status of family growing up	(Ref: Got by)		
	Lived comfortably	2.25	1.82
	Found it difficult	1.22	1.00
	Found it very difficult	2.06	1.00
Abuse	(Ref: No)		
	Yes	1.82	1.15
Outsider growing up	(Ref: No)		
	Yes	1.82	1.30
Self-rated health growing up	(Ref: Good)		
	Excellent	2.28	1.64
	Very good	1.97	1.28
	Fair	1.47	1.00
	Poor	1.81	1.00
Immigration status	(Ref: Born in this country)		
	Born in another country	2.08	1.00
Age 12 religious service attendance	(Ref: Never)		
	At least 1/week	1.18	1.00
	1-3/month	1.60	1.00
	< 1/month	1.68	1.00
Year of birth	(Ref: 1998-2005; current age: 18-24)		
	1988-1998; age 25-34	1.64	1.00
	1978-1988; age 35-44	2.09	1.39
	1968-1978; age 45-54	2.73	1.96
	1958-1968; age 55-64	1.91	1.32
	1948-1957; age 65-74	2.17	1.00
Gender	(Ref: Male)		
	Female	1.46	1.00
Religious affiliation	(Ref: No religion/Atheist/Agnostic)		
	Islam	1.22	1.00
	Buddhism	1.50	1.00
	Chinese folk/traditional religion	1.65	1.00
	Christianity	1.39	1.00
	Collapsed affiliations with prevalence<3%	1.83	1.00
Race/ethnicity	(Ref: Plurality group)		
	Non-plurality groups	1.51	1.00

163. Table S163. Nationally representative descriptive statistics for India conditional on smokers: Childhood predictors

Characteristic	N = 1,484[1]
**Relationship with mother**	
Very good	1,331 (90%)
Somewhat good	91 (6.1%)
Somewhat bad	6 (0.4%)
Very bad	9 (0.6%)
Does not apply	35 (2.4%)
(Missing)	11 (0.7%)
**Relationship with father**	
Very good	1,222 (82%)
Somewhat good	134 (9.1%)
Somewhat bad	26 (1.8%)
Very bad	10 (0.7%)
Does not apply	75 (5.1%)
(Missing)	16 (1.1%)
**Parent marital status**	
Parents married	567 (38%)
Divorced	23 (1.5%)
Parents were never married	98 (6.6%)
One or both parents had died	88 (5.9%)
(Missing)	708 (48%)
**Subjective financial status of family growing up**	
Lived comfortably	529 (36%)
Got by	344 (23%)
Found it difficult	306 (21%)
Found it very difficult	294 (20%)
(Missing)	10 (0.7%)
**Abuse**	
Yes	242 (16%)
No	1,110 (75%)
(Missing)	132 (8.9%)
**Outsider growing up**	
Yes	248 (17%)
No	1,231 (83%)
(Missing)	5 (0.3%)
**Self-rated health growing up**	
Excellent	258 (17%)
Very good	455 (31%)
Good	452 (30%)
Fair	264 (18%)
Poor	44 (3.0%)
(Missing)	9 (0.6%)
**Immigration status**	
Born in this country	1,458 (98%)
Born in another country	23 (1.6%)
(Missing)	3 (0.2%)
**Age 12 religious service attendance**	
At least 1/week	572 (39%)
1-3/month	389 (26%)
<1/month	326 (22%)
Never	156 (11%)
(Missing)	41 (2.7%)

164. Table S164. Nationally representative descriptive statistics for India conditional on smokers: Demographic variables

Characteristic	N = 1,484[1]
**Year of birth**	
1998-2005; age 18-24	231 (16%)
1988-1998; age 25-34	345 (23%)
1978-1988; age 35-44	338 (23%)
1968-1978; age 45-54	273 (18%)
1958-1968; age 55-64	200 (13%)
1948-1958; age 65-74	77 (5.2%)
1938-1948; age 75-84	16 (1.1%)
1938 or earlier; age 85+	3 (0.2%)
(Missing)	0 (0%)
**Gender**	
Male	1,126 (76%)
Female	358 (24%)
Other	0 (0%)
(Missing)	0 (0%)
**Religious affiliation**	
Christianity	24 (1.6%)
Islam	184 (12%)
Hinduism	1,242 (84%)
Buddhism	8 (0.5%)
Judaism	0 (0%)
Sikhism	8 (0.5%)
Baha'i	0 (0%)
Jainism	1 (<0.1%)
Shinto	0 (0%)
Taoism	0 (0%)
Confucianism	0 (0%)
Primal, Animist, or Folk religion	3 (0.2%)
Spiritism	0 (0%)
Umbanda, Candomble, and other African-derived religions	0 (0%)
Chinese folk/traditional religion	0 (0%)
Some other religion	0 (<0.1%)
No religion/Atheist/Agnostic	0 (0%)
(Missing)	13 (0.9%)
**Race/Ethnicity**	
General	405 (27%)
Other backward caste	478 (32%)
Schedule caste	460 (31%)
Schedule tribe	118 (8.0%)
(Missing)	23 (1.6%)

[1]n (%)

165. Table S165. Childhood predictors regression for India conditional on smokers

Variable	Category	Est	95% CI	SE	Global p-value
Relationship with mother	(Ref: Very bad/somewhat bad)				0.424
	Very good/somewhat good	4.18	(-7.04, 15.41)	5.71	
Relationship with father	(Ref: Very bad/somewhat bad)				0.047
	Very good/somewhat good	8.74	(0.12, 17.36)	4.40	
Parent marital status	(Ref: Parents married)				0.015
	Divorced	-3.25	(-10.44, 3.95)	3.37	
	Parents were never married	1.11	(-4.17, 6.39)	2.55	
	One or both parents had died	3.08	(-2.66, 8.81)	2.85	
Subjective financial status of family growing up	(Ref: Got by)				0.541
	Lived comfortably	1.20	(-1.18, 3.58)	1.21	
	Found it difficult	0.73	(-2.41, 3.88)	1.60	
	Found it very difficult	2.58	(-1.76, 6.93)	2.21	
Abuse	(Ref: No)				0.224
	Yes	1.62	(-1.30, 4.53)	1.48	
Outsider growing up	(Ref: No)				0.530
	Yes	0.79	(-2.06, 3.63)	1.44	
Self-rated health growing up	(Ref: Good)				0.252
	Excellent	-0.15	(-3.16, 2.86)	1.53	
	Very good	-0.51	(-4.17, 3.16)	1.87	
	Fair	-1.78	(-4.78, 1.23)	1.53	
	Poor	9.56	(-0.59, 19.70)	5.18	
Immigration status	(Ref: Born in this country)				0.289
	Born in another country	3.48	(-3.00, 9.96)	3.31	
Age 12 religious service attendance	(Ref: Never)				0.344
	At least 1/week	-2.08	(-6.20, 2.04)	2.10	
	1-3/month	-0.25	(-4.13, 3.62)	1.97	
	< 1/month	-2.43	(-6.19, 1.32)	1.91	
Year of birth	(Ref: 1998-2005; current age: 18-24)				0.463
	1988-1998; age 25-34	1.37	(-3.32, 6.07)	2.40	
	1978-1988; age 35-44	0.60	(-3.74, 4.95)	2.22	
	1968-1978; age 45-54	2.78	(-2.08, 7.64)	2.48	
	1958-1968; age 55-64	2.55	(-2.54, 7.65)	2.60	
	1948-1957; age 65-74	0.07	(-5.04, 5.18)	2.61	
	1938-1948; age 75-84	4.20	(-3.42, 11.81)	3.89	
Gender	(Ref: Male)				0.428
	Female	2.56	(-3.98, 9.09)	3.33	
Religious affiliation	(Ref: Hinduism)				0.357
	Islam	3.27	(-1.31, 7.85)	2.34	
	Collapsed affiliations with prevalence<3%	-1.23	(-7.16, 4.71)	3.03	
Race/ethnicity	(Ref: Plurality group)				0.087
	Non-plurality groups	1.82	(-0.36, 4.01)	1.11	

166. Table S166. Sensitivity to unmeasured confounding of childhood predictors in India conditional on smokers

Variable	Category	E-value for Estimate	E-value for 95% CI
Relationship with mother	(Ref: Very bad/somewhat bad)		
	Very good/somewhat good	1.91	1.00
Relationship with father	(Ref: Very bad/somewhat bad)		
	Very good/somewhat good	2.82	1.10
Parent marital status	(Ref: Parents married)		
	Divorced	1.74	1.00
	Parents were never married	1.35	1.00
	One or both parents had died	1.71	1.00
Subjective financial status of family growing up	(Ref: Got by)		
	Lived comfortably	1.36	1.00
	Found it difficult	1.27	1.00
	Found it very difficult	1.62	1.00
Abuse	(Ref: No)		
	Yes	1.45	1.00
Outsider growing up	(Ref: No)		
	Yes	1.28	1.00
Self-rated health growing up	(Ref: Good)		
	Excellent	1.11	1.00
	Very good	1.21	1.00
	Fair	1.48	1.00
	Poor	3.00	1.00
Immigration status	(Ref: Born in this country)		
	Born in another country	1.78	1.00
Age 12 religious service attendance	(Ref: Never)		
	At least 1/week	1.53	1.00
	1-3/month	1.14	1.00
	< 1/month	1.60	1.00
Year of birth	(Ref: 1998-2005; current age: 18-24)		
	1988-1998; age 25-34	1.40	1.00
	1978-1988; age 35-44	1.24	1.00
	1968-1978; age 45-54	1.66	1.00
	1958-1968; age 55-64	1.62	1.00
	1948-1957; age 65-74	1.07	1.00
	1938-1948; age 75-84	1.91	1.00
Gender	(Ref: Male)		
	Female	1.62	1.00
Religious affiliation	(Ref: Hinduism)		
	Islam	1.75	1.00
	Collapsed affiliations with prevalence<3%	1.37	1.00
Race/ethnicity	(Ref: Plurality group)		
	Non-plurality groups	1.48	1.00

167. Table S167. Nationally representative descriptive statistics for Indonesia conditional on smokers: Childhood predictors

Characteristic	N = 2,944[1]
**Relationship with mother**	
Very good	2,583 (88%)
Somewhat good	287 (9.8%)
Somewhat bad	29 (1.0%)
Very bad	18 (0.6%)
Does not apply	20 (0.7%)
(Missing)	6 (0.2%)
**Relationship with father**	
Very good	2,536 (86%)
Somewhat good	295 (10%)
Somewhat bad	35 (1.2%)
Very bad	25 (0.8%)
Does not apply	30 (1.0%)
(Missing)	23 (0.8%)
**Parent marital status**	
Parents married	2,278 (77%)
Divorced	222 (7.6%)
Parents were never married	23 (0.8%)
One or both parents had died	304 (10%)
(Missing)	117 (4.0%)
**Subjective financial status of family growing up**	
Lived comfortably	1,362 (46%)
Got by	1,296 (44%)
Found it difficult	196 (6.7%)
Found it very difficult	84 (2.9%)
(Missing)	6 (0.2%)
**Abuse**	
Yes	238 (8.1%)
No	2,663 (90%)
(Missing)	43 (1.5%)
**Outsider growing up**	
Yes	168 (5.7%)
No	2,773 (94%)
(Missing)	3 (<0.1%)
**Self-rated health growing up**	
Excellent	542 (18%)
Very good	824 (28%)
Good	1,033 (35%)
Fair	520 (18%)
Poor	26 (0.9%)
(Missing)	0 (0%)
**Immigration status**	
Born in this country	2,932 (100%)
Born in another country	12 (0.4%)
(Missing)	0 (0%)
**Age 12 religious service attendance**	
At least 1/week	2,331 (79%)
1-3/month	355 (12%)
<1/month	121 (4.1%)
Never	116 (3.9%)
(Missing)	22 (0.7%)

168. Table S168. Nationally representative descriptive statistics for Indonesia conditional on smokers: Demographic variables

Characteristic	N = 2,944[1]
**Year of birth**	
1998-2005; age 18-24	477 (16%)
1988-1998; age 25-34	740 (25%)
1978-1988; age 35-44	709 (24%)
1968-1978; age 45-54	564 (19%)
1958-1968; age 55-64	372 (13%)
1948-1958; age 65-74	77 (2.6%)
1938-1948; age 75-84	0 (0%)
1938 or earlier; age 85+	6 (0.2%)
(Missing)	0 (0%)
**Gender**	
Male	2,496 (85%)
Female	441 (15%)
Other	4 (0.1%)
(Missing)	3 (0.1%)
**Religious affiliation**	
Christianity	194 (6.6%)
Islam	2,727 (93%)
Hinduism	18 (0.6%)
Buddhism	0 (0%)
Judaism	0 (0%)
Sikhism	0 (0%)
Baha'i	0 (0%)
Jainism	0 (0%)
Shinto	0 (0%)
Taoism	0 (0%)
Confucianism	1 (<0.1%)
Primal, Animist, or Folk religion	0 (0%)
Spiritism	0 (0%)
Umbanda, Candomble, and other African-derived religions	0 (0%)
Chinese folk/traditional religion	0 (0%)
Some other religion	0 (0%)
No religion/Atheist/Agnostic	0 (0%)
(Missing)	5 (0.2%)
**Race/Ethnicity**	
Bali	15 (0.5%)
Banjar/Melayu Banjar	126 (4.3%)
Batak	71 (2.4%)
Betawi	110 (3.7%)
Bugis	98 (3.3%)
Jawa	1,164 (40%)
Madura	116 (3.9%)
Makasar	36 (1.2%)
Minangkabau	117 (4.0%)
Other	484 (16%)
Sunda/Parahyangan	593 (20%)
(Missing)	15 (0.5%)

[1]n (%)

169. Table S169. Childhood predictors regression for Indonesia conditional on smokers

Variable	Category	Est	95% CI	SE	Global p-value
Relationship with mother	(Ref: Very bad/somewhat bad)				0.255
	Very good/somewhat good	-1.72	(-4.80, 1.36)	1.57	
Relationship with father	(Ref: Very bad/somewhat bad)				0.770
	Very good/somewhat good	-0.16	(-2.94, 2.61)	1.41	
Parent marital status	(Ref: Parents married)				0.745
	Divorced	-0.34	(-1.60, 0.92)	0.64	
	Parents were never married	-0.83	(-3.01, 1.34)	1.11	
	One or both parents had died	0.29	(-1.39, 1.97)	0.85	
Subjective financial status of family growing up	(Ref: Got by)				0.023
	Lived comfortably	-0.43	(-1.15, 0.29)	0.37	
	Found it difficult	-0.23	(-1.81, 1.36)	0.81	
	Found it very difficult	2.80	(0.61, 4.99)	1.12	
Abuse	(Ref: No)				0.347
	Yes	0.75	(-0.83, 2.34)	0.81	
Outsider growing up	(Ref: No)				0.227
	Yes	1.11	(-0.72, 2.94)	0.93	
Self-rated health growing up	(Ref: Good)				0.543
	Excellent	0.28	(-0.78, 1.34)	0.54	
	Very good	0.43	(-0.46, 1.33)	0.46	
	Fair	0.83	(-0.20, 1.87)	0.53	
	Poor	0.15	(-3.48, 3.77)	1.85	
Immigration status	(Ref: Born in this country)				0.030
	Born in another country	-3.68	(-7.39, 0.03)	1.88	
Age 12 religious service attendance	(Ref: Never)				0.684
	At least 1/week	0.21	(-1.26, 1.67)	0.75	
	1-3/month	0.11	(-1.75, 1.97)	0.95	
	< 1/month	1.31	(-1.12, 3.74)	1.24	
Year of birth	(Ref: 1998-2005; current age: 18-24)				0.017
	1988-1998; age 25-34	1.60	(0.47, 2.73)	0.58	
	1978-1988; age 35-44	2.06	(0.81, 3.31)	0.64	
	1968-1978; age 45-54	0.76	(-0.44, 1.95)	0.61	
	1958-1968; age 55-64	1.20	(-0.44, 2.84)	0.84	
	1948-1957; age 65-74	0.53	(-1.94, 3.01)	1.26	
	1938 or earlier; 85 or older	-0.56	(-6.99, 5.87)	3.28	
Gender	(Ref: Male)				7.44e-12
	Female	-3.65	(-4.86, -2.43)	0.62	
	Other	0.03	(-17.39, 17.45)	7.64	
Religious affiliation	(Ref: Islam)				0.987
	Christianity	0.11	(-2.39, 2.62)	1.28	
	Collapsed affiliations with prevalence<3%	0.18	(-3.01, 3.38)	1.63	
Race/ethnicity	(Ref: Plurality group)				0.110
	Non-plurality groups	0.60	(-0.30, 1.50)	0.45	

170. Table S170. Sensitivity to unmeasured confounding of childhood predictors in Indonesia conditional on smokers

Variable	Category	E-value for Estimate	E-value for 95% CI
Relationship with mother	(Ref: Very bad/somewhat bad)		
	Very good/somewhat good	1.81	1.00
Relationship with father	(Ref: Very bad/somewhat bad)		
	Very good/somewhat good	1.17	1.00
Parent marital status	(Ref: Parents married)		
	Divorced	1.26	1.00
	Parents were never married	1.47	1.00
	One or both parents had died	1.24	1.00
Subjective financial status of family growing up	(Ref: Got by)		
	Lived comfortably	1.30	1.00
	Found it difficult	1.20	1.00
	Found it very difficult	2.23	1.38
Abuse	(Ref: No)		
	Yes	1.44	1.00
Outsider growing up	(Ref: No)		
	Yes	1.58	1.00
Self-rated health growing up	(Ref: Good)		
	Excellent	1.23	1.00
	Very good	1.30	1.00
	Fair	1.47	1.00
	Poor	1.16	1.00
Immigration status	(Ref: Born in this country)		
	Born in another country	2.60	1.00
Age 12 religious service attendance	(Ref: Never)		
	At least 1/week	1.19	1.00
	1-3/month	1.14	1.00
	< 1/month	1.65	1.00
Year of birth	(Ref: 1998-2005; current age: 18-24)		
	1988-1998; age 25-34	1.76	1.32
	1978-1988; age 35-44	1.94	1.46
	1968-1978; age 45-54	1.44	1.00
	1958-1968; age 55-64	1.61	1.00
	1948-1957; age 65-74	1.35	1.00
	1938 or earlier; 85 or older	1.36	1.00
Gender	(Ref: Male)		
	Female	2.58	2.08
	Other	1.06	1.00
Religious affiliation	(Ref: Islam)		
	Christianity	1.14	1.00
	Collapsed affiliations with prevalence<3%	1.18	1.00
Race/ethnicity	(Ref: Plurality group)		
	Non-plurality groups	1.38	1.00

171. Table S171. Nationally representative descriptive statistics for Israel conditional on smokers: Childhood predictors

Characteristic	N = 882[1]
**Relationship with mother**	
Very good	612 (69%)
Somewhat good	218 (25%)
Somewhat bad	30 (3.4%)
Very bad	6 (0.7%)
Does not apply	7 (0.8%)
(Missing)	9 (1.0%)
**Relationship with father**	
Very good	499 (57%)
Somewhat good	230 (26%)
Somewhat bad	84 (9.5%)
Very bad	10 (1.2%)
Does not apply	50 (5.7%)
(Missing)	9 (1.1%)
**Parent marital status**	
Parents married	730 (83%)
Divorced	97 (11%)
Parents were never married	9 (1.0%)
One or both parents had died	26 (3.0%)
(Missing)	20 (2.3%)
**Subjective financial status of family growing up**	
Lived comfortably	202 (23%)
Got by	420 (48%)
Found it difficult	195 (22%)
Found it very difficult	60 (6.8%)
(Missing)	5 (0.5%)
**Abuse**	
Yes	0 (0%)
No	0 (0%)
(Missing)	882 (100%)
**Outsider growing up**	
Yes	103 (12%)
No	760 (86%)
(Missing)	19 (2.2%)
**Self-rated health growing up**	
Excellent	440 (50%)
Very good	289 (33%)
Good	123 (14%)
Fair	26 (2.9%)
Poor	0 (0%)
(Missing)	4 (0.5%)
**Immigration status**	
Born in this country	673 (76%)
Born in another country	209 (24%)
(Missing)	1 (<0.1%)
**Age 12 religious service attendance**	
At least 1/week	245 (28%)
1-3/month	75 (8.5%)
<1/month	156 (18%)
Never	405 (46%)
(Missing)	2 (0.2%)

172. Table S172. Nationally representative descriptive statistics for Israel conditional on smokers: Demographic variables

Characteristic	N = 882[1]
**Year of birth**	
1998-2005; age 18-24	99 (11%)
1988-1998; age 25-34	186 (21%)
1978-1988; age 35-44	209 (24%)
1968-1978; age 45-54	160 (18%)
1958-1968; age 55-64	134 (15%)
1948-1958; age 65-74	74 (8.3%)
1938-1948; age 75-84	18 (2.0%)
1938 or earlier; age 85+	3 (0.3%)
(Missing)	0 (0%)
**Gender**	
Male	623 (71%)
Female	255 (29%)
Other	0 (<0.1%)
(Missing)	4 (0.5%)
**Religious affiliation**	
Christianity	19 (2.1%)
Islam	172 (19%)
Hinduism	0 (0%)
Buddhism	0 (0%)
Judaism	664 (75%)
Sikhism	0 (0%)
Baha'i	1 (0.1%)
Jainism	0 (0%)
Shinto	0 (0%)
Taoism	0 (0%)
Confucianism	0 (0%)
Primal, Animist, or Folk religion	0 (0%)
Spiritism	0 (0%)
Umbanda, Candomble, and other African-derived religions	0 (0%)
Chinese folk/traditional religion	0 (0%)
Some other religion	2 (0.2%)
No religion/Atheist/Agnostic	24 (2.8%)
(Missing)	0 (0%)
**Race/Ethnicity**	
Arab	182 (21%)
Jewish	679 (77%)
Other	13 (1.5%)
(Missing)	9 (1.0%)

[1]n (%)

173. Table S173. Childhood predictors regression for Israel conditional on smokers

Variable	Category	Est	95% CI	SE	Global p-value
Relationship with mother	(Ref: Very bad/somewhat bad)				0.160
	Very good/somewhat good	1.96	(-0.79, 4.71)	1.40	
Relationship with father	(Ref: Very bad/somewhat bad)				0.852
	Very good/somewhat good	0.01	(-2.24, 2.25)	1.15	
Parent marital status	(Ref: Parents married)				0.265
	Divorced	0.45	(-4.00, 4.89)	2.08	
	Parents were never married	3.69	(-1.19, 8.57)	2.48	
	One or both parents had died	-1.85	(-4.90, 1.20)	1.54	
Subjective financial status of family growing up	(Ref: Got by)				0.018
	Lived comfortably	0.94	(-1.67, 3.56)	1.33	
	Found it difficult	-0.63	(-2.72, 1.45)	1.06	
	Found it very difficult	-3.02	(-5.26, -0.77)	1.14	
Abuse	(Ref: No)				
Outsider growing up	(Ref: No)				0.318
	Yes	1.10	(-1.15, 3.35)	1.15	
Self-rated health growing up	(Ref: Good)				0.404
	Excellent	0.00	(-2.49, 2.50)	1.26	
	Very good	1.31	(-1.08, 3.70)	1.21	
	Fair	1.31	(-3.54, 6.16)	2.47	
Immigration status	(Ref: Born in this country)				0.161
	Born in another country	-1.21	(-2.92, 0.49)	0.87	
Age 12 religious service attendance	(Ref: Never)				0.053
	At least 1/week	-2.38	(-4.77, 0.01)	1.22	
	1-3/month	-3.61	(-6.39, -0.82)	1.42	
	< 1/month	-0.66	(-2.68, 1.36)	1.03	
Year of birth	(Ref: 1998-2005; current age: 18-24)				1.89e-04
	1988-1998; age 25-34	2.93	(-0.39, 6.25)	1.69	
	1978-1988; age 35-44	3.62	(1.58, 5.65)	1.04	
	1968-1978; age 45-54	3.06	(1.06, 5.07)	1.02	
	1958-1968; age 55-64	5.25	(2.82, 7.68)	1.24	
	1948-1957; age 65-74	4.75	(1.87, 7.63)	1.47	
	1938-1948; age 75-84	3.02	(-0.23, 6.28)	1.66	
	1938 or earlier; 85 or older	-2.00	(-6.55, 2.55)	2.32	
Gender	(Ref: Male)				1.16e-08
	Female	-4.01	(-5.39, -2.63)	0.71	
	Other	-9.81	(-13.51, -6.11)	1.89	
Religious affiliation	(Ref: Judaism)				0.756
	Islam	0.44	(-7.75, 8.62)	4.15	
	Collapsed affiliations with prevalence<3%	1.09	(-2.84, 5.01)	1.98	
Race/ethnicity	(Ref: Plurality group)				0.057
	Non-plurality groups	6.96	(-0.94, 14.86)	4.01	

174. Table S174. Sensitivity to unmeasured confounding of childhood predictors in Israel conditional on smokers

Variable	Category	E-value for Estimate	E-value for 95% CI
Relationship with mother	(Ref: Very bad/somewhat bad)		
	Very good/somewhat good	1.68	1.00
Relationship with father	(Ref: Very bad/somewhat bad)		
	Very good/somewhat good	1.02	1.00
Parent marital status	(Ref: Parents married)		
	Divorced	1.25	1.00
	Parents were never married	2.16	1.00
	One or both parents had died	1.65	1.00
Subjective financial status of family growing up	(Ref: Got by)		
	Lived comfortably	1.41	1.00
	Found it difficult	1.31	1.00
	Found it very difficult	1.97	1.36
Abuse	(Ref: No)		
Outsider growing up	(Ref: No)		
	Yes	1.45	1.00
Self-rated health growing up	(Ref: Good)		
	Excellent	1.01	1.00
	Very good	1.51	1.00
	Fair	1.51	1.00
Immigration status	(Ref: Born in this country)		
	Born in another country	1.48	1.00
Age 12 religious service attendance	(Ref: Never)		
	At least 1/week	1.80	1.00
	1-3/month	2.13	1.37
	< 1/month	1.32	1.00
Year of birth	(Ref: 1998-2005; current age: 18-24)		
	1988-1998; age 25-34	1.94	1.00
	1978-1988; age 35-44	2.14	1.58
	1968-1978; age 45-54	1.98	1.44
	1958-1968; age 55-64	2.62	1.92
	1948-1957; age 65-74	2.47	1.66
	1938-1948; age 75-84	1.97	1.00
	1938 or earlier; 85 or older	1.69	1.00
Gender	(Ref: Male)		
	Female	2.25	1.86
	Other	4.36	2.91
Religious affiliation	(Ref: Judaism)		
	Islam	1.25	1.00
	Collapsed affiliations with prevalence<3%	1.45	1.00
Race/ethnicity	(Ref: Plurality group)		
	Non-plurality groups	3.20	1.00

175. Table S175. Nationally representative descriptive statistics for Japan conditional on smokers: Childhood predictors

Characteristic	N = 4,766[1]
**Relationship with mother**	
Very good	1,175 (25%)
Somewhat good	2,215 (46%)
Somewhat bad	689 (14%)
Very bad	191 (4.0%)
Does not apply	486 (10%)
(Missing)	11 (0.2%)
**Relationship with father**	
Very good	862 (18%)
Somewhat good	2,042 (43%)
Somewhat bad	862 (18%)
Very bad	290 (6.1%)
Does not apply	700 (15%)
(Missing)	10 (0.2%)
**Parent marital status**	
Parents married	4,011 (84%)
Divorced	343 (7.2%)
Parents were never married	152 (3.2%)
One or both parents had died	178 (3.7%)
(Missing)	82 (1.7%)
**Subjective financial status of family growing up**	
Lived comfortably	1,821 (38%)
Got by	2,110 (44%)
Found it difficult	579 (12%)
Found it very difficult	245 (5.1%)
(Missing)	11 (0.2%)
**Abuse**	
Yes	454 (9.5%)
No	4,293 (90%)
(Missing)	19 (0.4%)
**Outsider growing up**	
Yes	556 (12%)
No	3,826 (80%)
(Missing)	384 (8.1%)
**Self-rated health growing up**	
Excellent	617 (13%)
Very good	1,703 (36%)
Good	1,513 (32%)
Fair	711 (15%)
Poor	199 (4.2%)
(Missing)	24 (0.5%)
**Immigration status**	
Born in this country	4,505 (95%)
Born in another country	46 (1.0%)
(Missing)	215 (4.5%)
**Age 12 religious service attendance**	
At least 1/week	94 (2.0%)
1-3/month	272 (5.7%)
<1/month	1,212 (25%)
Never	3,157 (66%)
(Missing)	31 (0.7%)

176. **Table S176. Nationally representative descriptive statistics for Japan conditional on smokers: Demographic variables**

**Characteristic**	**N = 4,766**[1]
**Year of birth**	
1998-2005; age 18-24	199 (4.2%)
1988-1998; age 25-34	496 (10%)
1978-1988; age 35-44	883 (19%)
1968-1978; age 45-54	1,067 (22%)
1958-1968; age 55-64	892 (19%)
1948-1958; age 65-74	1,044 (22%)
1938-1948; age 75-84	175 (3.7%)
1938 or earlier; age 85+	10 (0.2%)
(Missing)	0 (0%)
**Gender**	
Male	3,170 (67%)
Female	1,582 (33%)
Other	3 (<0.1%)
(Missing)	11 (0.2%)
**Religious affiliation**	
Christianity	76 (1.6%)
Islam	7 (0.1%)
Hinduism	4 (<0.1%)
Buddhism	1,595 (33%)
Judaism	0 (0%)
Sikhism	0 (0%)
Baha'i	7 (0.1%)
Jainism	1 (<0.1%)
Shinto	102 (2.1%)
Taoism	3 (<0.1%)
Confucianism	7 (0.1%)
Primal, Animist, or Folk religion	2 (<0.1%)
Spiritism	0 (0%)
Umbanda, Candomble, and other African-derived religions	0 (0%)
Chinese folk/traditional religion	0 (0%)
Some other religion	11 (0.2%)
No religion/Atheist/Agnostic	2,899 (61%)
(Missing)	52 (1.1%)

[1]n (%)

177. Table S177. Childhood predictors regression for Japan conditional on smokers

Variable	Category	Est	95% CI	SE	Global p-value
Relationship with mother	(Ref: Very bad/somewhat bad)				0.187
	Very good/somewhat good	-0.51	(-1.29, 0.27)	0.40	
Relationship with father	(Ref: Very bad/somewhat bad)				0.010
	Very good/somewhat good	-0.95	(-1.68, -0.22)	0.37	
Parent marital status	(Ref: Parents married)				0.186
	Divorced	0.95	(-0.27, 2.17)	0.62	
	Parents were never married	-1.21	(-3.04, 0.62)	0.93	
	One or both parents had died	-0.12	(-2.06, 1.81)	0.99	
Subjective financial status of family growing up	(Ref: Got by)				0.859
	Lived comfortably	0.03	(-0.58, 0.65)	0.31	
	Found it difficult	0.18	(-0.71, 1.06)	0.45	
	Found it very difficult	0.61	(-0.86, 2.08)	0.75	
Abuse	(Ref: No)				0.754
	Yes	0.18	(-0.99, 1.34)	0.59	
Outsider growing up	(Ref: No)				0.066
	Yes	-0.87	(-1.84, 0.10)	0.49	
Self-rated health growing up	(Ref: Good)				0.006
	Excellent	1.32	(0.43, 2.21)	0.45	
	Very good	0.54	(-0.10, 1.18)	0.33	
	Fair	1.37	(0.44, 2.31)	0.48	
	Poor	0.06	(-1.37, 1.49)	0.73	
Immigration status	(Ref: Born in this country)				0.001
	Born in another country	-3.42	(-5.73, -1.11)	1.17	
Age 12 religious service attendance	(Ref: Never)				0.038
	At least 1/week	-0.90	(-2.68, 0.87)	0.91	
	1-3/month	-1.47	(-2.74, -0.19)	0.65	
	< 1/month	-0.73	(-1.38, -0.09)	0.33	
Year of birth	(Ref: 1998-2005; current age: 18-24)				0.000
	1988-1998; age 25-34	3.36	(1.45, 5.26)	0.97	
	1978-1988; age 35-44	5.31	(3.48, 7.13)	0.93	
	1968-1978; age 45-54	6.26	(4.44, 8.08)	0.93	
	1958-1968; age 55-64	7.08	(5.24, 8.92)	0.94	
	1948-1957; age 65-74	5.17	(3.34, 7.00)	0.93	
	1938-1948; age 75-84	4.32	(2.10, 6.55)	1.14	
	1938 or earlier; 85 or older	2.42	(-0.09, 4.92)	1.28	
Gender	(Ref: Male)				2.69e-11
	Female	-2.04	(-2.62, -1.46)	0.30	
	Other	1.66	(-4.39, 7.72)	3.09	
Religious affiliation	(Ref: No religion/Atheist/Agnostic)				0.570
	Buddhism	0.32	(-0.30, 0.94)	0.32	
	Collapsed affiliations with prevalence<3%	0.00	(-1.44, 1.44)	0.74	
Race/ethnicity	(Ref: Plurality group)				

178. Table S178. Sensitivity to unmeasured confounding of childhood predictors in Japan conditional on smokers

Variable	Category	E-value for Estimate	E-value for 95% CI
Relationship with mother	(Ref: Very bad/somewhat bad)		
	Very good/somewhat good	1.31	1.00
Relationship with father	(Ref: Very bad/somewhat bad)		
	Very good/somewhat good	1.46	1.18
Parent marital status	(Ref: Parents married)		
	Divorced	1.46	1.00
	Parents were never married	1.55	1.00
	One or both parents had died	1.13	1.00
Subjective financial status of family growing up	(Ref: Got by)		
	Lived comfortably	1.07	1.00
	Found it difficult	1.16	1.00
	Found it very difficult	1.34	1.00
Abuse	(Ref: No)		
	Yes	1.16	1.00
Outsider growing up	(Ref: No)		
	Yes	1.43	1.00
Self-rated health growing up	(Ref: Good)		
	Excellent	1.58	1.28
	Very good	1.32	1.00
	Fair	1.60	1.28
	Poor	1.09	1.00
Immigration status	(Ref: Born in this country)		
	Born in another country	2.28	1.52
Age 12 religious service attendance	(Ref: Never)		
	At least 1/week	1.45	1.00
	1-3/month	1.63	1.17
	< 1/month	1.39	1.11
Year of birth	(Ref: 1998-2005; current age: 18-24)		
	1988-1998; age 25-34	2.26	1.63
	1978-1988; age 35-44	3.00	2.30
	1968-1978; age 45-54	3.42	2.66
	1958-1968; age 55-64	3.81	2.97
	1948-1957; age 65-74	2.95	2.25
	1938-1948; age 75-84	2.61	1.84
	1938 or earlier; 85 or older	1.94	1.00
Gender	(Ref: Male)		
	Female	1.82	1.63
	Other	1.70	1.00
Religious affiliation	(Ref: No religion/Atheist/Agnostic)		
	Buddhism	1.23	1.00
	Collapsed affiliations with prevalence<3%	1.01	1.00
Race/ethnicity	(Ref: Plurality group)		

179. Table S179. Nationally representative descriptive statistics for Kenya conditional on smokers: Childhood predictors

Characteristic	N = 713[1]
**Relationship with mother**	
Very good	596 (84%)
Somewhat good	92 (13%)
Somewhat bad	9 (1.2%)
Very bad	3 (0.4%)
Does not apply	7 (0.9%)
(Missing)	7 (0.9%)
**Relationship with father**	
Very good	465 (65%)
Somewhat good	136 (19%)
Somewhat bad	17 (2.4%)
Very bad	20 (2.8%)
Does not apply	69 (9.6%)
(Missing)	6 (0.9%)
**Parent marital status**	
Parents married	546 (77%)
Divorced	53 (7.5%)
Parents were never married	42 (5.8%)
One or both parents had died	43 (6.0%)
(Missing)	29 (4.1%)
**Subjective financial status of family growing up**	
Lived comfortably	170 (24%)
Got by	209 (29%)
Found it difficult	268 (38%)
Found it very difficult	64 (9.0%)
(Missing)	2 (0.3%)
**Abuse**	
Yes	127 (18%)
No	581 (82%)
(Missing)	5 (0.7%)
**Outsider growing up**	
Yes	97 (14%)
No	615 (86%)
(Missing)	1 (0.1%)
**Self-rated health growing up**	
Excellent	287 (40%)
Very good	159 (22%)
Good	175 (25%)
Fair	74 (10%)
Poor	14 (1.9%)
(Missing)	3 (0.5%)
**Immigration status**	
Born in this country	707 (99%)
Born in another country	6 (0.9%)
(Missing)	0 (0%)
**Age 12 religious service attendance**	
At least 1/week	564 (79%)
1-3/month	100 (14%)
<1/month	23 (3.2%)
Never	23 (3.2%)
(Missing)	4 (0.6%)

180. Table S180. Nationally representative descriptive statistics for Kenya conditional on smokers: Demographic variables

Characteristic	N = 713[1]
**Year of birth**	
1998-2005; age 18-24	128 (18%)
1988-1998; age 25-34	183 (26%)
1978-1988; age 35-44	152 (21%)
1968-1978; age 45-54	120 (17%)
1958-1968; age 55-64	71 (9.9%)
1948-1958; age 65-74	56 (7.8%)
1938-1948; age 75-84	3 (0.5%)
1938 or earlier; age 85+	0 (0%)
(Missing)	0 (0%)
**Gender**	
Male	616 (86%)
Female	94 (13%)
Other	0 (0%)
(Missing)	3 (0.4%)
**Religious affiliation**	
Christianity	613 (86%)
Islam	80 (11%)
Hinduism	0 (0%)
Buddhism	0 (0%)
Judaism	0 (0%)
Sikhism	0 (0%)
Baha'i	0 (0%)
Jainism	0 (0%)
Shinto	0 (0%)
Taoism	0 (0%)
Confucianism	0 (0%)
Primal, Animist, or Folk religion	1 (0.2%)
Spiritism	0 (0%)
Umbanda, Candomble, and other African-derived religions	0 (0%)
Chinese folk/traditional religion	0 (0%)
Some other religion	0 (0%)
No religion/Atheist/Agnostic	18 (2.5%)
(Missing)	0 (0%)
**Race/Ethnicity**	
Embu	9 (1.3%)
Kalenjin	61 (8.6%)
Kamba	121 (17%)
Kenyan Somali/Somali	35 (5.0%)
Kikuyu	198 (28%)
Kisii	23 (3.2%)
Luhya	69 (9.6%)
Luo	46 (6.4%)
Maasai	7 (1.0%)
Meru	65 (9.2%)
Miji Kenda tribes	47 (6.6%)
Other	29 (4.1%)
(Missing)	2 (0.3%)

[1]n (%)

181. Table S181. Childhood predictors regression for Kenya conditional on smokers

Variable	Category	Est	95% CI	SE	Global p-value
Relationship with mother	(Ref: Very bad/somewhat bad)				0.019
	Very good/somewhat good	2.61	(0.43, 4.78)	1.11	
Relationship with father	(Ref: Very bad/somewhat bad)				0.249
	Very good/somewhat good	1.63	(-1.14, 4.39)	1.41	
Parent marital status	(Ref: Parents married)				0.625
	Divorced	0.63	(-1.63, 2.88)	1.15	
	Parents were never married	1.01	(-1.36, 3.37)	1.21	
	One or both parents had died	2.14	(-1.93, 6.21)	2.08	
Subjective financial status of family growing up	(Ref: Got by)				0.515
	Lived comfortably	0.59	(-1.29, 2.47)	0.96	
	Found it difficult	0.75	(-0.61, 2.11)	0.69	
	Found it very difficult	-0.47	(-2.32, 1.38)	0.94	
Abuse	(Ref: No)				0.412
	Yes	0.64	(-0.95, 2.23)	0.81	
Outsider growing up	(Ref: No)				0.040
	Yes	1.75	(0.03, 3.47)	0.87	
Self-rated health growing up	(Ref: Good)				0.040
	Excellent	1.05	(-0.58, 2.67)	0.83	
	Very good	-0.77	(-2.13, 0.58)	0.69	
	Fair	-0.84	(-2.53, 0.85)	0.86	
	Poor	-0.59	(-3.37, 2.19)	1.42	
Immigration status	(Ref: Born in this country)				0.034
	Born in another country	-3.00	(-5.85, -0.15)	1.45	
Age 12 religious service attendance	(Ref: Never)				0.833
	At least 1/week	1.48	(-3.77, 6.73)	2.68	
	1-3/month	1.27	(-4.17, 6.70)	2.77	
	< 1/month	0.03	(-5.64, 5.70)	2.89	
Year of birth	(Ref: 1998-2005; current age: 18-24)				0.146
	1988-1998; age 25-34	0.16	(-1.87, 2.19)	1.03	
	1978-1988; age 35-44	1.25	(-0.75, 3.26)	1.02	
	1968-1978; age 45-54	1.89	(-0.07, 3.84)	1.00	
	1958-1968; age 55-64	2.27	(-0.11, 4.65)	1.21	
	1948-1957; age 65-74	-0.37	(-2.67, 1.93)	1.17	
	1938-1948; age 75-84	-1.15	(-4.36, 2.07)	1.64	
Gender	(Ref: Male)				0.323
	Female	1.98	(-2.24, 6.19)	2.14	
Religious affiliation	(Ref: Christianity)				0.313
	Islam	0.14	(-1.52, 1.80)	0.84	
	Collapsed affiliations with prevalence<3%	4.30	(-1.47, 10.07)	2.94	
Race/ethnicity	(Ref: Plurality group)				0.103
	Non-plurality groups	1.09	(-0.23, 2.41)	0.67	

182. Table S182. Sensitivity to unmeasured confounding of childhood predictors in Kenya conditional on smokers

Variable	Category	E-value for Estimate	E-value for 95% CI
Relationship with mother	(Ref: Very bad/somewhat bad)		
	Very good/somewhat good	2.22	1.32
Relationship with father	(Ref: Very bad/somewhat bad)		
	Very good/somewhat good	1.81	1.00
Parent marital status	(Ref: Parents married)		
	Divorced	1.40	1.00
	Parents were never married	1.56	1.00
	One or both parents had died	2.02	1.00
Subjective financial status of family growing up	(Ref: Got by)		
	Lived comfortably	1.39	1.00
	Found it difficult	1.46	1.00
	Found it very difficult	1.33	1.00
Abuse	(Ref: No)		
	Yes	1.41	1.00
Outsider growing up	(Ref: No)		
	Yes	1.86	1.08
Self-rated health growing up	(Ref: Good)		
	Excellent	1.58	1.00
	Very good	1.47	1.00
	Fair	1.49	1.00
	Poor	1.39	1.00
Immigration status	(Ref: Born in this country)		
	Born in another country	2.39	1.18
Age 12 religious service attendance	(Ref: Never)		
	At least 1/week	1.75	1.00
	1-3/month	1.67	1.00
	< 1/month	1.07	1.00
Year of birth	(Ref: 1998-2005; current age: 18-24)		
	1988-1998; age 25-34	1.17	1.00
	1978-1988; age 35-44	1.66	1.00
	1968-1978; age 45-54	1.92	1.00
	1958-1968; age 55-64	2.08	1.00
	1948-1957; age 65-74	1.29	1.00
	1938-1948; age 75-84	1.62	1.00
Gender	(Ref: Male)		
	Female	1.95	1.00
Religious affiliation	(Ref: Christianity)		
	Islam	1.16	1.00
	Collapsed affiliations with prevalence<3%	3.02	1.00
Race/ethnicity	(Ref: Plurality group)		
	Non-plurality groups	1.60	1.00

183. Table S183. Nationally representative descriptive statistics for Mexico conditional on smokers: Childhood predictors

Characteristic	N = 1,438[1]
**Relationship with mother**	
Very good	1,011 (70%)
Somewhat good	292 (20%)
Somewhat bad	45 (3.1%)
Very bad	25 (1.7%)
Does not apply	48 (3.3%)
(Missing)	18 (1.2%)
**Relationship with father**	
Very good	779 (54%)
Somewhat good	380 (26%)
Somewhat bad	83 (5.8%)
Very bad	72 (5.0%)
Does not apply	107 (7.5%)
(Missing)	16 (1.1%)
**Parent marital status**	
Parents married	939 (65%)
Divorced	117 (8.1%)
Parents were never married	207 (14%)
One or both parents had died	49 (3.4%)
(Missing)	125 (8.7%)
**Subjective financial status of family growing up**	
Lived comfortably	538 (37%)
Got by	484 (34%)
Found it difficult	349 (24%)
Found it very difficult	56 (3.9%)
(Missing)	11 (0.7%)
**Abuse**	
Yes	218 (15%)
No	1,130 (79%)
(Missing)	89 (6.2%)
**Outsider growing up**	
Yes	206 (14%)
No	1,203 (84%)
(Missing)	29 (2.0%)
**Self-rated health growing up**	
Excellent	555 (39%)
Very good	348 (24%)
Good	369 (26%)
Fair	131 (9.1%)
Poor	33 (2.3%)
(Missing)	3 (0.2%)
**Immigration status**	
Born in this country	1,352 (94%)
Born in another country	39 (2.7%)
(Missing)	47 (3.3%)
**Age 12 religious service attendance**	
At least 1/week	534 (37%)
1-3/month	298 (21%)
<1/month	322 (22%)
Never	273 (19%)
(Missing)	12 (0.9%)

184. Table S184. Nationally representative descriptive statistics for Mexico conditional on smokers: Demographic variables

Characteristic	N = 1,438[1]
**Year of birth**	
1998-2005; age 18-24	261 (18%)
1988-1998; age 25-34	381 (26%)
1978-1988; age 35-44	324 (23%)
1968-1978; age 45-54	198 (14%)
1958-1968; age 55-64	150 (10%)
1948-1958; age 65-74	101 (7.0%)
1938-1948; age 75-84	18 (1.3%)
1938 or earlier; age 85+	6 (0.4%)
(Missing)	0 (0%)
**Gender**	
Male	953 (66%)
Female	480 (33%)
Other	1 (<0.1%)
(Missing)	4 (0.3%)
**Religious affiliation**	
Christianity	1,306 (91%)
Islam	0 (<0.1%)
Hinduism	0 (0%)
Buddhism	1 (<0.1%)
Judaism	2 (0.2%)
Sikhism	2 (0.1%)
Baha'i	0 (0%)
Jainism	0 (0%)
Shinto	1 (<0.1%)
Taoism	4 (0.3%)
Confucianism	0 (0%)
Primal, Animist, or Folk religion	2 (0.1%)
Spiritism	0 (0%)
Umbanda, Candomble, and other African-derived religions	0 (0%)
Chinese folk/traditional religion	0 (0%)
Some other religion	2 (0.1%)
No religion/Atheist/Agnostic	100 (7.0%)
(Missing)	17 (1.2%)
**Race/Ethnicity**	
Black	28 (1.9%)
Indigenous	109 (7.5%)
Mestizo	722 (50%)
Mulatto	27 (1.9%)
Other	73 (5.1%)
White	294 (20%)
(Missing)	186 (13%)

[1]n (%)

185. Table S185. Childhood predictors regression for Mexico conditional on smokers

Variable	Category	Est	95% CI	SE	Global p-value
Relationship with mother	(Ref: Very bad/somewhat bad)				0.614
	Very good/somewhat good	-0.47	(-2.75, 1.81)	1.16	
Relationship with father	(Ref: Very bad/somewhat bad)				0.283
	Very good/somewhat good	0.55	(-0.60, 1.70)	0.58	
Parent marital status	(Ref: Parents married)				0.741
	Divorced	-0.52	(-1.99, 0.94)	0.75	
	Parents were never married	-0.47	(-1.71, 0.78)	0.63	
	One or both parents had died	-0.37	(-3.11, 2.37)	1.37	
Subjective financial status of family growing up	(Ref: Got by)				0.305
	Lived comfortably	-0.34	(-1.99, 1.32)	0.84	
	Found it difficult	-1.20	(-2.95, 0.55)	0.89	
	Found it very difficult	-1.31	(-3.38, 0.76)	1.05	
Abuse	(Ref: No)				0.686
	Yes	0.21	(-0.95, 1.37)	0.59	
Outsider growing up	(Ref: No)				0.346
	Yes	-0.51	(-1.59, 0.57)	0.55	
Self-rated health growing up	(Ref: Good)				0.554
	Excellent	0.51	(-0.47, 1.50)	0.50	
	Very good	0.86	(-0.33, 2.05)	0.61	
	Fair	1.10	(-2.78, 4.97)	1.98	
	Poor	0.57	(-1.34, 2.48)	0.97	
Immigration status	(Ref: Born in this country)				0.020
	Born in another country	3.79	(0.55, 7.03)	1.65	
Age 12 religious service attendance	(Ref: Never)				0.157
	At least 1/week	-0.82	(-2.56, 0.93)	0.89	
	1-3/month	-1.78	(-3.58, 0.03)	0.92	
	< 1/month	-1.68	(-3.66, 0.30)	1.01	
Year of birth	(Ref: 1998-2005; current age: 18-24)				0.032
	1988-1998; age 25-34	0.19	(-1.53, 1.91)	0.88	
	1978-1988; age 35-44	1.04	(-0.97, 3.06)	1.03	
	1968-1978; age 45-54	1.80	(-0.54, 4.14)	1.19	
	1958-1968; age 55-64	1.45	(-0.78, 3.69)	1.14	
	1948-1957; age 65-74	3.52	(0.75, 6.29)	1.41	
	1938-1948; age 75-84	5.56	(0.83, 10.29)	2.41	
	1938 or earlier; 85 or older	-0.28	(-3.74, 3.18)	1.73	
Gender	(Ref: Male)				1.09e-04
	Female	-1.79	(-2.65, -0.93)	0.44	
	Other	2.33	(-5.06, 9.72)	3.77	
Religious affiliation	(Ref: No religion/Atheist/Agnostic)				0.316
	Christianity	-3.40	(-8.21, 1.40)	2.45	
	Collapsed affiliations with prevalence<3%	-3.69	(-8.51, 1.12)	2.46	
Race/ethnicity	(Ref: Plurality group)				0.380
	Non-plurality groups	0.48	(-1.22, 2.18)	0.84	

186. Table S186. Sensitivity to unmeasured confounding of childhood predictors in Mexico conditional on smokers

Variable	Category	E-value for Estimate	E-value for 95% CI
Relationship with mother	(Ref: Very bad/somewhat bad)		
	Very good/somewhat good	1.32	1.00
Relationship with father	(Ref: Very bad/somewhat bad)		
	Very good/somewhat good	1.35	1.00
Parent marital status	(Ref: Parents married)		
	Divorced	1.34	1.00
	Parents were never married	1.32	1.00
	One or both parents had died	1.27	1.00
Subjective financial status of family growing up	(Ref: Got by)		
	Lived comfortably	1.26	1.00
	Found it difficult	1.60	1.00
	Found it very difficult	1.64	1.00
Abuse	(Ref: No)		
	Yes	1.19	1.00
Outsider growing up	(Ref: No)		
	Yes	1.33	1.00
Self-rated health growing up	(Ref: Good)		
	Excellent	1.34	1.00
	Very good	1.47	1.00
	Fair	1.56	1.00
	Poor	1.36	1.00
Immigration status	(Ref: Born in this country)		
	Born in another country	2.62	1.35
Age 12 religious service attendance	(Ref: Never)		
	At least 1/week	1.46	1.00
	1-3/month	1.82	1.00
	< 1/month	1.78	1.00
Year of birth	(Ref: 1998-2005; current age: 18-24)		
	1988-1998; age 25-34	1.18	1.00
	1978-1988; age 35-44	1.54	1.00
	1968-1978; age 45-54	1.82	1.00
	1958-1968; age 55-64	1.70	1.00
	1948-1957; age 65-74	2.50	1.43
	1938-1948; age 75-84	3.47	1.47
	1938 or earlier; 85 or older	1.23	1.00
Gender	(Ref: Male)		
	Female	1.82	1.50
	Other	2.02	1.00
Religious affiliation	(Ref: No religion/Atheist/Agnostic)		
	Christianity	2.45	1.00
	Collapsed affiliations with prevalence<3%	2.58	1.00
Race/ethnicity	(Ref: Plurality group)		
	Non-plurality groups	1.32	1.00

187. Table S187. Nationally representative descriptive statistics for Nigeria conditional on smokers: Childhood predictors

Characteristic	N = 368[1]
**Relationship with mother**	
Very good	311 (85%)
Somewhat good	43 (12%)
Somewhat bad	4 (1.1%)
Very bad	2 (0.6%)
Does not apply	8 (2.1%)
(Missing)	0 (0%)
**Relationship with father**	
Very good	293 (80%)
Somewhat good	51 (14%)
Somewhat bad	12 (3.4%)
Very bad	4 (1.0%)
Does not apply	6 (1.6%)
(Missing)	1 (0.3%)
**Parent marital status**	
Parents married	280 (76%)
Divorced	12 (3.4%)
Parents were never married	14 (3.7%)
One or both parents had died	55 (15%)
(Missing)	7 (1.9%)
**Subjective financial status of family growing up**	
Lived comfortably	97 (26%)
Got by	116 (32%)
Found it difficult	113 (31%)
Found it very difficult	41 (11%)
(Missing)	1 (0.2%)
**Abuse**	
Yes	73 (20%)
No	273 (74%)
(Missing)	22 (5.9%)
**Outsider growing up**	
Yes	46 (12%)
No	317 (86%)
(Missing)	6 (1.7%)
**Self-rated health growing up**	
Excellent	125 (34%)
Very good	140 (38%)
Good	82 (22%)
Fair	12 (3.4%)
Poor	8 (2.3%)
(Missing)	1 (0.2%)
**Immigration status**	
Born in this country	367 (100%)
Born in another country	1 (0.3%)
(Missing)	0 (0%)
**Age 12 religious service attendance**	
At least 1/week	311 (84%)
1-3/month	44 (12%)
<1/month	9 (2.4%)
Never	2 (0.5%)
(Missing)	2 (0.7%)

188. Table S188. Nationally representative descriptive statistics for Nigeria conditional on smokers: Demographic variables

Characteristic	N = 368[1]
**Year of birth**	
1998-2005; age 18-24	60 (16%)
1988-1998; age 25-34	110 (30%)
1978-1988; age 35-44	116 (32%)
1968-1978; age 45-54	50 (14%)
1958-1968; age 55-64	21 (5.8%)
1948-1958; age 65-74	1 (0.4%)
1938-1948; age 75-84	7 (1.8%)
1938 or earlier; age 85+	2 (0.5%)
(Missing)	0 (0%)
**Gender**	
Male	310 (84%)
Female	58 (16%)
Other	0 (0%)
(Missing)	0 (0%)
**Religious affiliation**	
Christianity	169 (46%)
Islam	199 (54%)
Hinduism	0 (0%)
Buddhism	0 (0%)
Judaism	0 (0%)
Sikhism	0 (0%)
Baha'i	0 (0%)
Jainism	0 (0%)
Shinto	0 (0%)
Taoism	0 (0%)
Confucianism	0 (0.1%)
Primal, Animist, or Folk religion	0 (0.1%)
Spiritism	0 (0%)
Umbanda, Candomble, and other African-derived religions	0 (0%)
Chinese folk/traditional religion	0 (0%)
Some other religion	0 (0%)
No religion/Atheist/Agnostic	0 (0%)
(Missing)	0 (0%)
**Race/Ethnicity**	
Edo	8 (2.1%)
Efik	2 (0.6%)
Fulani	15 (4.1%)
Hausa	150 (41%)
Ibibio	8 (2.1%)
Idoma	5 (1.4%)
Igala	3 (0.9%)
Igbo (Ibo)	49 (13%)
Ijaw	2 (0.5%)
Kanuri	5 (1.4%)
Other	53 (14%)
Tiv	16 (4.2%)
Urhobo	0 (0.1%)
Yoruba	53 (14%)
(Missing)	0 (0%)

[1]n (%)

189. Table S189. Childhood predictors regression for Nigeria conditional on smokers

Variable	Category	Est	95% CI	SE	Global p-value
Relationship with mother	(Ref: Very bad/somewhat bad)				0.228
	Very good/somewhat good	-2.47	(-6.53, 1.59)	2.07	
Relationship with father	(Ref: Very bad/somewhat bad)				0.216
	Very good/somewhat good	1.32	(-0.88, 3.51)	1.12	
Parent marital status	(Ref: Parents married)				0.465
	Divorced	-1.99	(-5.74, 1.77)	1.92	
	Parents were never married	3.99	(-5.02, 13.00)	4.60	
	One or both parents had died	0.20	(-3.88, 4.29)	1.96	
Subjective financial status of family growing up	(Ref: Got by)				0.401
	Lived comfortably	-2.77	(-6.18, 0.63)	1.74	
	Found it difficult	-2.16	(-5.96, 1.65)	1.94	
	Found it very difficult	-2.83	(-6.62, 0.96)	1.93	
Abuse	(Ref: No)				0.340
	Yes	-1.28	(-3.94, 1.38)	1.36	
Outsider growing up	(Ref: No)				0.766
	Yes	-0.72	(-6.10, 4.65)	2.74	
Self-rated health growing up	(Ref: Good)				0.365
	Excellent	-0.05	(-4.20, 4.10)	2.12	
	Very good	-1.15	(-4.18, 1.87)	1.54	
	Fair	-3.01	(-6.33, 0.31)	1.70	
	Poor	2.46	(-5.33, 10.26)	3.98	
Immigration status	(Ref: Born in this country)				0.000
	Born in another country	14.97	(12.57, 17.38)	1.23	
Age 12 religious service attendance	(Ref: Never)				0.348
	At least 1/week	5.41	(-2.95, 13.77)	4.26	
	1-3/month	7.73	(-0.96, 16.43)	4.44	
	< 1/month	5.78	(-2.30, 13.86)	4.12	
Year of birth	(Ref: 1998-2005; current age: 18-24)				3.44e-07
	1988-1998; age 25-34	1.71	(-1.27, 4.69)	1.52	
	1978-1988; age 35-44	2.85	(-0.14, 5.83)	1.52	
	1968-1978; age 45-54	2.01	(-2.00, 6.03)	2.05	
	1958-1968; age 55-64	-0.41	(-2.96, 2.15)	1.30	
	1948-1957; age 65-74	6.32	(2.15, 10.49)	2.13	
	1938-1948; age 75-84	-0.60	(-3.88, 2.69)	1.66	
	1938 or earlier; 85 or older	-0.51	(-7.64, 6.62)	3.64	
Gender	(Ref: Male)				0.303
	Female	3.57	(-3.21, 10.36)	3.46	
Religious affiliation	(Ref: Christianity)				0.617
	Christianity	-1.67	(-6.46, 3.11)	2.44	
	Collapsed affiliations with prevalence<3%	3.63	(-10.36, 17.62)	7.14	
Race/ethnicity	(Ref: Plurality group)				0.856
	Non-plurality groups	0.18	(-4.41, 4.77)	2.34	

190. Table S190. Sensitivity to unmeasured confounding of childhood predictors in Nigeria conditional on smokers

Variable	Category	E-value for Estimate	E-value for 95% CI
Relationship with mother	(Ref: Very bad/somewhat bad)		
	Very good/somewhat good	1.78	1.00
Relationship with father	(Ref: Very bad/somewhat bad)		
	Very good/somewhat good	1.49	1.00
Parent marital status	(Ref: Parents married)		
	Divorced	1.66	1.00
	Parents were never married	2.18	1.00
	One or both parents had died	1.15	1.00
Subjective financial status of family growing up	(Ref: Got by)		
	Lived comfortably	1.86	1.00
	Found it difficult	1.70	1.00
	Found it very difficult	1.87	1.00
Abuse	(Ref: No)		
	Yes	1.48	1.00
Outsider growing up	(Ref: No)		
	Yes	1.33	1.00
Self-rated health growing up	(Ref: Good)		
	Excellent	1.07	1.00
	Very good	1.45	1.00
	Fair	1.92	1.00
	Poor	1.78	1.00
Immigration status	(Ref: Born in this country)		
	Born in another country	6.78	5.39
Age 12 religious service attendance	(Ref: Never)		
	At least 1/week	2.58	1.00
	1-3/month	3.32	1.00
	< 1/month	2.69	1.00
Year of birth	(Ref: 1998-2005; current age: 18-24)		
	1988-1998; age 25-34	1.59	1.00
	1978-1988; age 35-44	1.88	1.00
	1968-1978; age 45-54	1.67	1.00
	1958-1968; age 55-64	1.23	1.00
	1948-1957; age 65-74	2.85	1.70
	1938-1948; age 75-84	1.29	1.00
	1938 or earlier; 85 or older	1.26	1.00
Gender	(Ref: Male)		
	Female	2.07	1.00
Religious affiliation	(Ref: Christianity)		
	Christianity	1.58	1.00
	Collapsed affiliations with prevalence<3%	2.08	1.00
Race/ethnicity	(Ref: Plurality group)		
	Non-plurality groups	1.14	1.00

191. Table S191. Nationally representative descriptive statistics for Philippines conditional on smokers: Childhood predictors

Characteristic	N = 1,214[1]
**Relationship with mother**	
Very good	721 (59%)
Somewhat good	425 (35%)
Somewhat bad	31 (2.5%)
Very bad	6 (0.5%)
Does not apply	17 (1.4%)
(Missing)	14 (1.1%)
**Relationship with father**	
Very good	783 (64%)
Somewhat good	334 (28%)
Somewhat bad	43 (3.5%)
Very bad	11 (0.9%)
Does not apply	22 (1.8%)
(Missing)	21 (1.7%)
**Parent marital status**	
Parents married	1,015 (84%)
Divorced	15 (1.2%)
Parents were never married	142 (12%)
One or both parents had died	13 (1.1%)
(Missing)	29 (2.4%)
**Subjective financial status of family growing up**	
Lived comfortably	218 (18%)
Got by	699 (58%)
Found it difficult	219 (18%)
Found it very difficult	77 (6.4%)
(Missing)	1 (<0.1%)
**Abuse**	
Yes	110 (9.1%)
No	1,084 (89%)
(Missing)	19 (1.6%)
**Outsider growing up**	
Yes	119 (9.8%)
No	1,091 (90%)
(Missing)	4 (0.3%)
**Self-rated health growing up**	
Excellent	229 (19%)
Very good	115 (9.5%)
Good	508 (42%)
Fair	304 (25%)
Poor	59 (4.8%)
(Missing)	0 (0%)
**Immigration status**	
Born in this country	1,211 (100%)
Born in another country	3 (0.2%)
(Missing)	0 (0%)
**Age 12 religious service attendance**	
At least 1/week	507 (42%)
1-3/month	383 (32%)
<1/month	253 (21%)
Never	62 (5.1%)
(Missing)	10 (0.8%)

192. Table S192. Nationally representative descriptive statistics for Philippines conditional on smokers: Demographic variables

Characteristic	N = 1,214[1]
**Year of birth**	
1998-2005; age 18-24	195 (16%)
1988-1998; age 25-34	357 (29%)
1978-1988; age 35-44	284 (23%)
1968-1978; age 45-54	186 (15%)
1958-1968; age 55-64	128 (11%)
1948-1958; age 65-74	54 (4.4%)
1938-1948; age 75-84	9 (0.7%)
1938 or earlier; age 85+	0 (0%)
(Missing)	0 (0%)
**Gender**	
Male	1,011 (83%)
Female	198 (16%)
Other	1 (<0.1%)
(Missing)	4 (0.3%)
**Religious affiliation**	
Christianity	1,136 (94%)
Islam	65 (5.4%)
Hinduism	0 (0%)
Buddhism	0 (0%)
Judaism	0 (0%)
Sikhism	0 (0%)
Baha'i	0 (0%)
Jainism	0 (0%)
Shinto	0 (0%)
Taoism	0 (0%)
Confucianism	0 (0%)
Primal, Animist, or Folk religion	4 (0.3%)
Spiritism	0 (0%)
Umbanda, Candomble, and other African-derived religions	0 (0%)
Chinese folk/traditional religion	0 (0%)
Some other religion	5 (0.4%)
No religion/Atheist/Agnostic	2 (0.2%)
(Missing)	1 (<0.1%)
**Race/Ethnicity**	
Aeta	0 (0%)
Badjao	0 (0%)
Bicolano/Bikolano	70 (5.8%)
Cebuano	153 (13%)
Chinese-Filipino	0 (0%)
Igorot	9 (0.8%)
Ilocano/Ilokano	97 (8.0%)
Ilonggo/Hiligaynon	109 (9.0%)
Kapampangan	31 (2.6%)
Maguindanaoan	14 (1.2%)
Mangyan	0 (0%)
Maranao	10 (0.8%)
Masbateno	17 (1.4%)
Other	43 (3.6%)
Pangasinense	29 (2.4%)
Tagalog	436 (36%)
Tausug	25 (2.1%)
Visayan/Bisaya	110 (9.1%)
Waray	42 (3.5%)
Zamboangueno	16 (1.3%)
(Missing)	0 (0%)

193. Table S193. Childhood predictors regression for Philippines conditional on smokers

Variable	Category	Est	95% CI	SE	Global p-value
Relationship with mother	(Ref: Very bad/somewhat bad)				0.084
	Very good/somewhat good	-1.39	(-2.98, 0.19)	0.81	
Relationship with father	(Ref: Very bad/somewhat bad)				0.633
	Very good/somewhat good	-0.36	(-1.94, 1.22)	0.81	
Parent marital status	(Ref: Parents married)				0.002
	Divorced	-2.45	(-3.75, -1.15)	0.66	
	Parents were never married	0.18	(-1.33, 1.69)	0.76	
	One or both parents had died	1.21	(-2.84, 5.26)	2.06	
Subjective financial status of family growing up	(Ref: Got by)				0.326
	Lived comfortably	-0.64	(-1.79, 0.51)	0.59	
	Found it difficult	-1.04	(-2.27, 0.20)	0.63	
	Found it very difficult	0.13	(-2.44, 2.70)	1.31	
Abuse	(Ref: No)				0.395
	Yes	1.04	(-1.50, 3.58)	1.29	
Outsider growing up	(Ref: No)				0.465
	Yes	0.55	(-0.94, 2.03)	0.76	
Self-rated health growing up	(Ref: Good)				0.166
	Excellent	0.32	(-1.15, 1.79)	0.75	
	Very good	1.34	(-1.21, 3.90)	1.31	
	Fair	1.36	(0.20, 2.51)	0.59	
	Poor	1.51	(-0.57, 3.60)	1.06	
Immigration status	(Ref: Born in this country)				0.707
	Born in another country	1.19	(-5.02, 7.40)	3.17	
Age 12 religious service attendance	(Ref: Never)				0.211
	At least 1/week	-1.85	(-4.81, 1.11)	1.51	
	1-3/month	-0.91	(-3.82, 1.99)	1.48	
	< 1/month	-0.76	(-3.66, 2.14)	1.48	
Year of birth	(Ref: 1998-2005; current age: 18-24)				0.002
	1988-1998; age 25-34	0.40	(-1.48, 2.29)	0.96	
	1978-1988; age 35-44	2.28	(0.46, 4.10)	0.93	
	1968-1978; age 45-54	2.48	(0.49, 4.48)	1.02	
	1958-1968; age 55-64	2.73	(0.58, 4.88)	1.10	
	1948-1957; age 65-74	1.79	(-1.12, 4.70)	1.48	
	1938-1948; age 75-84	5.24	(0.47, 10.00)	2.43	
Gender	(Ref: Male)				3.29e-13
	Female	-3.68	(-4.58, -2.78)	0.46	
	Other	-3.93	(-7.14, -0.72)	1.63	
Religious affiliation	(Ref: Christianity)				0.257
	Islam	2.62	(-1.30, 6.55)	2.00	
	Collapsed affiliations with prevalence<3%	-1.36	(-4.40, 1.67)	1.55	
Race/ethnicity	(Ref: Plurality group)				0.166
	Non-plurality groups	-0.77	(-1.87, 0.33)	0.56	

194. Table S194. Sensitivity to unmeasured confounding of childhood predictors in Philippines conditional on smokers

Variable	Category	E-value for Estimate	E-value for 95% CI
Relationship with mother	(Ref: Very bad/somewhat bad)		
	Very good/somewhat good	1.71	1.00
Relationship with father	(Ref: Very bad/somewhat bad)		
	Very good/somewhat good	1.28	1.00
Parent marital status	(Ref: Parents married)		
	Divorced	2.13	1.61
	Parents were never married	1.18	1.00
	One or both parents had died	1.64	1.00
Subjective financial status of family growing up	(Ref: Got by)		
	Lived comfortably	1.41	1.00
	Found it difficult	1.57	1.00
	Found it very difficult	1.15	1.00
Abuse	(Ref: No)		
	Yes	1.57	1.00
Outsider growing up	(Ref: No)		
	Yes	1.36	1.00
Self-rated health growing up	(Ref: Good)		
	Excellent	1.26	1.00
	Very good	1.69	1.00
	Fair	1.69	1.20
	Poor	1.76	1.00
Immigration status	(Ref: Born in this country)		
	Born in another country	1.63	1.00
Age 12 religious service attendance	(Ref: Never)		
	At least 1/week	1.89	1.00
	1-3/month	1.52	1.00
	< 1/month	1.45	1.00
Year of birth	(Ref: 1998-2005; current age: 18-24)		
	1988-1998; age 25-34	1.30	1.00
	1978-1988; age 35-44	2.07	1.33
	1968-1978; age 45-54	2.15	1.34
	1958-1968; age 55-64	2.25	1.38
	1948-1957; age 65-74	1.87	1.00
	1938-1948; age 75-84	3.48	1.34
Gender	(Ref: Male)		
	Female	2.68	2.27
	Other	2.80	1.45
Religious affiliation	(Ref: Christianity)		
	Islam	2.21	1.00
	Collapsed affiliations with prevalence<3%	1.70	1.00
Race/ethnicity	(Ref: Plurality group)		
	Non-plurality groups	1.46	1.00

195. Table S195. Nationally representative descriptive statistics for Poland conditional on smokers: Childhood predictors

Characteristic	N = 3,230[1]
**Relationship with mother**	
Very good	1,262 (39%)
Somewhat good	1,716 (53%)
Somewhat bad	147 (4.6%)
Very bad	14 (0.4%)
Does not apply	45 (1.4%)
(Missing)	47 (1.4%)
**Relationship with father**	
Very good	1,053 (33%)
Somewhat good	1,718 (53%)
Somewhat bad	231 (7.1%)
Very bad	30 (0.9%)
Does not apply	142 (4.4%)
(Missing)	56 (1.7%)
**Parent marital status**	
Parents married	2,588 (80%)
Divorced	275 (8.5%)
Parents were never married	103 (3.2%)
One or both parents had died	97 (3.0%)
(Missing)	167 (5.2%)
**Subjective financial status of family growing up**	
Lived comfortably	457 (14%)
Got by	1,880 (58%)
Found it difficult	653 (20%)
Found it very difficult	201 (6.2%)
(Missing)	38 (1.2%)
**Abuse**	
Yes	121 (3.7%)
No	3,089 (96%)
(Missing)	20 (0.6%)
**Outsider growing up**	
Yes	181 (5.6%)
No	2,910 (90%)
(Missing)	139 (4.3%)
**Self-rated health growing up**	
Excellent	852 (26%)
Very good	1,501 (46%)
Good	655 (20%)
Fair	163 (5.1%)
Poor	50 (1.6%)
(Missing)	7 (0.2%)
**Immigration status**	
Born in this country	3,158 (98%)
Born in another country	59 (1.8%)
(Missing)	13 (0.4%)
**Age 12 religious service attendance**	
At least 1/week	1,192 (37%)
1-3/month	900 (28%)
<1/month	822 (25%)
Never	173 (5.3%)
(Missing)	144 (4.5%)

196. Table S196. Nationally representative descriptive statistics for Poland conditional on smokers: Demographic variables

Characteristic	N = 3,230[1]
**Year of birth**	
1998-2005; age 18-24	265 (8.2%)
1988-1998; age 25-34	619 (19%)
1978-1988; age 35-44	731 (23%)
1968-1978; age 45-54	636 (20%)
1958-1968; age 55-64	587 (18%)
1948-1958; age 65-74	344 (11%)
1938-1948; age 75-84	41 (1.3%)
1938 or earlier; age 85+	6 (0.2%)
(Missing)	0 (0%)
**Gender**	
Male	2,024 (63%)
Female	1,197 (37%)
Other	3 (<0.1%)
(Missing)	6 (0.2%)
**Religious affiliation**	
Christianity	3,029 (94%)
Islam	0 (0%)
Hinduism	0 (0%)
Buddhism	0 (0%)
Judaism	0 (0%)
Sikhism	0 (0%)
Baha'i	0 (0%)
Jainism	0 (0%)
Shinto	0 (0%)
Taoism	0 (0%)
Confucianism	0 (0%)
Primal, Animist, or Folk religion	2 (<0.1%)
Spiritism	0 (0%)
Umbanda, Candomble, and other African-derived religions	0 (0%)
Chinese folk/traditional religion	0 (0%)
Some other religion	0 (0%)
No religion/Atheist/Agnostic	195 (6.0%)
(Missing)	5 (0.1%)
**Race/Ethnicity**	
Belarussian	2 (<0.1%)
German	0 (0%)
Kashubians	1 (<0.1%)
Other	1 (<0.1%)
Polish	3,191 (99%)
Silesia	5 (0.2%)
Ukrainian	26 (0.8%)
(Missing)	3 (0.1%)

[1]n (%)

197. Table S197. Childhood predictors regression for Poland conditional on smokers

Variable	Category	Est	95% CI	SE	Global p-value
Relationship with mother	(Ref: Very bad/somewhat bad)				0.051
	Very good/somewhat good	-1.59	(-3.28, 0.10)	0.86	
Relationship with father	(Ref: Very bad/somewhat bad)				0.603
	Very good/somewhat good	-0.38	(-1.82, 1.07)	0.74	
Parent marital status	(Ref: Parents married)				0.641
	Divorced	-0.41	(-1.90, 1.09)	0.74	
	Parents were never married	-0.64	(-2.26, 0.99)	0.83	
	One or both parents had died	-0.64	(-3.07, 1.79)	1.24	
Subjective financial status of family growing up	(Ref: Got by)				0.447
	Lived comfortably	-0.15	(-0.99, 0.68)	0.43	
	Found it difficult	0.79	(-1.19, 2.77)	1.01	
	Found it very difficult	1.21	(-0.46, 2.89)	0.85	
Abuse	(Ref: No)				0.013
	Yes	1.93	(0.39, 3.47)	0.79	
Outsider growing up	(Ref: No)				0.436
	Yes	-0.49	(-1.82, 0.84)	0.68	
Self-rated health growing up	(Ref: Good)				3.95e-04
	Excellent	2.08	(1.02, 3.15)	0.54	
	Very good	0.91	(-0.25, 2.06)	0.59	
	Fair	-0.61	(-2.40, 1.18)	0.91	
	Poor	-2.65	(-5.72, 0.42)	1.57	
Immigration status	(Ref: Born in this country)				0.848
	Born in another country	-0.24	(-2.70, 2.23)	1.26	
Age 12 religious service attendance	(Ref: Never)				0.394
	At least 1/week	-1.01	(-2.74, 0.71)	0.88	
	1-3/month	-1.34	(-2.98, 0.30)	0.84	
	< 1/month	-1.03	(-2.69, 0.62)	0.85	
Year of birth	(Ref: 1998-2005; current age: 18-24)				1.03e-07
	1988-1998; age 25-34	1.88	(0.66, 3.10)	0.62	
	1978-1988; age 35-44	2.66	(1.50, 3.82)	0.59	
	1968-1978; age 45-54	2.95	(1.71, 4.20)	0.64	
	1958-1968; age 55-64	5.18	(3.20, 7.16)	1.01	
	1948-1957; age 65-74	2.99	(1.10, 4.88)	0.96	
	1938-1948; age 75-84	2.45	(-1.78, 6.68)	2.16	
	1938 or earlier; 85 or older	1.12	(-3.66, 5.90)	2.44	
Gender	(Ref: Male)				5.17e-14
	Female	-2.23	(-3.39, -1.07)	0.59	
	Other	-4.68	(-5.94, -3.42)	0.64	
Religious affiliation	(Ref: No religion/Atheist/Agnostic)				0.001
	Christianity	0.17	(-1.05, 1.39)	0.62	
	Collapsed affiliations with prevalence<3%	-4.15	(-6.63, -1.68)	1.26	
Race/ethnicity	(Ref: Plurality group)				0.454
	Non-plurality groups	1.72	(-2.79, 6.23)	2.30	

198. Table S198. Sensitivity to unmeasured confounding of childhood predictors in Poland conditional on smokers

Variable	Category	E-value for Estimate	E-value for 95% CI
Relationship with mother	(Ref: Very bad/somewhat bad)		
	Very good/somewhat good	1.71	1.00
Relationship with father	(Ref: Very bad/somewhat bad)		
	Very good/somewhat good	1.27	1.00
Parent marital status	(Ref: Parents married)		
	Divorced	1.28	1.00
	Parents were never married	1.37	1.00
	One or both parents had died	1.38	1.00
Subjective financial status of family growing up	(Ref: Got by)		
	Lived comfortably	1.15	1.00
	Found it difficult	1.43	1.00
	Found it very difficult	1.58	1.00
Abuse	(Ref: No)		
	Yes	1.83	1.27
Outsider growing up	(Ref: No)		
	Yes	1.31	1.00
Self-rated health growing up	(Ref: Good)		
	Excellent	1.89	1.51
	Very good	1.47	1.00
	Fair	1.36	1.00
	Poor	2.09	1.00
Immigration status	(Ref: Born in this country)		
	Born in another country	1.20	1.00
Age 12 religious service attendance	(Ref: Never)		
	At least 1/week	1.51	1.00
	1-3/month	1.63	1.00
	< 1/month	1.52	1.00
Year of birth	(Ref: 1998-2005; current age: 18-24)		
	1988-1998; age 25-34	1.82	1.38
	1978-1988; age 35-44	2.10	1.68
	1968-1978; age 45-54	2.21	1.76
	1958-1968; age 55-64	3.13	2.30
	1948-1957; age 65-74	2.22	1.55
	1938-1948; age 75-84	2.02	1.00
	1938 or earlier; 85 or older	1.55	1.00
Gender	(Ref: Male)		
	Female	1.94	1.54
	Other	2.91	2.39
Religious affiliation	(Ref: No religion/Atheist/Agnostic)		
	Christianity	1.16	1.00
	Collapsed affiliations with prevalence<3%	2.68	1.75
Race/ethnicity	(Ref: Plurality group)		
	Non-plurality groups	1.76	1.00

199. Table S199. Nationally representative descriptive statistics for South Africa conditional on smokers: Childhood predictors

Characteristic	N = 669[1]
**Relationship with mother**	
Very good	567 (85%)
Somewhat good	58 (8.7%)
Somewhat bad	16 (2.4%)
Very bad	8 (1.2%)
Does not apply	13 (1.9%)
(Missing)	7 (1.0%)
**Relationship with father**	
Very good	415 (62%)
Somewhat good	83 (12%)
Somewhat bad	34 (5.0%)
Very bad	42 (6.2%)
Does not apply	77 (12%)
(Missing)	18 (2.7%)
**Parent marital status**	
Parents married	322 (48%)
Divorced	41 (6.1%)
Parents were never married	214 (32%)
One or both parents had died	44 (6.5%)
(Missing)	49 (7.3%)
**Subjective financial status of family growing up**	
Lived comfortably	263 (39%)
Got by	227 (34%)
Found it difficult	123 (18%)
Found it very difficult	55 (8.2%)
(Missing)	1 (<0.1%)
**Abuse**	
Yes	128 (19%)
No	519 (78%)
(Missing)	21 (3.2%)
**Outsider growing up**	
Yes	110 (16%)
No	559 (84%)
(Missing)	0 (0%)
**Self-rated health growing up**	
Excellent	335 (50%)
Very good	145 (22%)
Good	89 (13%)
Fair	65 (9.7%)
Poor	29 (4.4%)
(Missing)	6 (0.9%)
**Immigration status**	
Born in this country	648 (97%)
Born in another country	20 (3.1%)
(Missing)	0 (0%)
**Age 12 religious service attendance**	
At least 1/week	421 (63%)
1-3/month	145 (22%)
<1/month	35 (5.3%)
Never	61 (9.2%)
(Missing)	6 (0.9%)

200. Table S200. Nationally representative descriptive statistics for South Africa conditional on smokers: Demographic variables

Characteristic	N = 669[1]
**Year of birth**	
1998-2005; age 18-24	104 (16%)
1988-1998; age 25-34	196 (29%)
1978-1988; age 35-44	178 (27%)
1968-1978; age 45-54	94 (14%)
1958-1968; age 55-64	61 (9.2%)
1948-1958; age 65-74	17 (2.6%)
1938-1948; age 75-84	15 (2.2%)
1938 or earlier; age 85+	0 (0%)
(Missing)	2 (0.3%)
**Gender**	
Male	497 (74%)
Female	167 (25%)
Other	2 (0.2%)
(Missing)	3 (0.4%)
**Religious affiliation**	
Christianity	581 (87%)
Islam	28 (4.2%)
Hinduism	1 (0.1%)
Buddhism	1 (0.2%)
Judaism	0 (0%)
Sikhism	0 (0%)
Baha'i	0 (0%)
Jainism	0 (0%)
Shinto	0 (0%)
Taoism	0 (0%)
Confucianism	0 (0%)
Primal, Animist, or Folk religion	18 (2.7%)
Spiritism	0 (0%)
Umbanda, Candomble, and other African-derived religions	0 (0%)
Chinese folk/traditional religion	0 (0%)
Some other religion	2 (0.3%)
No religion/Atheist/Agnostic	34 (5.0%)
(Missing)	4 (0.6%)
**Race/Ethnicity**	
Asian/Indian	0 (0%)
Black	532 (80%)
Colored	132 (20%)
Other	0 (0%)
White	4 (0.6%)
(Missing)	0 (0%)

[1]n (%)

201. Table S201. Childhood predictors regression for South Africa conditional on smokers

Variable	Category	Est	95% CI	SE	Global p-value
Relationship with mother	(Ref: Very bad/somewhat bad)				0.145
	Very good/somewhat good	-7.54	(-17.63, 2.55)	5.15	
Relationship with father	(Ref: Very bad/somewhat bad)				0.090
	Very good/somewhat good	-2.80	(-6.04, 0.45)	1.65	
Parent marital status	(Ref: Parents married)				0.078
	Divorced	-2.30	(-4.46, -0.14)	1.10	
	Parents were never married	-1.66	(-3.43, 0.10)	0.90	
	One or both parents had died	-1.48	(-4.18, 1.23)	1.32	
Subjective financial status of family growing up	(Ref: Got by)				0.396
	Lived comfortably	0.56	(-0.52, 1.63)	0.55	
	Found it difficult	2.08	(-0.53, 4.68)	1.33	
	Found it very difficult	0.01	(-2.18, 2.20)	1.12	
Abuse	(Ref: No)				0.211
	Yes	1.29	(-0.74, 3.32)	1.03	
Outsider growing up	(Ref: No)				0.264
	Yes	1.33	(-1.02, 3.68)	1.20	
Self-rated health growing up	(Ref: Good)				0.046
	Excellent	-1.71	(-3.47, 0.05)	0.90	
	Very good	0.64	(-2.23, 3.52)	1.47	
	Fair	-1.16	(-3.40, 1.08)	1.14	
	Poor	-3.56	(-6.22, -0.89)	1.36	
Immigration status	(Ref: Born in this country)				0.754
	Born in another country	0.17	(-2.86, 3.20)	1.54	
Age 12 religious service attendance	(Ref: Never)				0.171
	At least 1/week	-0.06	(-3.51, 3.39)	1.76	
	1-3/month	-1.51	(-4.96, 1.94)	1.76	
	< 1/month	-1.91	(-5.77, 1.96)	1.97	
Year of birth	(Ref: 1998-2005; current age: 18-24)				8.31e-04
	1988-1998; age 25-34	0.68	(-0.75, 2.11)	0.73	
	1978-1988; age 35-44	2.28	(0.23, 4.34)	1.05	
	1968-1978; age 45-54	1.52	(-0.39, 3.44)	0.98	
	1958-1968; age 55-64	0.56	(-2.47, 3.59)	1.54	
	1948-1957; age 65-74	-0.24	(-5.17, 4.69)	2.23	
	1938-1948; age 75-84	-2.64	(-5.24, -0.03)	1.33	
Gender	(Ref: Male)				0.028
	Female	0.17	(-1.27, 1.60)	0.73	
	Other	-5.79	(-10.01, -1.56)	2.16	
Religious affiliation	(Ref: No religion/Atheist/Agnostic)				0.423
	Islam	-2.23	(-7.76, 3.31)	2.82	
	Christianity	0.50	(-3.72, 4.73)	2.15	
	Collapsed affiliations with prevalence<3%	0.86	(-3.98, 5.70)	2.46	
Race/ethnicity	(Ref: Plurality group)				0.005
	Non-plurality groups	4.15	(1.23, 7.07)	1.49	

202. Table S202. Sensitivity to unmeasured confounding of childhood predictors in South Africa conditional on smokers

Variable	Category	E-value for Estimate	E-value for 95% CI
Relationship with mother	(Ref: Very bad/somewhat bad)		
	Very good/somewhat good	4.71	1.00
Relationship with father	(Ref: Very bad/somewhat bad)		
	Very good/somewhat good	2.22	1.00
Parent marital status	(Ref: Parents married)		
	Divorced	2.02	1.16
	Parents were never married	1.78	1.00
	One or both parents had died	1.71	1.00
Subjective financial status of family growing up	(Ref: Got by)		
	Lived comfortably	1.36	1.00
	Found it difficult	1.94	1.00
	Found it very difficult	1.04	1.00
Abuse	(Ref: No)		
	Yes	1.64	1.00
Outsider growing up	(Ref: No)		
	Yes	1.66	1.00
Self-rated health growing up	(Ref: Good)		
	Excellent	1.80	1.00
	Very good	1.39	1.00
	Fair	1.59	1.00
	Poor	2.54	1.49
Immigration status	(Ref: Born in this country)		
	Born in another country	1.17	1.00
Age 12 religious service attendance	(Ref: Never)		
	At least 1/week	1.10	1.00
	1-3/month	1.72	1.00
	< 1/month	1.87	1.00
Year of birth	(Ref: 1998-2005; current age: 18-24)		
	1988-1998; age 25-34	1.41	1.00
	1978-1988; age 35-44	2.02	1.21
	1968-1978; age 45-54	1.73	1.00
	1958-1968; age 55-64	1.36	1.00
	1948-1957; age 65-74	1.21	1.00
	1938-1948; age 75-84	2.16	1.08
Gender	(Ref: Male)		
	Female	1.17	1.00
	Other	3.63	1.75
Religious affiliation	(Ref: No religion/Atheist/Agnostic)		
	Islam	2.00	1.00
	Christianity	1.33	1.00
	Collapsed affiliations with prevalence<3%	1.48	1.00
Race/ethnicity	(Ref: Plurality group)		
	Non-plurality groups	2.80	1.62

203. Table S203. Nationally representative descriptive statistics for Spain conditional on smokers: Childhood predictors

Characteristic	N = 2,082[1]
**Relationship with mother**	
Very good	1,446 (69%)
Somewhat good	458 (22%)
Somewhat bad	92 (4.4%)
Very bad	30 (1.4%)
Does not apply	48 (2.3%)
(Missing)	9 (0.4%)
**Relationship with father**	
Very good	1,310 (63%)
Somewhat good	489 (23%)
Somewhat bad	107 (5.2%)
Very bad	63 (3.0%)
Does not apply	97 (4.7%)
(Missing)	16 (0.8%)
**Parent marital status**	
Parents married	1,729 (83%)
Divorced	155 (7.4%)
Parents were never married	99 (4.8%)
One or both parents had died	39 (1.9%)
(Missing)	60 (2.9%)
**Subjective financial status of family growing up**	
Lived comfortably	709 (34%)
Got by	945 (45%)
Found it difficult	381 (18%)
Found it very difficult	39 (1.9%)
(Missing)	9 (0.4%)
**Abuse**	
Yes	275 (13%)
No	1,765 (85%)
(Missing)	42 (2.0%)
**Outsider growing up**	
Yes	230 (11%)
No	1,818 (87%)
(Missing)	34 (1.6%)
**Self-rated health growing up**	
Excellent	787 (38%)
Very good	751 (36%)
Good	435 (21%)
Fair	51 (2.5%)
Poor	41 (2.0%)
(Missing)	15 (0.7%)
**Immigration status**	
Born in this country	1,867 (90%)
Born in another country	207 (9.9%)
(Missing)	8 (0.4%)
**Age 12 religious service attendance**	
At least 1/week	739 (35%)
1-3/month	381 (18%)
<1/month	433 (21%)
Never	514 (25%)
(Missing)	15 (0.7%)

204. Table S204. Nationally representative descriptive statistics for Spain conditional on smokers: Demographic variables

Characteristic	N = 2,082[1]
**Year of birth**	
1998-2005; age 18-24	165 (7.9%)
1988-1998; age 25-34	323 (16%)
1978-1988; age 35-44	491 (24%)
1968-1978; age 45-54	481 (23%)
1958-1968; age 55-64	358 (17%)
1948-1958; age 65-74	230 (11%)
1938-1948; age 75-84	32 (1.6%)
1938 or earlier; age 85+	2 (<0.1%)
(Missing)	0 (0%)
**Gender**	
Male	1,128 (54%)
Female	946 (45%)
Other	3 (0.2%)
(Missing)	4 (0.2%)
**Religious affiliation**	
Christianity	1,691 (81%)
Islam	49 (2.4%)
Hinduism	1 (<0.1%)
Buddhism	3 (0.2%)
Judaism	2 (0.1%)
Sikhism	2 (<0.1%)
Baha'i	0 (0%)
Jainism	0 (0%)
Shinto	0 (0%)
Taoism	0 (0%)
Confucianism	1 (<0.1%)
Primal, Animist, or Folk religion	4 (0.2%)
Spiritism	0 (0%)
Umbanda, Candomble, and other African-derived religions	0 (0%)
Chinese folk/traditional religion	0 (0%)
Some other religion	9 (0.4%)
No religion/Atheist/Agnostic	314 (15%)
(Missing)	7 (0.3%)

[1]n (%)

205. Table S205. Childhood predictors regression for Spain conditional on smokers

Variable	Category	Est	95% CI	SE	Global p-value
Relationship with mother	(Ref: Very bad/somewhat bad)				0.108
	Very good/somewhat good	-1.50	(-3.53, 0.53)	1.03	
Relationship with father	(Ref: Very bad/somewhat bad)				0.250
	Very good/somewhat good	-0.83	(-2.54, 0.88)	0.86	
Parent marital status	(Ref: Parents married)				0.023
	Divorced	-1.48	(-3.74, 0.78)	1.10	
	Parents were never married	-2.05	(-3.74, -0.35)	0.86	
	One or both parents had died	-0.12	(-2.71, 2.48)	1.32	
Subjective financial status of family growing up	(Ref: Got by)				0.526
	Lived comfortably	-0.13	(-1.13, 0.87)	0.51	
	Found it difficult	0.54	(-0.77, 1.85)	0.67	
	Found it very difficult	2.07	(-1.48, 5.62)	1.81	
Abuse	(Ref: No)				0.402
	Yes	0.59	(-0.90, 2.08)	0.76	
Outsider growing up	(Ref: No)				0.394
	Yes	0.62	(-1.04, 2.28)	0.84	
Self-rated health growing up	(Ref: Good)				0.921
	Excellent	0.31	(-0.88, 1.51)	0.61	
	Very good	0.09	(-1.24, 1.42)	0.67	
	Fair	-0.05	(-2.56, 2.45)	1.28	
	Poor	-1.03	(-4.90, 2.84)	1.97	
Immigration status	(Ref: Born in this country)				0.014
	Born in another country	-1.46	(-2.66, -0.26)	0.61	
Age 12 religious service attendance	(Ref: Never)				0.044
	At least 1/week	-1.02	(-2.22, 0.19)	0.61	
	1-3/month	-1.69	(-3.01, -0.36)	0.67	
	< 1/month	-0.73	(-2.13, 0.66)	0.70	
Year of birth	(Ref: 1998-2005; current age: 18-24)				0.000
	1988-1998; age 25-34	2.01	(0.41, 3.60)	0.80	
	1978-1988; age 35-44	4.11	(2.85, 5.37)	0.64	
	1968-1978; age 45-54	4.93	(3.66, 6.20)	0.65	
	1958-1968; age 55-64	6.04	(4.59, 7.49)	0.74	
	1948-1957; age 65-74	5.11	(2.87, 7.34)	1.14	
	1938-1948; age 75-84	2.08	(-0.32, 4.48)	1.22	
	1938 or earlier; 85 or older	1.55	(-3.66, 6.76)	2.66	
Gender	(Ref: Male)				4.07e-06
	Female	-2.16	(-3.03, -1.30)	0.44	
	Other	-0.73	(-6.75, 5.30)	3.07	
Religious affiliation	(Ref: No religion/Atheist/Agnostic)				0.141
	Christianity	-0.38	(-1.61, 0.85)	0.63	
	Collapsed affiliations with prevalence<3%	-1.16	(-5.92, 3.60)	2.25	
Race/ethnicity	(Ref: Plurality group)				

206. Table S206. Sensitivity to unmeasured confounding of childhood predictors in Spain conditional on smokers

Variable	Category	E-value for Estimate	E-value for 95% CI
Relationship with mother	(Ref: Very bad/somewhat bad)		
	Very good/somewhat good	1.67	1.00
Relationship with father	(Ref: Very bad/somewhat bad)		
	Very good/somewhat good	1.44	1.00
Parent marital status	(Ref: Parents married)		
	Divorced	1.66	1.00
	Parents were never married	1.85	1.25
	One or both parents had died	1.13	1.00
Subjective financial status of family growing up	(Ref: Got by)		
	Lived comfortably	1.14	1.00
	Found it difficult	1.33	1.00
	Found it very difficult	1.86	1.00
Abuse	(Ref: No)		
	Yes	1.35	1.00
Outsider growing up	(Ref: No)		
	Yes	1.36	1.00
Self-rated health growing up	(Ref: Good)		
	Excellent	1.23	1.00
	Very good	1.12	1.00
	Fair	1.09	1.00
	Poor	1.51	1.00
Immigration status	(Ref: Born in this country)		
	Born in another country	1.65	1.21
Age 12 religious service attendance	(Ref: Never)		
	At least 1/week	1.50	1.00
	1-3/month	1.73	1.26
	< 1/month	1.40	1.00
Year of birth	(Ref: 1998-2005; current age: 18-24)		
	1988-1998; age 25-34	1.84	1.29
	1978-1988; age 35-44	2.61	2.14
	1968-1978; age 45-54	2.95	2.44
	1958-1968; age 55-64	3.46	2.81
	1948-1957; age 65-74	3.03	2.15
	1938-1948; age 75-84	1.86	1.00
	1938 or earlier; 85 or older	1.68	1.00
Gender	(Ref: Male)		
	Female	1.89	1.60
	Other	1.40	1.00
Religious affiliation	(Ref: No religion/Atheist/Agnostic)		
	Christianity	1.26	1.00
	Collapsed affiliations with prevalence<3%	1.55	1.00
Race/ethnicity	(Ref: Plurality group)		

207. Table S207. Nationally representative descriptive statistics for Sweden conditional on smokers: Childhood predictors

Characteristic	N = 1,858[1]
**Relationship with mother**	
Very good	1,040 (56%)
Somewhat good	527 (28%)
Somewhat bad	190 (10%)
Very bad	72 (3.9%)
Does not apply	27 (1.5%)
(Missing)	1 (<0.1%)
**Relationship with father**	
Very good	835 (45%)
Somewhat good	570 (31%)
Somewhat bad	195 (10%)
Very bad	149 (8.0%)
Does not apply	108 (5.8%)
(Missing)	0 (0%)
**Parent marital status**	
Parents married	1,232 (66%)
Divorced	290 (16%)
Parents were never married	250 (13%)
One or both parents had died	59 (3.2%)
(Missing)	25 (1.4%)
**Subjective financial status of family growing up**	
Lived comfortably	626 (34%)
Got by	1,004 (54%)
Found it difficult	198 (11%)
Found it very difficult	27 (1.5%)
(Missing)	3 (0.2%)
**Abuse**	
Yes	406 (22%)
No	1,449 (78%)
(Missing)	3 (0.1%)
**Outsider growing up**	
Yes	322 (17%)
No	1,508 (81%)
(Missing)	28 (1.5%)
**Self-rated health growing up**	
Excellent	700 (38%)
Very good	582 (31%)
Good	342 (18%)
Fair	168 (9.1%)
Poor	61 (3.3%)
(Missing)	4 (0.2%)
**Immigration status**	
Born in this country	1,659 (89%)
Born in another country	178 (9.6%)
(Missing)	21 (1.1%)
**Age 12 religious service attendance**	
At least 1/week	131 (7.0%)
1-3/month	193 (10%)
<1/month	750 (40%)
Never	778 (42%)
(Missing)	7 (0.4%)

208. Table S208. Nationally representative descriptive statistics for Sweden conditional on smokers: Demographic variables

Characteristic	N = 1,858[1]
**Year of birth**	
1998-2005; age 18-24	161 (8.7%)
1988-1998; age 25-34	322 (17%)
1978-1988; age 35-44	274 (15%)
1968-1978; age 45-54	268 (14%)
1958-1968; age 55-64	320 (17%)
1948-1958; age 65-74	302 (16%)
1938-1948; age 75-84	203 (11%)
1938 or earlier; age 85+	8 (0.5%)
(Missing)	0 (0%)
**Gender**	
Male	858 (46%)
Female	992 (53%)
Other	4 (0.2%)
(Missing)	3 (0.1%)
**Religious affiliation**	
Christianity	1,261 (68%)
Islam	109 (5.9%)
Hinduism	7 (0.4%)
Buddhism	4 (0.2%)
Judaism	9 (0.5%)
Sikhism	2 (<0.1%)
Baha'i	0 (0%)
Jainism	0 (0%)
Shinto	0 (0%)
Taoism	0 (0%)
Confucianism	2 (<0.1%)
Primal, Animist, or Folk religion	6 (0.3%)
Spiritism	0 (0%)
Umbanda, Candomble, and other African-derived religions	0 (0%)
Chinese folk/traditional religion	0 (0%)
Some other religion	15 (0.8%)
No religion/Atheist/Agnostic	439 (24%)
(Missing)	3 (0.2%)

[1]n (%)

209. Table S209. Childhood predictors regression for Sweden conditional on smokers

Variable	Category	Est	95% CI	SE	Global p-value
Relationship with mother	(Ref: Very bad/somewhat bad)				0.528
	Very good/somewhat good	-0.41	(-1.70, 0.88)	0.66	
Relationship with father	(Ref: Very bad/somewhat bad)				0.377
	Very good/somewhat good	-0.50	(-1.63, 0.63)	0.58	
Parent marital status	(Ref: Parents married)				0.183
	Divorced	1.26	(0.06, 2.46)	0.61	
	Parents were never married	0.90	(-0.29, 2.09)	0.61	
	One or both parents had died	0.78	(-1.45, 3.02)	1.14	
Subjective financial status of family growing up	(Ref: Got by)				0.648
	Lived comfortably	0.39	(-0.48, 1.27)	0.44	
	Found it difficult	0.16	(-1.12, 1.43)	0.65	
	Found it very difficult	1.33	(-1.33, 3.98)	1.35	
Abuse	(Ref: No)				0.015
	Yes	-1.23	(-2.23, -0.23)	0.51	
Outsider growing up	(Ref: No)				0.063
	Yes	0.97	(-0.06, 2.00)	0.53	
Self-rated health growing up	(Ref: Good)				0.337
	Excellent	0.88	(-0.44, 2.19)	0.67	
	Very good	0.29	(-0.93, 1.50)	0.62	
	Fair	1.12	(-0.50, 2.73)	0.83	
	Poor	1.48	(-0.63, 3.59)	1.08	
Immigration status	(Ref: Born in this country)				0.912
	Born in another country	0.01	(-1.41, 1.43)	0.72	
Age 12 religious service attendance	(Ref: Never)				0.024
	At least 1/week	-1.75	(-3.16, -0.35)	0.72	
	1-3/month	-0.40	(-1.73, 0.93)	0.68	
	< 1/month	0.39	(-0.59, 1.36)	0.50	
Year of birth	(Ref: 1998-2005; current age: 18-24)				1.08e-09
	1988-1998; age 25-34	1.57	(0.02, 3.12)	0.79	
	1978-1988; age 35-44	3.31	(1.72, 4.89)	0.81	
	1968-1978; age 45-54	4.23	(2.63, 5.84)	0.82	
	1958-1968; age 55-64	4.51	(2.85, 6.17)	0.85	
	1948-1957; age 65-74	5.13	(3.17, 7.08)	1.00	
	1938-1948; age 75-84	2.38	(0.41, 4.34)	1.00	
	1938 or earlier; 85 or older	6.65	(-4.67, 17.96)	5.76	
Gender	(Ref: Male)				0.107
	Female	0.95	(0.06, 1.84)	0.45	
	Other	1.10	(-12.50, 14.71)	6.94	
Religious affiliation	(Ref: No religion/Atheist/Agnostic)				0.033
	Islam	2.54	(-0.00, 5.08)	1.30	
	Christianity	0.98	(0.04, 1.93)	0.48	
	Collapsed affiliations with prevalence<3%	-1.08	(-3.28, 1.12)	1.12	
Race/ethnicity	(Ref: Plurality group)				

210. Table S210. Sensitivity to unmeasured confounding of childhood predictors in Sweden conditional on smokers

Variable	Category	E-value for Estimate	E-value for 95% CI
Relationship with mother	(Ref: Very bad/somewhat bad)		
	Very good/somewhat good	1.30	1.00
Relationship with father	(Ref: Very bad/somewhat bad)		
	Very good/somewhat good	1.34	1.00
Parent marital status	(Ref: Parents married)		
	Divorced	1.65	1.10
	Parents were never married	1.51	1.00
	One or both parents had died	1.46	1.00
Subjective financial status of family growing up	(Ref: Got by)		
	Lived comfortably	1.29	1.00
	Found it difficult	1.17	1.00
	Found it very difficult	1.67	1.00
Abuse	(Ref: No)		
	Yes	1.64	1.21
Outsider growing up	(Ref: No)		
	Yes	1.53	1.00
Self-rated health growing up	(Ref: Good)		
	Excellent	1.50	1.00
	Very good	1.24	1.00
	Fair	1.59	1.00
	Poor	1.73	1.00
Immigration status	(Ref: Born in this country)		
	Born in another country	1.04	1.00
Age 12 religious service attendance	(Ref: Never)		
	At least 1/week	1.84	1.27
	1-3/month	1.29	1.00
	< 1/month	1.29	1.00
Year of birth	(Ref: 1998-2005; current age: 18-24)		
	1988-1998; age 25-34	1.77	1.06
	1978-1988; age 35-44	2.48	1.83
	1968-1978; age 45-54	2.91	2.19
	1958-1968; age 55-64	3.05	2.29
	1948-1957; age 65-74	3.37	2.42
	1938-1948; age 75-84	2.09	1.30
	1938 or earlier; 85 or older	4.29	1.00
Gender	(Ref: Male)		
	Female	1.53	1.10
	Other	1.59	1.00
Religious affiliation	(Ref: No religion/Atheist/Agnostic)		
	Islam	2.15	1.02
	Christianity	1.54	1.08
	Collapsed affiliations with prevalence<3%	1.58	1.00
Race/ethnicity	(Ref: Plurality group)		

211. Table S211. Nationally representative descriptive statistics for Tanzania conditional on smokers: Childhood predictors

Characteristic	N = 557[1]
**Relationship with mother**	
Very good	456 (82%)
Somewhat good	62 (11%)
Somewhat bad	10 (1.8%)
Very bad	2 (0.3%)
Does not apply	22 (3.9%)
(Missing)	6 (1.1%)
**Relationship with father**	
Very good	403 (72%)
Somewhat good	74 (13%)
Somewhat bad	19 (3.5%)
Very bad	19 (3.3%)
Does not apply	35 (6.3%)
(Missing)	6 (1.1%)
**Parent marital status**	
Parents married	425 (76%)
Divorced	32 (5.8%)
Parents were never married	50 (9.0%)
One or both parents had died	20 (3.6%)
(Missing)	30 (5.3%)
**Subjective financial status of family growing up**	
Lived comfortably	148 (27%)
Got by	197 (35%)
Found it difficult	156 (28%)
Found it very difficult	49 (8.7%)
(Missing)	8 (1.5%)
**Abuse**	
Yes	59 (11%)
No	497 (89%)
(Missing)	1 (0.2%)
**Outsider growing up**	
Yes	52 (9.3%)
No	505 (91%)
(Missing)	0 (0%)
**Self-rated health growing up**	
Excellent	149 (27%)
Very good	120 (22%)
Good	179 (32%)
Fair	84 (15%)
Poor	19 (3.4%)
(Missing)	5 (1.0%)
**Immigration status**	
Born in this country	555 (100%)
Born in another country	1 (<0.1%)
(Missing)	1 (0.2%)
**Age 12 religious service attendance**	
At least 1/week	338 (61%)
1-3/month	154 (28%)
<1/month	24 (4.4%)
Never	30 (5.3%)
(Missing)	12 (2.1%)

212. Table S212. Nationally representative descriptive statistics for Tanzania conditional on smokers: Demographic variables

Characteristic	N = 557[1]
**Year of birth**	
1998-2005; age 18-24	96 (17%)
1988-1998; age 25-34	152 (27%)
1978-1988; age 35-44	123 (22%)
1968-1978; age 45-54	91 (16%)
1958-1968; age 55-64	55 (9.9%)
1948-1958; age 65-74	36 (6.5%)
1938-1948; age 75-84	4 (0.7%)
1938 or earlier; age 85+	0 (0%)
(Missing)	0 (0%)
**Gender**	
Male	432 (78%)
Female	125 (22%)
Other	0 (0%)
(Missing)	0 (0%)
**Religious affiliation**	
Christianity	289 (52%)
Islam	249 (45%)
Hinduism	0 (0%)
Buddhism	0 (0%)
Judaism	0 (0%)
Sikhism	0 (0%)
Baha'i	0 (0%)
Jainism	0 (0%)
Shinto	0 (0%)
Taoism	0 (0%)
Confucianism	0 (0%)
Primal, Animist, or Folk religion	0 (0%)
Spiritism	0 (0%)
Umbanda, Candomble, and other African-derived religions	0 (0%)
Chinese folk/traditional religion	0 (0%)
Some other religion	0 (0%)
No religion/Atheist/Agnostic	18 (3.2%)
(Missing)	0 (<0.1%)
**Race/Ethnicity**	
African	557 (100%)
Arab	0 (0%)
Indian	0 (0%)
(Missing)	0 (0%)

[1]n (%)

213. Table S213. Childhood predictors regression for Tanzania conditional on smokers

Variable	Category	Est	95% CI	SE	Global p-value
Relationship with mother	(Ref: Very bad/somewhat bad)				0.253
	Very good/somewhat good	1.51	(-1.10, 4.12)	1.33	
Relationship with father	(Ref: Very bad/somewhat bad)				0.696
	Very good/somewhat good	0.63	(-2.52, 3.77)	1.60	
Parent marital status	(Ref: Parents married)				0.438
	Divorced	0.29	(-2.59, 3.18)	1.47	
	Parents were never married	0.29	(-1.50, 2.07)	0.91	
	One or both parents had died	2.11	(-0.44, 4.66)	1.30	
Subjective financial status of family growing up	(Ref: Got by)				0.987
	Lived comfortably	-0.13	(-1.87, 1.61)	0.89	
	Found it difficult	0.12	(-2.46, 2.71)	1.32	
	Found it very difficult	0.25	(-1.93, 2.43)	1.11	
Abuse	(Ref: No)				0.206
	Yes	-1.51	(-3.85, 0.82)	1.19	
Outsider growing up	(Ref: No)				0.302
	Yes	1.52	(-1.36, 4.41)	1.47	
Self-rated health growing up	(Ref: Good)				0.085
	Excellent	-0.58	(-2.38, 1.22)	0.92	
	Very good	-2.06	(-4.48, 0.37)	1.24	
	Fair	-0.33	(-2.28, 1.62)	0.99	
	Poor	-3.01	(-5.41, -0.60)	1.23	
Immigration status	(Ref: Born in this country)				0.012
	Born in another country	-2.15	(-3.81, -0.48)	0.85	
Age 12 religious service attendance	(Ref: Never)				0.265
	At least 1/week	0.89	(-1.76, 3.53)	1.32	
	1-3/month	1.32	(-0.91, 3.55)	1.10	
	< 1/month	0.93	(-1.63, 3.49)	1.28	
Year of birth	(Ref: 1998-2005; current age: 18-24)				0.274
	1988-1998; age 25-34	1.41	(-1.07, 3.89)	1.27	
	1978-1988; age 35-44	1.98	(-0.55, 4.50)	1.29	
	1968-1978; age 45-54	0.98	(-1.18, 3.14)	1.10	
	1958-1968; age 55-64	3.04	(-2.49, 8.57)	2.82	
	1948-1957; age 65-74	-0.13	(-2.12, 1.85)	1.01	
	1938-1948; age 75-84	7.71	(0.09, 15.34)	3.89	
Gender	(Ref: Male)				0.270
	Female	6.78	(-5.22, 18.78)	6.12	
Religious affiliation	(Ref: No religion/Atheist/Agnostic)				0.113
	Islam	2.86	(-0.52, 6.24)	1.72	
	Christianity	1.16	(-1.46, 3.78)	1.33	
Race/ethnicity	(Ref: Plurality group)				

214. Table S214. Sensitivity to unmeasured confounding of childhood predictors in Tanzania conditional on smokers

Variable	Category	E-value for Estimate	E-value for 95% CI
Relationship with mother	(Ref: Very bad/somewhat bad)		
	Very good/somewhat good	1.73	1.00
Relationship with father	(Ref: Very bad/somewhat bad)		
	Very good/somewhat good	1.39	1.00
Parent marital status	(Ref: Parents married)		
	Divorced	1.24	1.00
	Parents were never married	1.24	1.00
	One or both parents had died	1.96	1.00
Subjective financial status of family growing up	(Ref: Got by)		
	Lived comfortably	1.15	1.00
	Found it difficult	1.14	1.00
	Found it very difficult	1.22	1.00
Abuse	(Ref: No)		
	Yes	1.73	1.00
Outsider growing up	(Ref: No)		
	Yes	1.74	1.00
Self-rated health growing up	(Ref: Good)		
	Excellent	1.37	1.00
	Very good	1.94	1.00
	Fair	1.26	1.00
	Poor	2.32	1.38
Immigration status	(Ref: Born in this country)		
	Born in another country	1.98	1.33
Age 12 religious service attendance	(Ref: Never)		
	At least 1/week	1.49	1.00
	1-3/month	1.66	1.00
	< 1/month	1.51	1.00
Year of birth	(Ref: 1998-2005; current age: 18-24)		
	1988-1998; age 25-34	1.69	1.00
	1978-1988; age 35-44	1.91	1.00
	1968-1978; age 45-54	1.53	1.00
	1958-1968; age 55-64	2.34	1.00
	1948-1957; age 65-74	1.15	1.00
	1938-1948; age 75-84	4.91	1.13
Gender	(Ref: Male)		
	Female	4.27	1.00
Religious affiliation	(Ref: No religion/Atheist/Agnostic)		
	Islam	2.26	1.00
	Christianity	1.60	1.00
Race/ethnicity	(Ref: Plurality group)		

215. Table S215. Nationally representative descriptive statistics for Türkiye conditional on smokers: Childhood predictors

Characteristic	N = 789[1]
**Relationship with mother**	
Very good	515 (65%)
Somewhat good	217 (28%)
Somewhat bad	26 (3.4%)
Very bad	18 (2.2%)
Does not apply	11 (1.4%)
(Missing)	2 (0.2%)
**Relationship with father**	
Very good	416 (53%)
Somewhat good	213 (27%)
Somewhat bad	50 (6.4%)
Very bad	57 (7.2%)
Does not apply	36 (4.6%)
(Missing)	17 (2.1%)
**Parent marital status**	
Parents married	694 (88%)
Divorced	42 (5.4%)
Parents were never married	4 (0.5%)
One or both parents had died	35 (4.4%)
(Missing)	14 (1.8%)
**Subjective financial status of family growing up**	
Lived comfortably	265 (34%)
Got by	346 (44%)
Found it difficult	114 (14%)
Found it very difficult	62 (7.9%)
(Missing)	2 (0.2%)
**Abuse**	
Yes	92 (12%)
No	681 (86%)
(Missing)	16 (2.0%)
**Outsider growing up**	
Yes	96 (12%)
No	688 (87%)
(Missing)	5 (0.7%)
**Self-rated health growing up**	
Excellent	211 (27%)
Very good	210 (27%)
Good	212 (27%)
Fair	122 (15%)
Poor	35 (4.4%)
(Missing)	0 (0%)
**Immigration status**	
Born in this country	761 (96%)
Born in another country	28 (3.6%)
(Missing)	0 (0%)
**Age 12 religious service attendance**	
At least 1/week	366 (46%)
1-3/month	136 (17%)
<1/month	105 (13%)
Never	175 (22%)
(Missing)	7 (0.9%)

216. **Table S216. Nationally representative descriptive statistics for Türkiye conditional on smokers: Demographic variables**

**Characteristic**	**N = 789**[1]
**Year of birth**	
1998-2005; age 18-24	116 (15%)
1988-1998; age 25-34	181 (23%)
1978-1988; age 35-44	176 (22%)
1968-1978; age 45-54	151 (19%)
1958-1968; age 55-64	106 (13%)
1948-1958; age 65-74	48 (6.0%)
1938-1948; age 75-84	8 (1.1%)
1938 or earlier; age 85+	3 (0.4%)
(Missing)	0 (0%)
**Gender**	
Male	478 (61%)
Female	311 (39%)
Other	0 (0%)
(Missing)	0 (0%)
**Religious affiliation**	
Christianity	1 (0.1%)
Islam	769 (97%)
Hinduism	0 (0%)
Buddhism	0 (0%)
Judaism	1 (0.1%)
Sikhism	0 (0%)
Baha'i	0 (0%)
Jainism	0 (0%)
Shinto	0 (0%)
Taoism	0 (0%)
Confucianism	0 (0%)
Primal, Animist, or Folk religion	0 (0%)
Spiritism	0 (0%)
Umbanda, Candomble, and other African-derived religions	0 (0%)
Chinese folk/traditional religion	0 (0%)
Some other religion	0 (0%)
No religion/Atheist/Agnostic	9 (1.1%)
(Missing)	10 (1.2%)
**Race/Ethnicity**	
Albanian	7 (0.8%)
Arab	19 (2.4%)
Armenian	0 (0%)
Azeri	8 (1.0%)
Bosnian	3 (0.4%)
Circassian	16 (2.0%)
Georgian	4 (0.5%)
Greek	1 (0.1%)
Kurdish/Zaza	146 (18%)
Laz	11 (1.4%)
Other	30 (3.7%)
Turkish	540 (68%)
Uyghur	1 (0.2%)
(Missing)	3 (0.4%)

[1]n (%)

217. Table S217. Childhood predictors regression for Türkiye conditional on smokers

Variable	Category	Est	95% CI	SE	Global p-value
Relationship with mother	(Ref: Very bad/somewhat bad)				0.158
	Very good/somewhat good	2.87	(-1.15, 6.89)	2.05	
Relationship with father	(Ref: Very bad/somewhat bad)				0.023
	Very good/somewhat good	-3.47	(-6.62, -0.32)	1.60	
Parent marital status	(Ref: Parents married)				0.406
	Divorced	-0.19	(-4.33, 3.95)	2.11	
	Parents were never married	-6.80	(-16.20, 2.60)	4.79	
	One or both parents had died	-2.75	(-8.59, 3.08)	2.98	
Subjective financial status of family growing up	(Ref: Got by)				0.924
	Lived comfortably	0.30	(-1.91, 2.51)	1.13	
	Found it difficult	-0.56	(-4.16, 3.04)	1.84	
	Found it very difficult	-1.01	(-5.23, 3.21)	2.15	
Abuse	(Ref: No)				0.372
	Yes	1.41	(-1.80, 4.61)	1.63	
Outsider growing up	(Ref: No)				0.612
	Yes	0.85	(-2.46, 4.16)	1.69	
Self-rated health growing up	(Ref: Good)				0.047
	Excellent	-2.74	(-5.47, -0.01)	1.39	
	Very good	-3.73	(-6.55, -0.91)	1.44	
	Fair	-2.73	(-6.25, 0.79)	1.80	
	Poor	2.08	(-4.53, 8.69)	3.37	
Immigration status	(Ref: Born in this country)				0.660
	Born in another country	1.68	(-5.82, 9.17)	3.82	
Age 12 religious service attendance	(Ref: Never)				0.539
	At least 1/week	-0.54	(-3.13, 2.05)	1.32	
	1-3/month	-2.04	(-5.13, 1.06)	1.58	
	< 1/month	-1.43	(-4.27, 1.40)	1.45	
Year of birth	(Ref: 1998-2005; current age: 18-24)				0.420
	1988-1998; age 25-34	1.87	(-1.18, 4.92)	1.56	
	1978-1988; age 35-44	2.15	(-0.91, 5.22)	1.56	
	1968-1978; age 45-54	2.40	(-1.11, 5.92)	1.79	
	1958-1968; age 55-64	1.18	(-2.70, 5.05)	1.97	
	1948-1957; age 65-74	0.75	(-5.96, 7.46)	3.42	
	1938-1948; age 75-84	-3.87	(-10.17, 2.43)	3.21	
	1938 or earlier; 85 or older	2.56	(-1.62, 6.74)	2.13	
Gender	(Ref: Male)				1.67e-15
	Female	-8.43	(-10.45, -6.40)	1.03	
Religious affiliation	(Ref: Islam)				0.046
	Collapsed affiliations with prevalence<3%	-3.69	(-7.35, -0.03)	1.87	
Race/ethnicity	(Ref: Plurality group)				0.485
	Non-plurality groups	0.80	(-1.63, 3.23)	1.24	

218. Table S218. Sensitivity to unmeasured confounding of childhood predictors in Türkiye conditional on smokers

Variable	Category	E-value for Estimate	E-value for 95% CI
Relationship with mother	(Ref: Very bad/somewhat bad)		
	Very good/somewhat good	1.74	1.00
Relationship with father	(Ref: Very bad/somewhat bad)		
	Very good/somewhat good	1.86	1.18
Parent marital status	(Ref: Parents married)		
	Divorced	1.13	1.00
	Parents were never married	2.59	1.00
	One or both parents had died	1.72	1.00
Subjective financial status of family growing up	(Ref: Got by)		
	Lived comfortably	1.17	1.00
	Found it difficult	1.24	1.00
	Found it very difficult	1.35	1.00
Abuse	(Ref: No)		
	Yes	1.44	1.00
Outsider growing up	(Ref: No)		
	Yes	1.31	1.00
Self-rated health growing up	(Ref: Good)		
	Excellent	1.71	1.03
	Very good	1.91	1.33
	Fair	1.71	1.00
	Poor	1.58	1.00
Immigration status	(Ref: Born in this country)		
	Born in another country	1.50	1.00
Age 12 religious service attendance	(Ref: Never)		
	At least 1/week	1.24	1.00
	1-3/month	1.57	1.00
	< 1/month	1.44	1.00
Year of birth	(Ref: 1998-2005; current age: 18-24)		
	1988-1998; age 25-34	1.54	1.00
	1978-1988; age 35-44	1.59	1.00
	1968-1978; age 45-54	1.64	1.00
	1958-1968; age 55-64	1.39	1.00
	1948-1957; age 65-74	1.29	1.00
	1938-1948; age 75-84	1.94	1.00
	1938 or earlier; 85 or older	1.68	1.00
Gender	(Ref: Male)		
	Female	2.99	2.49
Religious affiliation	(Ref: Islam)		
	Collapsed affiliations with prevalence<3%	1.90	1.05
Race/ethnicity	(Ref: Plurality group)		
	Non-plurality groups	1.30	1.00

219. Table S219. Nationally representative descriptive statistics for United Kingdom conditional on smokers: Childhood predictors

Characteristic	N = 928[1]
**Relationship with mother**	
Very good	571 (62%)
Somewhat good	212 (23%)
Somewhat bad	66 (7.1%)
Very bad	49 (5.3%)
Does not apply	24 (2.6%)
(Missing)	6 (0.6%)
**Relationship with father**	
Very good	480 (52%)
Somewhat good	225 (24%)
Somewhat bad	82 (8.9%)
Very bad	61 (6.6%)
Does not apply	70 (7.5%)
(Missing)	9 (0.9%)
**Parent marital status**	
Parents married	690 (74%)
Divorced	107 (12%)
Parents were never married	76 (8.2%)
One or both parents had died	36 (3.9%)
(Missing)	18 (2.0%)
**Subjective financial status of family growing up**	
Lived comfortably	433 (47%)
Got by	332 (36%)
Found it difficult	115 (12%)
Found it very difficult	41 (4.4%)
(Missing)	6 (0.7%)
**Abuse**	
Yes	224 (24%)
No	694 (75%)
(Missing)	9 (1.0%)
**Outsider growing up**	
Yes	274 (30%)
No	636 (69%)
(Missing)	17 (1.9%)
**Self-rated health growing up**	
Excellent	336 (36%)
Very good	323 (35%)
Good	181 (19%)
Fair	61 (6.6%)
Poor	19 (2.1%)
(Missing)	8 (0.8%)
**Immigration status**	
Born in this country	815 (88%)
Born in another country	109 (12%)
(Missing)	4 (0.5%)
**Age 12 religious service attendance**	
At least 1/week	294 (32%)
1-3/month	144 (16%)
<1/month	161 (17%)
Never	321 (35%)
(Missing)	6 (0.7%)

220. **Table S220. Nationally representative descriptive statistics for United Kingdom conditional on smokers: Demographic variables**

Characteristic	N = 928[1]
**Year of birth**	
1998-2005; age 18-24	38 (4.0%)
1988-1998; age 25-34	220 (24%)
1978-1988; age 35-44	190 (20%)
1968-1978; age 45-54	157 (17%)
1958-1968; age 55-64	148 (16%)
1948-1958; age 65-74	133 (14%)
1938-1948; age 75-84	39 (4.2%)
1938 or earlier; age 85+	1 (0.1%)
(Missing)	0 (0%)
**Gender**	
Male	525 (57%)
Female	398 (43%)
Other	1 (0.1%)
(Missing)	3 (0.4%)
**Religious affiliation**	
Christianity	609 (66%)
Islam	43 (4.6%)
Hinduism	13 (1.4%)
Buddhism	3 (0.3%)
Judaism	7 (0.7%)
Sikhism	5 (0.5%)
Baha'i	4 (0.5%)
Jainism	0 (0%)
Shinto	0 (0%)
Taoism	0 (0%)
Confucianism	0 (0%)
Primal, Animist, or Folk religion	11 (1.2%)
Spiritism	0 (0%)
Umbanda, Candomble, and other African-derived religions	0 (0%)
Chinese folk/traditional religion	0 (0%)
Some other religion	3 (0.3%)
No religion/Atheist/Agnostic	226 (24%)
(Missing)	4 (0.4%)
**Race/Ethnicity**	
Asian	61 (6.6%)
Black	25 (2.7%)
Other	22 (2.3%)
White	814 (88%)
(Missing)	6 (0.7%)

[1]n (%)

221. Table S221. Childhood predictors regression for United Kingdom conditional on smokers

Variable	Category	Est	95% CI	SE	Global p-value
Relationship with mother	(Ref: Very bad/somewhat bad)				0.665
	Very good/somewhat good	0.41	(-1.48, 2.29)	0.96	
Relationship with father	(Ref: Very bad/somewhat bad)				0.127
	Very good/somewhat good	-1.47	(-3.38, 0.45)	0.98	
Parent marital status	(Ref: Parents married)				0.686
	Divorced	0.37	(-1.53, 2.26)	0.96	
	Parents were never married	1.27	(-0.97, 3.51)	1.14	
	One or both parents had died	-0.53	(-4.97, 3.91)	2.27	
Subjective financial status of family growing up	(Ref: Got by)				0.380
	Lived comfortably	1.13	(-0.28, 2.53)	0.72	
	Found it difficult	1.13	(-1.26, 3.53)	1.22	
	Found it very difficult	1.30	(-1.80, 4.40)	1.58	
Abuse	(Ref: No)				0.025
	Yes	1.79	(0.19, 3.40)	0.82	
Outsider growing up	(Ref: No)				0.003
	Yes	-2.13	(-3.64, -0.62)	0.77	
Self-rated health growing up	(Ref: Good)				0.524
	Excellent	-1.64	(-3.51, 0.23)	0.95	
	Very good	-1.19	(-3.00, 0.62)	0.92	
	Fair	-1.06	(-3.69, 1.57)	1.33	
	Poor	-1.33	(-4.84, 2.18)	1.79	
Immigration status	(Ref: Born in this country)				0.032
	Born in another country	-1.95	(-3.75, -0.15)	0.92	
Age 12 religious service attendance	(Ref: Never)				0.008
	At least 1/week	-2.60	(-4.41, -0.79)	0.93	
	1-3/month	-0.85	(-2.92, 1.23)	1.06	
	< 1/month	-2.51	(-4.52, -0.51)	1.02	
Year of birth	(Ref: 1998-2005; current age: 18-24)				2.32e-11
	1988-1998; age 25-34	0.06	(-2.73, 2.86)	1.42	
	1978-1988; age 35-44	4.42	(1.33, 7.51)	1.58	
	1968-1978; age 45-54	4.70	(1.53, 7.88)	1.62	
	1958-1968; age 55-64	6.25	(3.11, 9.39)	1.60	
	1948-1957; age 65-74	6.45	(3.08, 9.82)	1.72	
	1938-1948; age 75-84	4.26	(-0.51, 9.03)	2.43	
	1938 or earlier; 85 or older	2.23	(-5.71, 10.18)	4.05	
Gender	(Ref: Male)				0.619
	Female	0.12	(-1.19, 1.44)	0.67	
	Other	2.55	(-2.67, 7.77)	2.66	
Religious affiliation	(Ref: No religion/Atheist/Agnostic)				0.573
	Islam	-1.54	(-5.05, 1.96)	1.79	
	Christianity	-1.09	(-2.75, 0.58)	0.85	
	Collapsed affiliations with prevalence<3%	-1.44	(-5.17, 2.28)	1.90	
Race/ethnicity	(Ref: Plurality group)				0.003
	Non-plurality groups	-3.41	(-5.66, -1.16)	1.15	

222. Table S222. Sensitivity to unmeasured confounding of childhood predictors in United Kingdom conditional on smokers

Variable	Category	E-value for Estimate	E-value for 95% CI
Relationship with mother	(Ref: Very bad/somewhat bad)		
	Very good/somewhat good	1.27	1.00
Relationship with father	(Ref: Very bad/somewhat bad)		
	Very good/somewhat good	1.65	1.00
Parent marital status	(Ref: Parents married)		
	Divorced	1.26	1.00
	Parents were never married	1.59	1.00
	One or both parents had died	1.32	1.00
Subjective financial status of family growing up	(Ref: Got by)		
	Lived comfortably	1.54	1.00
	Found it difficult	1.54	1.00
	Found it very difficult	1.60	1.00
Abuse	(Ref: No)		
	Yes	1.76	1.17
Outsider growing up	(Ref: No)		
	Yes	1.88	1.36
Self-rated health growing up	(Ref: Good)		
	Excellent	1.71	1.00
	Very good	1.56	1.00
	Fair	1.52	1.00
	Poor	1.61	1.00
Immigration status	(Ref: Born in this country)		
	Born in another country	1.82	1.15
Age 12 religious service attendance	(Ref: Never)		
	At least 1/week	2.04	1.42
	1-3/month	1.44	1.00
	< 1/month	2.01	1.32
Year of birth	(Ref: 1998-2005; current age: 18-24)		
	1988-1998; age 25-34	1.09	1.00
	1978-1988; age 35-44	2.73	1.61
	1968-1978; age 45-54	2.84	1.68
	1958-1968; age 55-64	3.54	2.22
	1948-1957; age 65-74	3.64	2.21
	1938-1948; age 75-84	2.66	1.00
	1938 or earlier; 85 or older	1.91	1.00
Gender	(Ref: Male)		
	Female	1.14	1.00
	Other	2.02	1.00
Religious affiliation	(Ref: No religion/Atheist/Agnostic)		
	Islam	1.68	1.00
	Christianity	1.52	1.00
	Collapsed affiliations with prevalence<3%	1.65	1.00
Race/ethnicity	(Ref: Plurality group)		
	Non-plurality groups	2.33	1.55

223. Table S223. Nationally representative descriptive statistics for United States conditional on smokers: Childhood predictors

Characteristic	N = 4,176[1]
**Relationship with mother**	
Very good	1,988 (48%)
Somewhat good	1,242 (30%)
Somewhat bad	511 (12%)
Very bad	322 (7.7%)
Does not apply	95 (2.3%)
(Missing)	18 (0.4%)
**Relationship with father**	
Very good	1,494 (36%)
Somewhat good	1,310 (31%)
Somewhat bad	634 (15%)
Very bad	365 (8.7%)
Does not apply	369 (8.8%)
(Missing)	4 (0.1%)
**Parent marital status**	
Parents married	2,517 (60%)
Divorced	827 (20%)
Parents were never married	593 (14%)
One or both parents had died	113 (2.7%)
(Missing)	127 (3.0%)
**Subjective financial status of family growing up**	
Lived comfortably	1,388 (33%)
Got by	1,812 (43%)
Found it difficult	618 (15%)
Found it very difficult	357 (8.6%)
(Missing)	1 (<0.1%)
**Abuse**	
Yes	1,626 (39%)
No	2,530 (61%)
(Missing)	21 (0.5%)
**Outsider growing up**	
Yes	1,642 (39%)
No	2,467 (59%)
(Missing)	68 (1.6%)
**Self-rated health growing up**	
Excellent	1,634 (39%)
Very good	1,249 (30%)
Good	909 (22%)
Fair	265 (6.3%)
Poor	113 (2.7%)
(Missing)	5 (0.1%)
**Immigration status**	
Born in this country	3,975 (95%)
Born in another country	107 (2.6%)
(Missing)	94 (2.3%)
**Age 12 religious service attendance**	
At least 1/week	1,713 (41%)
1-3/month	773 (19%)
<1/month	726 (17%)
Never	917 (22%)
(Missing)	47 (1.1%)

224. Table S224. Nationally representative descriptive statistics for United States conditional on smokers: Demographic variables

Characteristic	N = 4,176[1]
**Year of birth**	
1998-2005; age 18-24	175 (4.2%)
1988-1998; age 25-34	895 (21%)
1978-1988; age 35-44	771 (18%)
1968-1978; age 45-54	667 (16%)
1958-1968; age 55-64	989 (24%)
1948-1958; age 65-74	525 (13%)
1938-1948; age 75-84	124 (3.0%)
1938 or earlier; age 85+	30 (0.7%)
(Missing)	0 (0%)
**Gender**	
Male	1,977 (47%)
Female	2,153 (52%)
Other	33 (0.8%)
(Missing)	13 (0.3%)
**Religious affiliation**	
Christianity	3,294 (79%)
Islam	4 (0.1%)
Hinduism	20 (0.5%)
Buddhism	4 (<0.1%)
Judaism	33 (0.8%)
Sikhism	18 (0.4%)
Baha'i	1 (<0.1%)
Jainism	2 (<0.1%)
Shinto	0 (<0.1%)
Taoism	0 (<0.1%)
Confucianism	0 (<0.1%)
Primal, Animist, or Folk religion	16 (0.4%)
Spiritism	0 (0%)
Umbanda, Candomble, and other African-derived religions	0 (0%)
Chinese folk/traditional religion	0 (0%)
Some other religion	63 (1.5%)
No religion/Atheist/Agnostic	711 (17%)
(Missing)	8 (0.2%)
**Race/Ethnicity**	
Asian	111 (2.7%)
Black	655 (16%)
Hispanic	757 (18%)
Other	150 (3.6%)
White	2,502 (60%)
(Missing)	2 (<0.1%)

[1]n (%)

225. Table S225. Childhood predictors regression for United States conditional on smokers

Variable	Category	Est	95% CI	SE	Global p-value
Relationship with mother	(Ref: Very bad/somewhat bad)				0.095
	Very good/somewhat good	-1.49	(-3.26, 0.28)	0.90	
Relationship with father	(Ref: Very bad/somewhat bad)				0.708
	Very good/somewhat good	0.22	(-1.05, 1.49)	0.65	
Parent marital status	(Ref: Parents married)				0.286
	Divorced	-0.75	(-2.09, 0.58)	0.68	
	Parents were never married	0.83	(-1.53, 3.18)	1.20	
	One or both parents had died	-1.39	(-3.85, 1.07)	1.24	
Subjective financial status of family growing up	(Ref: Got by)				0.005
	Lived comfortably	-1.77	(-2.95, -0.59)	0.60	
	Found it difficult	-0.69	(-2.02, 0.64)	0.68	
	Found it very difficult	2.18	(-1.00, 5.35)	1.62	
Abuse	(Ref: No)				0.009
	Yes	1.45	(0.35, 2.54)	0.56	
Outsider growing up	(Ref: No)				0.314
	Yes	-0.62	(-1.84, 0.60)	0.62	
Self-rated health growing up	(Ref: Good)				0.636
	Excellent	1.13	(-0.36, 2.63)	0.76	
	Very good	0.69	(-0.76, 2.14)	0.74	
	Fair	0.47	(-2.07, 3.01)	1.29	
	Poor	1.17	(-2.18, 4.52)	1.71	
Immigration status	(Ref: Born in this country)				1.44e-04
	Born in another country	-3.61	(-5.56, -1.66)	0.99	
Age 12 religious service attendance	(Ref: Never)				0.003
	At least 1/week	-2.79	(-4.41, -1.16)	0.83	
	1-3/month	-3.09	(-4.90, -1.28)	0.92	
	< 1/month	-2.55	(-4.64, -0.46)	1.07	
Year of birth	(Ref: 1998-2005; current age: 18-24)				4.12e-04
	1988-1998; age 25-34	4.27	(0.10, 8.43)	2.12	
	1978-1988; age 35-44	4.65	(0.86, 8.45)	1.94	
	1968-1978; age 45-54	6.33	(2.33, 10.32)	2.04	
	1958-1968; age 55-64	6.64	(2.68, 10.59)	2.02	
	1948-1957; age 65-74	8.04	(3.95, 12.12)	2.09	
	1938-1948; age 75-84	5.66	(1.46, 9.86)	2.14	
	1938 or earlier; 85 or older	8.75	(-2.96, 20.45)	5.97	
Gender	(Ref: Male)				5.03e-04
	Female	-1.69	(-2.73, -0.64)	0.53	
	Other	-5.91	(-10.81, -1.01)	2.50	
Religious affiliation	(Ref: No religion/Atheist/Agnostic)				0.030
	Christianity	-1.74	(-3.62, 0.14)	0.96	
	Collapsed affiliations with prevalence<3%	-3.18	(-5.56, -0.81)	1.21	
Race/ethnicity	(Ref: Plurality group)				3.24e-12
	Non-plurality groups	-4.07	(-5.26, -2.88)	0.61	

226. Table S226. Sensitivity to unmeasured confounding of childhood predictors in United States conditional on smokers

Variable	Category	E-value for Estimate	E-value for 95% CI
Relationship with mother	(Ref: Very bad/somewhat bad)		
	Very good/somewhat good	1.62	1.00
Relationship with father	(Ref: Very bad/somewhat bad)		
	Very good/somewhat good	1.18	1.00
Parent marital status	(Ref: Parents married)		
	Divorced	1.38	1.00
	Parents were never married	1.41	1.00
	One or both parents had died	1.58	1.00
Subjective financial status of family growing up	(Ref: Got by)		
	Lived comfortably	1.70	1.33
	Found it difficult	1.36	1.00
	Found it very difficult	1.83	1.00
Abuse	(Ref: No)		
	Yes	1.60	1.24
Outsider growing up	(Ref: No)		
	Yes	1.33	1.00
Self-rated health growing up	(Ref: Good)		
	Excellent	1.51	1.00
	Very good	1.36	1.00
	Fair	1.28	1.00
	Poor	1.51	1.00
Immigration status	(Ref: Born in this country)		
	Born in another country	2.29	1.67
Age 12 religious service attendance	(Ref: Never)		
	At least 1/week	2.02	1.52
	1-3/month	2.11	1.55
	< 1/month	1.94	1.28
Year of birth	(Ref: 1998-2005; current age: 18-24)		
	1988-1998; age 25-34	2.51	1.12
	1978-1988; age 35-44	2.65	1.42
	1968-1978; age 45-54	3.31	1.88
	1958-1968; age 55-64	3.44	1.99
	1948-1957; age 65-74	4.09	2.40
	1938-1948; age 75-84	3.03	1.61
	1938 or earlier; 85 or older	4.46	1.00
Gender	(Ref: Male)		
	Female	1.68	1.34
	Other	3.13	1.47
Religious affiliation	(Ref: No religion/Atheist/Agnostic)		
	Christianity	1.69	1.00
	Collapsed affiliations with prevalence<3%	2.15	1.40
Race/ethnicity	(Ref: Plurality group)		
	Non-plurality groups	2.44	2.05

227. Table S227. Population weighted meta-analysis of regression results (continuous).

Variable	Category	Est	95% CI	SE
Relationship with mother	(Ref: Very bad/somewhat bad)			
	Very good/somewhat good	1.19	(-3.27,5.65)	2.274
Relationship with father	(Ref: Very bad/somewhat bad)			
	Very good/somewhat good	3.16	(-0.28,6.59)	1.752
Parent marital status	(Ref: Parents married)			
	Divorced	-1.50	(-4.13,1.14)	1.344
	Single, never married	0.48	(-1.59,2.55)	1.054
	One or both parents had died	1.12	(-1.15,3.38)	1.155
Subjective financial status of family growing up	(Ref: Got by)			
	Lived comfortably	0.15	(-0.83,1.12)	0.497
	Found it difficult	0.07	(-1.22,1.35)	0.655
	Found it very difficult	1.57	(-0.22,3.36)	0.912
Abuse	(Ref: No)			
	Yes	1.06	(-0.13,2.24)	0.605
Outsider growing up	(Ref: No)			
	Yes	0.31	(-0.87,1.49)	0.602
Self-rated health growing up	(Ref: Good)			
	Excellent	0.13	(-1.10,1.35)	0.627
	Very good	-0.18	(-1.66,1.30)	0.754
	Fair	-0.59	(-1.85,0.67)	0.642
	Poor	4.20	(0.09,8.30)	2.095
Immigration status	(Ref: Born in this country)			
	Born in another country	0.90	(-1.73,3.53)	1.341
Age 12 religious service attendance	(Ref: Never)			
	At least 1/week	-1.10	(-2.80,0.60)	0.868
	1-3/month	-0.51	(-2.13,1.11)	0.825
	< 1/month	-1.14	(-2.71,0.43)	0.801
Year of birth	(Ref: 1998-2005; age 18-24)			
	1988-1998; age 25-34	1.77	(-0.16,3.70)	0.985
	1978-1988; age 35-44	2.07	(0.28,3.86)	0.913
	1968-1978; age 45-54	3.17	(1.18,5.17)	1.018
	1958-1968; age 55-64	3.22	(1.14,5.31)	1.064
	1948-1957; age 65-74	2.51	(0.39,4.63)	1.081
	1938-1948; age 75-84	3.05	(-0.32,6.41)	1.717
	1938 or earlier; 85 or older	2.23	(-0.63,5.08)	1.455
Gender	(Ref: Male)			
	Female	-0.08	(-2.70,2.54)	1.339
	Other	-2.44	(-5.33,0.45)	1.475

228. Table S228. Population weighted meta-analysis of E-values (continuous).

Variable	Category	E-value	E-value limit
Relationship with mother	(Ref: Very bad/somewhat bad)		
	Very good/somewhat good	1.75	1.00
Relationship with father	(Ref: Very bad/somewhat bad)		
	Very good/somewhat good	2.81	1.00
Parent marital status	(Ref: Parents married)		
	Divorced	1.90	1.00
	Single, never married	1.39	1.00
	One or both parents had died	1.71	1.00
Subjective financial status of family growing up	(Ref: Got by)		
	Lived comfortably	1.19	1.00
	Found it difficult	1.12	1.00
	Found it very difficult	1.94	1.00
Abuse	(Ref: No)		
	Yes	1.68	1.00
Outsider growing up	(Ref: No)		
	Yes	1.29	1.00
Self-rated health growing up	(Ref: Good)		
	Excellent	1.17	1.00
	Very good	1.21	1.00
	Fair	1.45	1.00
	Poor	3.50	1.15
Immigration status	(Ref: Born in this country)		
	Born in another country	1.61	1.00
Age 12 religious service attendance	(Ref: Never)		
	At least 1/week	1.70	1.00
	1-3/month	1.41	1.00
	< 1/month	1.73	1.00
Year of birth	(Ref: 1998-2005; age 18-24)		
	1988-1998; age 25-34	2.04	1.00
	1978-1988; age 35-44	2.20	1.28
	1968-1978; age 45-54	2.82	1.74
	1958-1968; age 55-64	2.85	1.73
	1948-1957; age 65-74	2.44	1.34
	1938-1948; age 75-84	2.75	1.00
	1938 or earlier; 85 or older	2.28	1.00
Gender	(Ref: Male)		
	Female	1.13	1.00
	Other	2.40	1.00

229. Table S229. Summary statistics of daily smoking outcome by country with missingness: Total (N = 202,898) and smoker samples (N = 38,290)

	Total sample				Smoker sample	
	N	Number of cigarettes smoked per day Mean (SD)	At least one cigarette smoked per day N (%)	Missing N (%)	N	Number of cigarettes smoked per day Mean (SD)
Overall	202,898	2.0 (5.7)	34,994 (18%)	3,296(1.6%)	38,290	11.3 (9.1)
Country						
Argentina	6,724	3.9 (8.0)	2,184 (33%)	109 (1.6%)	2,293	11.8 (10.1)
Australia	3,844	1.3 (4.6)	439 (12%)	44 (1.1%)	483	11.4 (8.3)
Brazil	13,204	2.4 (6.4)	2,546 (20%)	239 (1.8%)	2,785	12.2 (9.6)
Egypt	4,729	3.3 (7.6)	1,051 (22%)	34 (0.7%)	1,085	14.9 (9.5)
Germany	9,506	3.7 (7.8)	2,508 (27%)	83 (0.9%)	2,590	13.8 (9.3)
Hong Kong	3,012	2.0 (4.2)	926 (31%)	31 (1.0%)	956	6.4 (5.3)
India	12,765	0.7 (4.7)	971 (7.9%)	513 (4.0%)	1,484	8.3 (14.8)
Indonesia	6,992	3.9 (6.6)	2,516 (38%)	428 (6.1%)	2,944	10.2 (7.0)
Israel	3,669	3.1 (7.3)	854 (23%)	28 (0.8%)	882	13.1 (9.8)
Japan	20,543	3.0 (6.8)	4,515 (22%)	252 (1.2%)	4,766	13.6 (8.2)
Kenya	11,389	0.3 (1.9)	602 (5.3%)	111 (1.0%)	713	5.3 (6.6)
Mexico	5,776	1.2 (4.1)	1,316 (23%)	122 (2.1%)	1,438	5.3 (7.2)
Nigeria	6,827	0.3 (2.5)	305 (4.5%)	64 (0.9%)	368	5.8 (10.7)
Philippines	5,292	1.7 (4.6)	1,133 (22%)	81 (1.5%)	1,214	8.0 (6.7)
Poland	10,389	3.7 (7.0)	3,127 (30%)	102 (1.0%)	3,230	12.2 (7.6)
South Africa	2,651	1.7 (4.6)	625 (24%)	44 (1.7%)	669	7.0 (7.1)
Spain	6,290	3.6 (6.8)	2,009 (32%)	73 (1.2%)	2,082	11.2 (7.7)
Sweden	15,068	0.8 (3.3)	1,401 (9.6%)	457 (3.0%)	1,858	8.8 (6.8)
Tanzania	9,075	0.2 (1.8)	365 (4.1%)	193 (2.1%)	557	5.7 (7.0)
Türkiye	1,473	9.8 (13.3)	779 (53%)	10 (0.7%)	789	18.4 (13.1)
United Kingdom	5,368	1.8 (5.1)	873 (16%)	54 (1.0%)	928	10.7 (7.9)
United States	38,312	1.2 (4.6)	3,951 (10%)	226 (0.6%)	4,176	12.1 (8.6)

Figure S1. Forest plot for `Relationship with mother`-`Very good/somewhat good` effect

Relationship with mother (Ref: Very bad/somewhat bad)

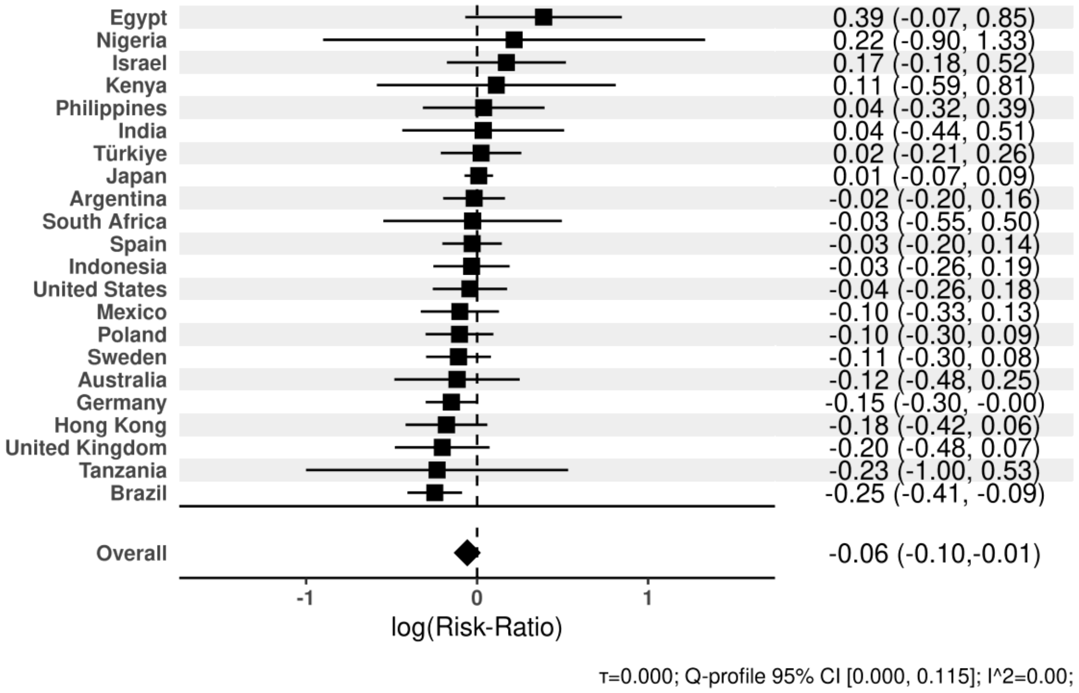

Figure S2. Forest plot for `Relationship with father`-`Very good/somewhat good` effect

Relationship with father (Ref: Very bad/somewhat bad)

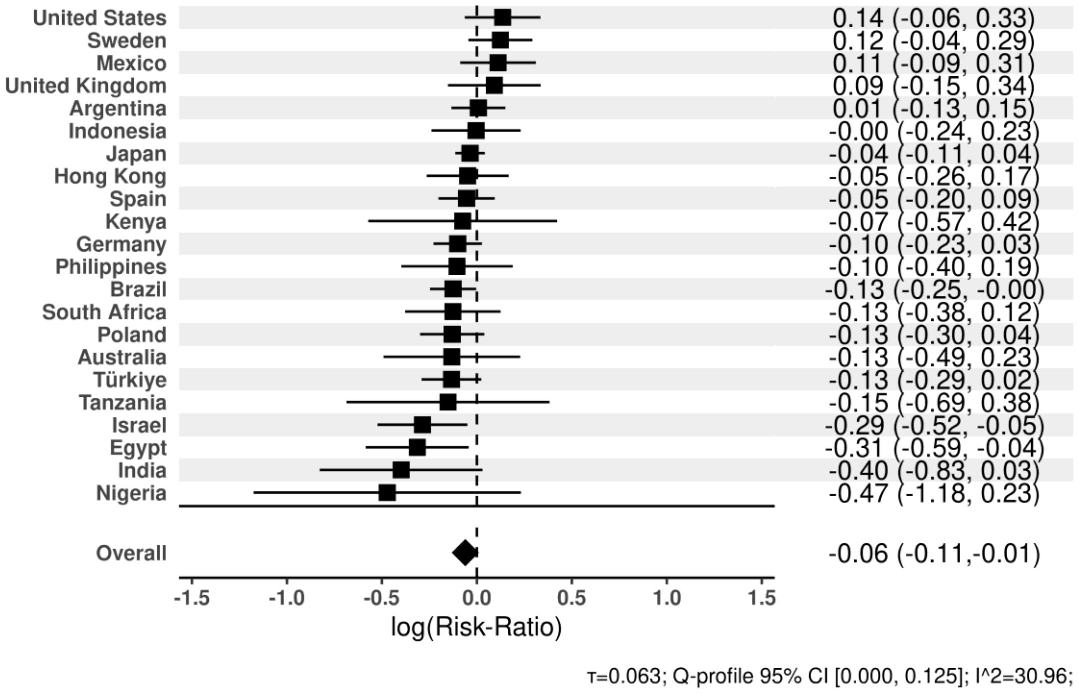

Figure S3. Forest plot for `Parent marital status`-`Divorced` effect

Parent marital status (Ref: Parents married)

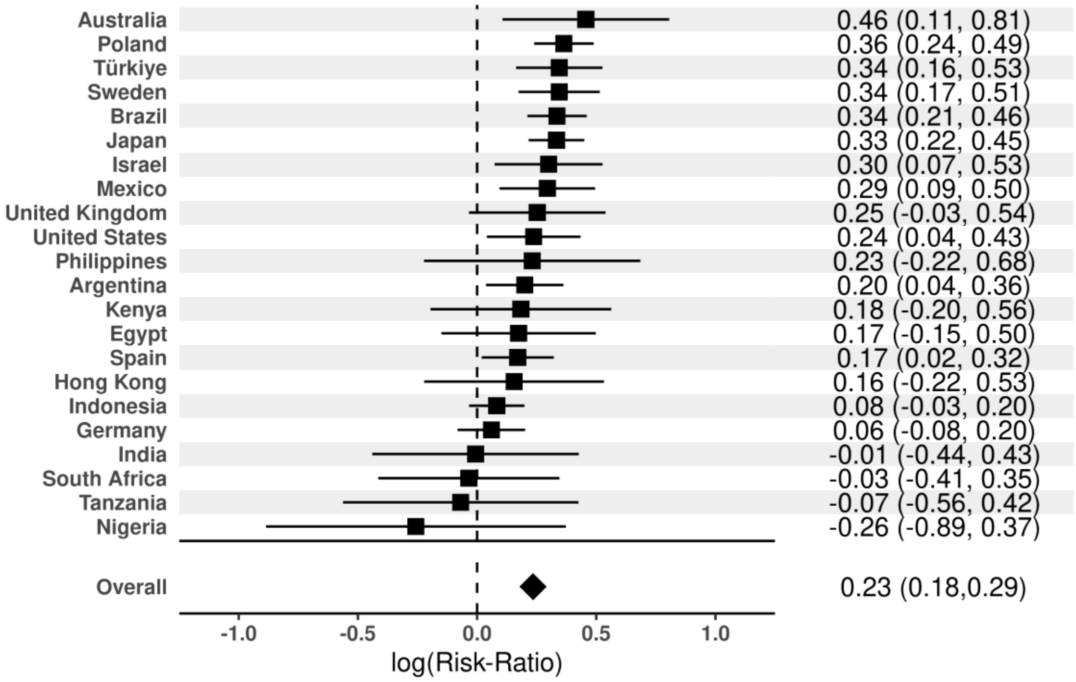

τ=0.069; Q-profile 95% CI [0.026, 0.144]; I^2=32.75;

Figure S4. Forest plot for `Parent marital status`-`Single, never married` effect

Parent marital status (Ref: Parents married)

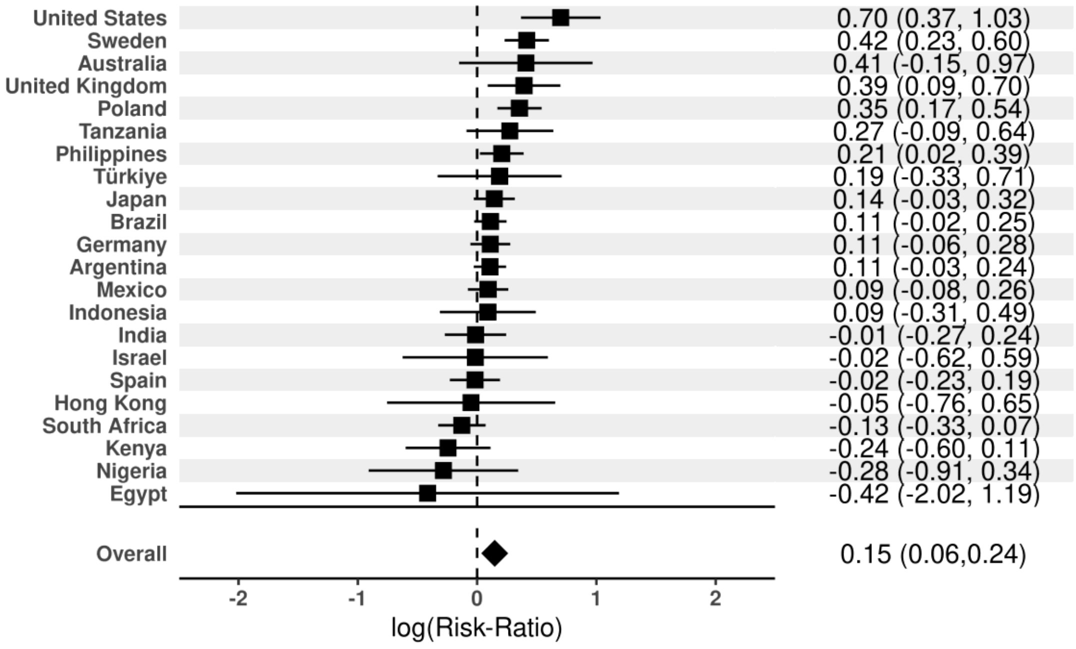

τ=0.152; Q-profile 95% CI [0.060, 0.249]; I^2=62.05;

Figure S5. Forest plot for `Parent marital status`-`One or both parents had died` effect

Parent marital status (Ref: Parents married)

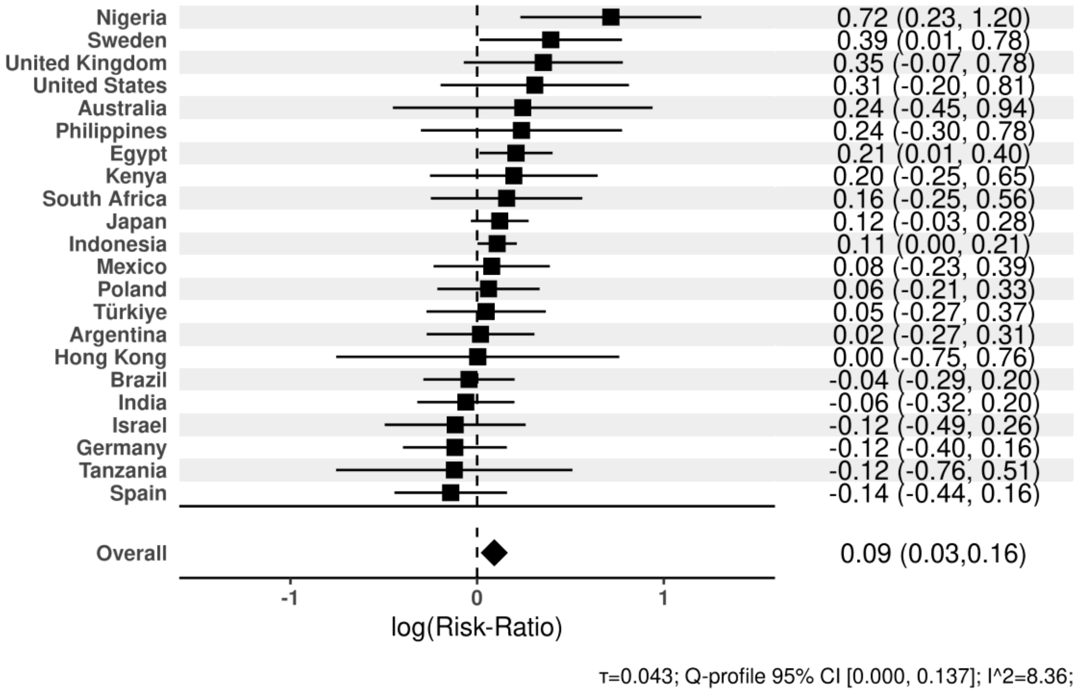

Figure S6. Forest plot for `Subjective financial status of family growing up`-`Lived comfortably` effect

Subjective financial status of family growing up (Ref: Got by)

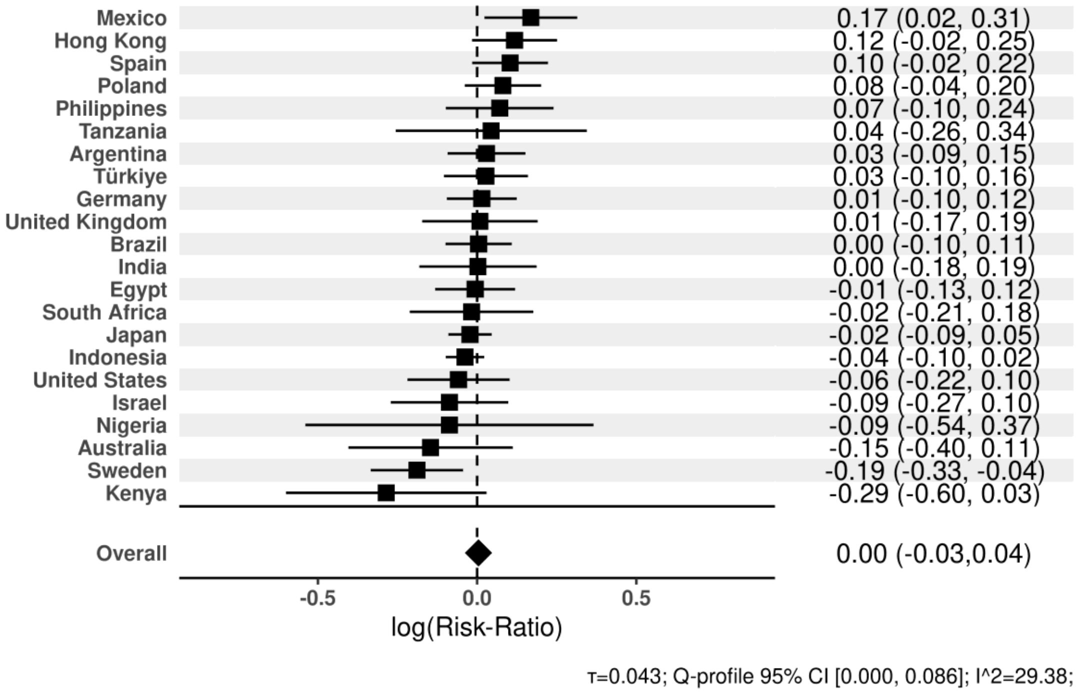

Figure S7. Forest plot for `Subjective financial status of family growing up`-`Found it difficult` effect

Subjective financial status of family growing up (Ref: Got by)

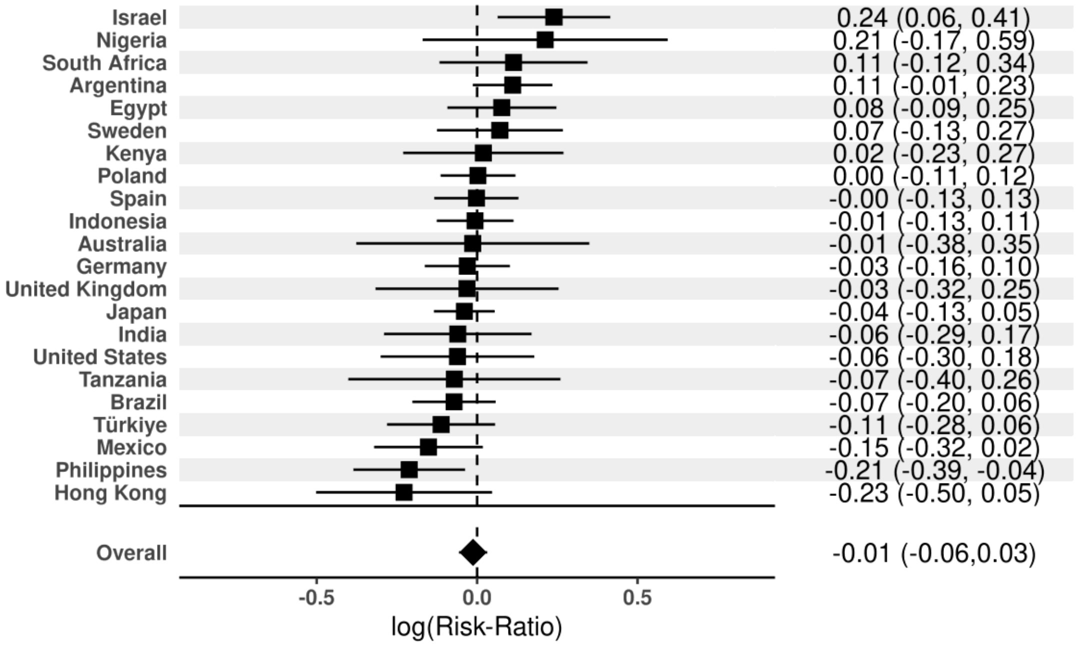

τ=0.055; Q-profile 95% CI [0.000, 0.113]; I^2=29.60;

Figure S8. Forest plot for `Subjective financial status of family growing up`-`Found it very difficult` effect

Subjective financial status of family growing up (Ref: Got by)

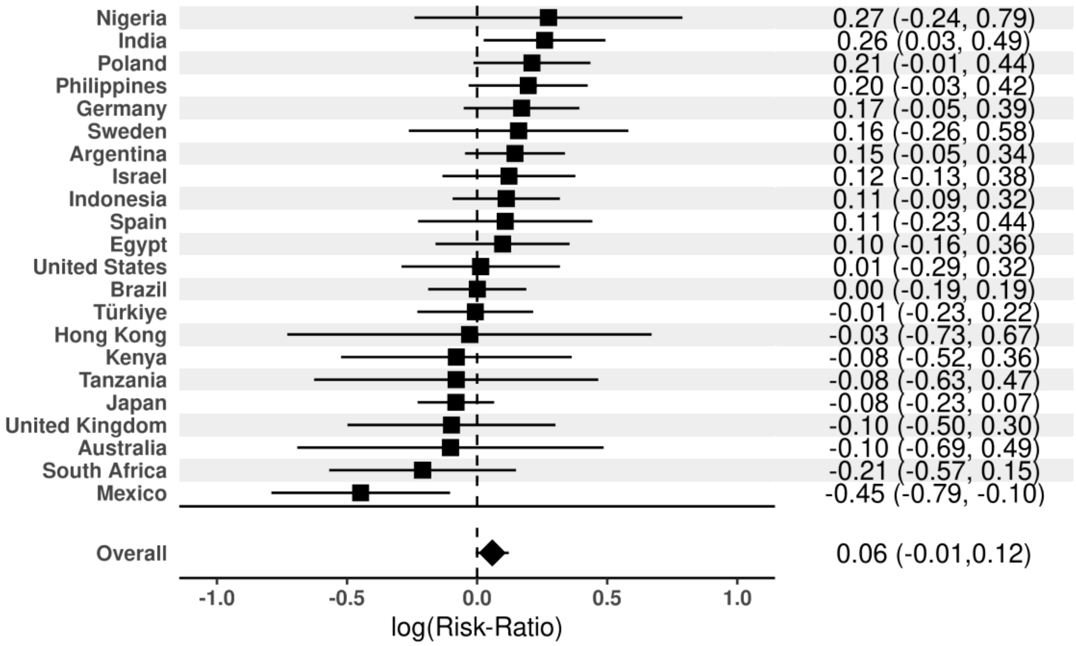

τ=0.061; Q-profile 95% CI [0.000, 0.159]; I^2=16.53;

Figure S9. Forest plot for `Abuse`-`Yes` effect

Abuse (Ref: No)

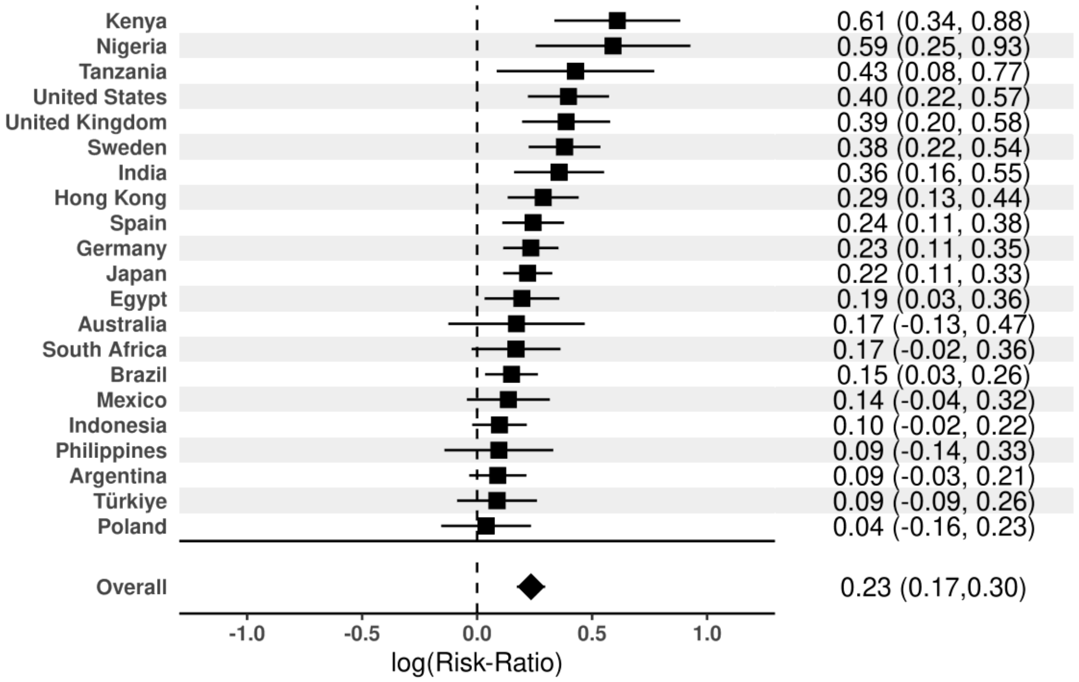

τ=0.111; Q-profile 95% CI [0.022, 0.166]; I^2=64.08;
Excluded countries: Israel

Figure S10. Forest plot for `Outsider growing up`-`Yes` effect

Outsider growing up (Ref: No)

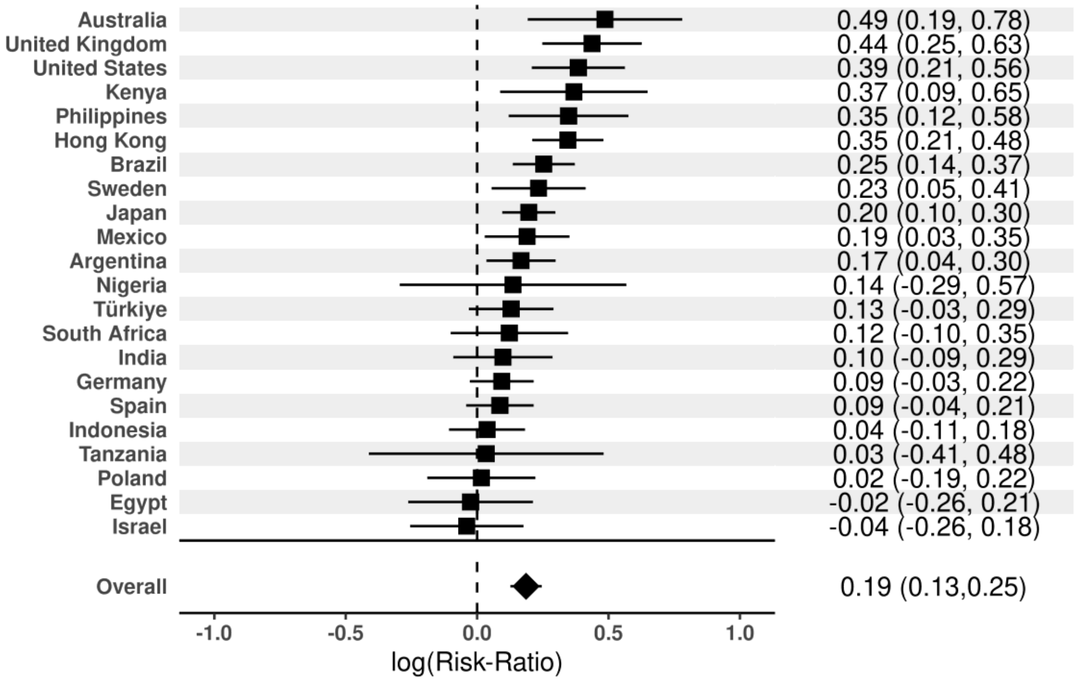

τ=0.105; Q-profile 95% CI [0.037, 0.165]; I^2=59.84;

Figure S11. Forest plot for `Self-rated health growing up`-`Excellent` effect

Self-rated health growing up (Ref: Good)

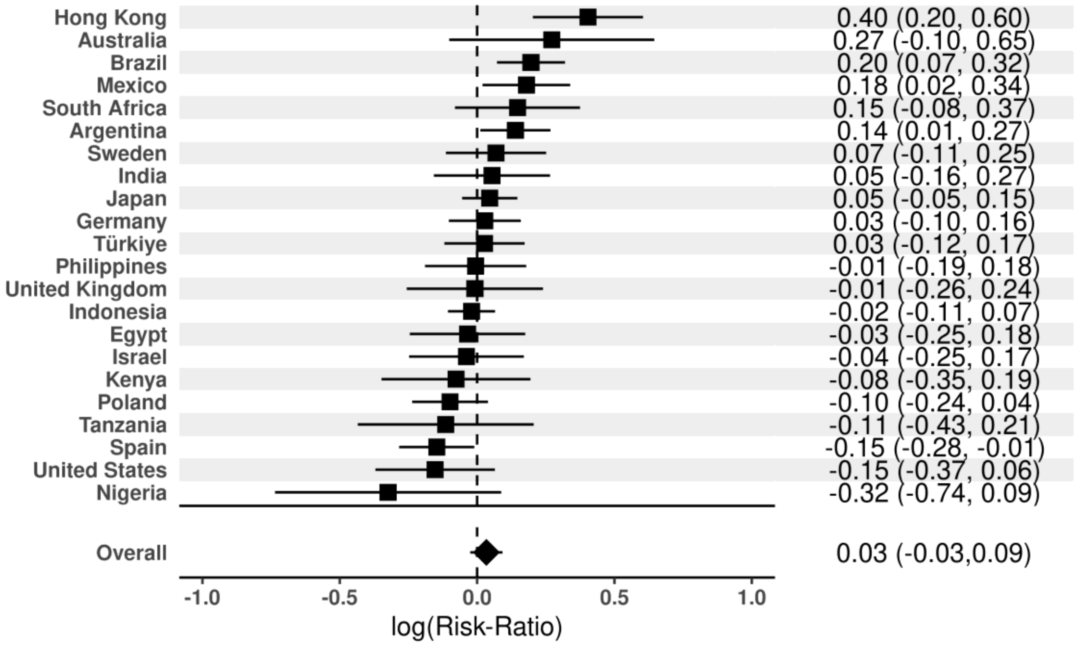

τ=0.103; Q-profile 95% CI [0.041, 0.161]; I^2=60.77;

Figure S12. Forest plot for `Self-rated health growing up`-`Very good` effect

Self-rated health growing up (Ref: Good)

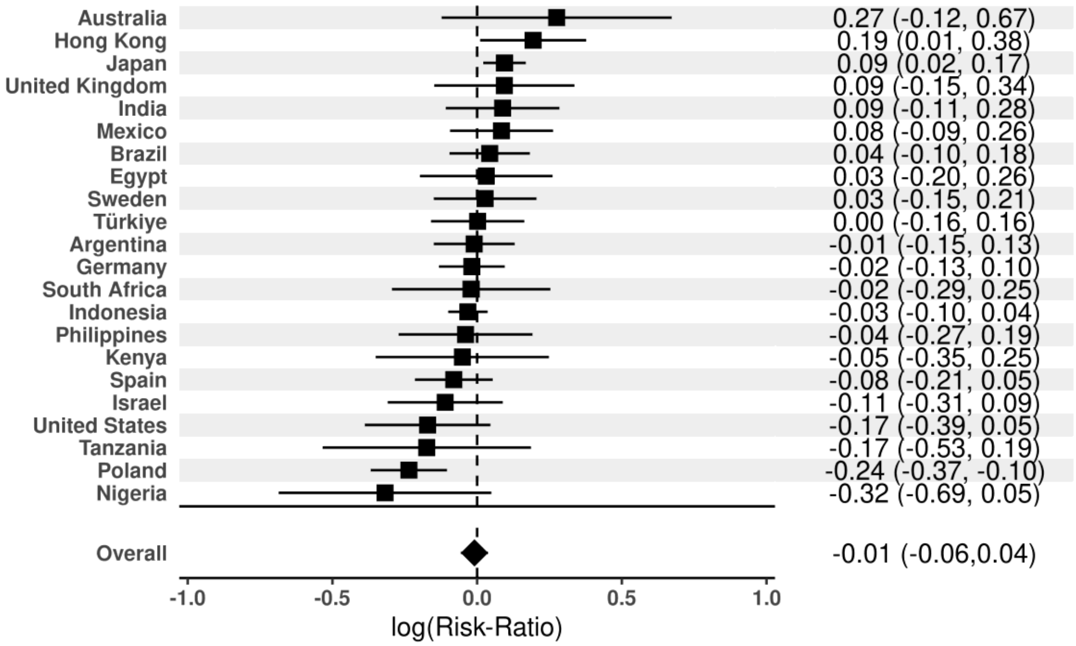

τ=0.067; Q-profile 95% CI [0.020, 0.123]; I^2=42.21;

Figure S13. Forest plot for `Self-rated health growing up`-`Fair` effect

Self-rated health growing up (Ref: Good)

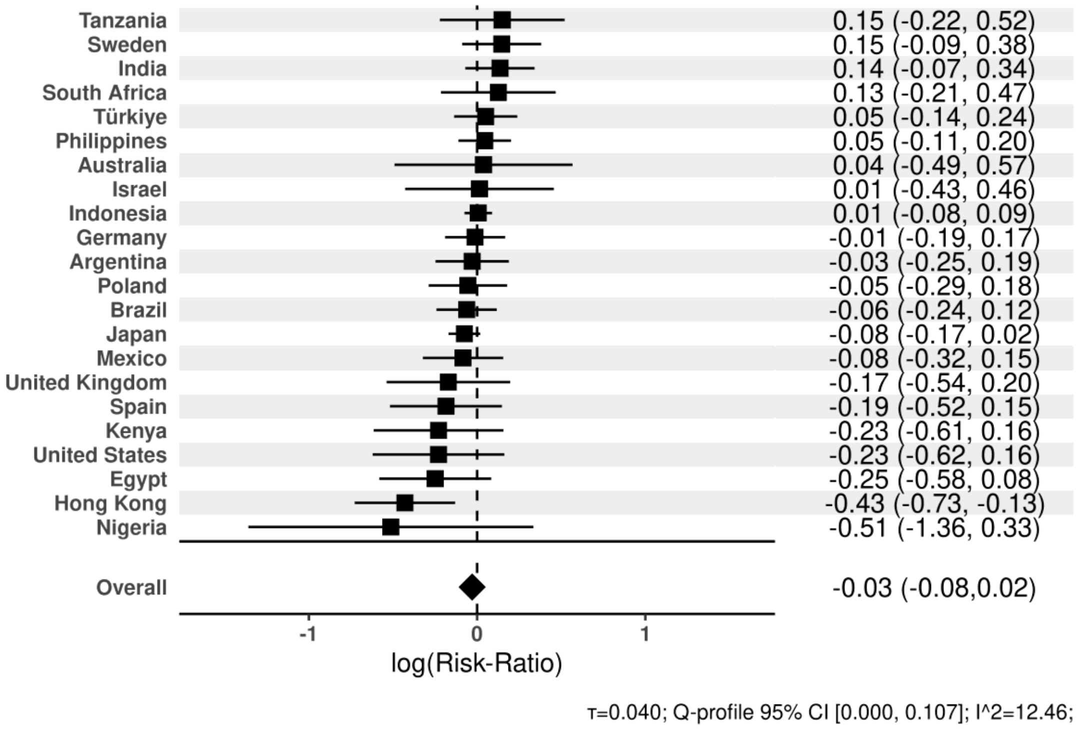

Figure S14. Forest plot for `Self-rated health growing up`-`Poor` effect

Self-rated health growing up (Ref: Good)

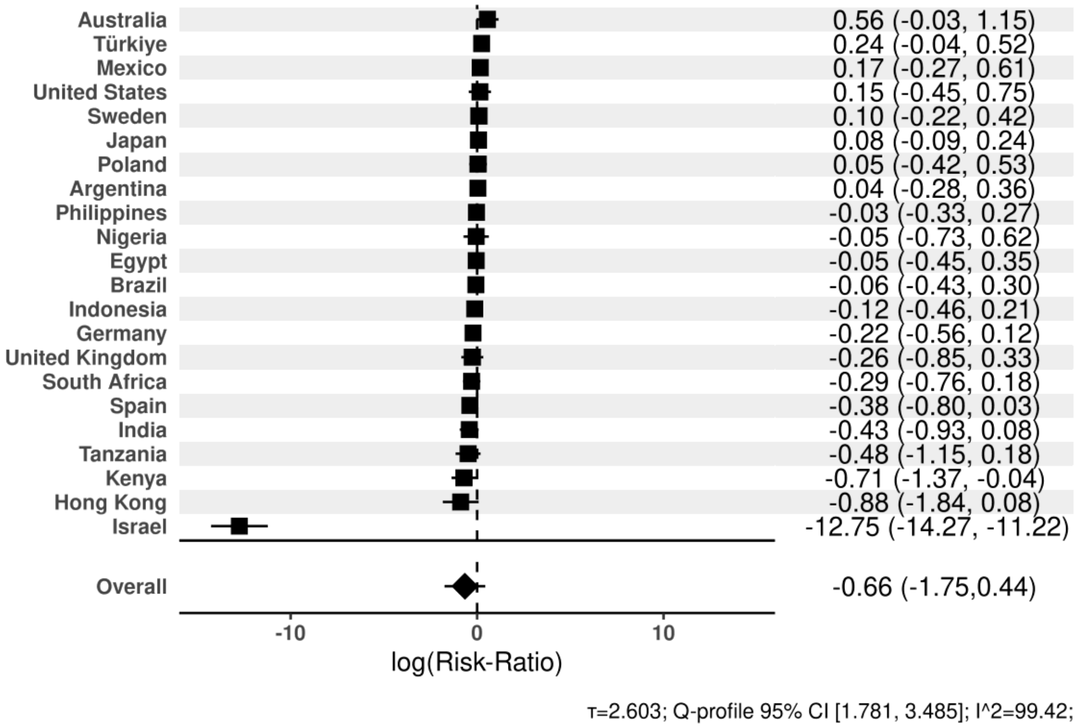

Figure S15. Forest plot for `Immigration status`-`Born in another country` effect

Immigration status (Ref: Born in this country)

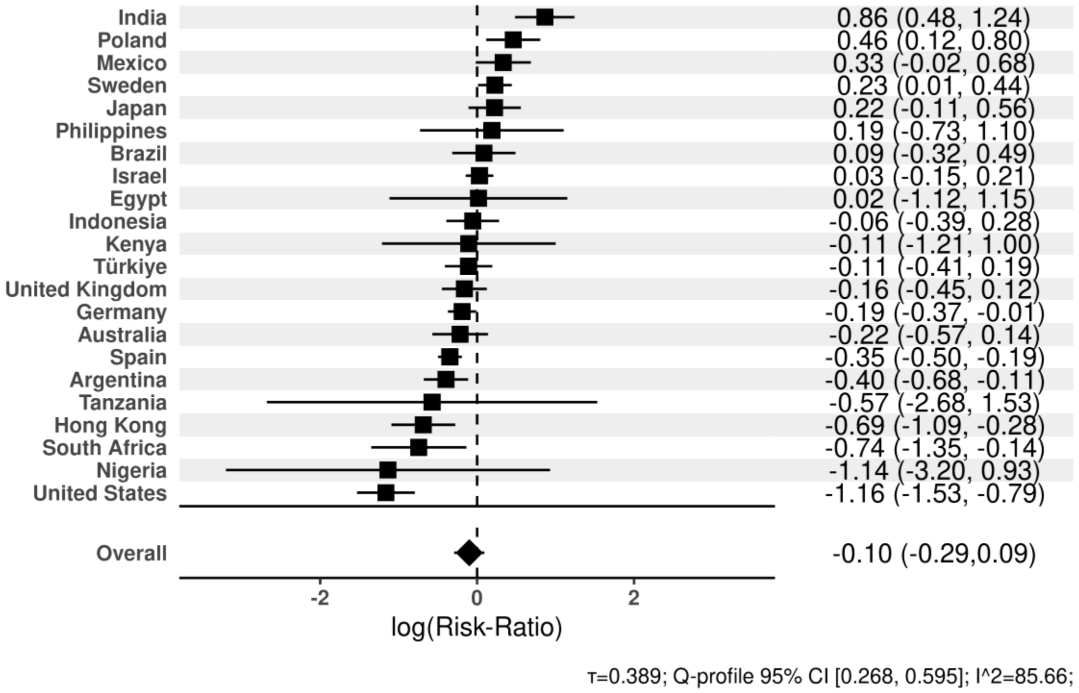

Figure S16. Forest plot for `Age 12 religious service attendance`-`At least 1/week` effect

Age 12 religious service attendance (Ref: Never)

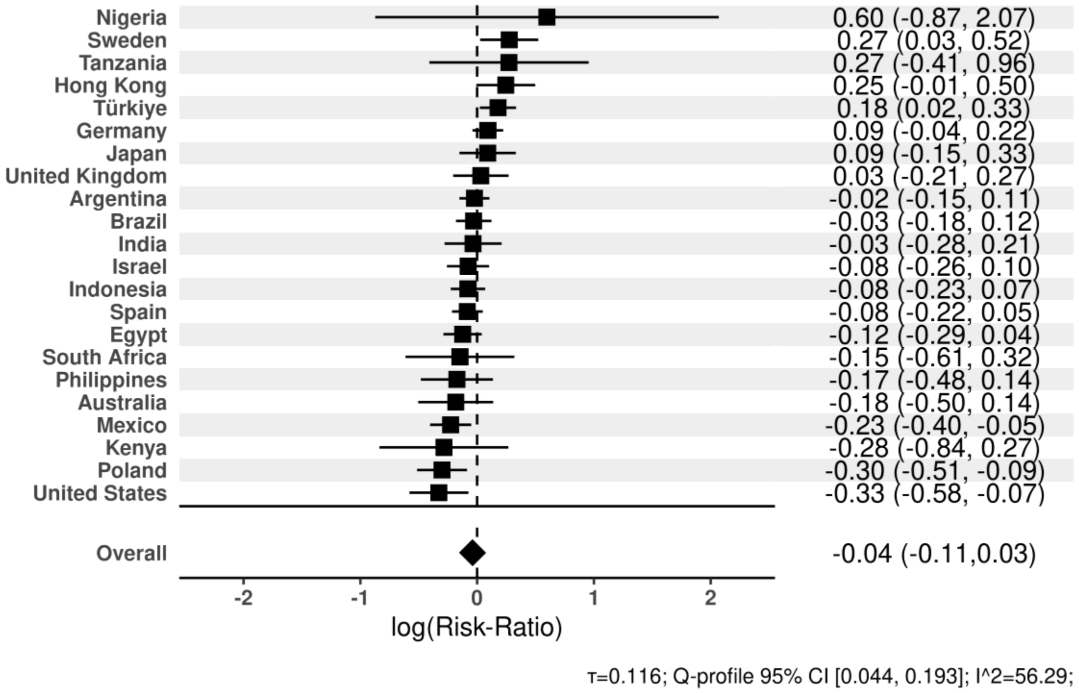

Figure S17. Forest plot for `Age 12 religious service attendance`-`1-3/month` effect

Age 12 religious service attendance (Ref: Never)

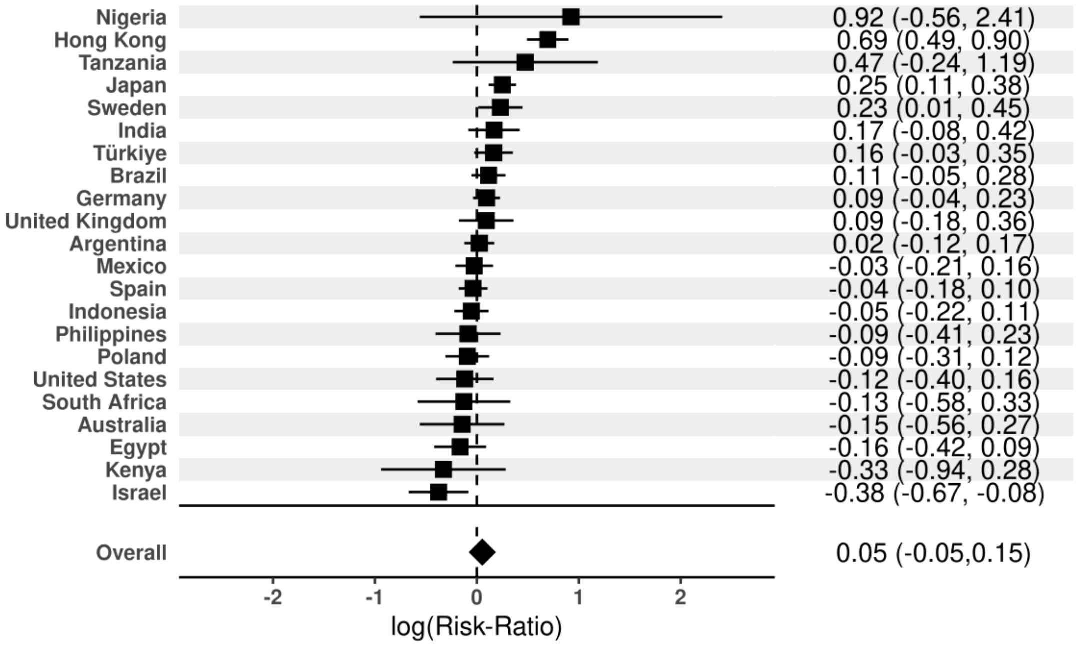

τ=0.192; Q-profile 95% CI [0.113, 0.287]; I^2=75.71;

Figure S18. Forest plot for `Age 12 religious service attendance`-`< 1/month` effect

Age 12 religious service attendance (Ref: Never)

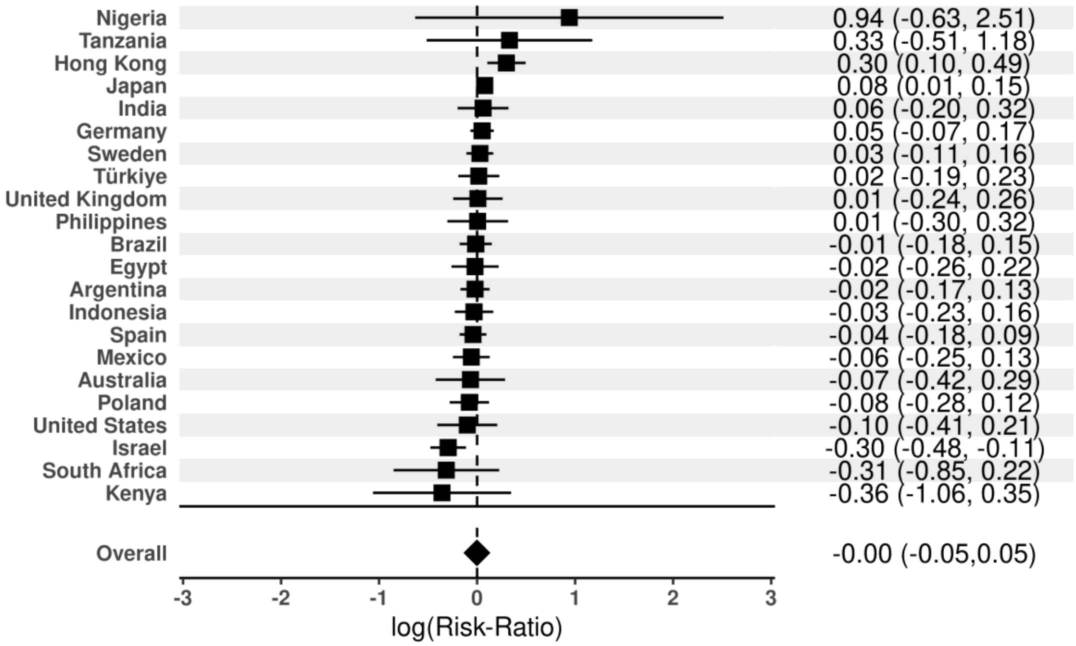

τ=0.062; Q-profile 95% CI [0.000, 0.132]; I^2=30.47;

Figure S19. Forest plot for `Year of birth`-`1988-1998; age 25-34` effect

Year of birth (Ref: 1998-2005; age 18-24)

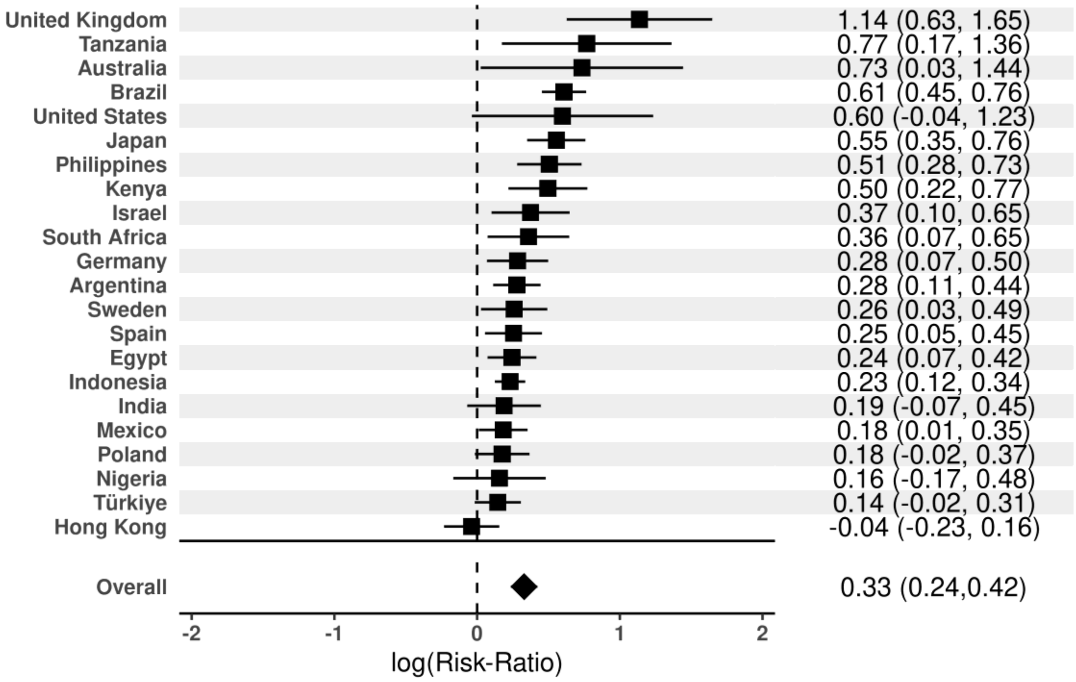

τ=0.180; Q-profile 95% CI [0.080, 0.257]; I^2=73.59;

Figure S20. Forest plot for `Year of birth`-`1978-1988; age 35-44` effect

Year of birth (Ref: 1998-2005; age 18-24)

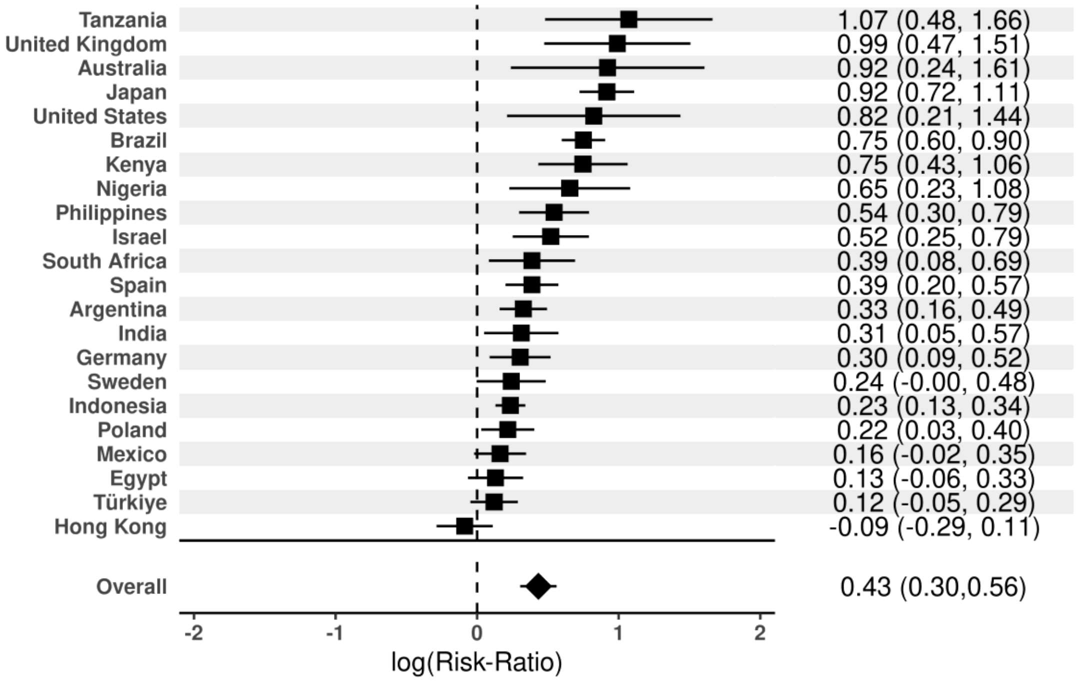

τ=0.271; Q-profile 95% CI [0.171, 0.385]; I^2=85.83;

Figure S21. Forest plot for `Year of birth`-`1968-1978; age 45-54` effect

Year of birth (Ref: 1998-2005; age 18-24)

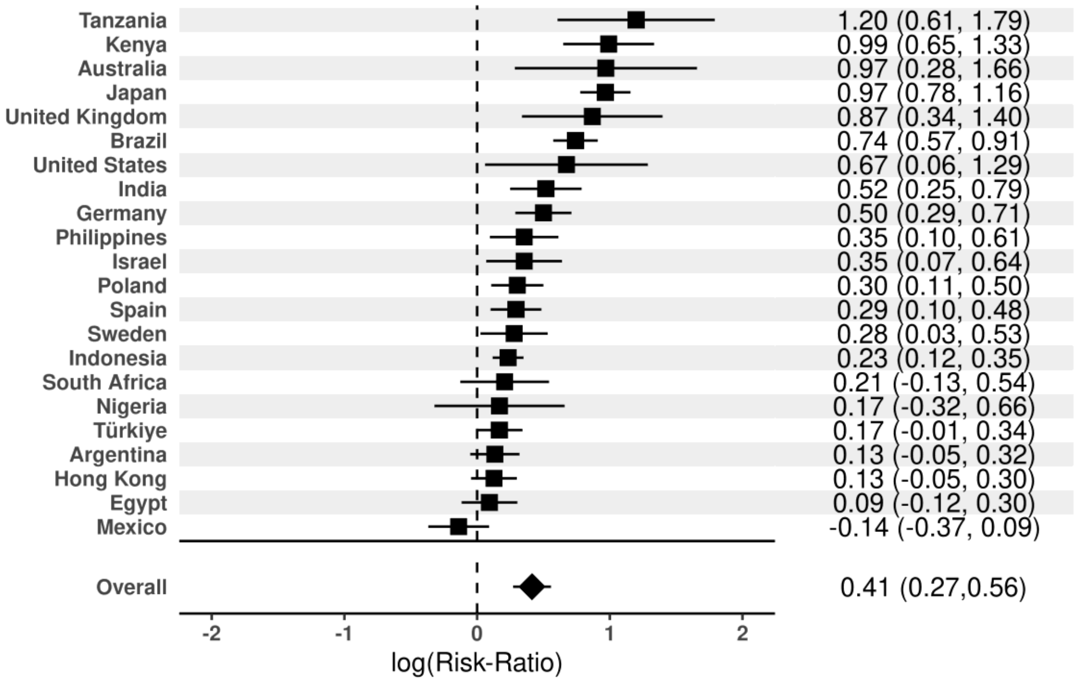

τ=0.304; Q-profile 95% CI [0.193, 0.429]; I^2=87.30;

Figure S22. Forest plot for `Year of birth`-`1958-1968; age 55-64` effect

Year of birth (Ref: 1998-2005; age 18-24)

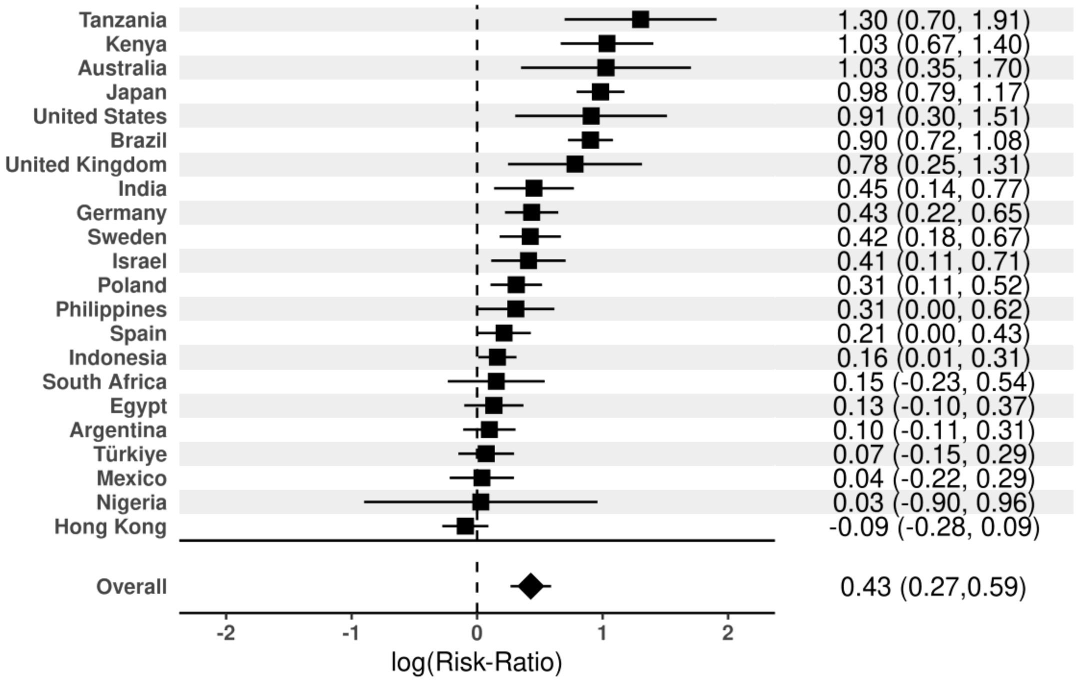

τ=0.346; Q-profile 95% CI [0.225, 0.491]; I^2=87.77;

Figure S23. Forest plot for `Year of birth`-`1948-1957; age 65-74` effect

Year of birth (Ref: 1998-2005; age 18-24)

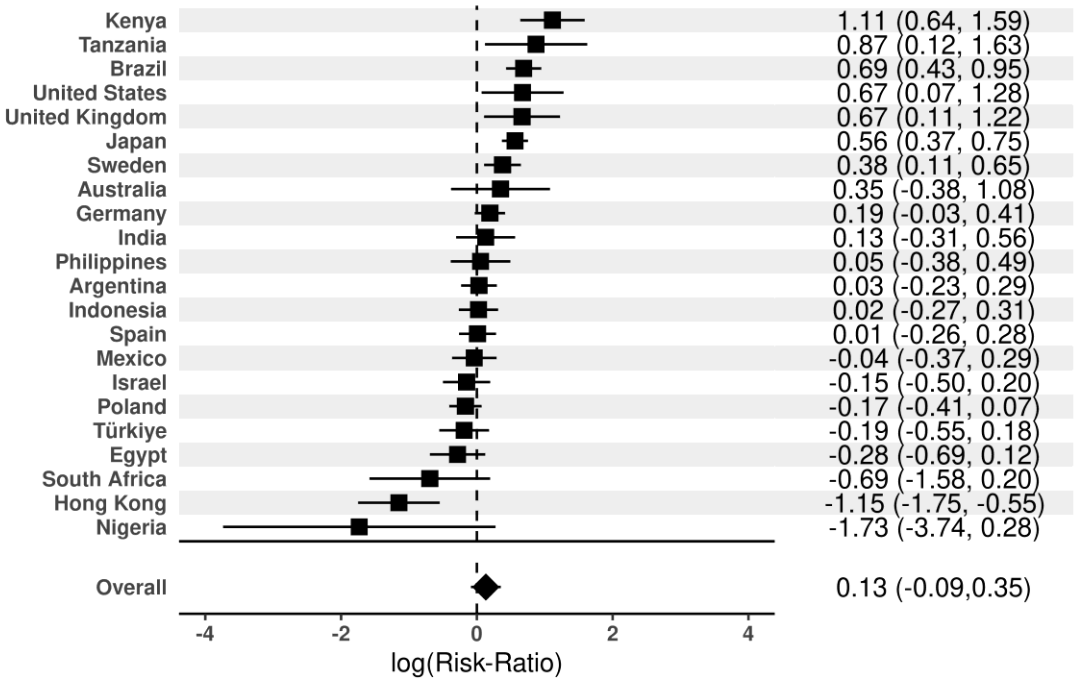

τ=0.468; Q-profile 95% CI [0.245, 0.638]; I^2=87.75;

Figure S24. Forest plot for `Year of birth`-`1938-1948; age 75-84` effect

Year of birth (Ref: 1998-2005; age 18-24)

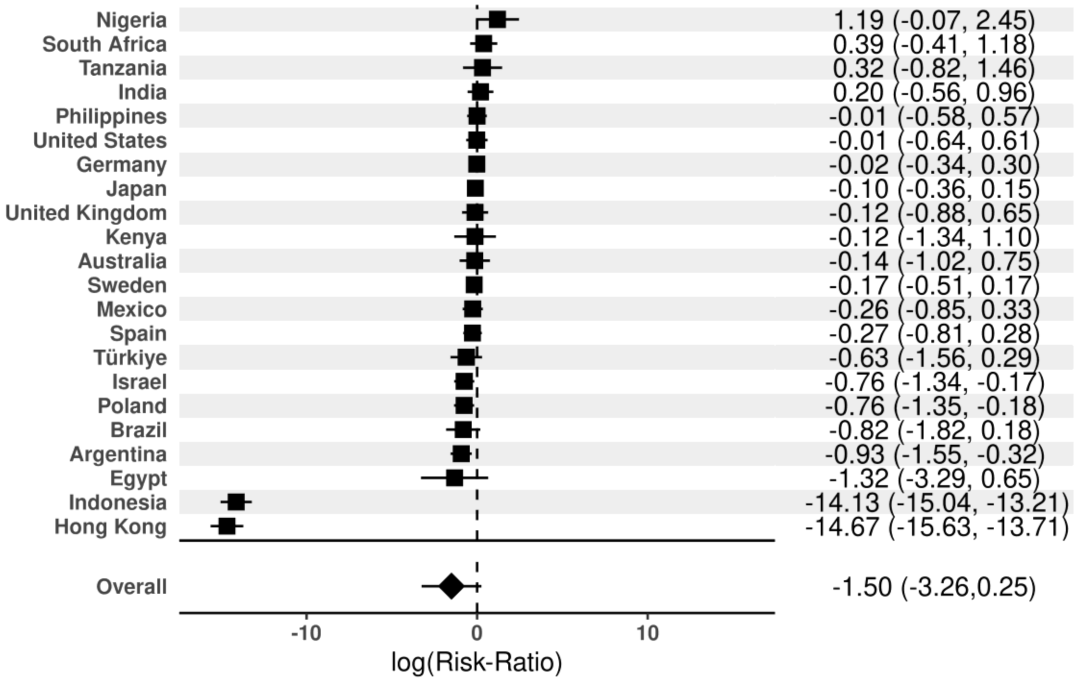

τ=4.179; Q-profile 95% CI [3.099, 5.686]; I^2=99.48;

Figure S25. Forest plot for `Year of birth`-`1938 or earlier; 85 or older` effect

Year of birth (Ref: 1998-2005; age 18-24)

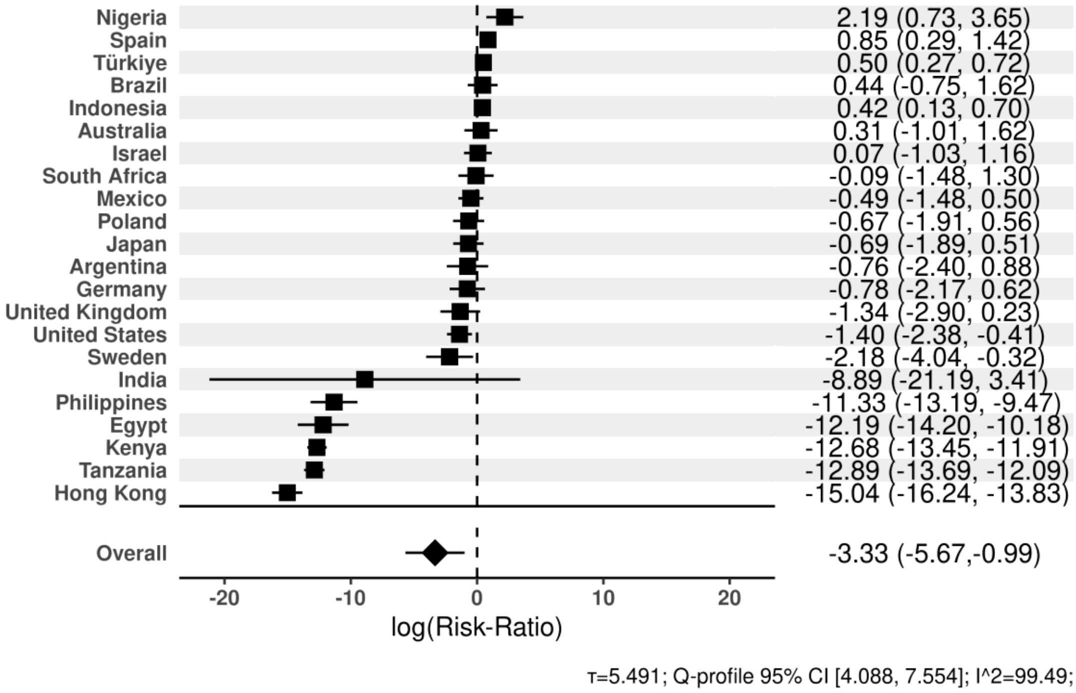

Figure S26. Forest plot for `Gender`-`Female` effect

Gender (Ref: Male)

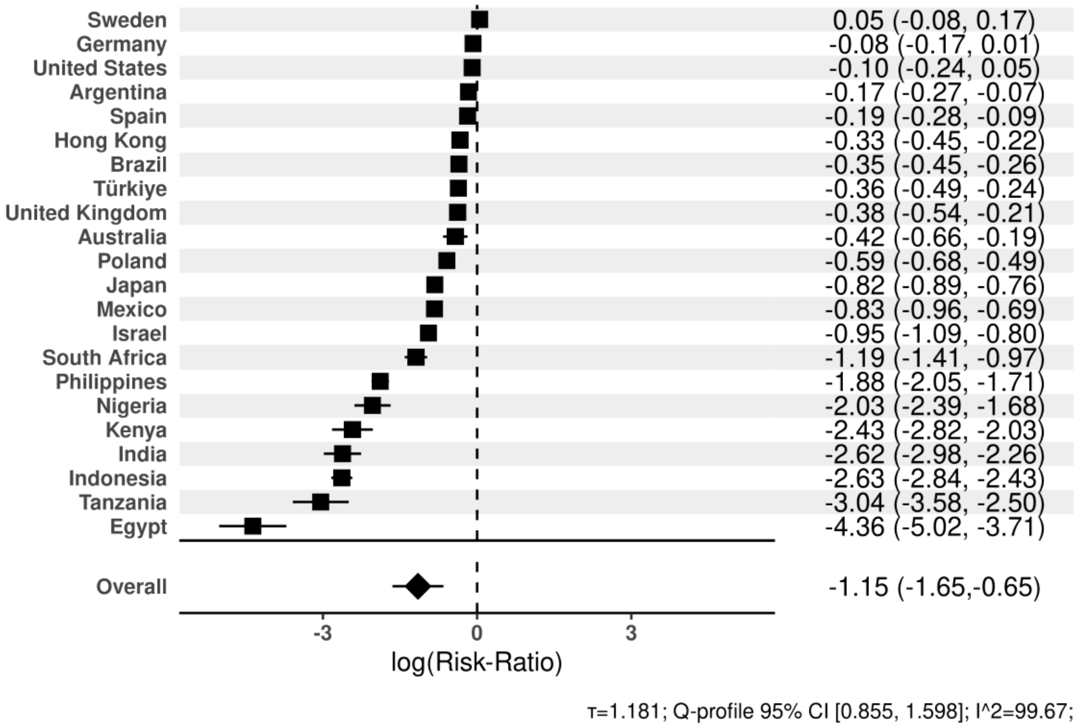

Figure S27. Forest plot for `Gender`-`Other` effect

Gender (Ref: Male)

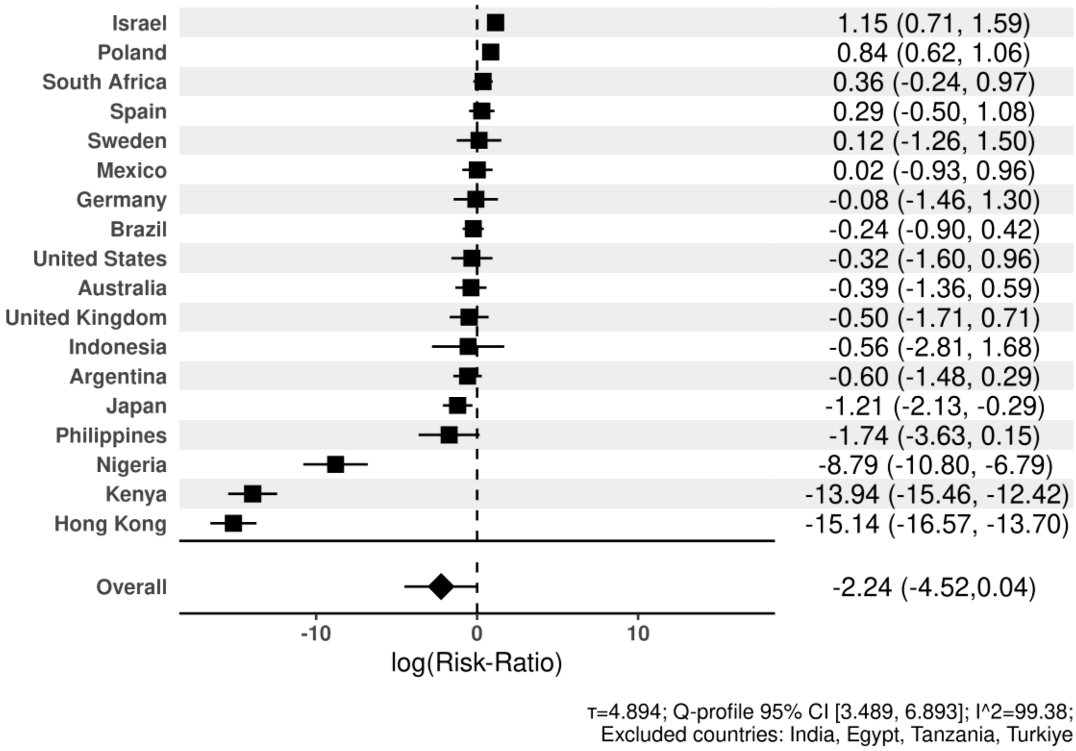

Figure S28.

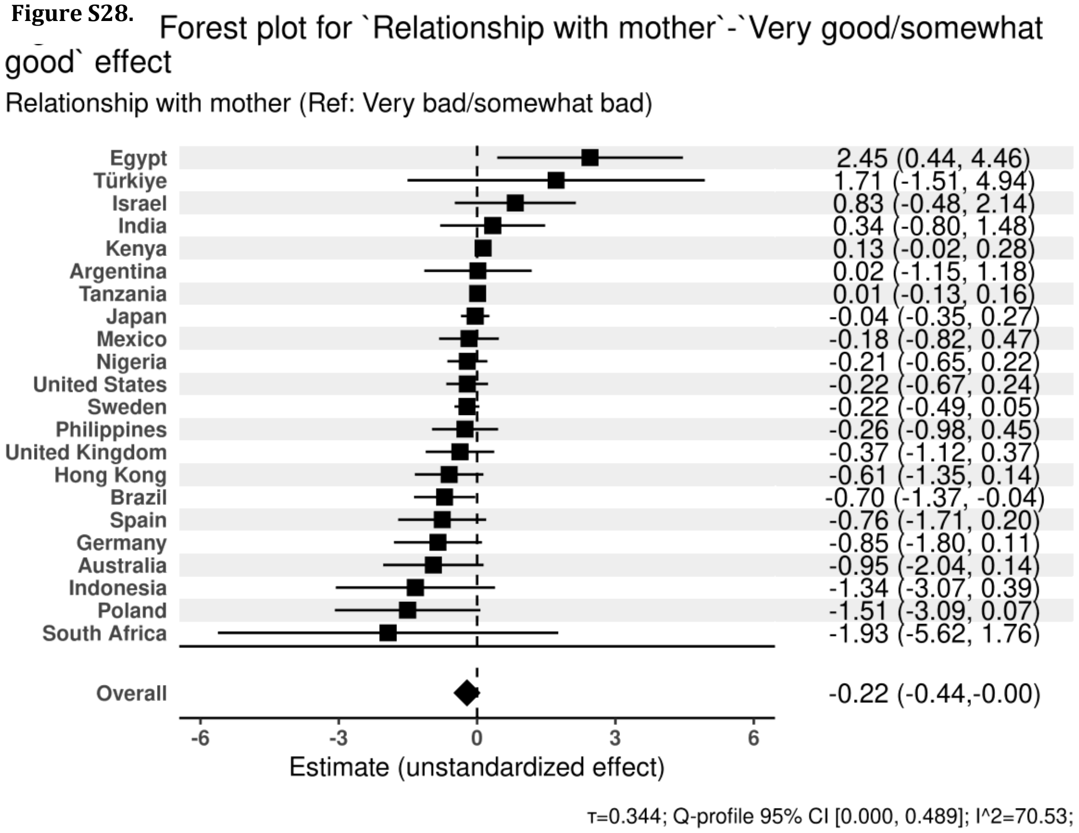

Figure S29.

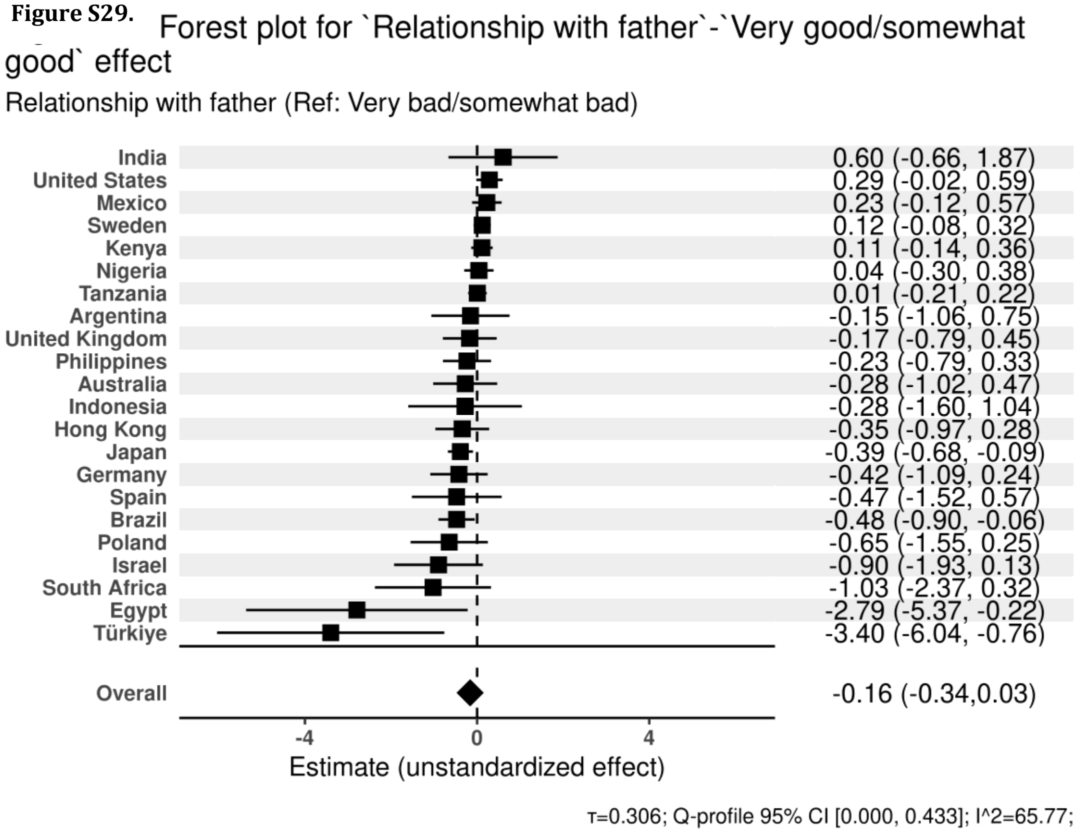

Figure S30. Forest plot for `Parent marital status`-`Divorced` effect

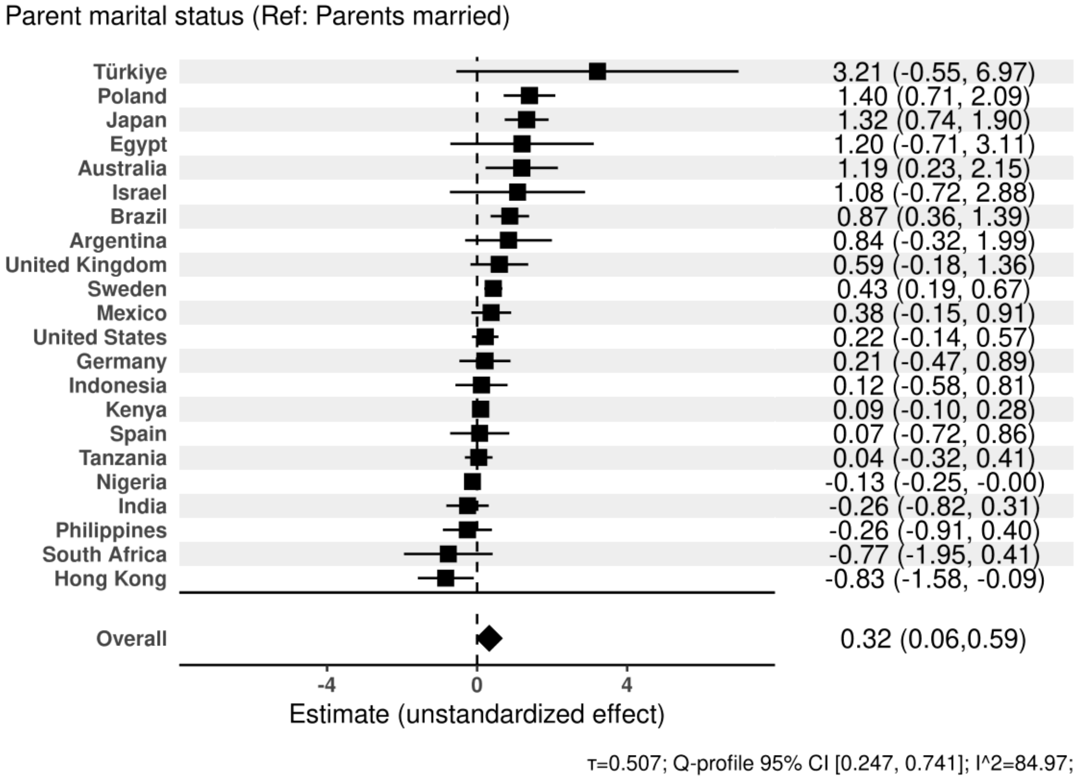

Figure S31. Forest plot for `Parent marital status`-`Single, never married` effect

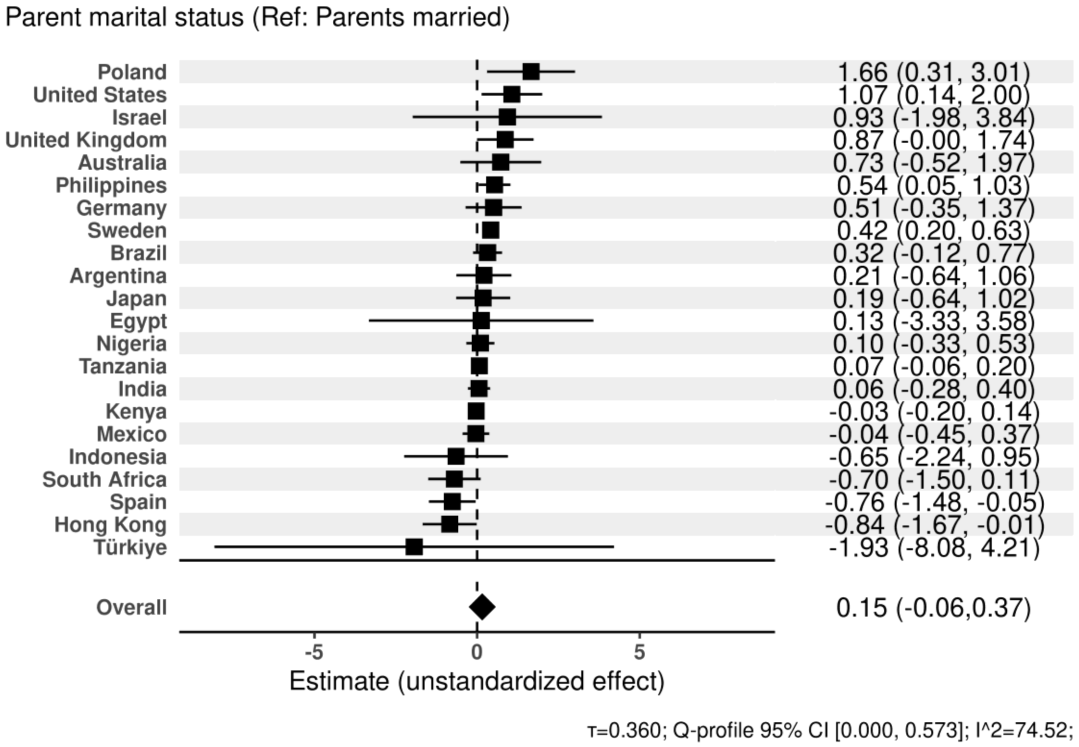

Figure S32. Forest plot for `Parent marital status`-`One or both parents had died` effect

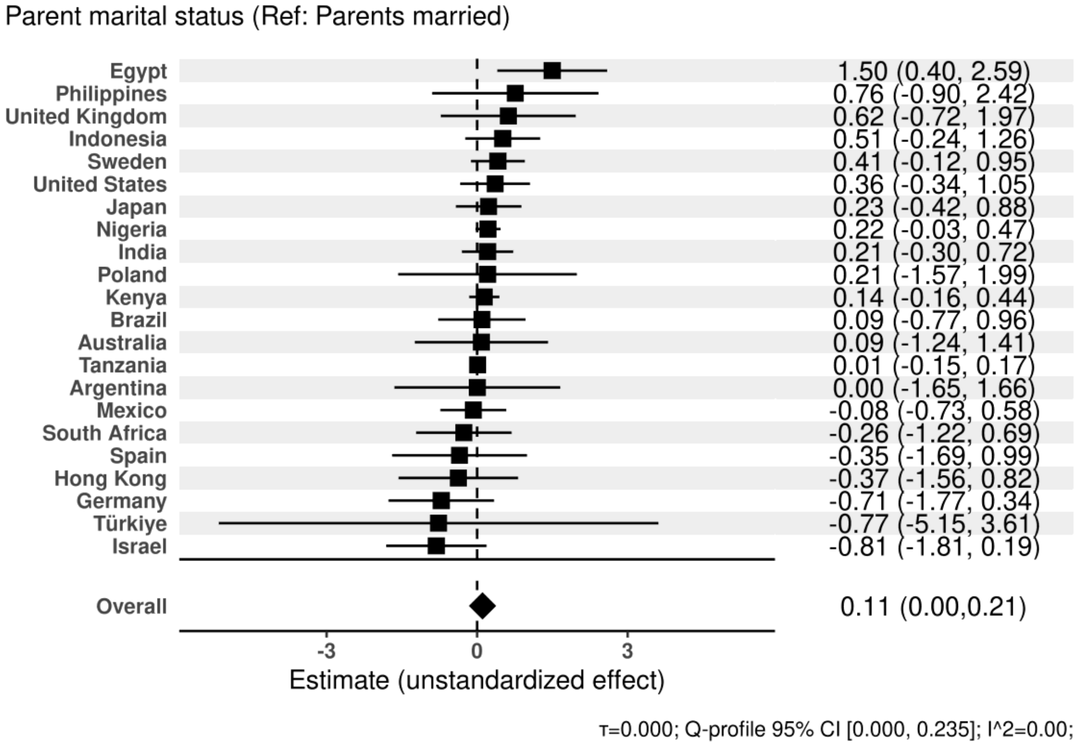

Figure S33. Forest plot for `Subjective financial status of family growing up`-`Lived comfortably` effect

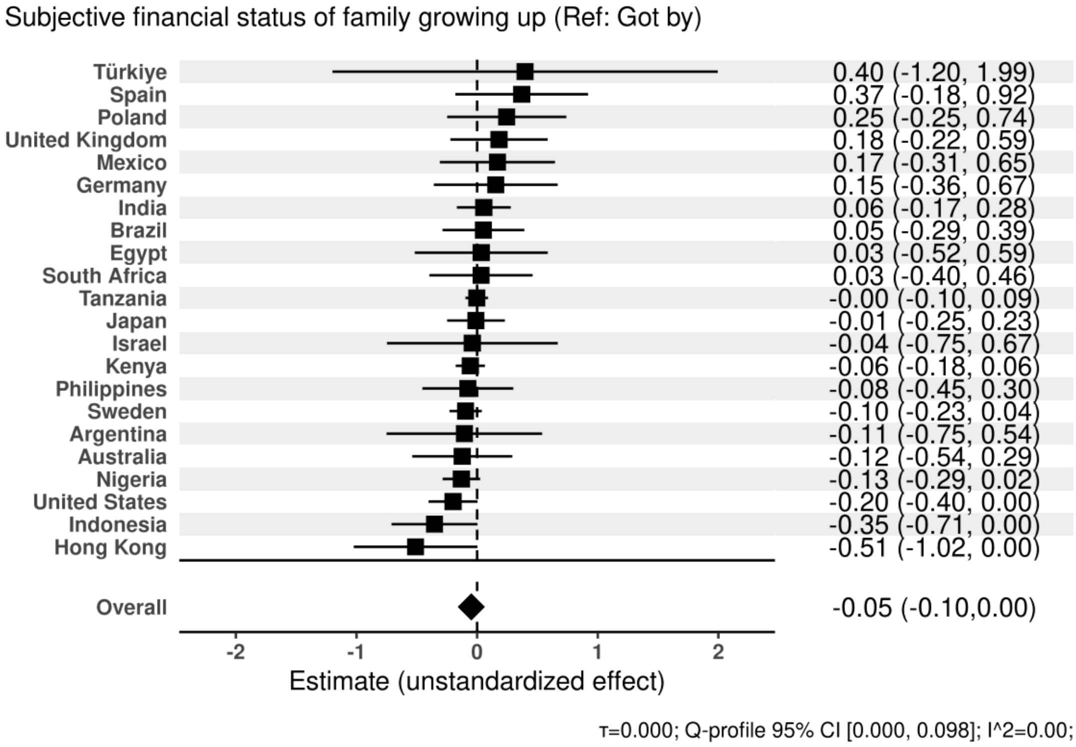

Figure S34. Forest plot for `Subjective financial status of family growing up`-`Found it difficult` effect

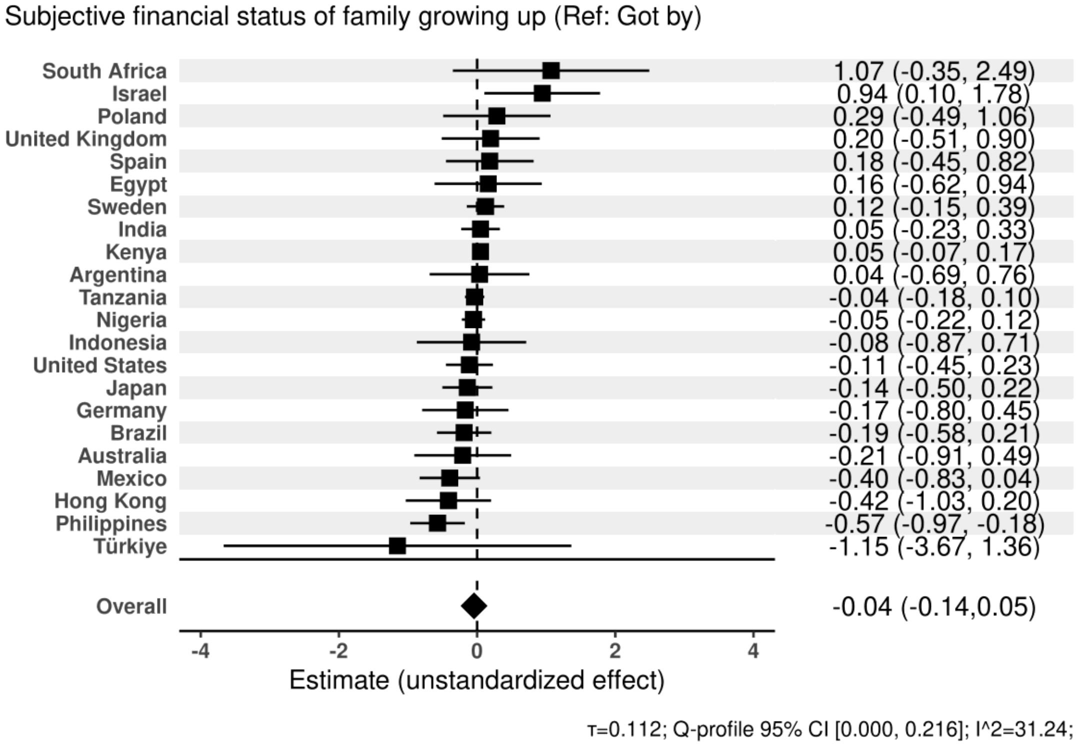

Figure S35. Forest plot for `Subjective financial status of family growing up`-`Found it very difficult` effect

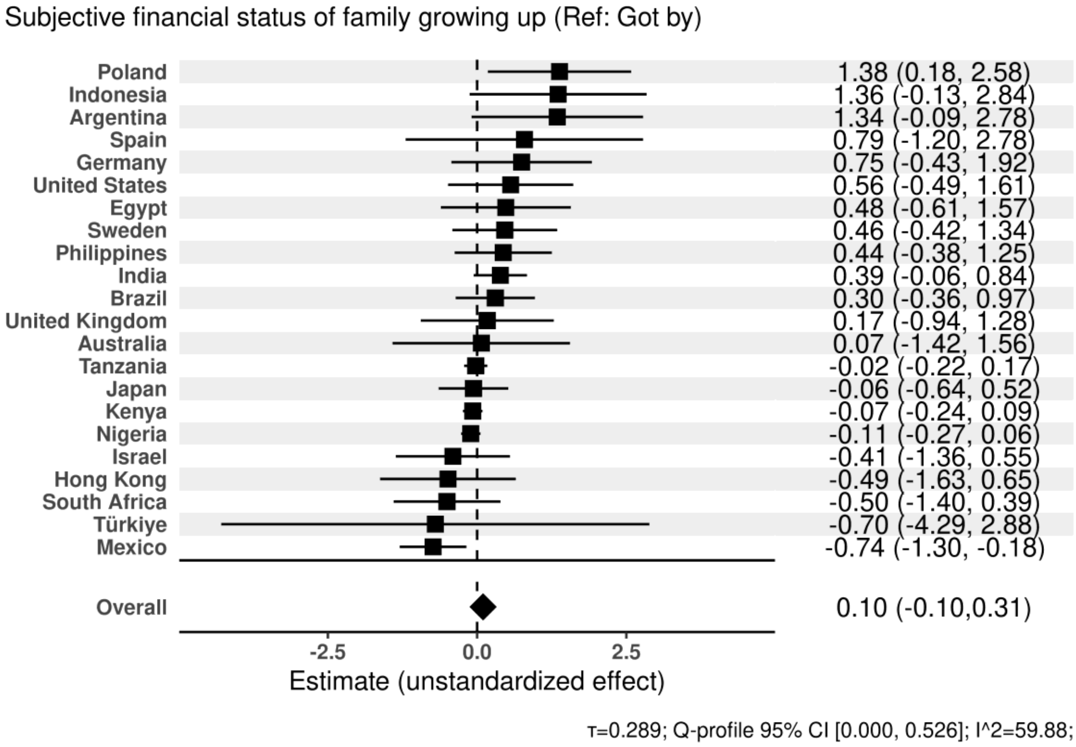

Figure S36. Forest plot for `Abuse`-`Yes` effect

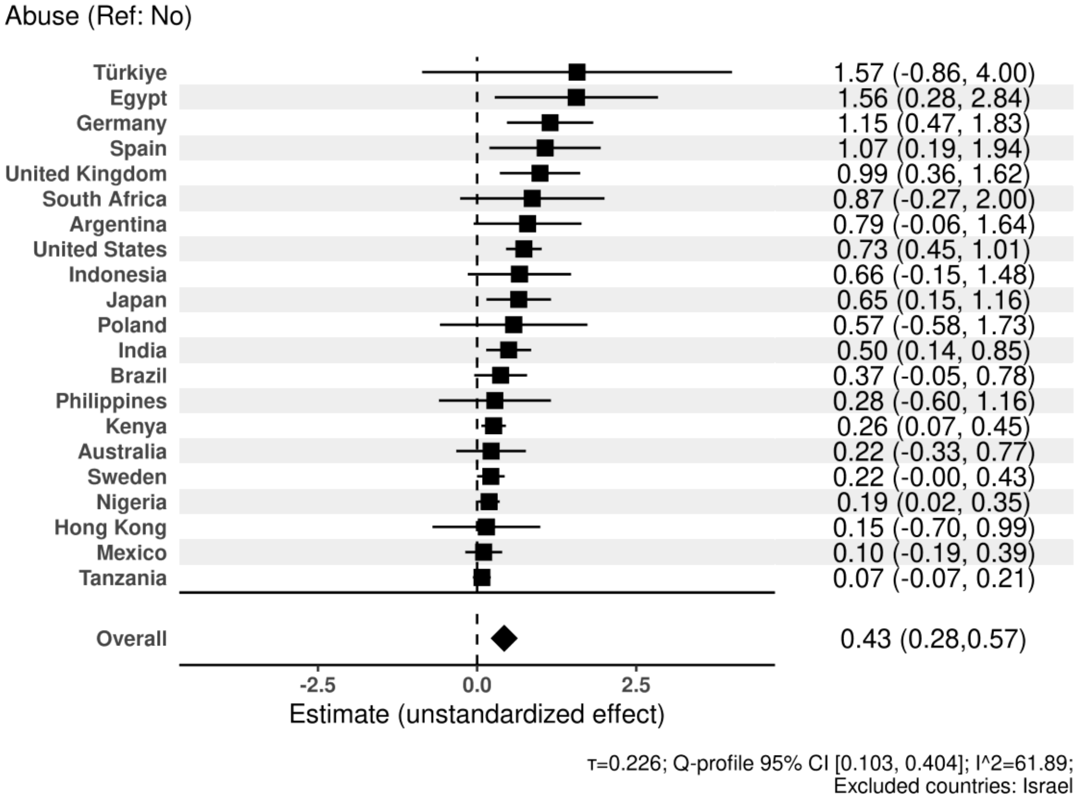

Figure S37. Forest plot for `Outsider growing up`-`Yes` effect

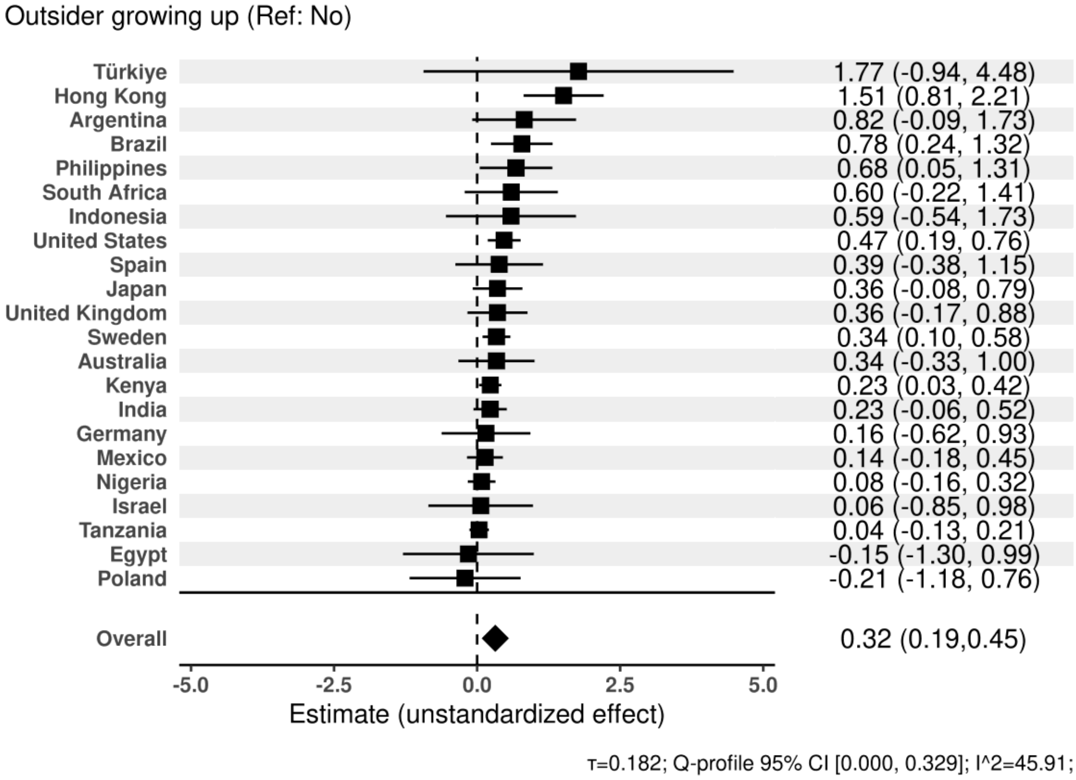

Figure S38. Forest plot for `Self-rated health growing up`-`Excellent` effect

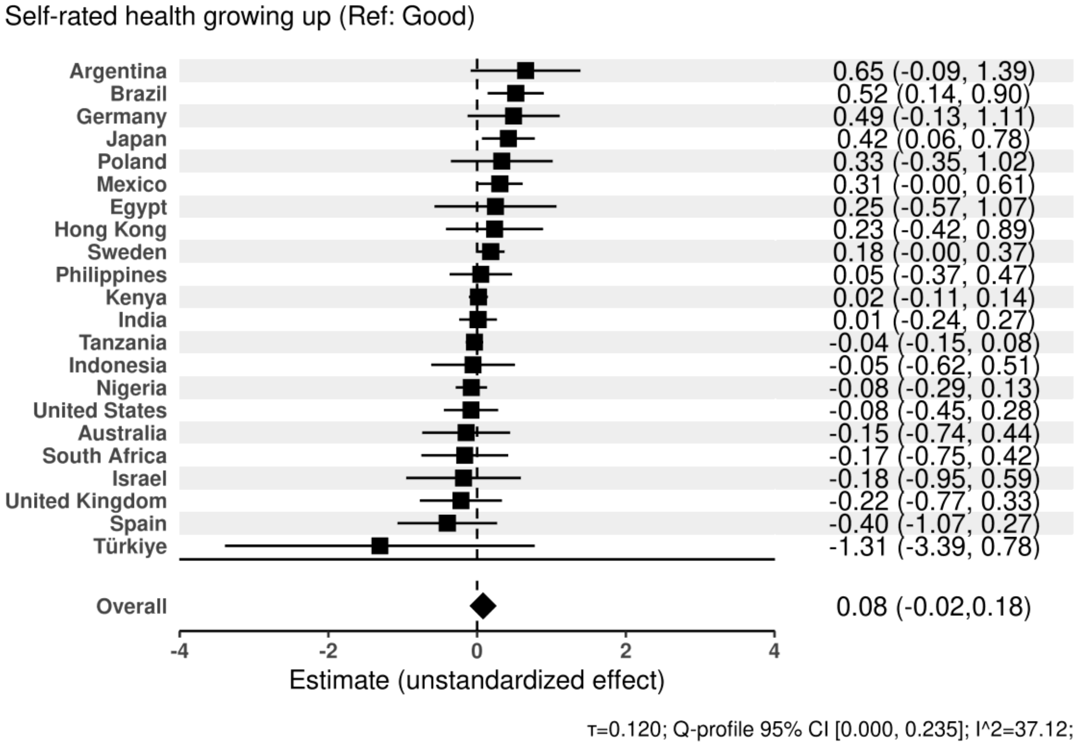

Figure S39. Forest plot for `Self-rated health growing up`-`Very good` effect

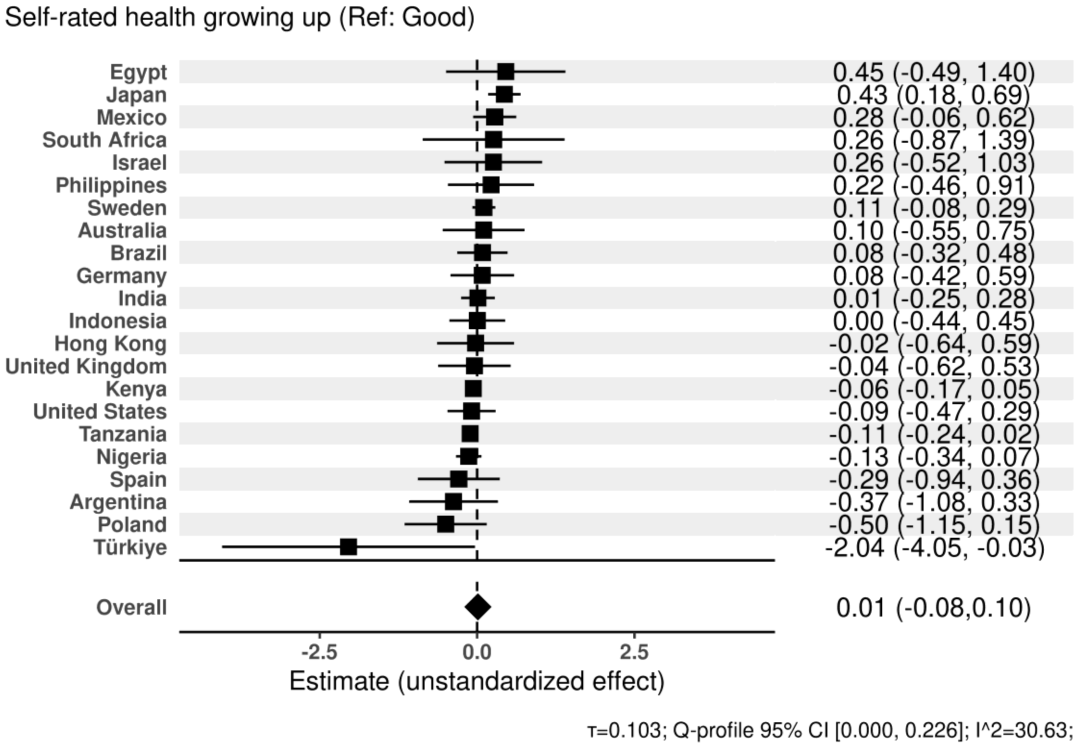

Figure S40. Forest plot for `Self-rated health growing up`-`Fair` effect

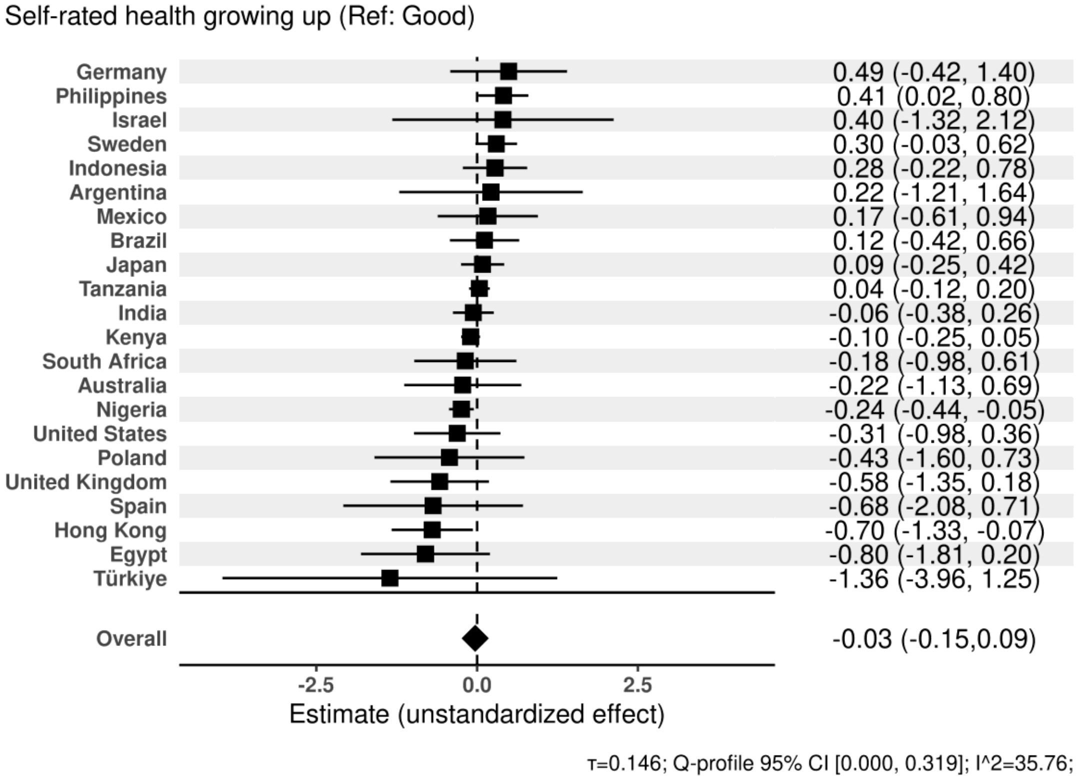

Figure S41. Forest plot for `Self-rated health growing up`-`Poor` effect

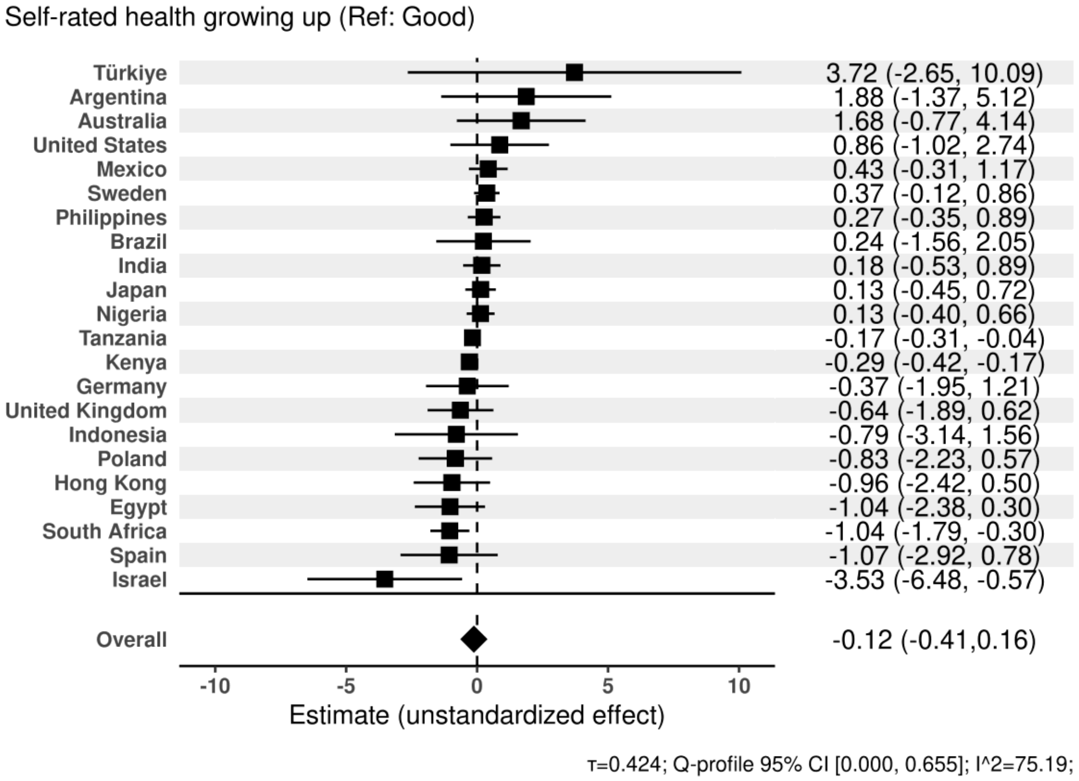

Figure S42. Forest plot for `Immigration status`-`Born in another country` effect

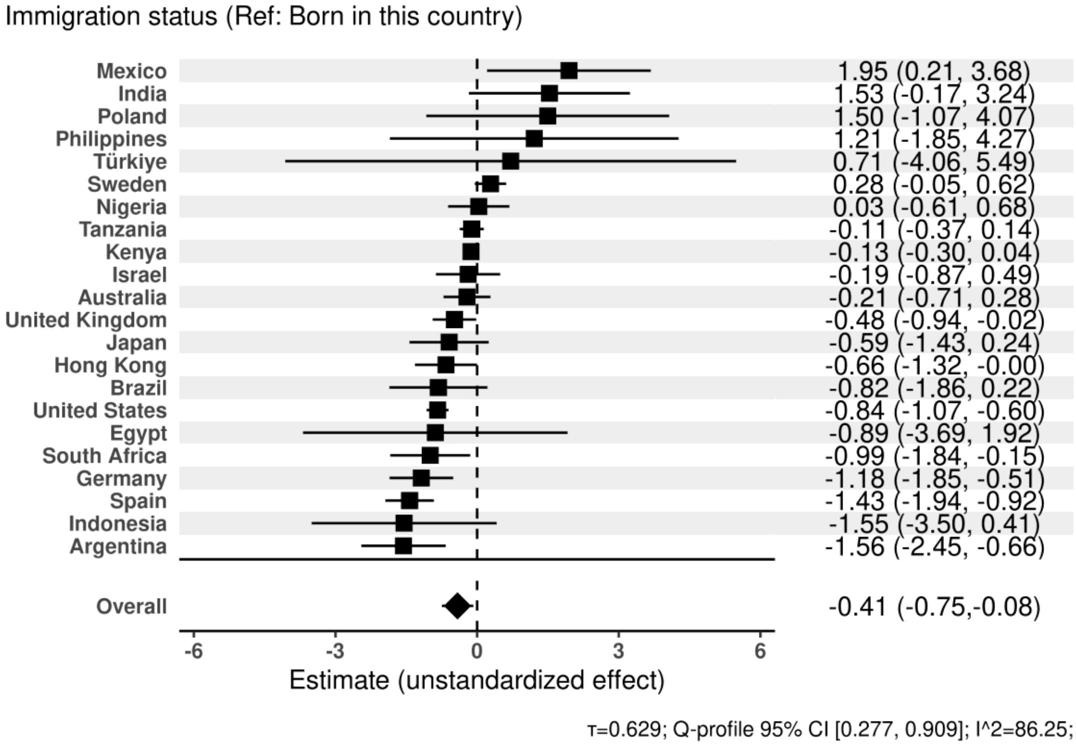

Figure S43. Forest plot for `Age 12 religious service attendance`-`At least 1/week` effect

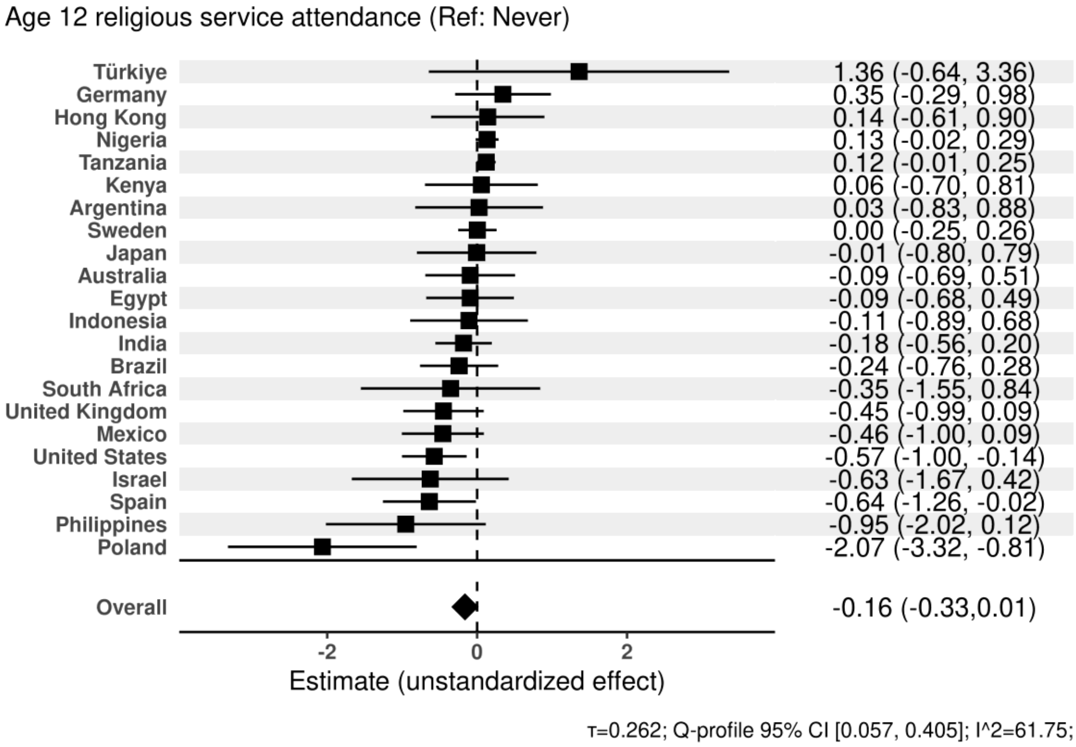

Figure S44. Forest plot for `Age 12 religious service attendance`-`1-3/month` effect

Age 12 religious service attendance (Ref: Never)

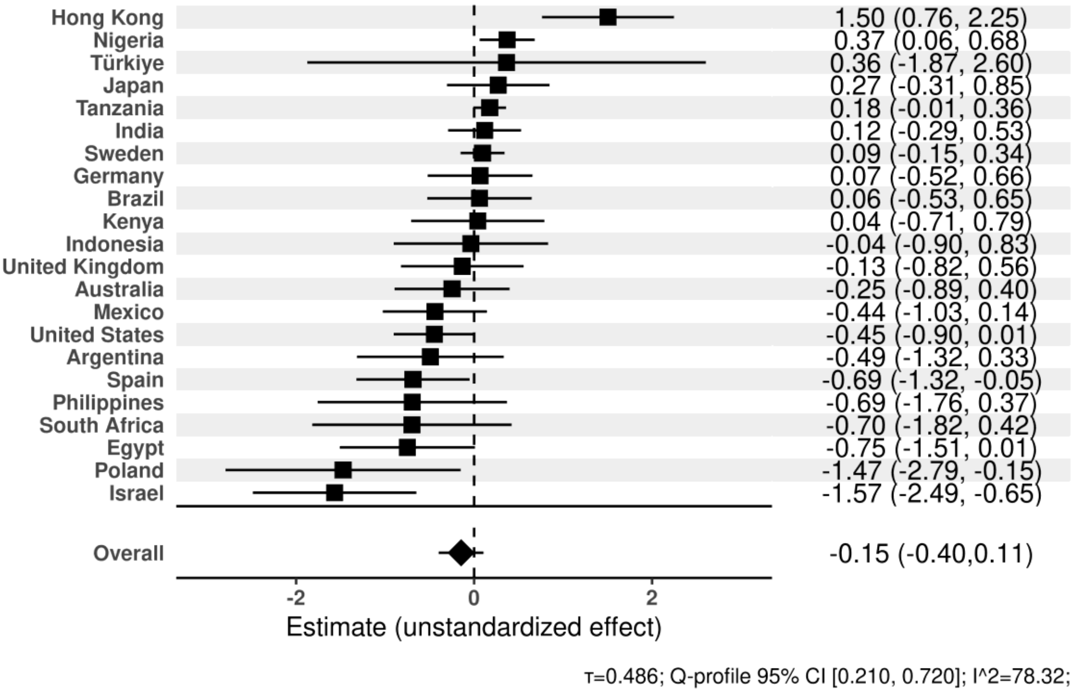

Figure S45. Forest plot for `Age 12 religious service attendance`-`< 1/month` effect

Age 12 religious service attendance (Ref: Never)

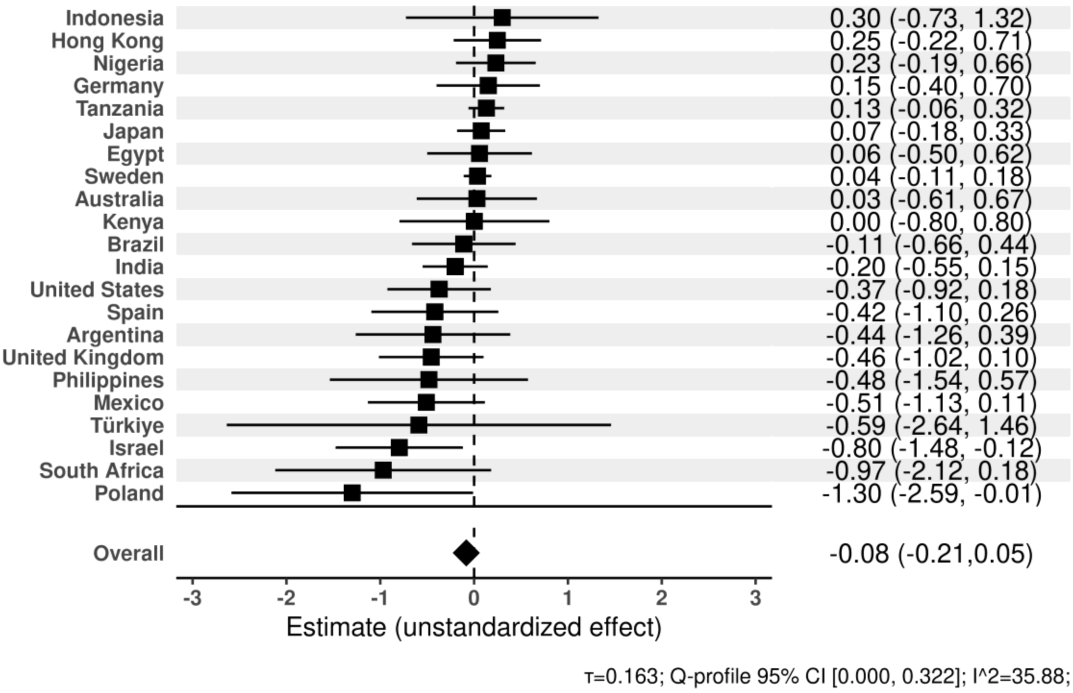

Figure S46.

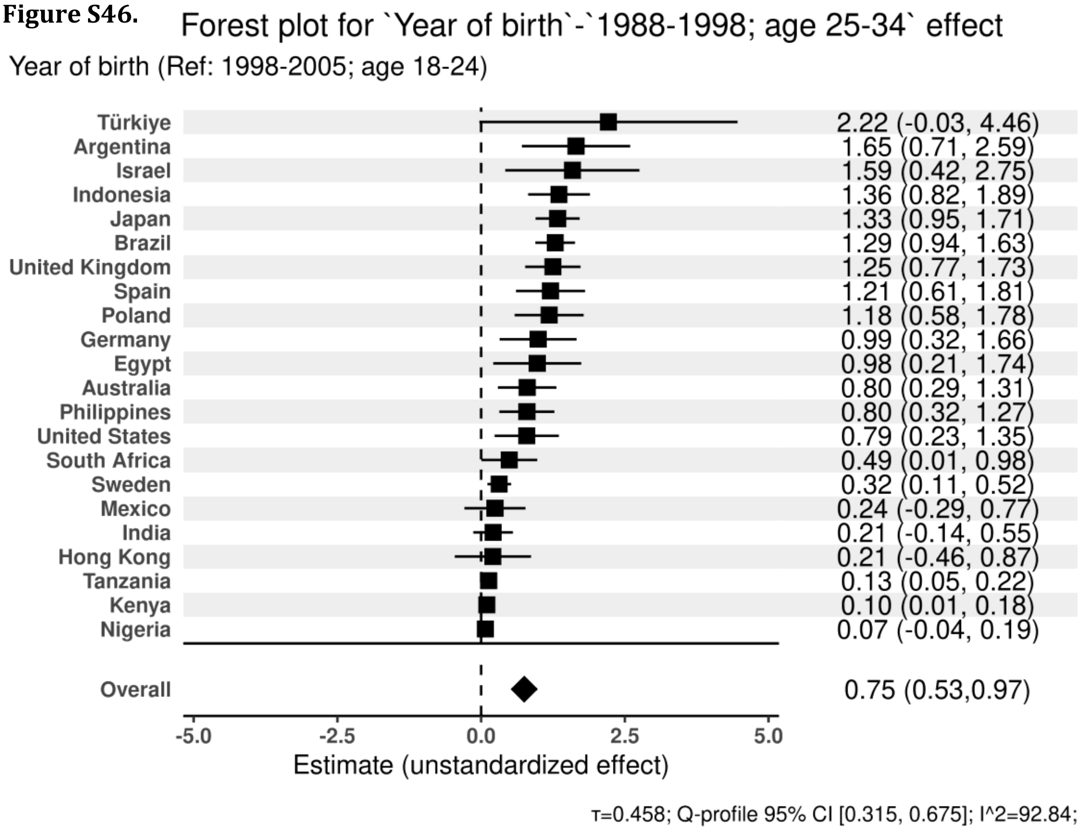

Figure S47.

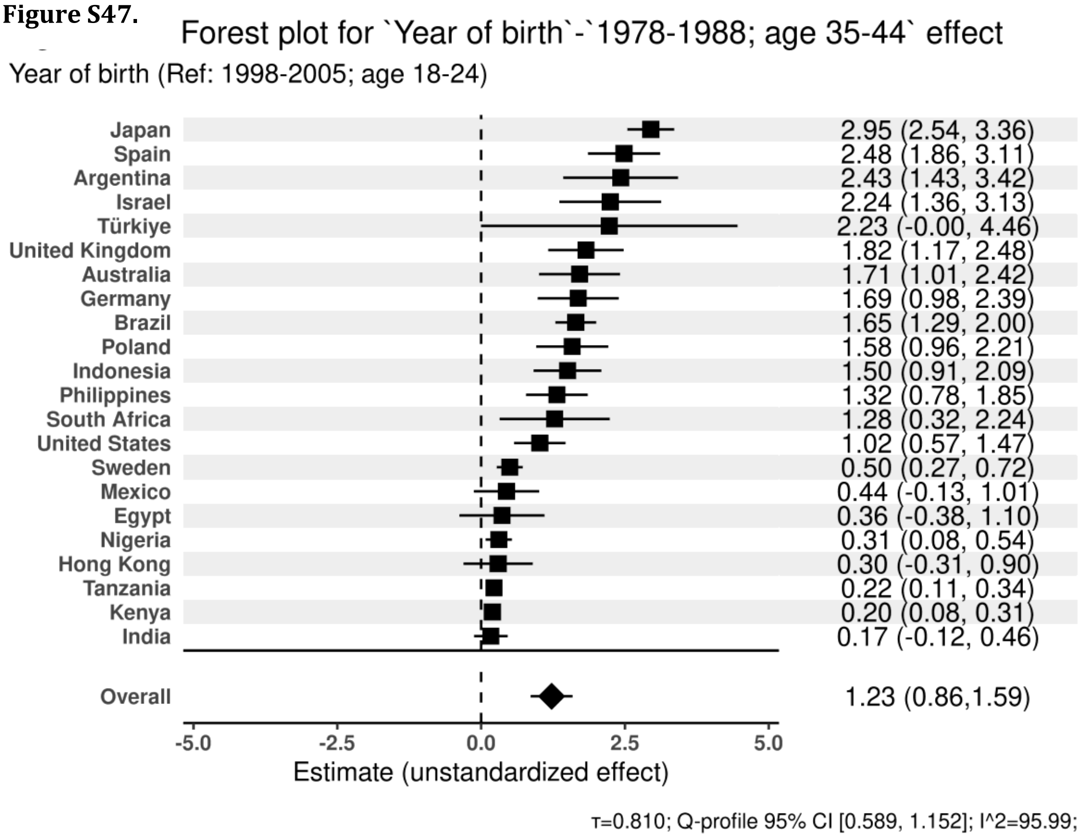

Figure S48.

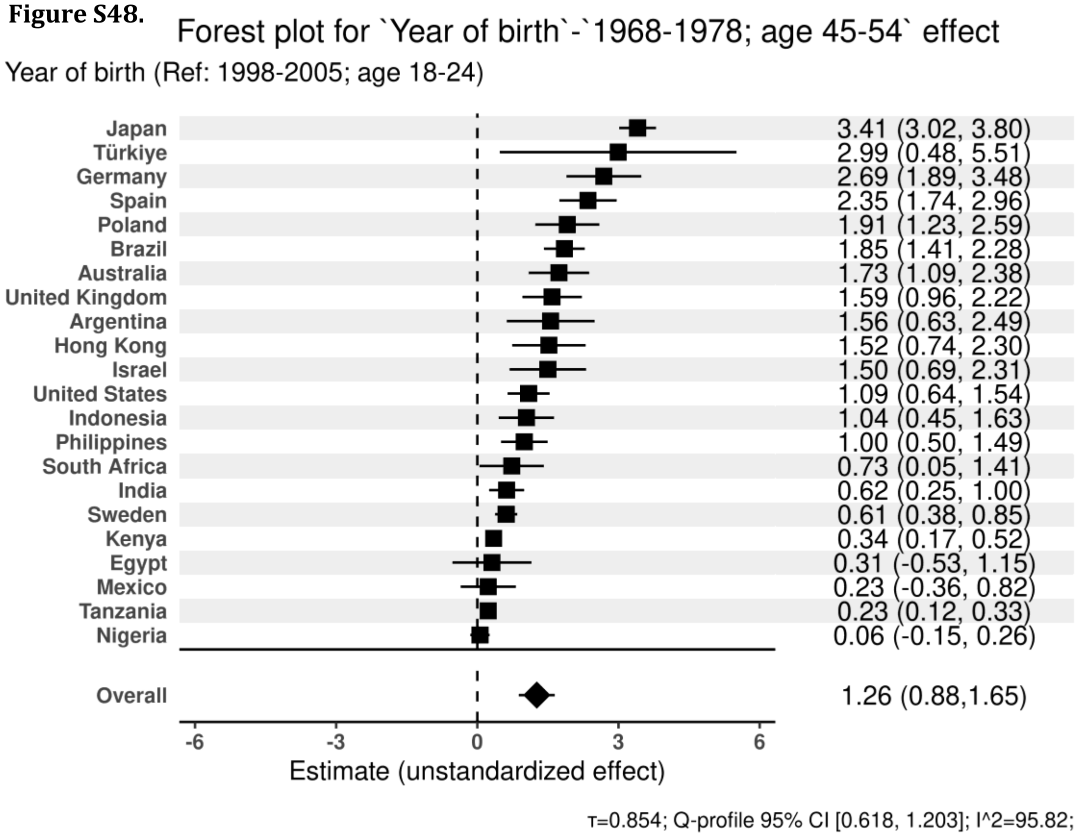

Figure S49.

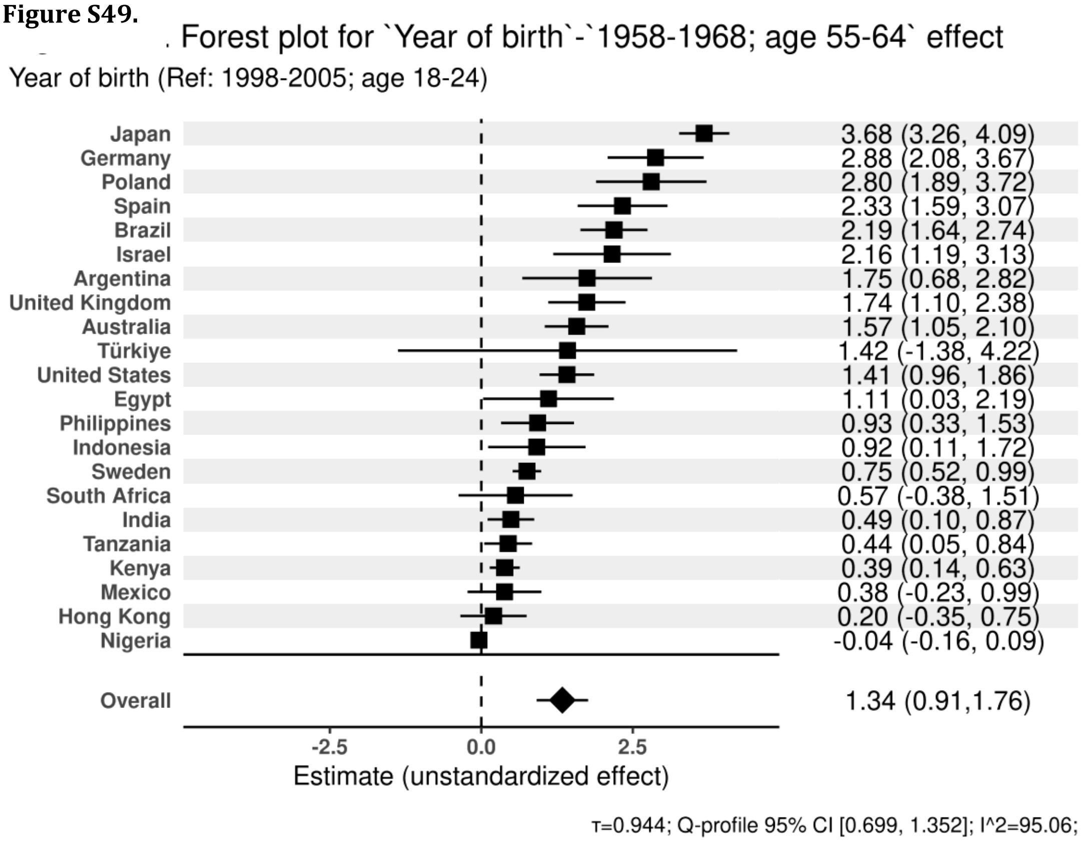

Figure S50.

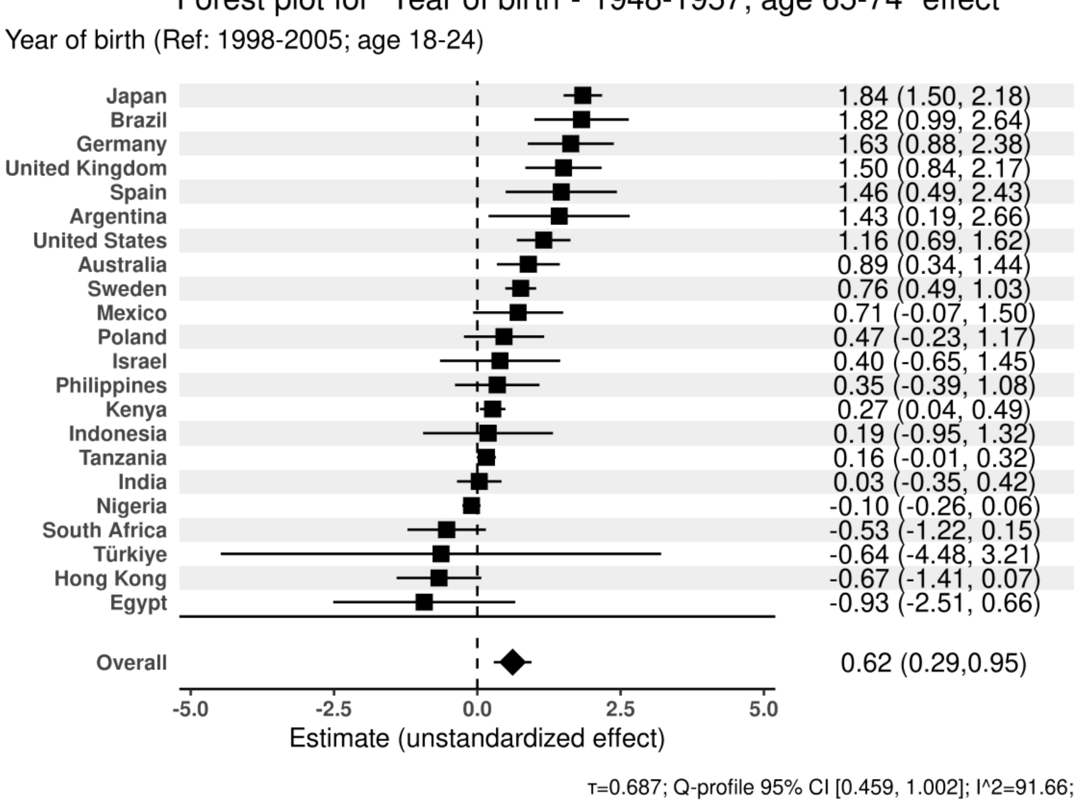

Figure S51.

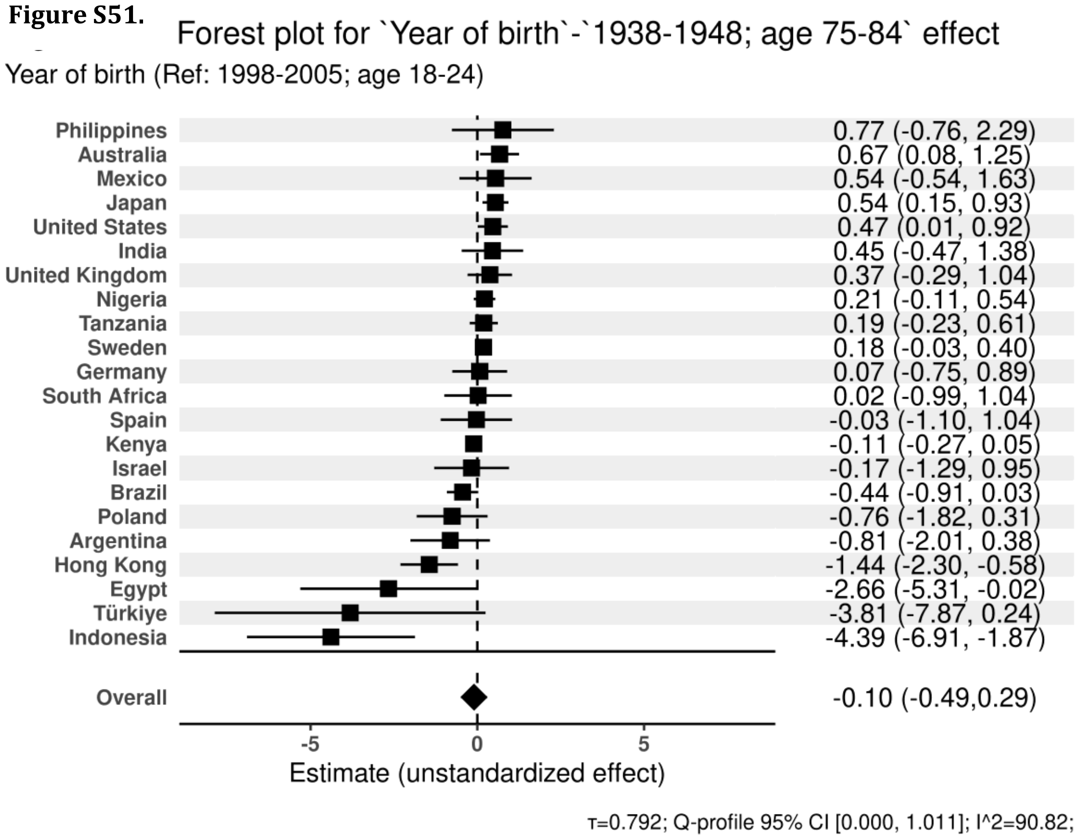

Figure S52. Forest plot for `Year of birth`-`1938 or earlier; 85 or older` effect

Year of birth (Ref: 1998-2005; age 18-24)

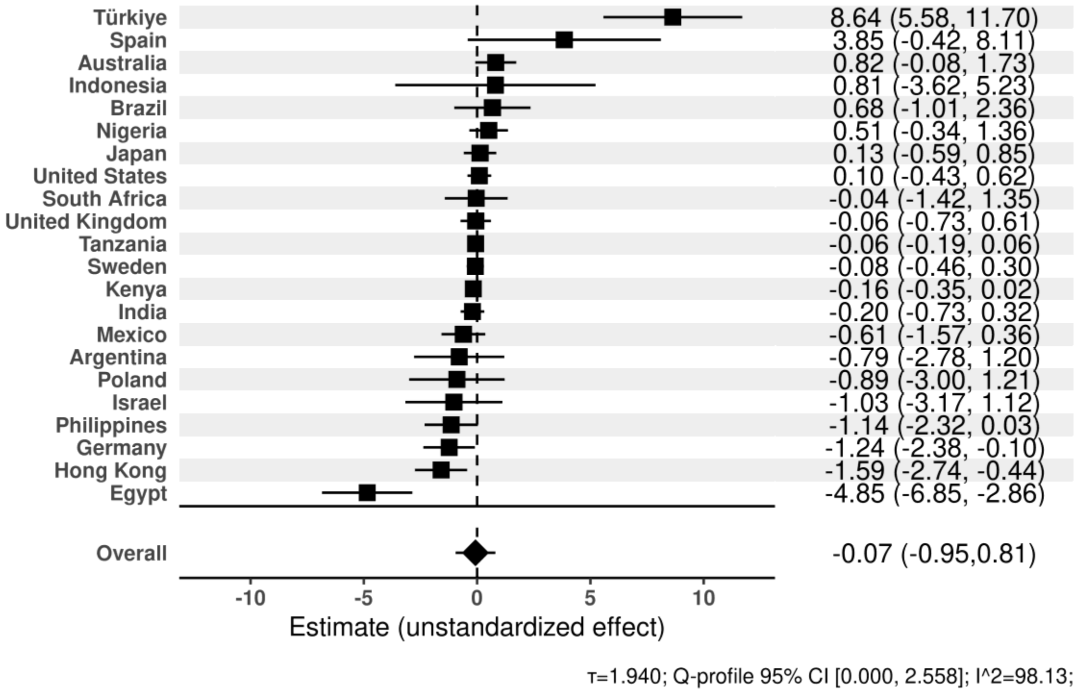

Figure S53. Forest plot for `Gender`-`Female` effect

Gender (Ref: Male)

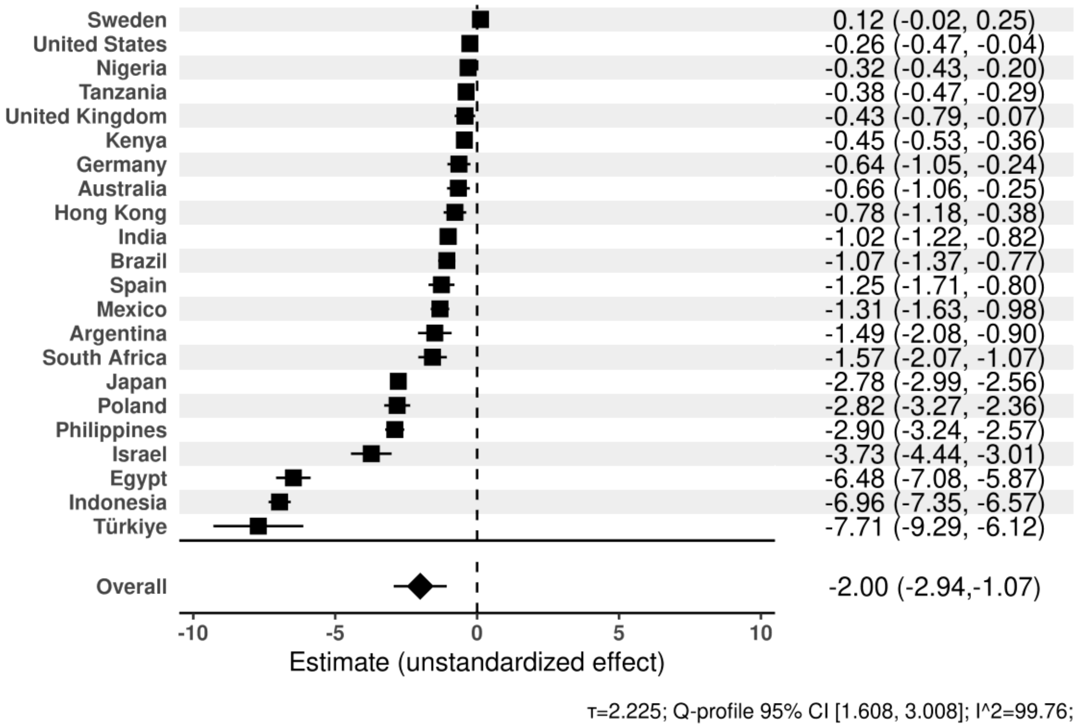

Figure S54.

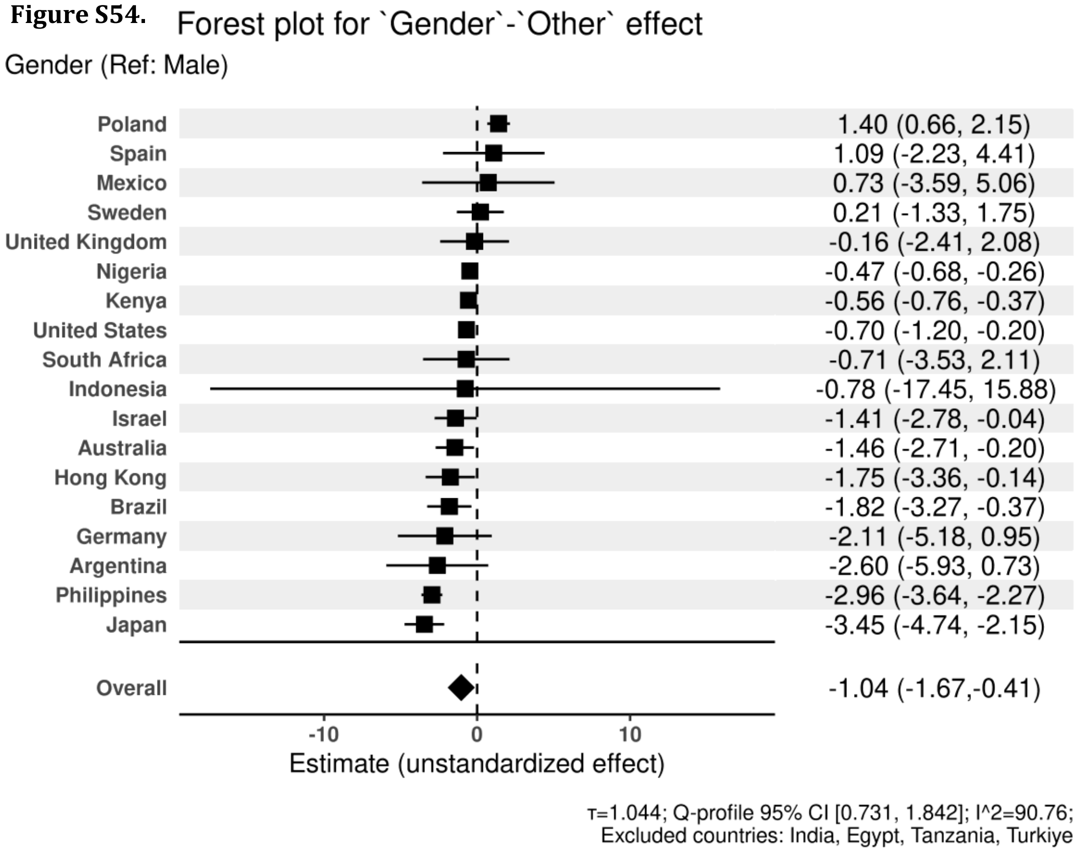

Forest plot for `Gender`-`Other` effect
Gender (Ref: Male)
Poland 1.40 (0.66, 2.15)
Spain 1.09 (-2.23, 4.41)
Mexico 0.73 (-3.59, 5.06)
Sweden 0.21 (-1.33, 1.75)
United Kingdom -0.16 (-2.41, 2.08)
Nigeria -0.47 (-0.68, -0.26)
Kenya -0.56 (-0.76, -0.37)
United States -0.70 (-1.20, -0.20)
South Africa -0.71 (-3.53, 2.11)
Indonesia -0.78 (-17.45, 15.88)
Israel -1.41 (-2.78, -0.04)
Australia -1.46 (-2.71, -0.20)
Hong Kong -1.75 (-3.36, -0.14)
Brazil -1.82 (-3.27, -0.37)
Germany -2.11 (-5.18, 0.95)
Argentina -2.60 (-5.93, 0.73)
Philippines -2.96 (-3.64, -2.27)
Japan -3.45 (-4.74, -2.15)
Overall -1.04 (-1.67,-0.41)
-10
0
10
Estimate (unstandardized effect)
τ=1.044; Q-profile 95% CI [0.731, 1.842]; I^2=90.76;
Excluded countries: India, Egypt, Tanzania, Turkiye

Figure S55. Forest plot for `Relationship with mother`-`Very good/somewhat good` effect

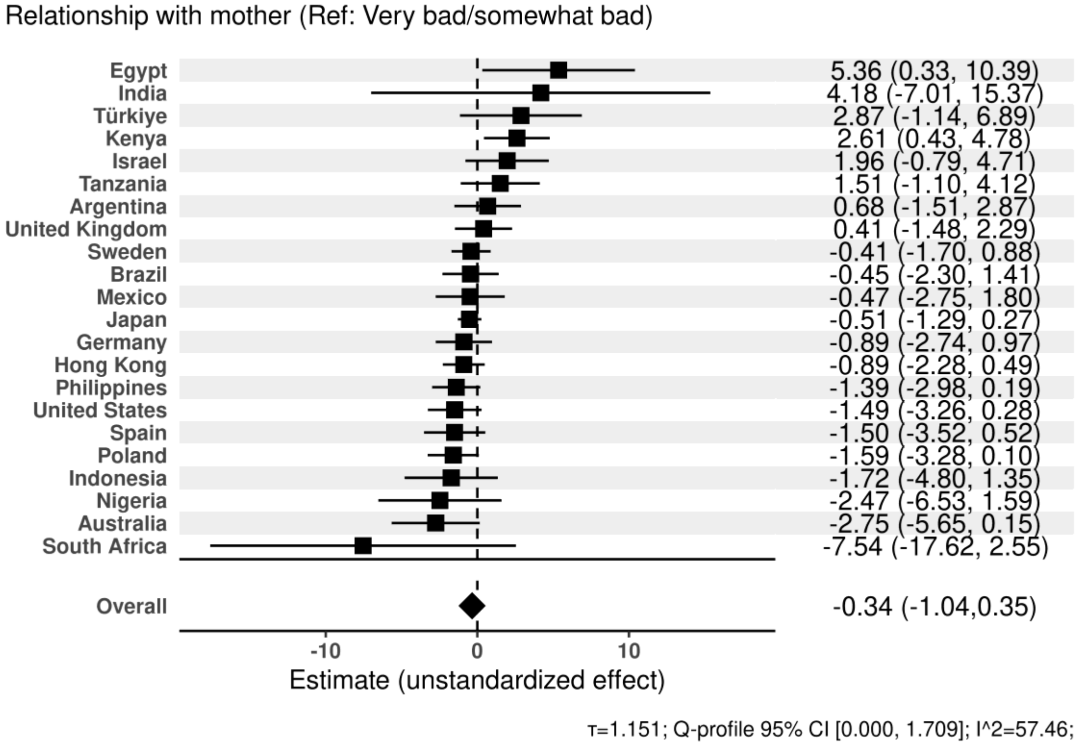

Figure S56. Forest plot for `Relationship with father`-`Very good/somewhat good` effect

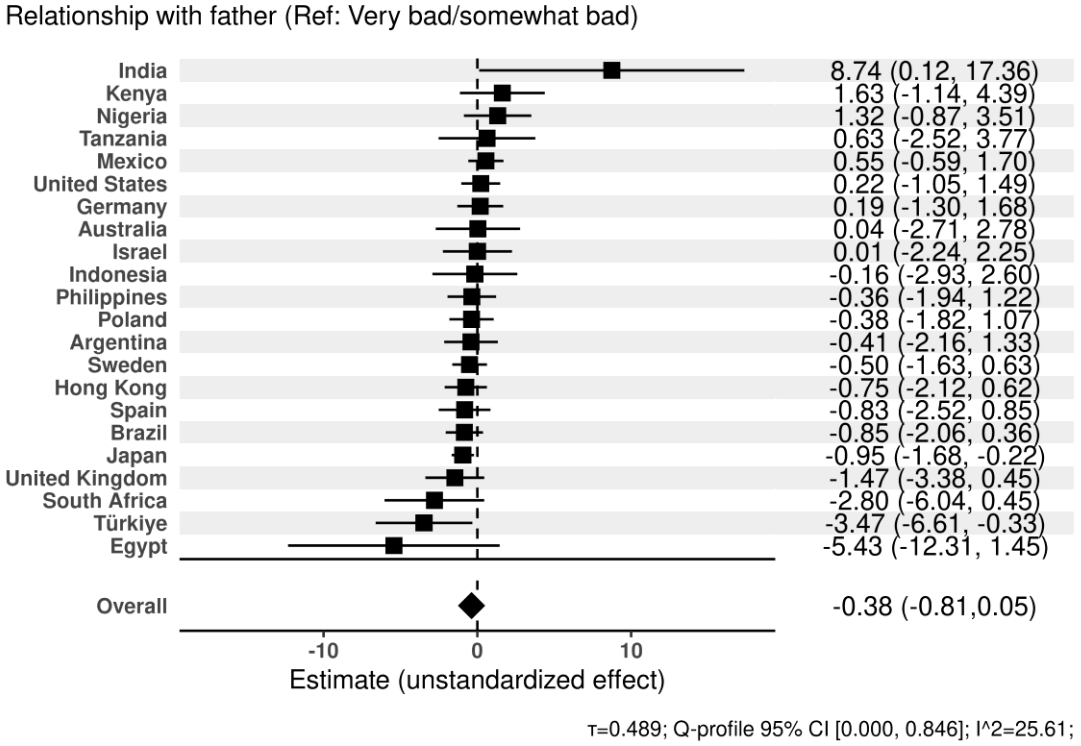

Figure S57.

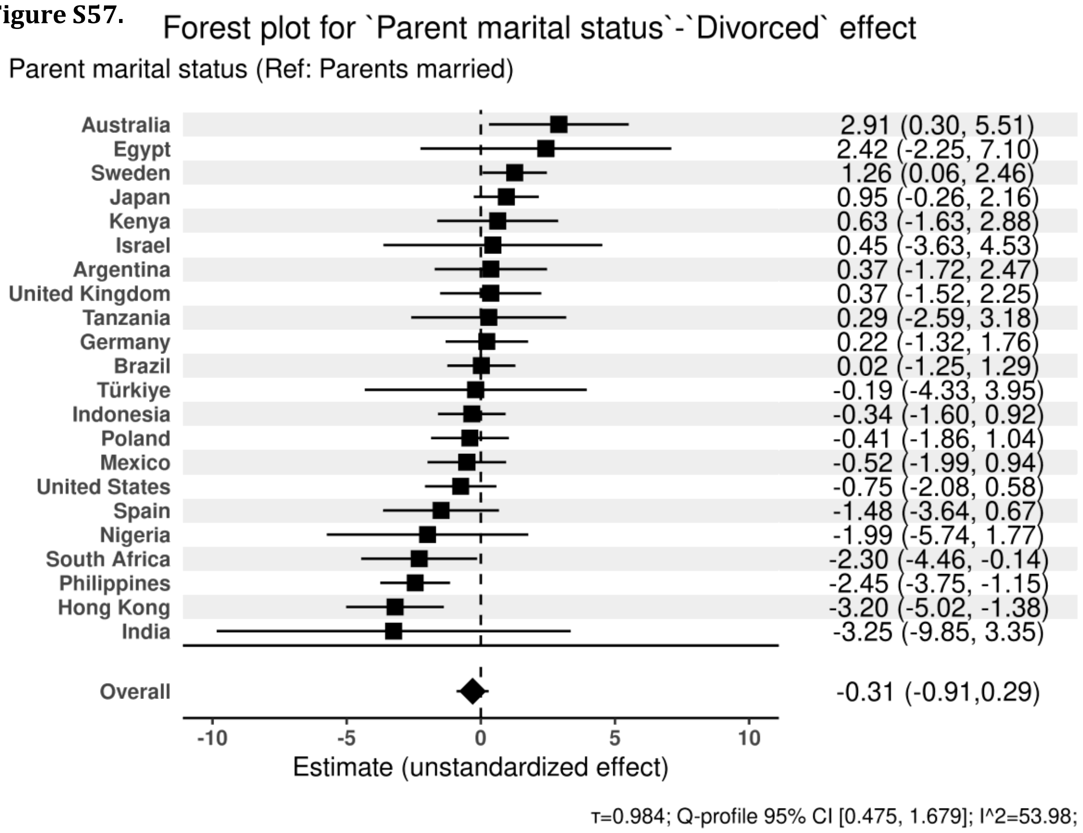

Figure S58.

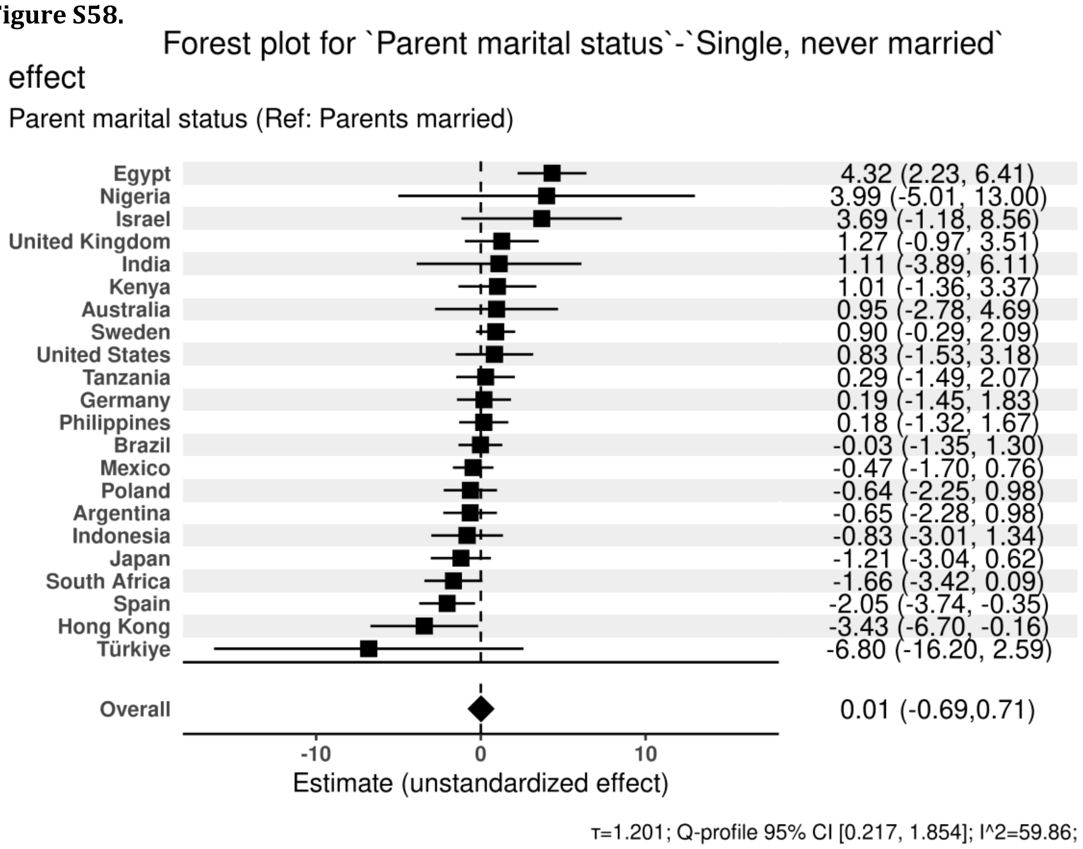

Figure S59. Forest plot for `Parent marital status`-`One or both parents had died` effect

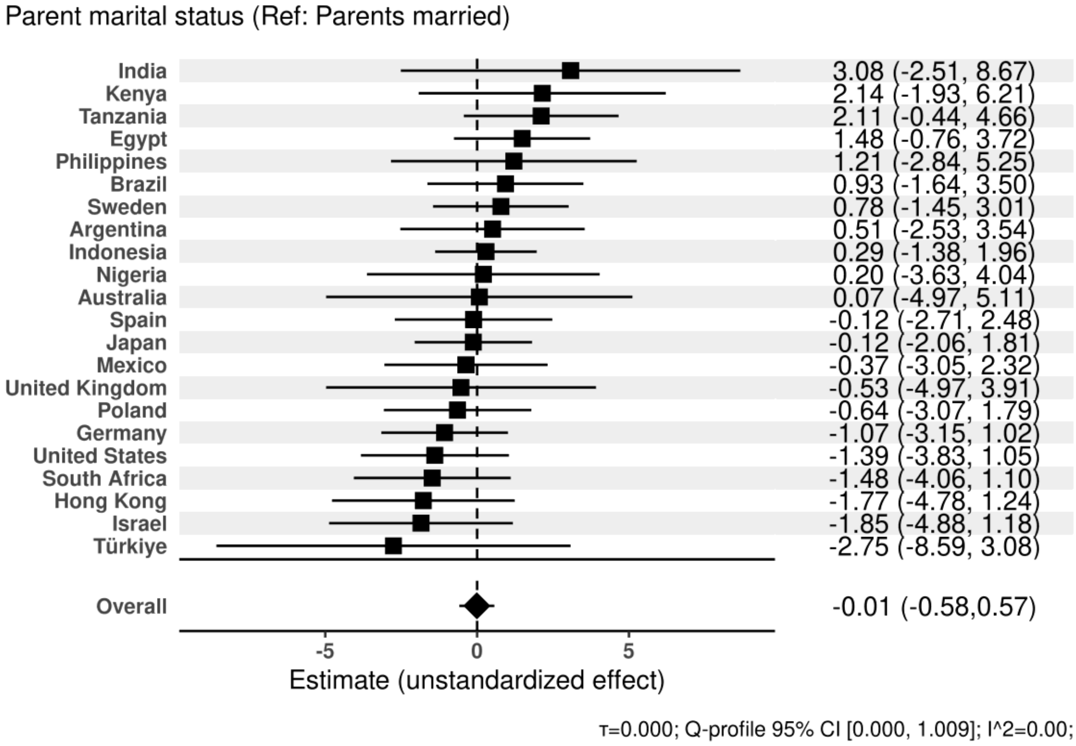

Figure S60. Forest plot for `Subjective financial status of family growing up`-`Lived comfortably` effect

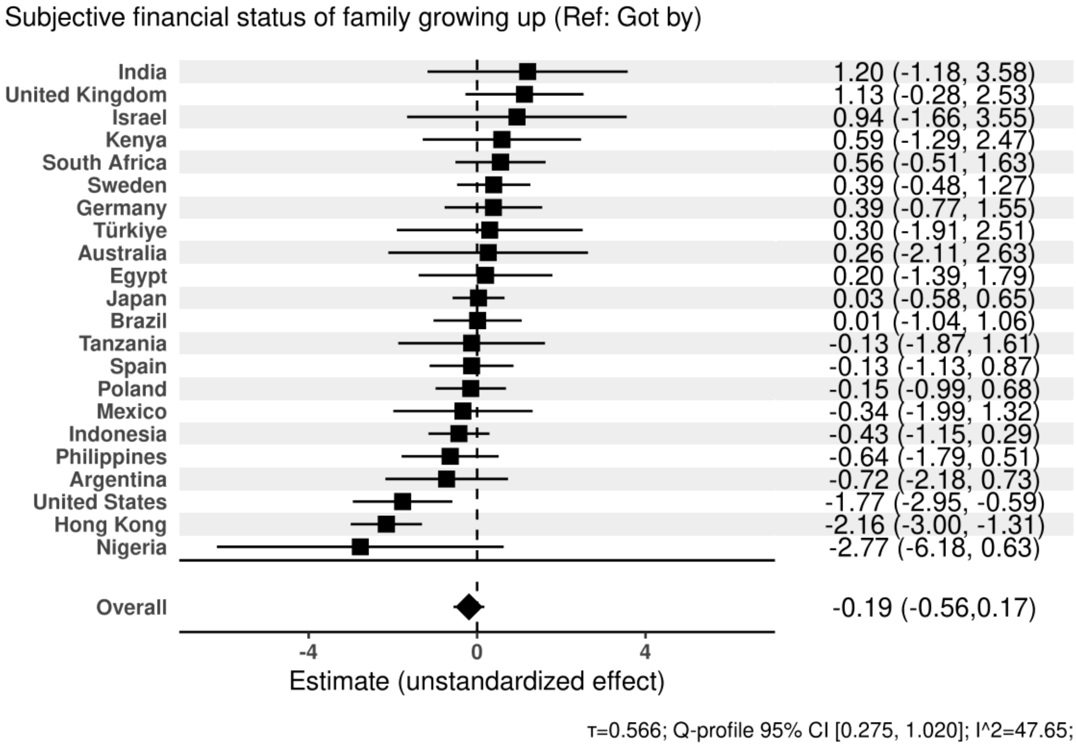

Figure S61. Forest plot for `Subjective financial status of family growing up`-`Found it difficult` effect

Subjective financial status of family growing up (Ref: Got by)

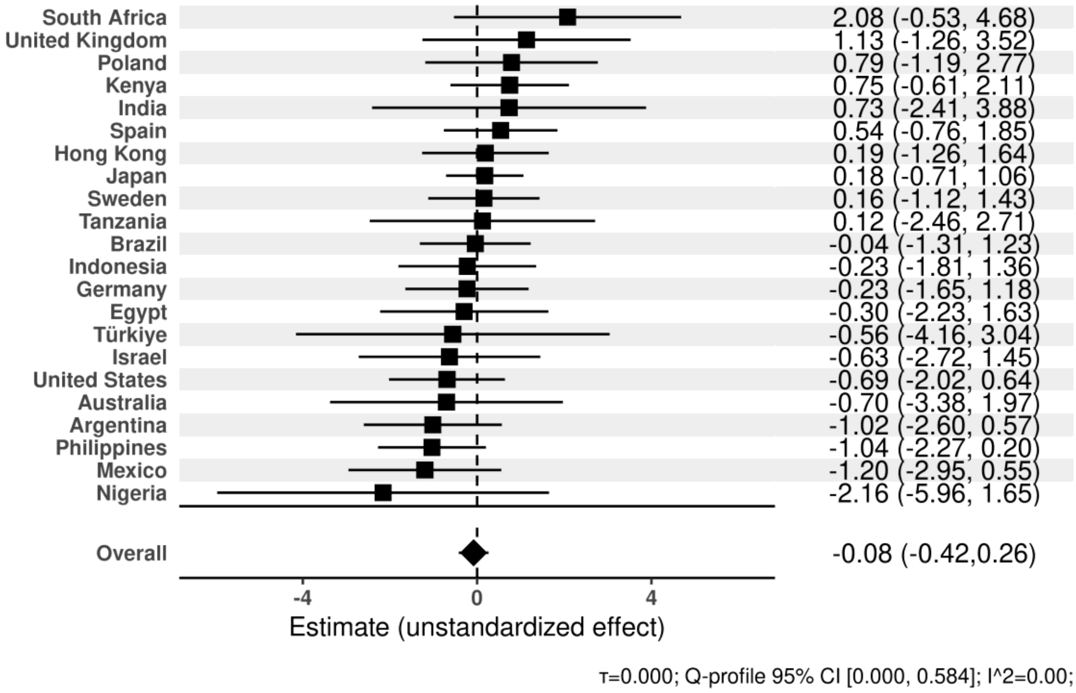

Figure S62. Forest plot for `Subjective financial status of family growing up`-`Found it very difficult` effect

Subjective financial status of family growing up (Ref: Got by)

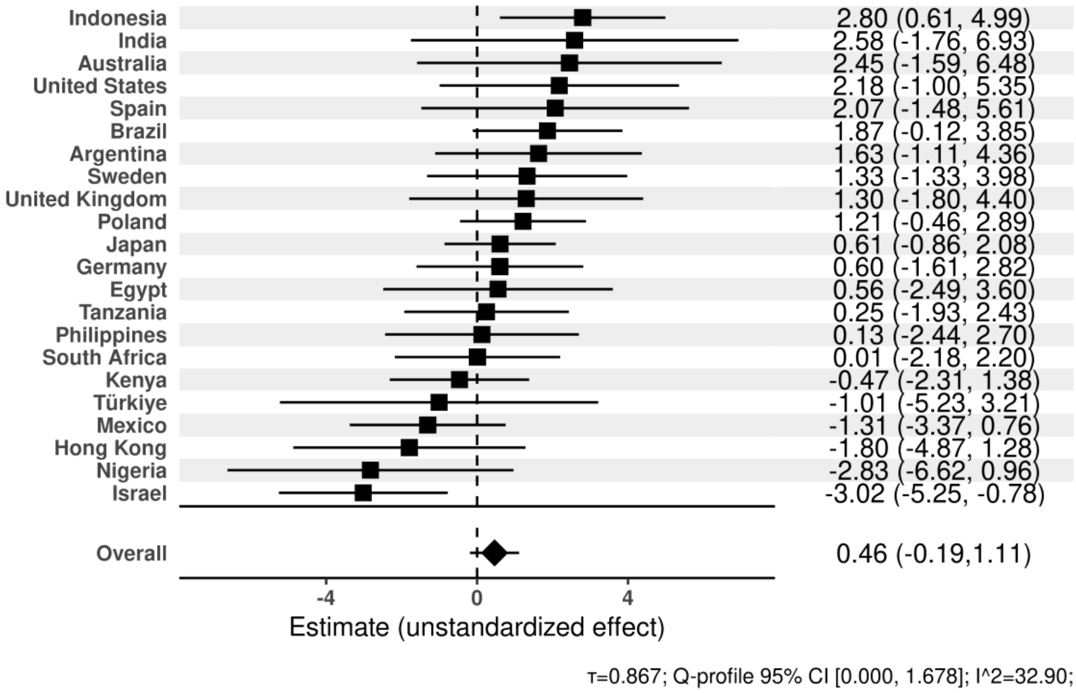

Figure S63.

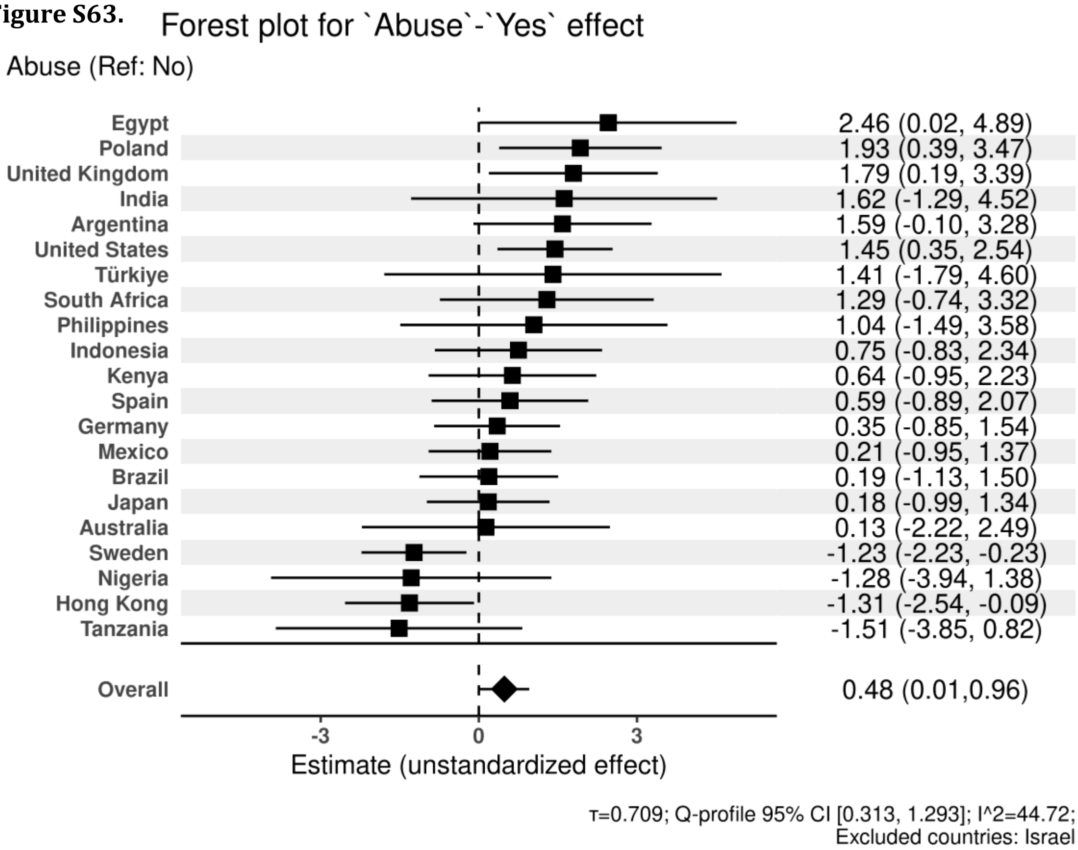

Figure S64.

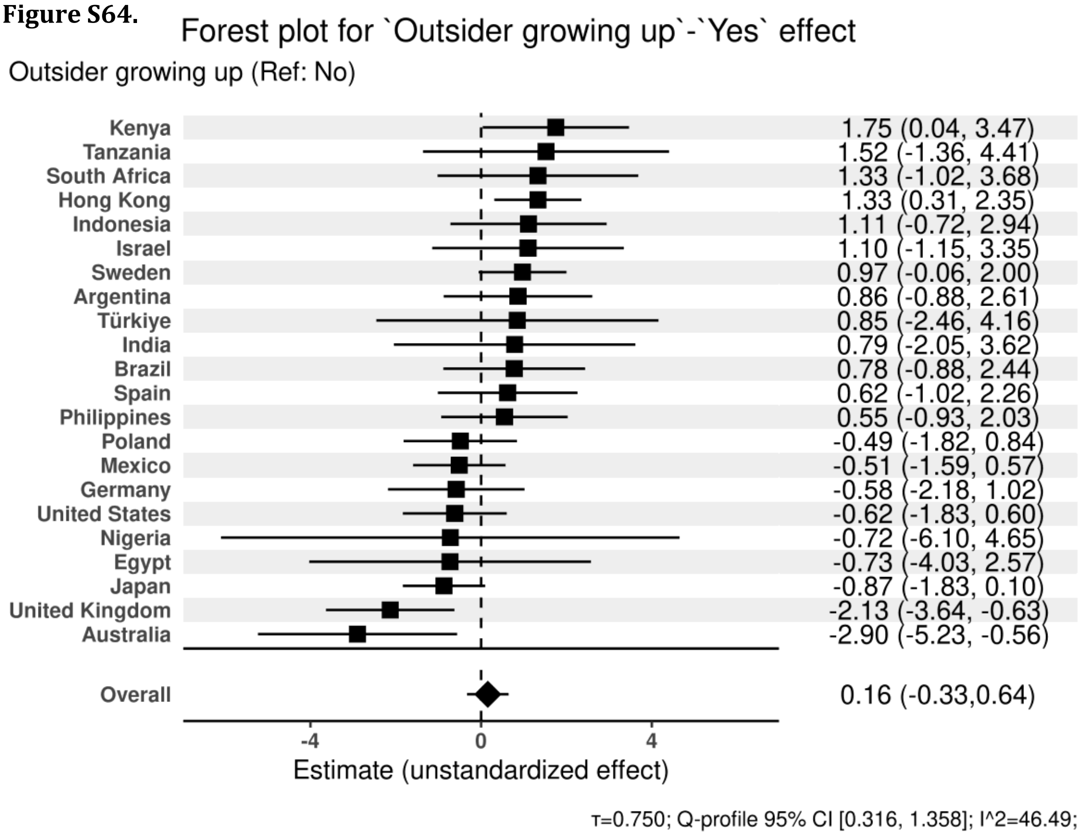

Figure S65.

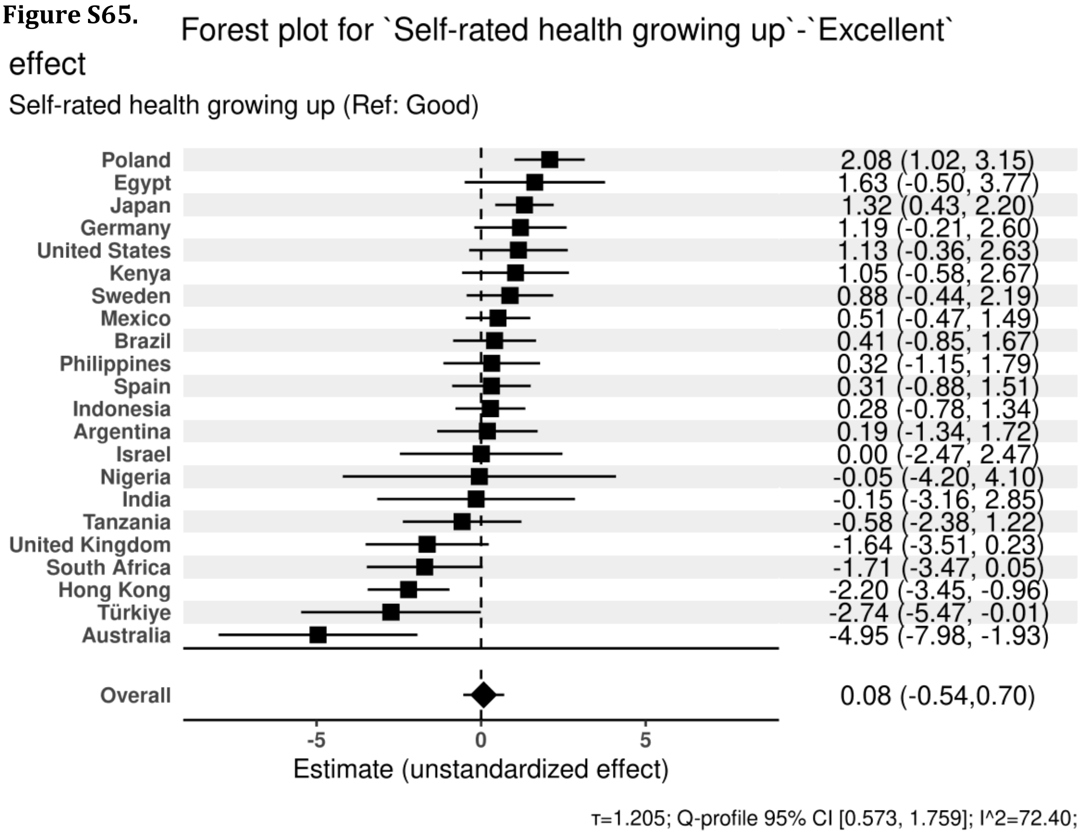

Figure S66.

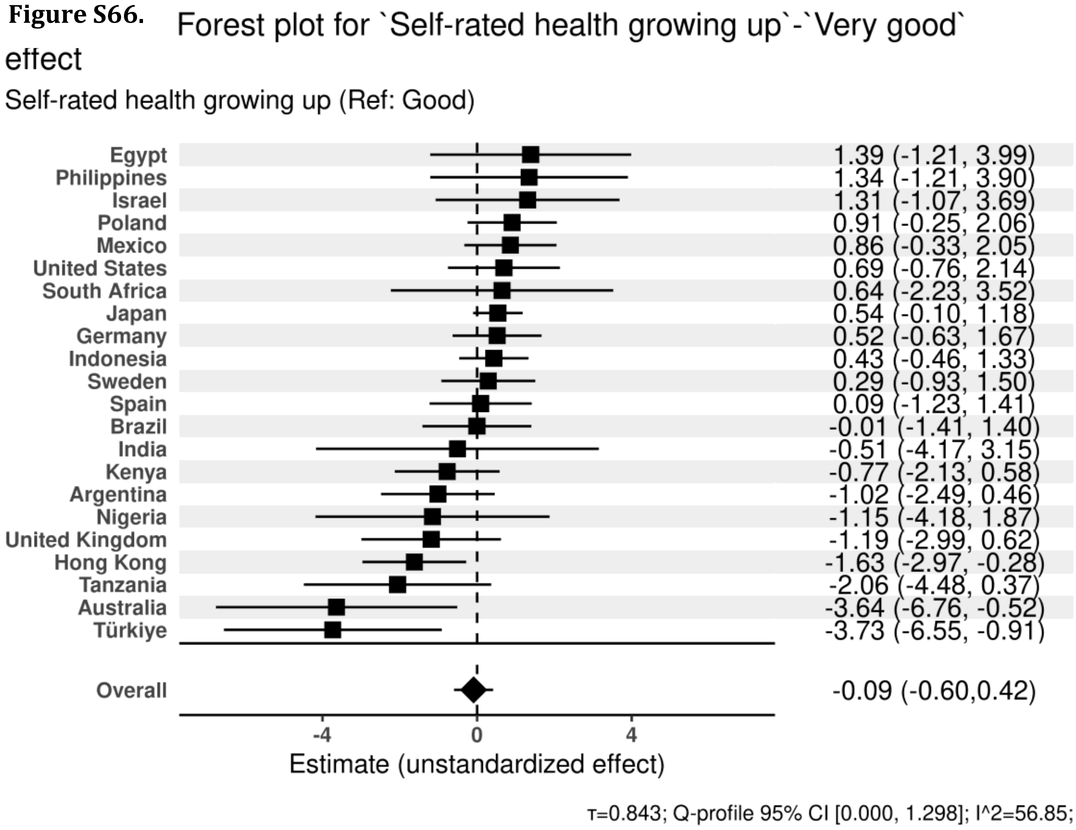

Figure S67.

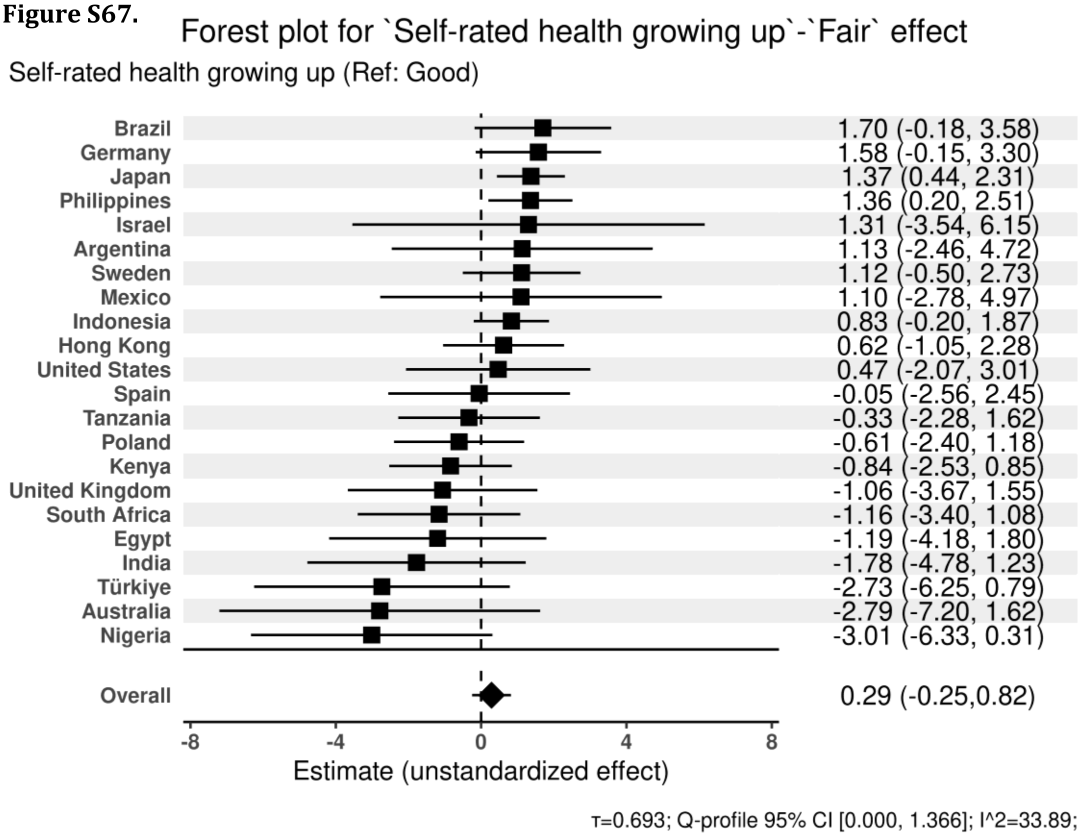

Figure S68.

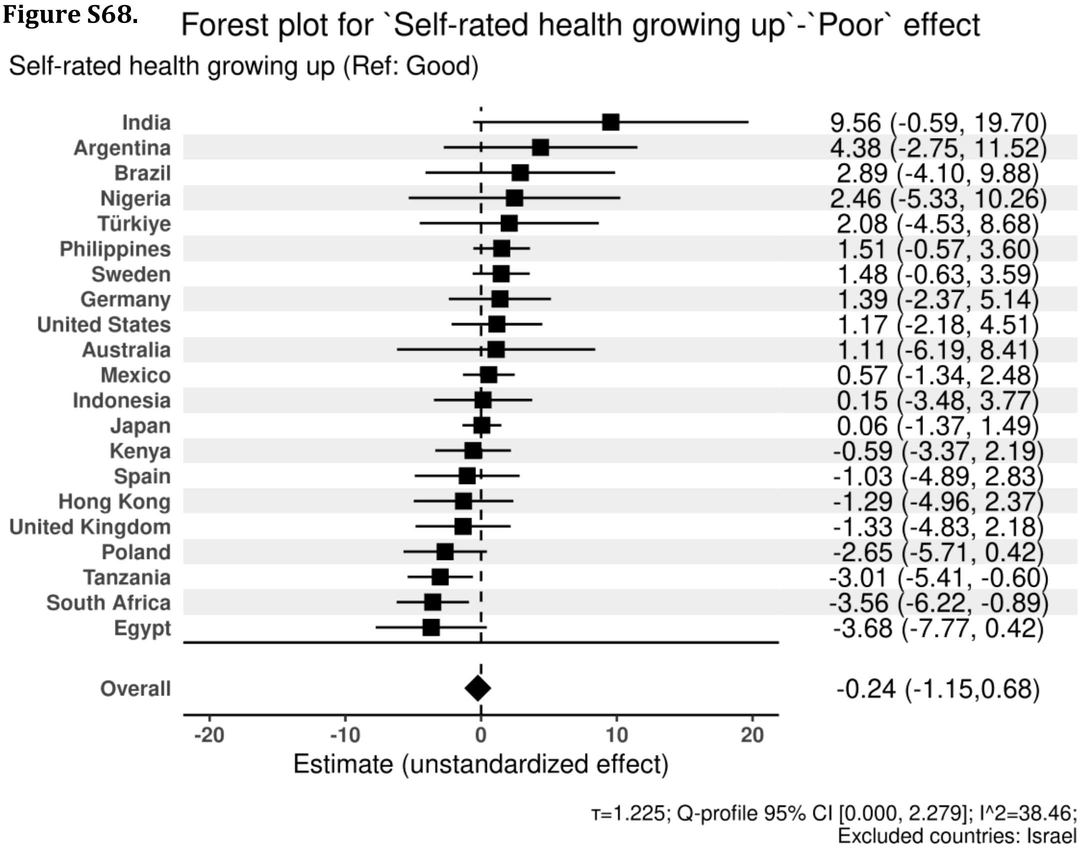

Figure S69. Forest plot for `Immigration status`-`Born in another country` effect

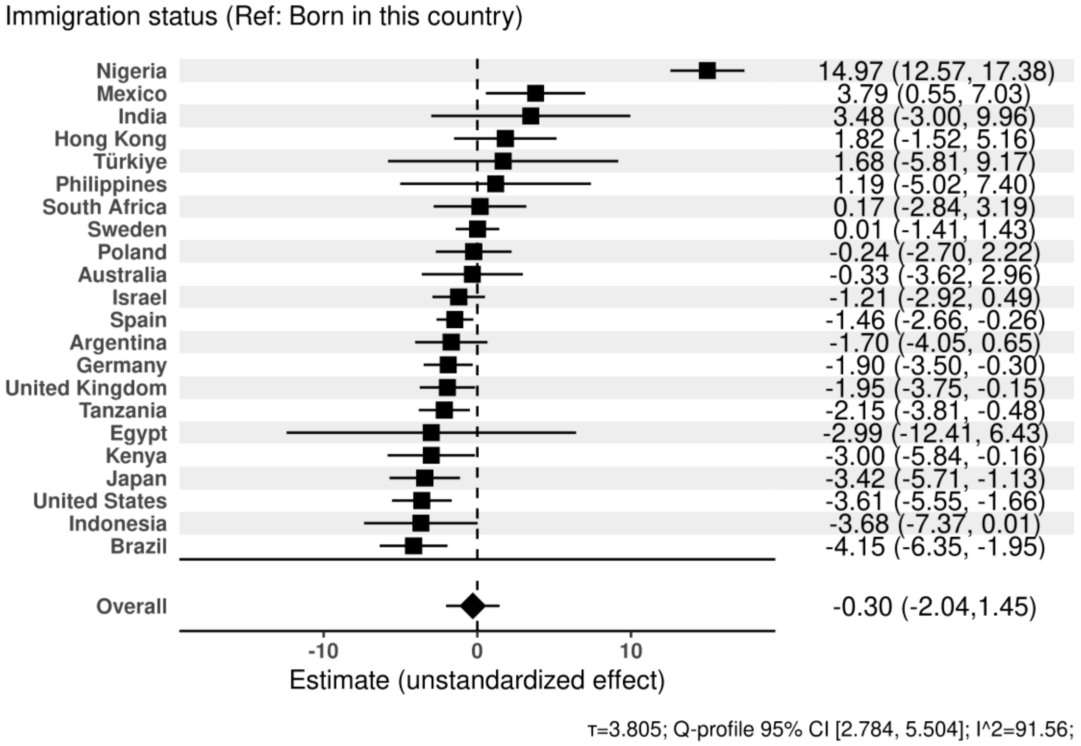

Figure S70. Forest plot for `Age 12 religious service attendance`-`At least 1/week` effect

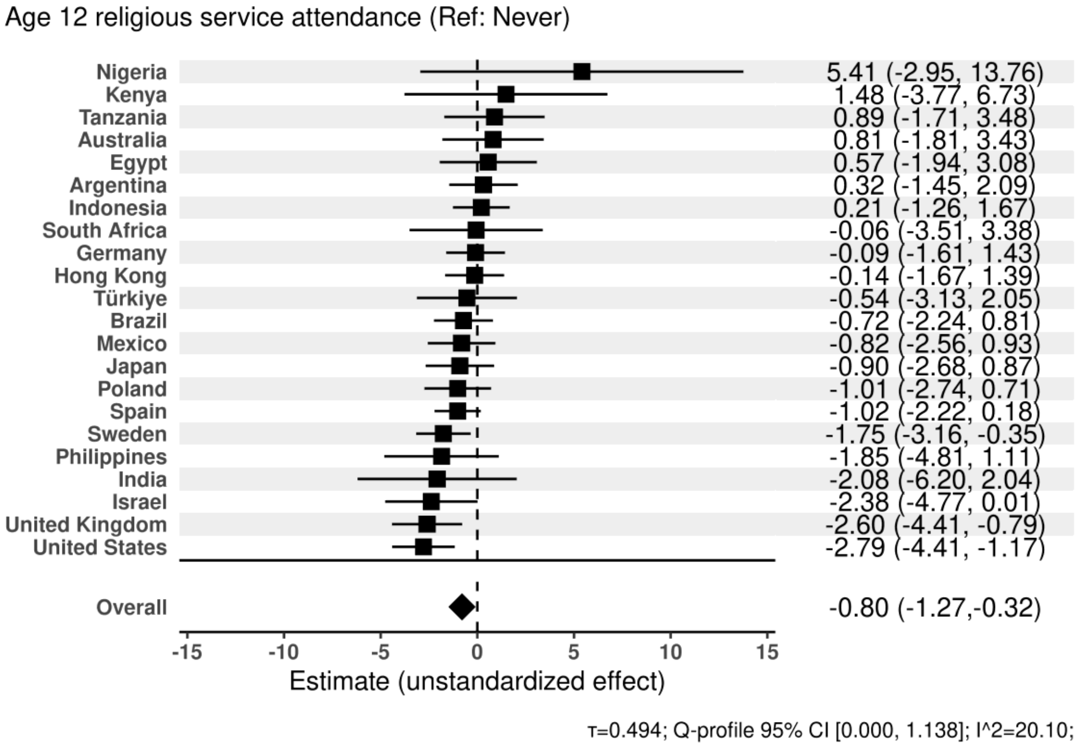

Figure S71. Forest plot for `Age 12 religious service attendance`-`1-3/month` effect

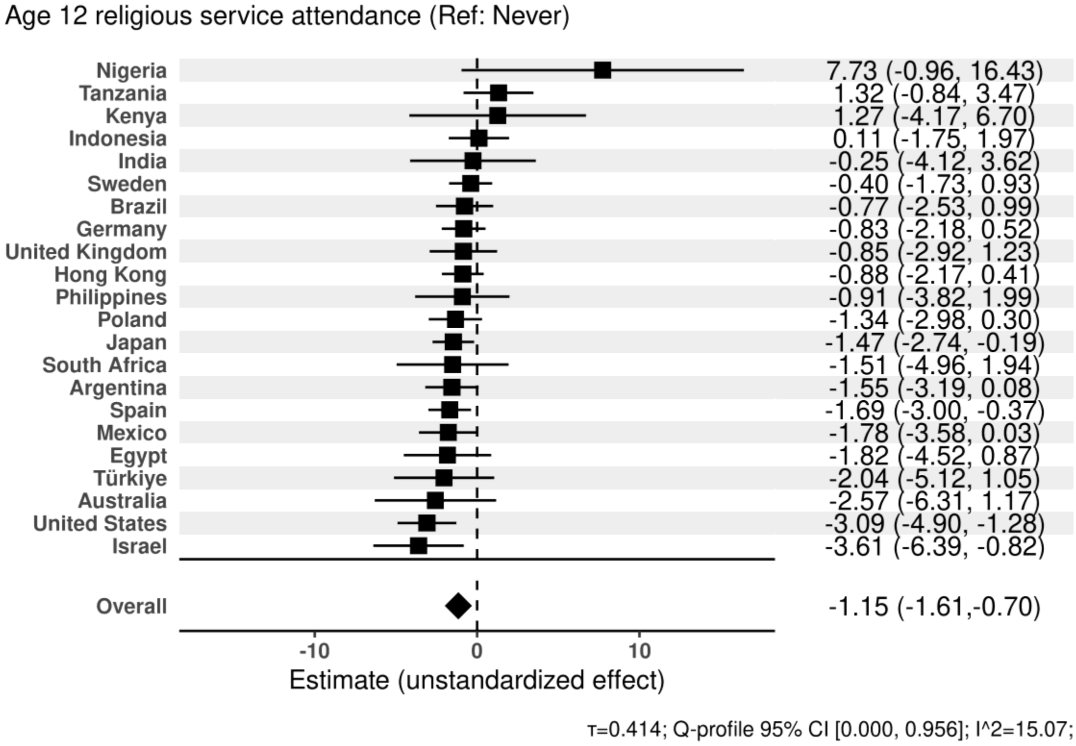

Figure S72. Forest plot for `Age 12 religious service attendance`-`< 1/month` effect

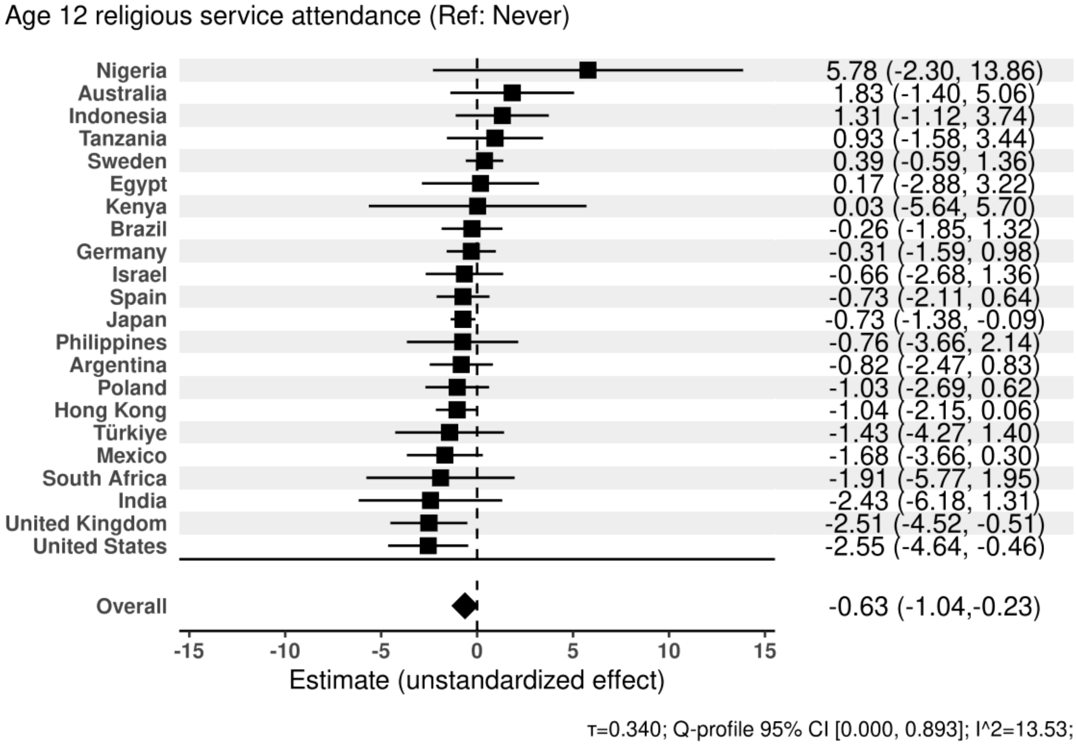

Figure S73.

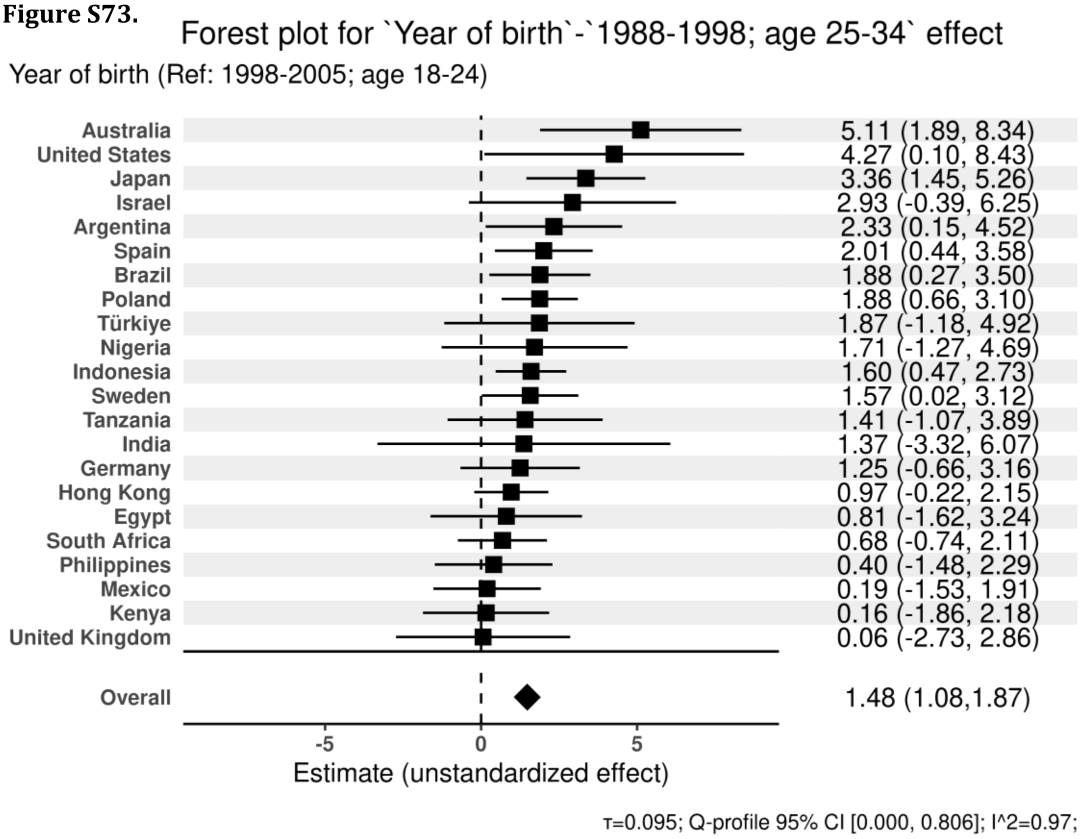

Figure S74.

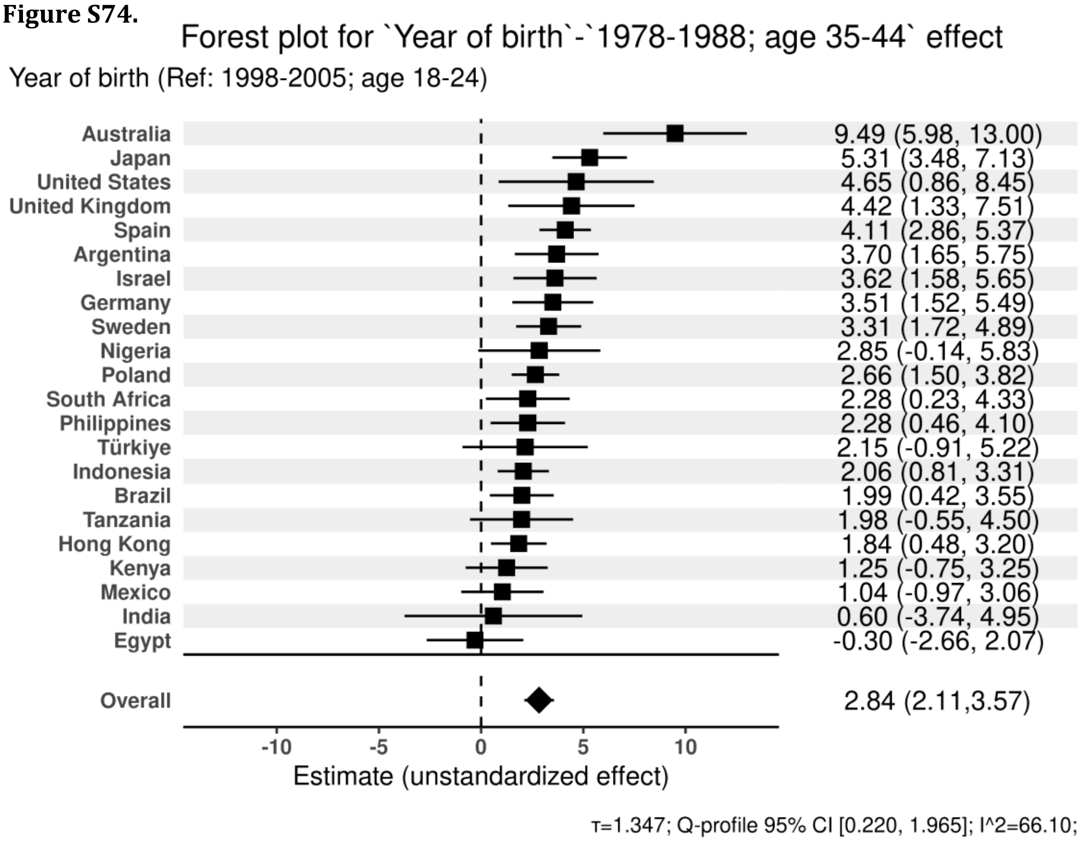

Figure S75.

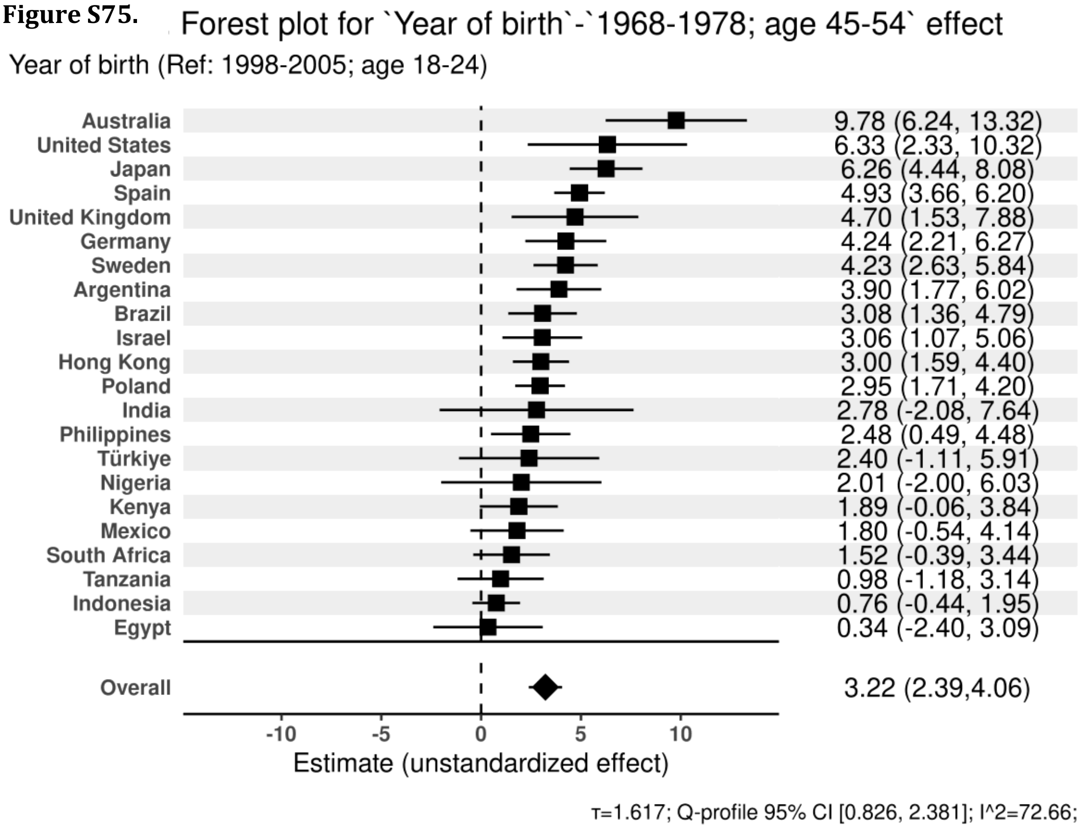

Figure S76.

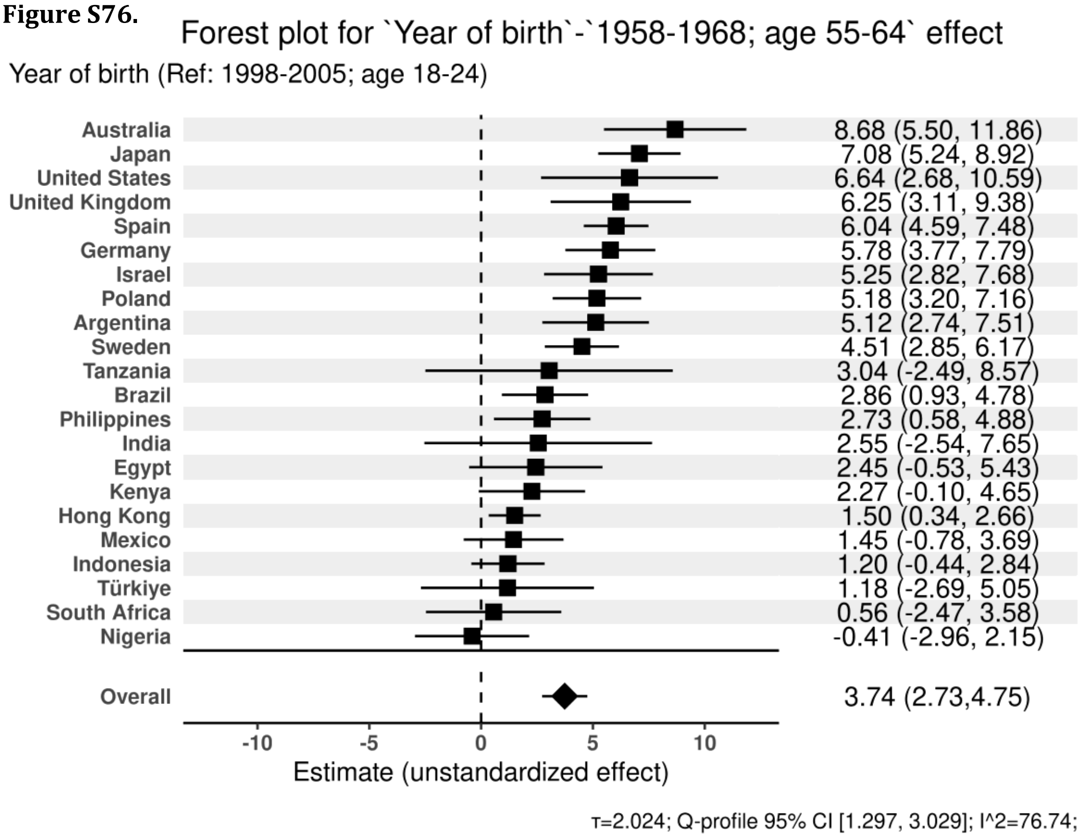

Figure S77.

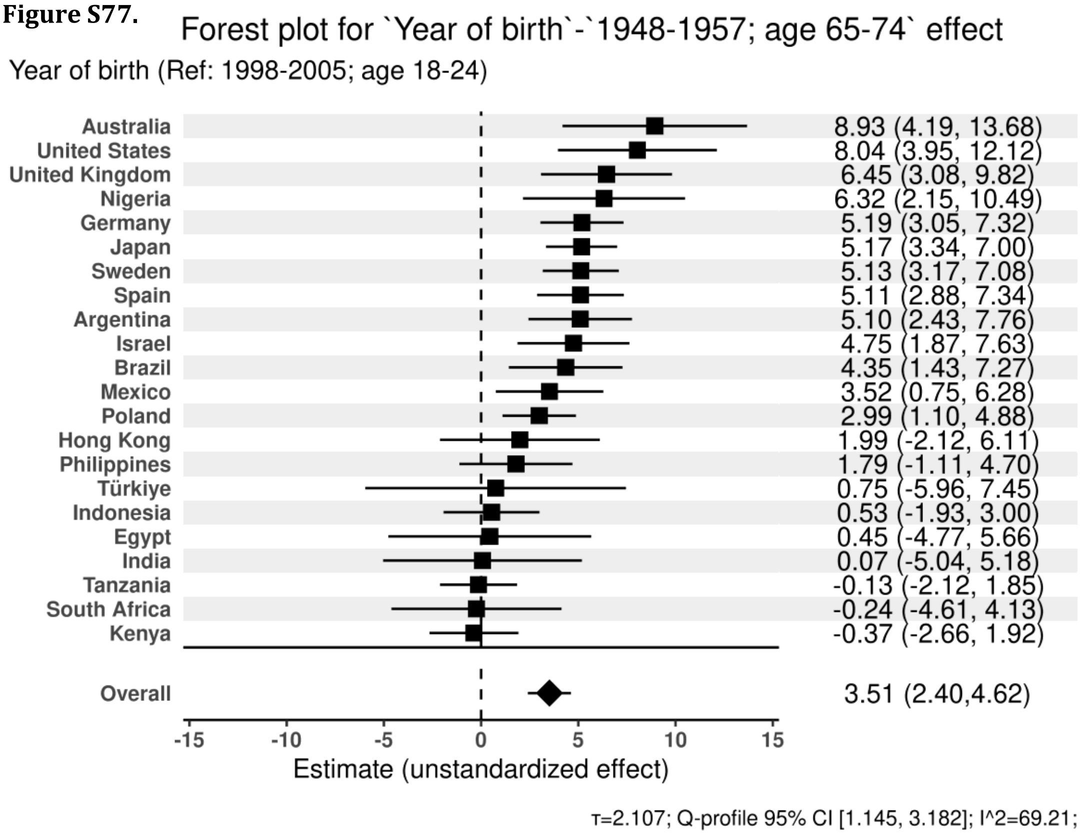

Figure S78.

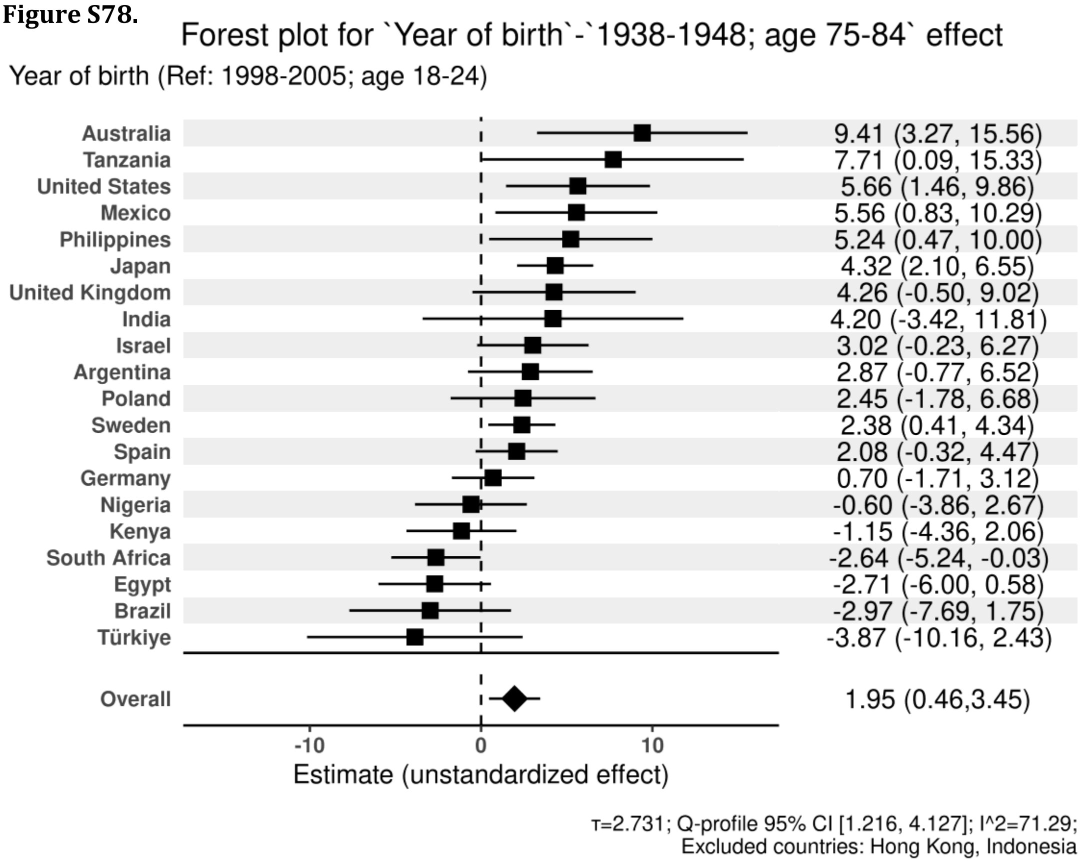

Figure S79.

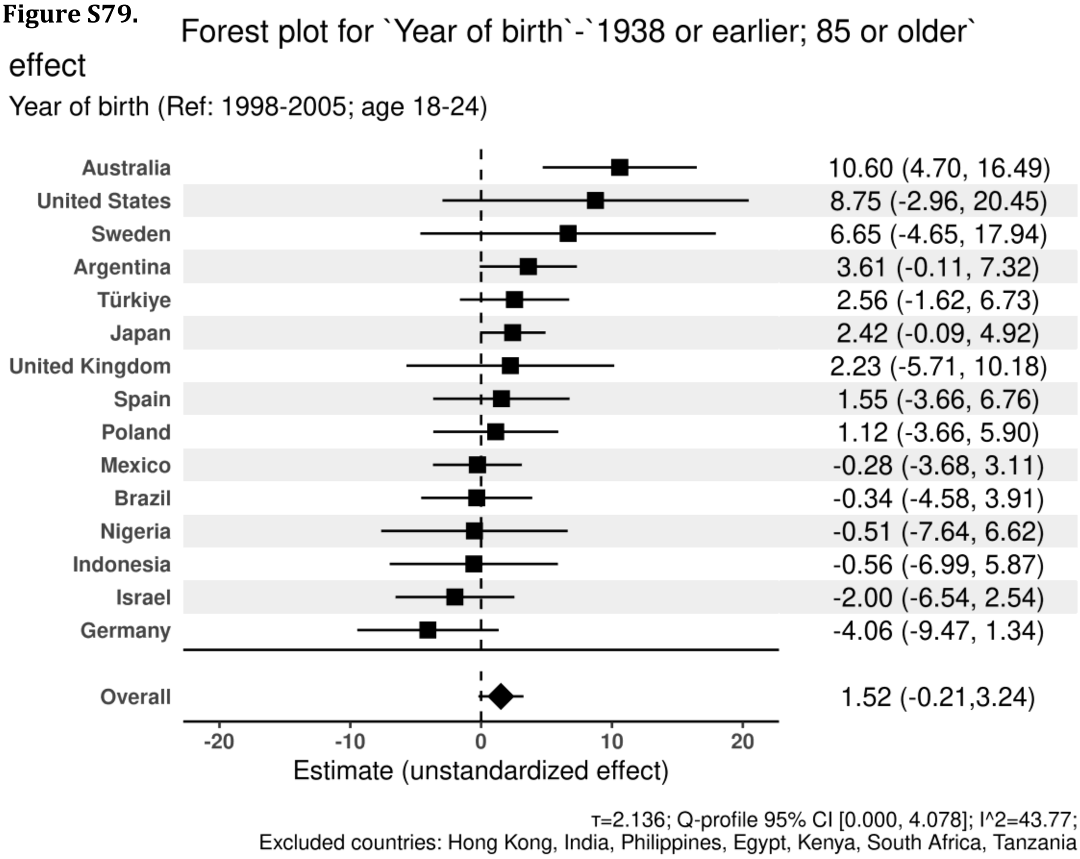

Figure S80.

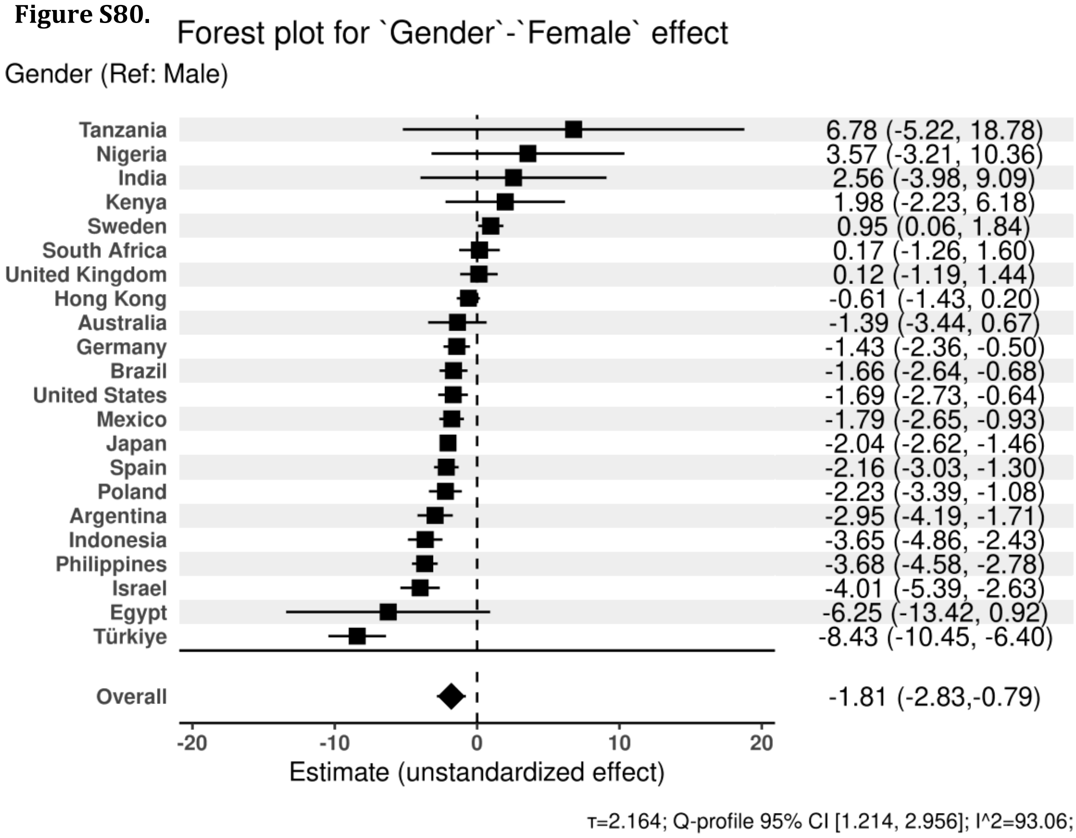

Figure S81.

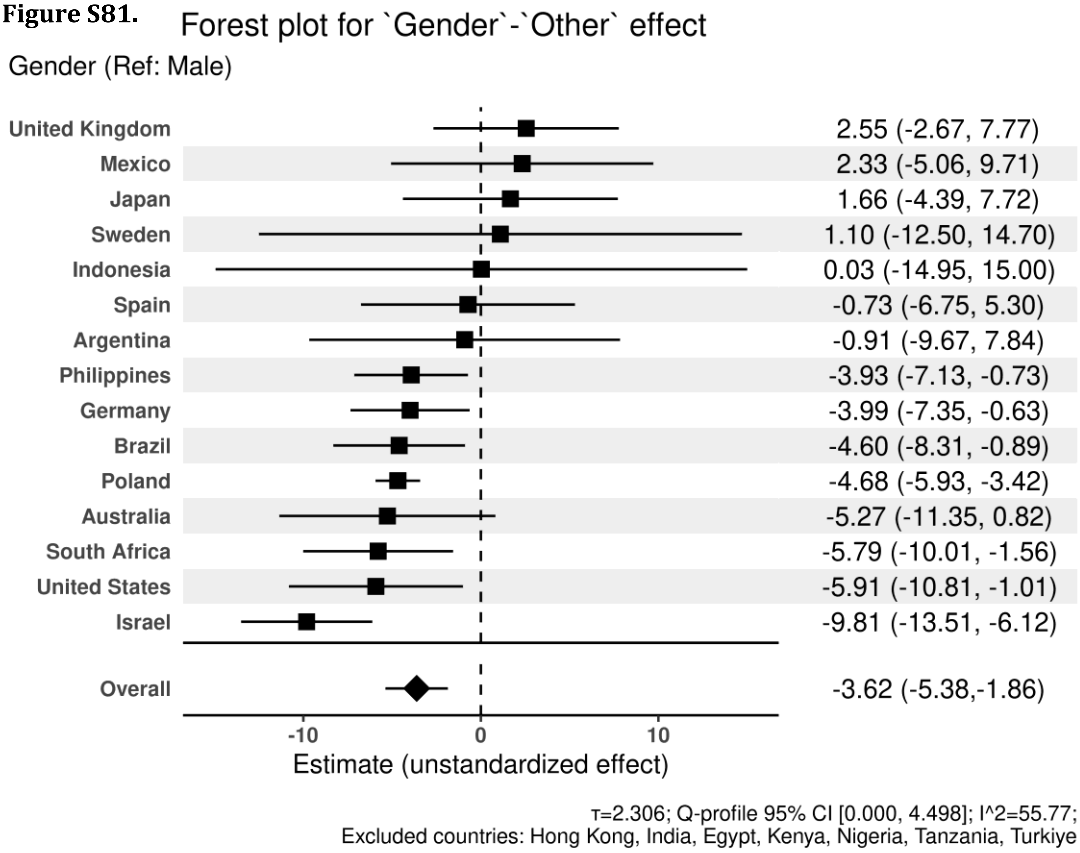

Forest plot for `Gender`-`Other` effect
Gender (Ref: Male)
United Kingdom
Mexico
Japan
Sweden
Indonesia
Spain
Argentina
Philippines
Germany
Brazil
Poland
Australia
South Africa
United States
Israel
Overall
2.55 (-2.67, 7.77)
2.33 (-5.06, 9.71)
1.66 (-4.39, 7.72)
1.10 (-12.50, 14.70)
0.03 (-14.95, 15.00)
-0.73 (-6.75, 5.30)
-0.91 (-9.67, 7.84)
-3.93 (-7.13, -0.73)
-3.99 (-7.35, -0.63)
-4.60 (-8.31, -0.89)
-4.68 (-5.93, -3.42)
-5.27 (-11.35, 0.82)
-5.79 (-10.01, -1.56)
-5.91 (-10.81, -1.01)
-9.81 (-13.51, -6.12)
-3.62 (-5.38,-1.86)
-10
0
10
Estimate (unstandardized effect)
τ=2.306; Q-profile 95% CI [0.000, 4.498]; I^2=55.77;
Excluded countries: Hong Kong, India, Egypt, Kenya, Nigeria, Tanzania, Turkiye
