# Supplementary material for: A cross-national analysis of childhood predictors of daily smoking in adulthood
Source: Commun Med (Lond). 2025 Jul 12;5:292. doi: 10.1038/s43856-025-01005-3 (PMC12255748; doi:10.1038/s43856-025-01005-3)
Supplement: Supplementary file 3 — Reporting Summary [file 43856_2025_1005_MOESM3_ESM.pdf]

nature portfolio

Corresponding author(s): DAPR COMMSMED-24-0813

Last updated by author(s): May 30, 2025

Reporting Summary

Nature Portfolio wishes to improve the reproducibility of the work that we publish. This form provides structure for consistency and transparency in reporting. For further information on Nature Portfolio policies, see our Editorial Policies and the Editorial Policy Checklist.

Statistics

For all statistical analyses, confirm that the following items are present in the figure legend, table legend, main text, or Methods section.

n/a	Confirmed	
☐	☒	The exact sample size (n) for each experimental group/condition, given as a discrete number and unit of measurement
☐	☒	A statement on whether measurements were taken from distinct samples or whether the same sample was measured repeatedly
☐	☒	The statistical test(s) used AND whether they are one- or two-sided<br>*Only common tests should be described solely by name; describe more complex techniques in the Methods section.*
☐	☒	A description of all covariates tested
☐	☒	A description of any assumptions or corrections, such as tests of normality and adjustment for multiple comparisons
☐	☒	A full description of the statistical parameters including central tendency (e.g. means) or other basic estimates (e.g. regression coefficient) AND variation (e.g. standard deviation) or associated estimates of uncertainty (e.g. confidence intervals)
☐	☒	For null hypothesis testing, the test statistic (e.g. F, t, r) with confidence intervals, effect sizes, degrees of freedom and P value noted<br>*Give P values as exact values whenever suitable.*
☒	☐	For Bayesian analysis, information on the choice of priors and Markov chain Monte Carlo settings
☐	☒	For hierarchical and complex designs, identification of the appropriate level for tests and full reporting of outcomes
☐	☒	Estimates of effect sizes (e.g. Cohen's d, Pearson's r), indicating how they were calculated

Our web collection on statistics for biologists contains articles on many of the points above.

Software and code

Policy information about availability of computer code

Data collection	Data collection was carried out by Gallup, Inc. The custom code is the organization's proprietary property.
Data analysis	All meta-analyses were conducted in R using the metafor package. The custom code for data analysis is available at the Center for Open Science (https://osf.io/vbype/).

For manuscripts utilizing custom algorithms or software that are central to the research but not yet described in published literature, software must be made available to editors and reviewers. We strongly encourage code deposition in a community repository (e.g. GitHub). See the Nature Portfolio guidelines for submitting code & software for further information.

Data

Policy information about availability of data

All manuscripts must include a data availability statement. This statement should provide the following information, where applicable:

- Accession codes, unique identifiers, or web links for publicly available datasets
- A description of any restrictions on data availability
- For clinical datasets or third party data, please ensure that the statement adheres to our policy

The data are publicly available through the Center for Open Science (https://osf.io/sm4cd/).

Research involving human participants, their data, or biological material

Policy information about studies with human participants or human data. See also policy information about sex, gender (identity/presentation), and sexual orientation and race, ethnicity and racism.

Reporting on sex and gender	Findings apply to gender that has response categories of "male," "female," and "other." Gender was considered in study design and determined based on self-reporting. Informed consent was obtained for sharing individual-level data. The sample (n = 202,898) consists of 98,411 males, 103,488 females, and 602 "other"s with 397 missing on gender. While gender was included in meta-analysis, no gender-specific analysis was performed, as it was beyond the scope of our study.
Reporting on race, ethnicity, or other socially relevant groupings	Data on race/ethnicity were collected in 19 of 22 countries participating in the study, and response categories were unique to each country (see Tables S1a to S22a of the on-line supplement). Race/Ethnicity was determined based on self-reporting, as participants were asked to identify themselves by selecting one of response categories provided in the survey. Race/ Ethnicity was not used as a proxy for other socially constructed/relevant variables, such as socioeconomic status.
Population characteristics	Population characteristics can are reported in Table 1 and Table S73 of the on-line supplement.
Recruitment	Gallup, Inc. carried out data collection, and the sampling design varied by country and weighted separately to ensure nationally representative sample within each county. Descriptions of how participants were recruited are provided in Ritter et al. (2024) and Padgett et al. (in press) cited in the manuscript.
Ethics oversight	Gallup, Inc.

Note that full information on the approval of the study protocol must also be provided in the manuscript.

Field-specific reporting

Please select the one below that is the best fit for your research. If you are not sure, read the appropriate sections before making your selection.

☐ Life sciences ☒ Behavioural & social sciences ☐ Ecological, evolutionary & environmental sciences

For a reference copy of the document with all sections, see nature.com/documents/nr-reporting-summary-flat.pdf

Behavioural & social sciences study design

All studies must disclose on these points even when the disclosure is negative.

Study description	Quantitative cross-sectional study of childhood predictors of daily cigarette consumption in adulthood, using retrospective data collected in 22 culturally and geographically diverse countries.
Research sample	Research sample consists of 202,898 adults (18 or older), weighted to be nationally representative of 22 countries: 13% age 18-24, 21% age 25-34, 18% age 35-44, 16% age 45-54, 14% age 55-64, 12% age 65-74, 4.3% age 75-84, and 0.7% age 85+; 49% male, 51% female, and 0.3% "other" gender; and 94% native-born and 5% foreign-born (see Table 1).
Sampling strategy	Random sampling, while different sampling schemes were used across countries based on the availability of existing panels and recruitment needs (see Padgett et al., in press, and Ritter et al., 2024, cited in the manuscript, for details)
Data collection	The Global Flourishing Study (GFS) is a study of 202,898 participants from 22 geographically and culturally diverse countries, with nationally representative sampling within each country, concerning the distribution of determinants of well-being. Wave 1 of the GFS data included the following countries and territories: Argentina, Australia, Brazil, Egypt, Germany, Hong Kong, India, Indonesia, Israel, Japan, Kenya, Mexico, Nigeria, the Philippines, Poland, South Africa, Spain, Sweden, Tanzania, Turkey, United Kingdom, and the United States. The countries were selected to (a) maximize coverage of the world's population, (b) ensure geographic, cultural, and religious diversity, and (c) prioritize feasibility and existing data collection infrastructure. Data collection was carried out by Gallup Inc. Data for Wave 1 were collected principally during 2023, with some countries beginning data collection in 2022 and exact dates varying by country. Four additional waves of panel data on the participants will be collected annually from 2024-2027. The precise sampling design to ensure nationally representative samples varied by country and further details are available in Ritter et al. (2024).
Timing	Data were collected principally in 2023, while some countries began data collection in 2022 (see Ritter et al., 2024 for details).
Data exclusions	No data were excluded.
Non-participation	N/A
Randomization	N/A

Reporting for specific materials, systems and methods

We require information from authors about some types of materials, experimental systems and methods used in many studies. Here, indicate whether each material, system or method listed is relevant to your study. If you are not sure if a list item applies to your research, read the appropriate section before selecting a response.

Materials & experimental systems

n/a	Involved in the study
☒	☐ Antibodies
☒	☐ Eukaryotic cell lines
☒	☐ Palaeontology and archaeology
☒	☐ Animals and other organisms
☒	☐ Clinical data
☒	☐ Dual use research of concern
☒	☐ Plants

Methods

n/a	Involved in the study
☒	☐ ChIP-seq
☒	☐ Flow cytometry
☒	☐ MRI-based neuroimaging

Plants

Seed stocks	N/A
Novel plant genotypes	N/A
Authentication	N/A
